# Supplementary material for: Oxidative cyclo-rearrangement of helicenes into chiral nanographenes
Source: Nat Commun. 2021 May 13;12:2786. doi: 10.1038/s41467-021-22992-6 (PMC8119938; doi:10.1038/s41467-021-22992-6)
Supplement: Supplementary file 1 — Supplementary Information [file 41467_2021_22992_MOESM1_ESM.pdf]

Supplementary Information

Oxidative Cyclo-Rearrangement of Helicenes into Chiral Nanographenes

Chengshuo Shen[1], Guoli Zhang[1], Yongle Ding[1], Na Yang[1], Fuwei Gan[1], Jeanne Crassous[2], Huibin Qiu[1,*]

[1]School of Chemistry and Chemical Engineering, Frontiers Science Center for Transformative Molecules, State Key Laboratory of Metal Matrix Composites, Shanghai Jiao Tong University, Shanghai, 200240, China.
[2]Univ Rennes, Institut des Sciences Chimiques de Rennes, UMR CNRS 6226, Campus de Beaulieu, Rennes 35042, France.

Table of Contents

1. Supplementary Methods 2
1.1 General Information 2
1.2 Synthesis of Helicene Substrates 2
1.3 Optimization of Oxidative Cyclo-Rearrangement Reactions 9
1.4 Oxidative Cyclo-Rearrangement and Cyclization of Helicenes 11
1.5 Synthesis of More Complicated Chiral Nanographenes 21
1.6 NMR Spectra 27
1.7 Crystal Structures 58
1.8 Chiral SFC Analysis 98
2. Optical and Chiroptical Spectroscopy 100
2.1 General Information 100
2.2 Additional Optical Spectra 100
3. Theoretical Calculations 103
3.1 General Information 103
3.2 Calculations on Free Energies 104
3.3 Calculated UV-vis Absorption and ECD Spectra 112
Supplementary References 130

1. Supplementary Methods

1.1 General Information

All the synthetic experiments were performed using standard Schlenk techniques unless otherwise stated. Starting materials and reagents were of AR grade quality and were purchased from commercial sources and used without further purification unless otherwise noted. NMR spectra were recorded on a Bruker Avance III HD 500 Spectrometer or a Bruker Avance Neo 700 Spectrometer. Chemical shifts were determined using residual signals of the deuterated solvents or using TMS as the internal standard and were reported in parts per million (ppm). High-resolution mass spectrometry (HR-MS) data were recorded on a Bruker SolariX 7.0T FT-ICR Mass Spectrometer using matrix-assisted laser desorption/ionization (MALDI) mode coupled to a Fourier-transform ion cyclotron resonance (FT-ICR) mass analyzer. Single crystal data were recorded on a Bruker D8 Venture Single Crystal X-Ray Diffractometer with graphite-monochromatic Cu K_α radiation (λ = 1.54178 Å) or Mo K_α radiation (λ = 0.71073 Å).

2-bromo[6]helicene, 2-formyl[6]helicene, 2-(1,2,2-triphenylvinyl)[6]helicene, 1-bromo-perylene-3,4,9,10-tetracarboxylic diimides (1-bromo-PDI) were synthesized according to the literatures.[1–3]

Preparative separation of the two enantiomers of carbo[8]helicene **8H**, carbo[9]helicene **9H** and 4-aza[8]helicene **8Ha** were performed by Daicel Separation Services with the enantiomeric excess (*e.e.*) values of the final products over 99%. The absolute configurations were determined by ECD spectra with comparison to the literature.[4]

1.2 Synthesis of Helicene Substrates

General Method

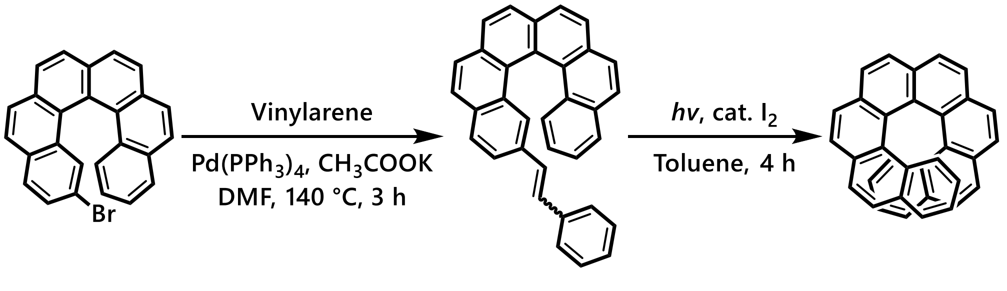

1.22 g of 2-bromo[6]helicene (3.0 mmol), 0.98 g of CH_3COOK (10 mmol) and 346 mg of $Pd(PPh_3)_4$ (10 mol %) were suspended in 10 mL of dried DMF under argon with vigorous stirring, and then 6.0 mmol of vinylarene (2 equiv.) was added into the suspension within 5 min. The mixture was then stirred at 140 °C for 3 hours and the resulting suspension was cooled to room temperature and poured into 50 mL of water. The mixture was extracted by CH_2Cl_2, and the organic layer was washed by water twice and dried over Na_2SO_4. The solvent was then removed at reduced pressure, and the

residue was purified by flash silica column chromatography (CH_2Cl_2 or ethyl acetate) to afford the corresponding 2-arylvinyl[6]helicene as a mixture of *cis* and *trans* isomers. Without further purification, the crude 2-arylvinyl[6]helicene was then dissolved in toluene with a concentration of 1 mmol·L^{-1}, and a catalytic amount of I_2 was added into the solution. The mixture was irradiated by a high-pressure mercury lamp (900 W) for 4 hours. Afterwards, the solvent was removed, and the residue was purified by silica column chromatography to afford the corresponding helicene.

[8]helicene 8H

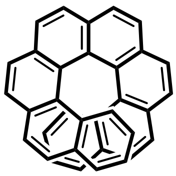

Starting from 1.22 g of 2-bromo[6]helicene (3.0 mmol) and 0.62 g of styrene (6.0 mmol), 1.02 g of crude 2-styryl[6]helicene (a mixture of *cis* and *trans* isomers) was obtained as paled yellow oil (79%). After the irradiation and purification, 348 mg of **8H** was obtained as yellow solid (34%). ^{1}H NMR (500 MHz, $CDCl_3$, 298 K): δ 8.02 (d, *J* = 8.2 Hz, 2H), 7.98 (d, *J* = 8.2 Hz, 2H), 7.79 (d, *J* = 8.1 Hz, 2H), 7.42 (d, *J* = 8.1 Hz, 2H), 7.30 (d, *J* = 7.9 Hz, 2H), 7.15 (d, *J* = 8.5 Hz, 2H), 7.08 (d, *J* = 8.5 Hz, 2H), 7.03 (d, *J* = 8.5 Hz, 2H), 6.97 (ddd, *J* = 7.9 Hz, *J* = 6.9 Hz, *J* = 1.1 Hz, 2H), 6.39 (ddd, *J* = 8.5 Hz, *J* = 6.9 Hz, *J* = 1.4 Hz, 2H). ^{13}C{^{1}H} NMR (126 MHz, $CDCl_3$, 298 K): δ 132.14, 131.75, 130.96, 130.91, 127.97, 127.96, 127.11, 127.03, 126.77, 126.72, 126.69, 126.61, 126.31, 125.66, 125.27, 124.59, 123.78, 123.58. MALDI-HR-MS for $C_{34}H_{20}^{\cdot+}$ ($M^{\cdot+}$): *m/z* = 428.15595 (calculated), 428.15675 (found).

[9]helicene 9H

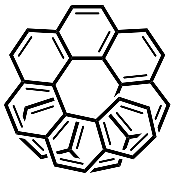

Starting from 1.22 g of 2-bromo[6]helicene (3.0 mmol) and 0.92 g of 2-vinylnaphthalene (6.0 mmol), 1.29 g of crude 2-((2-naphthyl)vinyl)[6]helicene (a mixture of *cis* and *trans* isomers) was obtained as paled yellow oil (89%). After the irradiation and purification, 596 mg of **9H** was obtained as yellow solid (47%). Single crystals suitable for X-ray diffraction were prepared by slow diffusion of pentane into a solution of **9H** in CH_2Cl_2. ^{1}H NMR (500 MHz, $CDCl_3$, 298 K): δ 7.99 (s, 2H), 7.83 (d, *J* = 8.1 Hz, 2H), 7.46 (d, *J* = 8.1 Hz, 2H), 7.36 (d, *J* = 7.9 Hz, 2H), 7.30 (d, *J* = 8.5 Hz, 2H), 7.18 (d, *J* = 8.5 Hz, 2H), 7.11 (d, *J* = 8.5 Hz, 2H), 7.09 (d, *J* = 8.2 Hz, 2H), 7.06 (d, *J* = 8.2 Hz, 2H), 7.00 (ddd, *J* = 7.9 Hz, *J* = 6.8 Hz, *J* = 1.1 Hz, 2H), 6.39 (ddd, *J* = 8.5 Hz, *J* = 6.8 Hz, *J* = 1.3 Hz, 2H). ^{13}C{^{1}H} NMR

(126 MHz, $CDCl_3$, 298 K): δ 132.22, 132.17, 131.05, 130.18, 127.48, 127.12, 126.86, 126.85, 126.67, 126.64, 126.38, 126.30, 126.11, 125.94, 125.55, 124.97, 124.60, 123.47, 123.06. MALDI-HR-MS for $C_{38}H_{22}^{\cdot+}$ ($M^{\cdot+}$): *m*/*z* = 478.17160 (calculated), 478.17360 (found).

4-methyl[8]helicene 8H$_{CH3}$

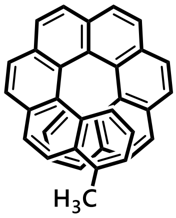

Starting from 1.22 g of 2-bromo[6]helicene (3.0 mmol) and 0.71 g of 1-methyl-2-vinylbenzene (6.0 mmol), 0.88 g of crude 2-(2-methylstyryl)[6]helicene (a mixture of *cis* and *trans* isomers) was obtained as paled yellow oil (66%). After the irradiation and purification, 362 mg of 4-methyl[8]helicene was obtained as yellow solid (41%). ^{1}H NMR (500 MHz, $CDCl_3$, 298 K): δ 8.01 (d, *J* = 8.2 Hz, 1H), 8.00 (d, *J* = 8.2 Hz, 1H), 7.97 (d, *J* = 8.2 Hz, 1H), 7.95 (d, *J* = 8.2 Hz, 1H), 7.78 (d, *J* = 8.1 Hz, 1H), 7.76 (d, *J* = 8.1 Hz, 1H), 7.41 (d, *J* = 8.1 Hz, 1H), 7.39 (d, *J* = 8.1 Hz, 1H), 7.33 (d, *J* = 8.7 Hz, 1H), 7.30 (d, *J* = 7.9 Hz, 1H), 7.19 (d, *J* = 8.5 Hz, 1H), 7.09 (d, *J* = 8.5 Hz, 1H), 7.07 (d, *J* = 8.7 Hz, 1H), 7.05 (d, *J* = 8.3 Hz, 1H), 6.97 (ddd, *J* = 7.9 Hz, *J* = 6.9 Hz, *J* = 1.0 Hz, 1H), 6.91 (d, *J* = 8.5 Hz, 1H), 6.79 (d, *J* = 6.9 Hz, 1H), 6.40 (ddd, *J* = 8.4 Hz, *J* = 6.9 Hz, *J* = 1.2 Hz, 1H), 6.29 (dd, *J* = 8.3 Hz, *J* = 6.9 Hz, 1H), 2.50 (s, 3H). $^{13}C\{^1H\}$ NMR (126 MHz, $CDCl_3$, 298 K): δ 132.58, 132.15, 132.05, 131.93, 130.85, 130.80, 130.53, 130.34, 128.20, 127.90, 127.85, 127.68, 127.02, 127.00, 126.99, 126.84, 126.75, 126.74, 126.61, 126.28, 126.23, 125.82, 125.76, 125.58, 125.43, 125.35, 124.99, 124.53, 123.68, 123.60, 123.36, 122.70, 122.66, 19.89. MALDI-HR-MS for $C_{35}H_{22}^{\cdot+}$ ($M^{\cdot+}$): *m*/*z* = 442.17160 (calculated), 442.17215 (found).

4-trifluoromethyl[8]helicene 8H$_{CF3}$

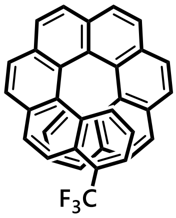

Starting from 1.22 g of 2-bromo[6]helicene (3.0 mmol) and 0.71 g of 1-trifluoromethyl-2-vinylbenzene (6.0 mmol), 1.13 g of crude 2-(2-trifluoromethylstyryl)[6]helicene (a mixture of *cis* and *trans* isomers) was obtained as paled yellow oil (76%). After the irradiation and purification, 543 mg of **8H$_{CF3}$** was obtained as yellow solid (49%). ^{1}H NMR (500 MHz, $CDCl_3$, 298 K): δ 8.05 (d, *J* = 8.2 Hz, 1H), 8.02 (d, *J* = 8.2 Hz, 1H), 7.99 (d, *J* = 8.2 Hz, 1H), 7.98 (d, *J* = 8.2 Hz, 1H), 7.85 (d, *J* = 8.1 Hz, 1H), 7.78 (d, *J* = 8.1 Hz, 1H), 7.48 (dq, *J* = 8.9 Hz, $J_{H\text{-}F}$ = 1.8 Hz, 1H), 7.43 (d, *J* = 8.1 Hz, 1H),

7.40 (d, $J = 8.1$ Hz, 1H), 7.33 (d, $J = 7.9$ Hz, 1H), 7.33 (d, $J = 7.4$ Hz, 1H), 7.29 (d, $J = 8.5$ Hz, 1H), 7.21 (d, $J = 8.4$ Hz, 1H), 7.19 (d, $J = 8.9$ Hz, 1H), 7.07 (d, $J = 8.5$ Hz, 1H), 7.02 (d, $J = 8.4$ Hz, 1H), 6.99 (ddd, $J = 7.9$ Hz, $J = 6.9$ Hz, $J = 1.1$ Hz, 1H), 6.40 (dd, $J = 8.4$ Hz, $J = 7.4$ Hz, 1H), 6.39 (ddd, $J = 8.4$ Hz, $J = 6.9$ Hz, $J = 1.2$ Hz, 1H). $^{13}C\{^1H\}$ NMR (126 MHz, $CDCl_3$, 298 K): δ 132.38, 132.29, 131.90, 131.11, 130.95, 130.40, 128.63, 128.07 (q, $J_{C\text{-}F} = 0.8$ Hz), 127.62, 127.61, 127.37, 127.35, 127.32, 127.30, 127.22, 127.11, 127.00, 126.77, 126.59, 126.51, 126.45, 125.44, 125.11, 125.05, 124.95 (q, $J_{C\text{-}F} = 273.6$ Hz), 124.77, 124.66 (q, $J_{C\text{-}F} = 30.0$ Hz), 123.94, 123.54, 123.00 (q, $J_{C\text{-}F} = 6.2$ Hz), 122.13 (q, $J_{C\text{-}F} = 2.6$ Hz), 121.91. $^{19}F\{^1H\}$ NMR (471 MHz, $CDCl_3$, 298 K): δ −58.9. MALDI-HR-MS for $C_{35}H_{19}F_3^{\cdot+}$ ($M^{\cdot+}$): m/z = 496.14334 (calculated), 496.14422 (found).

4-fluoro[8]helicene 8H$_F$

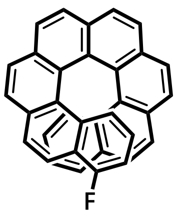

Starting from 1.22 g of 2-bromo[6]helicene (3.0 mmol) and 0.81 g of 1-fluoro-2-vinylbenzene (6.0 mmol), 0.93 g of crude 2-(2-fluorostyryl)[6]helicene (a mixture of *cis* and *trans* isomers) was obtained as paled yellow oil (69%). After the irradiation and purification, 344 mg of **8H$_F$** was obtained as yellow solid (37%). Single crystals suitable for X-ray diffraction were prepared by slow diffusion of pentane into a solution of **8H$_F$** in CH_2Cl_2. ^1H NMR (500 MHz, $CDCl_3$, 298 K): δ 8.03 (d, $J = 8.2$ Hz, 1H), 8.02 (d, $J = 8.2$ Hz, 1H), 7.98 (d, $J = 8.2$ Hz, 2H), 7.82 (d, $J = 8.1$ Hz, 1H), 7.79 (d, $J = 8.1$ Hz, 1H), 7.46 (d, $J = 8.7$ Hz, 1H), 7.44 (d, $J = 8.1$ Hz, 1H), 7.43 (d, $J = 8.1$ Hz, 1H), 7.33 (d, $J = 7.9$ Hz, 1H), 7.26 (d, $J = 8.5$ Hz, 1H), 7.16 (d, $J = 8.5$ Hz, 1H), 7.14 (d, $J = 8.7$ Hz, 1H), 7.04 (d, $J = 8.4$ Hz, 1H), 6.99 (ddd, $J = 7.9$ Hz, $J = 6.9$ Hz, $J = 1.0$ Hz, 1H), 6.81 (d, $J = 8.5$ Hz, 1H), 6.66 (ddd, $J_{H\text{-}F} = 10.3$ Hz, $J = 7.7$ Hz, $J = 0.7$ Hz, 1H), 6.40 (ddd, $J = 8.4$ Hz, $J = 6.9$ Hz, $J = 1.2$ Hz, 1H), 6.32 (ddd, $J = 8.5$ Hz, $J = 7.7$ Hz, $J_{H\text{-}F} = 6.0$ Hz, 1H). $^{13}C\{^1H\}$ NMR (126 MHz, $CDCl_3$, 298 K): δ 158.02 (d, $J_{C\text{-}F} = 156.9$ Hz), 132.32, 132.10, 131.87, 131.04, 131.02, 130.94, 129.65 (d, $J_{C\text{-}F} = 3.6$ Hz), 127.85, 127.67, 127.26 (d, $J_{C\text{-}F} = 2.9$ Hz), 127.23, 127.05, 127.00, 126.94, 126.92, 126.89, 126.66, 126.59, 126.46, 126.41, 125.95, 125.94, 125.67, 125.58, 125.40, 124.67, 123.90, 123.51, 123.39 (d, $J_{C\text{-}F} = 8.6$ Hz), 121.22 (d, $J_{C\text{-}F} = 16.1$ Hz), 119.90 (d, $J_{C\text{-}F} = 3.7$ Hz), 118.49 (d, $J_{C\text{-}F} = 7.2$ Hz), 108.43 (d, $J_{C\text{-}F} = 20.0$ Hz). $^{19}F\{^1H\}$ NMR (471 MHz, $CDCl_3$, 298 K): δ −124.50. MALDI-HR-MS for $C_{34}H_{19}F^{\cdot+}$ ($M^{\cdot+}$): m/z = 446.14653 (calculated), 446.14730 (found).

4-aza[8]helicene 8Ha

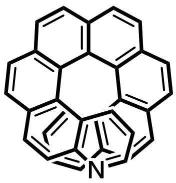

Starting from 1.22 g of 2-bromo[6]helicene (3.0 mmol) and 0.63 g of 2-vinylpyridine (6.0 mmol), 0.96 g of crude 2-((2-pyridyl)vinyl)[6]helicene (a mixture of *cis* and *trans* isomers) was obtained as paled yellow oil (74%). After the irradiation and purification, 342 mg of **8Ha** was obtained as yellow solid (36%). ^{1}H NMR (500 MHz, $CDCl_3$, 298 K): δ 8.36 (dd, J = 4.2 Hz, J = 1.6 Hz, 1H), 8.03 (d, J = 8.2 Hz, 1H), 8.02 (d, J = 8.2 Hz, 1H), 7.99 (d, J = 8.2 Hz, 1H), 7.98 (d, J = 8.2 Hz, 1H), 7.84 (d, J = 8.2 Hz, 1H), 7.82 (d, J = 8.1 Hz, 1H), 7.47 (d, J = 8.1 Hz, 1H), 7.45 (d, J = 8.1 Hz, 1H), 7.43 (d, J = 8.7 Hz, 1H), 7.34 (d, J = 8.1 Hz, 1H), 7.34 (dd, J = 8.4 Hz, J = 1.6 Hz, 1H), 7.33 (d, J = 8.7 Hz, 1H), 7.25 (d, J = 8.5 Hz, 1H), 7.12 (d, J = 8.5 Hz, 1H), 7.00 (d, J = 8.4 Hz, 1H), 6.99 (ddd, J = 8.1 Hz, J = 6.9 Hz, J = 1.1 Hz, 1H), 6.40 (ddd, J = 8.4 Hz, J = 6.9 Hz, J = 1.3 Hz, 1H), 6.31 (dd, J = 8.4 Hz, J = 4.2 Hz, 1H). ^{13}C{^{1}H} NMR (126 MHz, $CDCl_3$, 298 K): δ 148.10, 147.09, 132.24, 131.82, 131.24, 131.20, 131.20, 131.12, 130.62, 129.18, 127.88, 127.74, 127.72, 127.48, 127.41, 127.37, 127.28, 127.24, 127.18, 127.07, 126.97, 126.90, 126.79, 126.52, 126.11, 125.32, 125.10, 125.04, 124.83, 123.96, 123.61, 123.22, 118.23. MALDI-HR-MS for $C_{33}H_{19}N^{\cdot+}$ ($M^{\cdot+}$): m/z = 429.15120 (calculated), 429.15227 (found).

Methylthio[8]helicene 8Hmt

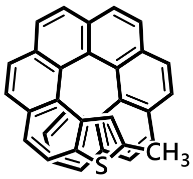

Starting from 407 mg of 2-bromo[6]helicene (1.0 mmol) and 248 mg of 2-methyl-5-vinylthiophene (2.0 mmol), 377 mg of crude 2-((2-pyridyl)vinyl)[6]helicene (a mixture of *cis* and *trans* isomers) was obtained as paled yellow oil (84%). After the irradiation and purification, 131 mg of **8Hmt** was obtained as yellow solid (35%). ^{1}H NMR (500 MHz, $CDCl_3$, 298 K): δ 8.06 (d, J = 8.2 Hz, 1H), 8.05 (d, J = 8.2 Hz, 1H), 8.04 (d, J = 8.1 Hz, 1H), 7.97 (d, J = 8.1 Hz, 1H), 7.92 (d, J = 8.1 Hz, 1H), 7.71 (d, J = 8.3 Hz, 1H), 7.68 (d, J = 8.1 Hz, 1H), 7.47 (d, J = 8.3 Hz, 1H), 7.37 (d, J = 8.5 Hz, 1H), 7.30 – 7.26 (m, 3H), 7.07 (d, J = 8.5 Hz, 1H), 7.00 (d, J = 8.4 Hz, 1H), 6.92 (ddd, J = 7.9 Hz, J = 6.9 Hz, J = 1.1 Hz, 1H), 6.38 (ddd, J = 8.4 Hz, J = 6.9 Hz, J = 1.3 Hz, 1H), 5.63 (quint, J = 1.1 Hz, 1H), 2.05 (d, J = 1.1 Hz, 3H). ^{13}C{^{1}H} NMR (126 MHz, $CDCl_3$, 298 K): δ 137.58, 136.83, 134.77, 132.01, 131.68, 131.67, 131.44, 131.23, 130.22, 128.54, 127.85, 127.65, 127.17, 127.12, 126.83, 126.73, 126.67, 126.60, 126.58, 126.52, 126.29, 126.16, 126.10, 125.96, 125.00, 124.76, 124.67, 123.56,

123.30, 123.01, 121.76, 120.15, 15.67. MALDI-HR-MS for $C_{33}H_{20}S^{·+}$ ($M^{·+}$): *m*/*z* = 44.12802 (calculated), 448.12947 (found).

Thiazo[8]helicene 8H$_{ta}$

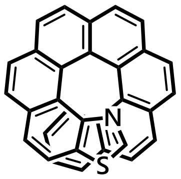

Starting from 407 mg of 2-bromo[6]helicene (1.0 mmol) and 222 mg of 5-vinylthiazole (2.0 mmol), 397 mg of crude 2-((2-pyridyl)vinyl)[6]helicene (a mixture of *cis* and *trans* isomers) was obtained as paled yellow oil (91%). After the irradiation and purification, 174 mg of **8H$_a$** was obtained as yellow solid (44%). ^{1}H NMR (500 MHz, $CDCl_3$, 298 K): δ 8.08 (d, J = 8.2 Hz, 1H), 8.05 (d, J = 8.2 Hz, 1H), 8.03 (d, J = 8.2 Hz, 1H), 7.97 (d, J = 8.2 Hz, 1H), 7.96 (d, J = 8.1 Hz, 1H), 7.94 (s, 1H), 7.82 (d, J = 8.3 Hz, 1H), 7.63 (d, J = 8.1 Hz, 1H), 7.52 (d, J = 8.3 Hz, 1H), 7.43 (d, J = 8.5 Hz, 1H), 7.31 (d, J = 8.5 Hz, 1H), 7.27 (d, J = 7.9 Hz, 1H), 7.27 (d, J = 8.5 Hz, 1H), 7.22 (d, J = 8.5 Hz, 1H), 7.08 (d, J = 8.5 Hz, 1H), 6.93 (ddd, J = 7.9 Hz, J = 6.9 Hz, J = 1.1 Hz, 1H), 6.42 (ddd, J = 8.5 Hz, J = 6.9 Hz, J = 1.3 Hz, 1H). $^{13}C\{^1H\}$ NMR (126 MHz, $CDCl_3$, 298 K): δ 148.96, 148.38, 131.94, 131.78, 131.19, 131.18, 131.14 (2C), 131.13, 128.55, 127.79, 127.33, 127.27, 127.24, 127.12, 127.07, 126.71, 126.57, 126.50, 126.46, 126.31, 126.29, 126.09, 126.07, 125.55, 125.35, 125.18, 124.48, 123.80, 123.60, 118.63. MALDI-HR-MS for $C_{31}H_{17}NS^{·+}$ ($M^{·+}$): *m*/*z* = 435.10762 (calculated), 435.10829 (found).

[7]helicene 7H

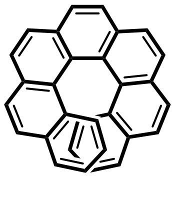

1.01 g of 3,6-dibromophenanthrene (3.0 mmol), 1.96 g of CH_3COOK (20 mmol) and 346 mg of $Pd(PPh_3)_4$ (10 mol %) were suspended in 6 mL of dried DMF under argon with vigorous stirring, and then 1.25 g of styrene (12 mmol, 4 equiv.) was added into the suspension within 5 min. The mixture was then stirred at 140 °C for 3 h and the resulting suspension was cooled to room temperature and poured into 50 mL of water. The mixture was extracted by CH_2Cl_2, and the organic layer was washed by water twice and was dried over Na_2SO_4. The solvent was then removed at reduced pressure, and the residue was purified by flash silica column chromatography (heptane : CH_2Cl_2 = 1 : 1, v/v) to afford crude 3,6-distyrylphenanthrene (a mixture of *cis* and *trans* isomers) as paled yellow solid (0.93 g, 81%). Without further purification, the crude 3,6-distyrylphenanthrene was then dissolved in toluene in a concentration of 1 mmol·L^{-1}, and a catalytic amount of I_2 was added into the solution.

The mixture was irradiated by a high-pressure mercury lamp (900 W) for 4 hours. Afterwards, the solvent was removed, and the residue was purified by silica column chromatography (heptane) to afford **7H** as yellow solid (307 mg, 33%). Single crystals suitable for X-ray diffraction were prepared by slow diffusion of pentane into a solution of **7H** in CH_2Cl_2. Spontaneous resolution was observed during the crystallization, and the structure of one enantiomer was recorded. ^{1}H NMR (500 MHz, $CDCl_3$, 298 K): δ 8.04 (s, 2H), 8.01 (d, J = 8.2 Hz, 2H), 7.95 (d, J = 8.2 Hz, 2H), 7.77 (d, J = 8.4 Hz, 2H), 7.53 (d, J = 8.4 Hz, 2H), 7.34 (d, J = 7.9 Hz, 2H), 7.20 (d, J = 8.5 Hz, 2H), 6.94 (ddd, J = 7.9 Hz, J = 6.8 Hz, J = 1.1 Hz, 2H), 6.39 (ddd, J = 8.5 Hz, J = 6.8 Hz, J = 1.3 Hz, 2H). ^{13}C{^{1}H} NMR (126 MHz, $CDCl_3$, 298 K): δ 132.10, 131.81, 130.88, 129.57, 128.40, 127.58, 127.40, 126.91, 126.72, 125.83, 125.34, 125.07, 124.33, 123.68. MALDI-HR-MS for $C_{30}H_{18}^{\cdot+}$ ($M^{\cdot+}$): m/z = 378.14030 (calculated), 378.13941 (found).

4-bromo[8]helicene 8H$_{\mathbf{Br}}$

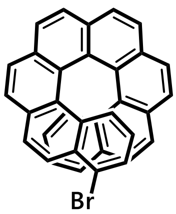

1.03 g of (2-bromobenzyl)triphenylphosphonium bromide (2.0 mmol) was suspended in 20 mL of dried THF under argon and cooled to −78 °C. 1.4 mL of *n*-butyllithium (1.6 mol·L^{-1} in hexane, 2.2 mmol, 1.1 eq.) was added, and the reaction mixture was firstly stirred at −78 °C for 5 min and then at room temperature for 30 min. The reaction mixture turned red during the procedure. Then the reaction was cooled to −78 °C again and 0.71 g of 2-formyl[6]helicene (2.0 mmol) dissolved in 5 mL of dried THF was added dropwise. The reaction was stirred at −78 °C for 5 min then at room temperature for 2 hours. The solvent was removed in vacuum and the residue was purified by flash silica column chromatography (CH_2Cl_2) to afford crude 2-(2-bromostyryl)[6]helicene (a mixture of *cis* and *trans* isomers) as paled yellow solid (0.89 g, 87%). Without further purification, the crude 2-(2-bromostyryl)[6]helicene was then dissolved in toluene in a concentration of 1 mmol·L^{-1}, and catalytic amount of I_2 was added into the solution. The mixture was irradiated by high-pressure mercury lamp (900 W) for 4 hours. Afterwards, the solvent was removed, and the residue was purified by silica column chromatography (heptane : CH_2Cl_2 = 10 : 1, v/v) to afford **8H$_{\mathbf{Br}}$** as yellow solid (505 mg, 57%). ^{1}H NMR (500 MHz, $CDCl_3$, 298 K): δ 8.03 (d, J = 8.2 Hz, 1H), 8.00 (d, J = 8.2 Hz, 1H), 7.97 (d, J = 8.2 Hz, 1H), 7.96 (d, J = 8.2 Hz, 1H), 7.82 (d, J = 8.1 Hz, 1H), 7.77 (d, J = 8.1 Hz, 1H), 7.59 (dd, J = 8.8 Hz, J = 0.5 Hz, 1H), 7.42 (d, J = 8.1 Hz, 2H), 7.35 (d, J = 7.9 Hz, 1H), 7.30 (d, J = 8.5 Hz, 1H), 7.25 (dd, J = 7.4 Hz, J = 1.0 Hz, 1H), 7.15 (d, J = 8.8 Hz, 1H), 7.15 (d, J = 8.5 Hz, 1H), 7.05 (ddd, J = 8.5 Hz, J = 1.1 Hz, J = 0.5 Hz, 1H), 7.04 (d, J = 8.4 Hz, 1H), 6.99 (ddd, J = 7.9

Hz, J = 6.9 Hz, J = 1.1 Hz, 1H), 6.40 (ddd, J = 8.5 Hz, J = 6.9 Hz, J = 1.3 Hz, 1H), 6.23 (dd, J = 8.4 Hz, J = 7.4 Hz, 1H). $^{13}C\{^1H\}$ NMR (126 MHz, $CDCl_3$, 298 K): δ 132.37, 132.13, 132.02, 130.98, 130.96, 130.86, 129.75, 129.50, 128.34, 127.63, 127.55, 127.49, 127.23, 127.14, 127.10, 127.05, 127.00, 126.97, 126.78, 126.67, 126.63, 126.50, 126.44, 126.39, 125.49, 125.43, 125.31, 125.25, 124.66, 124.02, 123.85, 123.53, 121.79. MALDI-HR-MS for $C_{34}H_{19}{}^{79}Br^{\cdot+}$ ($M^{\cdot+}$): m/z = 506.06646 (calculated), 506.06725 (found).

5,6-diphenyl[8]helicene 8H$_{2py}$

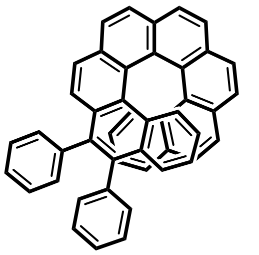

58.3 mg of 2-(1,2,2-triphenylvinyl)[6]helicene (0.1 mmol) was dissolved in 100 mL of toluene (1 mmol·L^{-1}), and catalytic amount of I_2 was added into the solution. The mixture was irradiated by an LED lamp (360 – 370 nm, 20 W) for 1 hour. Afterwards, the solvent was removed, and the residue was purified by silica column chromatography (heptane : CH_2Cl_2 = 10 : 1, v/v) to afford **8H$_{2py}$** as yellow solid (50.2 mg, 86%). ^1H NMR (500 MHz, $CDCl_3$, 298 K): δ 8.05 (d, J = 8.2 Hz, 1H), 8.05 (d, J = 8.2 Hz, 1H), 8.00 (d, J = 8.2 Hz, 1H), 7.98 (d, J = 8.2 Hz, 1H), 7.80 (d, J = 8.1 Hz, 1H), 7.70 (d, J = 8.5 Hz, 1H), 7.63 (dd, J = 8.1 Hz, J = 1.2 Hz, 1H), 7.44 (d, J = 8.2 Hz, 1H), 7.39 (t, J = 7.4 Hz, 1H), 7.38 (d, J = 8.5 Hz, 1H), 7.29 – 7.22 (m, 2H), 7.22 – 7.15 (m, 7H), 7.13 – 7.08 (m, 2H), 7.01 – 6.97 (m, 2H), 6.96 – 6.92 (m, 1H), 6.88 – 6.84 (m, 2H), 6.49 (ddd, J = 8.5 Hz, J = 6.8 Hz, J = 1.3 Hz, 1H), 6.39 (ddd, J = 8.4 Hz, J = 6.7 Hz, J = 1.3 Hz, 1H). $^{13}C\{^1H\}$ NMR (126 MHz, $CDCl_3$, 298 K): δ 139.74, 139.60, 136.34, 135.32, 132.36, 131.71, 131.68, 131.66, 131.63, 131.31, 131.08, 131.01, 130.82, 130.21, 130.15, 128.13, 127.77, 127.72, 127.55, 127.45, 127.32, 127.31, 127.27, 127.15, 127.14, 127.11, 126.93, 126.89, 126.68, 126.66, 126.39, 126.33, 126.32, 126.24, 125.91, 125.86, 125.68, 125.64, 125.41, 125.19, 124.78, 124.61, 124.00, 123.95, 123.63. MALDI-HR-MS for $C_{46}H_{28}{}^{\cdot+}$ ($M^{\cdot+}$): m/z = 580.21855 (calculated), 580.22134 (found).

1.3 Optimization of Oxidative Cyclo-Rearrangement Reactions

Typically, 0.05 mmol of primitive carbo[6]helicene **6H** or cabro[8]helicene **8H** and 2,3-dichloro-5,6-dicyano-1,4-benzoquinone (DDQ) (0.06 mmol, 1.2 equiv.) were suspended in 20 mL of dried CH_2Cl_2 under argon at 20 °C, and 0.1 mL of a strong acid (CF_3COOH, CH_3SO_3H or CF_3SO_3H) was added to the suspension dropwise with vigorous stirring. The reaction was stirred for 30 min and then quenched by 5 mL of saturated $NaHCO_3$ solution. The mixture was extracted by CH_2Cl_2, and the organic layer

was washed by water twice and dried over Na_2SO_4. The solvent was removed at reduced pressure, and the residue was examined by silica thin-layer chromatography (TLC) using heptane/CH_2Cl_2 (5/1, v/v) as the eluant. Briefly, the oxidation of **6H** gave rise to a string of unidentifiable spots with multicolor luminescence when CH_3SO_3H or CF_3SO_3H was used, while **8H** yielded only one green luminescent spot (later was proven to be **O8H**) in the presence of CF_3SO_3H (Supplementary Table 1).

Supplementary Table 1. Screening of reaction condition for the oxidative cyclo-rearrangement of **6H** and **8H** using TLC. Dot 1 for the raw product and dot 2 for the starting helicene.

	**6H**	**8H**
CF_3COOH	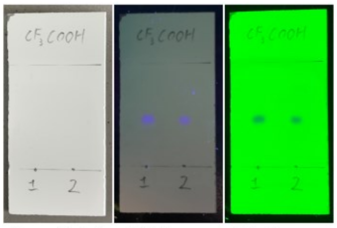<br>Sunlight 365 nm 254 nm<br>No reaction	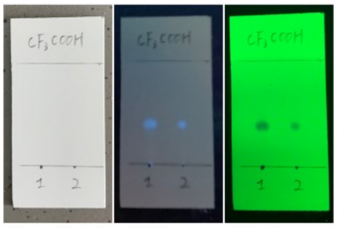<br>Sunlight 365 nm 254 nm<br>No reaction
CH_3SO_3H	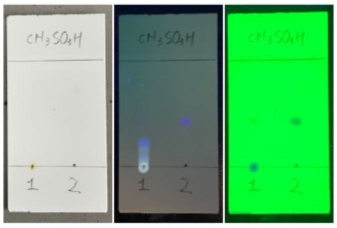<br>Sunlight 365 nm 254 nm<br>A string of unidentifiable products	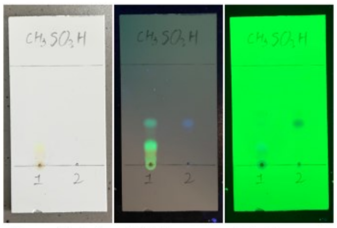<br>Sunlight 365 nm 254 nm<br>A string of unidentifiable products
CF_3SO_3H	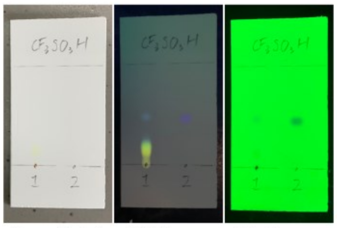<br>Sunlight 365 nm 254 nm<br>A string of unidentifiable products	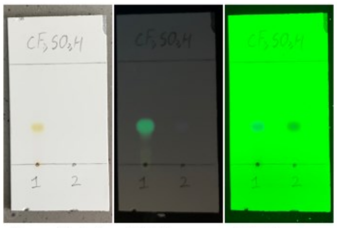<br>Sunlight 365 nm 254 nm<br>Mainly **O8H**

In addition, we have also investigated the effect of temperature, reaction time and solvent on the oxidative cyclo-rearrangement (Supplementary Table 2). Generally, (i) in the presence of CF_3SO_3H, the reaction underwent well in 0 °C but required a relatively longer reaction time (Entry 1 vs. Entry 2); (ii) in the presence of CH_3SO_3H, a larger amount of DDQ (5 equiv.) along with a lower temperature substantially enhanced the yield of **O8H** (Entry 3 vs. Entry 5), but the helicene precursor mostly decomposed at a higher reaction temperature or upon a long reaction time (Entries 4 and 6); (iii) the reaction also underwent well in $CHCl_3$ and CH_3NO_2 (Entries 7 and 8).

Supplementary Table 2. Oxidative cyclo-rearrangement of **8H** under various conditions.

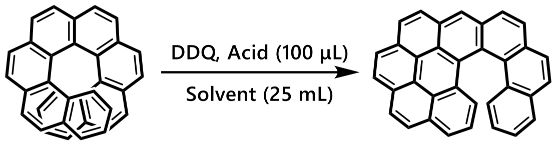

8H **O8H**

Entry	Acid	DDQ	Temperature	Time	Solvent	Yield[a]
1	CF_3SO_3H	1.2 equiv.	25 °C	30 min	CH_2Cl_2	49%
2	CF_3SO_3H	1.2 equiv.	0 °C	12 h	CH_2Cl_2	48%
3	CH_3SO_3H	1.2 equiv.	25 °C	30 min	CH_2Cl_2	Trace of **O8H** with a string of unidentifiable products
4	CH_3SO_3H	5 equiv.	25 °C	30 min	CH_2Cl_2	Mostly decomposed
5	CH_3SO_3H	5 equiv.	−20 °C	30 min	CH_2Cl_2	41%
6	CH_3SO_3H	5 equiv.	−20 °C	12 h	CH_2Cl_2	Trace of **O8H**, mostly decomposed
7	CF_3SO_3H	1.2 equiv.	25 °C	30 min	$CHCl_3$	39%
8	CF_3SO_3H	1.2 equiv.	25 °C	30 min	CH_3NO_2	38%

[a]Isolated yield after purification by column chromatography on silica gel.

1.4 Oxidative Cyclo-Rearrangement and Cyclization of Helicenes

General method

0.05 mmol of helicene substrate and 13.6 mg of 2,3-dichloro-5,6-dicyano-1,4-benzoquinone (DDQ) (0.06 mmol, 1.2 equiv.) were suspended in 20 mL of dried CH_2Cl_2 under argon at 20 °C, and 0.1 mL of CF_3SO_3H was added to the suspension dropwise with vigorous stirring. The reaction was stirred for 30 min and then quenched by 5 mL of saturated $NaHCO_3$ solution. The mixture was extracted by CH_2Cl_2, and the organic layer was washed by water twice and dried over Na_2SO_4. The solvent was removed at reduced pressure, and the residue was purified by silica column chromatography (heptane/CH_2Cl_2 = 10/1, v/v) to afford the corresponding nanographene.

Phenanthro[3,4,5,6-*defgh*][5]helicene O7H

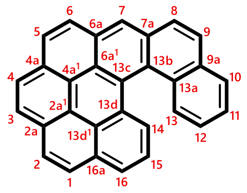

Starting from 18.9 mg of **7H** (0.05 mmol), 10.1 mg of **O7H** was obtained as orange solid (54%). Single crystals suitable for X-ray diffraction were prepared by slow diffusion of pentane into a solution of **O7H** in CH_2Cl_2. ^{1}H NMR (500 MHz, $CDCl_3$, 298 K): δ 9.25 (d, J = 8.0 Hz, 1H, *H*-14), 8.94 (d, J = 8.4 Hz, 1H, *H*-13), 8.49 (s, 1H, *H*-7), 8.42 (d, J = 8.1 Hz, 1H, *H*-3), 8.40 (d, J = 8.1 Hz,

1H, *H*-4), 8.17 (d, *J* = 8.8 Hz, 1H, *H*-2), 8.14 (d, *J* = 8.8 Hz, 1H, *H*-6), 8.14 (d, *J* = 7.8 Hz, 1H, *H*-16), 8.13 (d, *J* = 8.8 Hz, 1H, *H*-1), 8.10 (d, *J* = 8.8 Hz, 1H, *H*-5), 8.03 (d, *J* = 8.7 Hz, 1H, *H*-8), 7.92 (dd, *J* = 7.9 Hz, *J* = 1.3 Hz, 1H, *H*-10), 7.90 (d, *J* = 8.7 Hz, 1H, *H*-9), 7.66 (dd, *J* = 8.0 Hz, J = 7.8 Hz, 1H, *H*-15), 7.50 (ddd, *J* = 7.9 Hz, *J* = 6.9 Hz, *J* = 1.1 Hz, 1H, *H*-11), 7.15 (ddd, *J* = 8.4 Hz, *J* = 6.9 Hz, *J* = 1.4 Hz, 1H, *H*-12). $^{13}C\{^1H\}$ NMR (126 MHz, $CDCl_3$, 298 K): δ 132.69 (*C*-9a), 131.81 (*C*-7a), 131.75 (*C*-16a), 131.19 (*C*-13b), 130.36 (*C*-6a), 130.13 (*C*-13d), 129.61 (*C*-2a), 129.28 (*C*-4a), 128.68 (*C*-14), 128.16 (*C*-13 or *C*-10), 128.14 (*C*-13 or *C*-10), 127.72 (*C*-5 or *C*-9), 127.70 (*C*-5 or *C*-9), 127.49 (*C*-1), 127.21 (*C*-2, *C*-6 or *C*-11), 127.19 (*C*-2, *C*-6 or *C*-11), 127.17 (*C*-2, *C*-6 or *C*-11), 126.84 (*C*-8), 126.82 (*C*-13c), 126.58 (*C*-16), 126.28 (*C*-6a^1), 126.21 (*C*-13d^1), 126.07 (*C*-4), 125.73 (*C*-7), 125.57 (*C*-3), 125.49 (*C*-13b), 124.58 (*C*-15), 124.49 (*C*-12), 124.13 (*C*-2a^1), 123.90 (*C*-4a^1). MALDI-HR-MS for $C_{30}H_{16}^{\cdot+}$ ($M^{\cdot+}$): *m*/*z* = 376.12465 (calculated), 376.12429 (found).

Phenanthro[3,4,5,6-*defgh*][6]helicene O8H

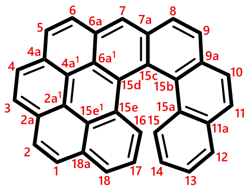

Starting from 21.4 mg of **8H** (0.05 mmol), 10.5 mg of **O8H** was obtained as orange solid (49%). Single crystals suitable for X-ray diffraction were prepared by slow diffusion of pentane into a solution of **O8H** in CH_2Cl_2. ^1H NMR (500 MHz, $CDCl_3$, 298 K): δ 8.67 (s, 1H, *H*-7), 8.46 (d, *J* = 8.2 Hz, 1H, *H*-4), 8.45 (d, *J* = 8.2 Hz, 1H, *H*-3), 8.25 (d, *J* = 8.8 Hz, 1H, *H*-6), 8.21 (d, *J* = 8.3 Hz, 1H, *H*-8), 8.19 (d, *J* = 8.7 Hz, 1H, *H*-2), 8.18 (d, *J* = 8.8 Hz, 1H, *H*-5), 8.14 (dd, *J* = 7.9 Hz, *J* = 0.8 Hz, 1H, *H*-16), 8.07 (d, *J* = 8.7 Hz, 1H, *H*-1), 8.03 (d, *J* = 8.3 Hz, 1H, *H*-9), 8.00 (d, *J* = 8.5 Hz, 1H, *H*-10), 7.98 (d, *J* = 8.5 Hz, 1H, *H*-11), 7.90 (d, *J* = 8.5 Hz, 1H, *H*-15), 7.85 (d, *J* = 7.8 Hz, 1H, *H*-18), 7.83 (dd, *J* = 7.9 Hz, *J* = 1.4 Hz, 1H, *H*-12), 7.17 (ddd, *J* = 7.9 Hz, *J* = 6.9 Hz, *J* = 1.1 Hz, 1H, *H*-13), 7.06 (dd, *J* = 7.9 Hz, *J* = 7.8 Hz, 1H, *H*-17), 6.50 (ddd, *J* = 8.5 Hz, *J* = 6.9 Hz, *J* = 1.4 Hz, 1H, *H*-14). $^{13}C\{^1H\}$ NMR (126 MHz, $CDCl_3$, 298 K): δ 132.35 (*C*-7a), 132.10 (*C*-11a), 131.49 (*C*-18a), 131.38 (*C*-9a), 130.43 (*C*-6a), 129.73 (*C*-2a), 129.56 (*C*-15e), 128.90 (*C*-15a), 128.66 (*C*-11), 128.01 (*C*-15d), 127.98 (*C*-15), 127.79 (*C*-5), 127.69 (*C*-1), 127.62 (*C*-12), 127.58 (*C*-9), 127.50 (*C*-8), 127.47 (*C*-15b), 127.36 (*C*-18), 127.33 (*C*-6), 126.86 (*C*-2), 126.49 (*C*-10), 126.10 (*C*-4), 125.81 (*C*-18), 125.70 (*C*-3), 125.50 (*C*-13), 125.38 (*C*-15e^1), 125.29 (*C*-6a^1), 125.16 (*C*-7 and *C*-14), 124.75 (*C*-17), 124.38 (*C*-2a^1), 123.86 (*C*-4a^1), 121.82 (*C*-15c). MALDI-HR-MS for $C_{34}H_{18}^{\cdot+}$ ($M^{\cdot+}$): *m*/*z* = 426.14030 (calculated), 426.14068 (found).

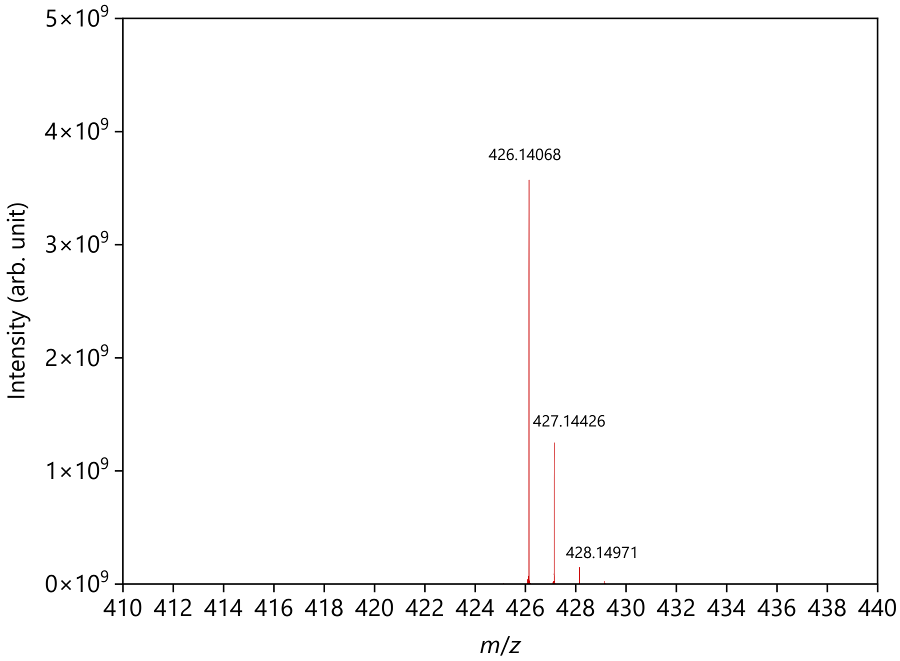

Supplementary Figure 1. MALDI-HR-MS spectrum of **O8H**.

***P*- and *M*-O8H**

Starting from enantiomerically pure *P*- and *M*-**8H**, enantiomerically pure *P*-and *M*-**O8H** were obtained as orange solid (45% and 48%, respectively) with *e.e.* values higher than 99% according to chiral HPLC.

Phenanthro[3,4,5,6-*defgh*][7]helicene O9H

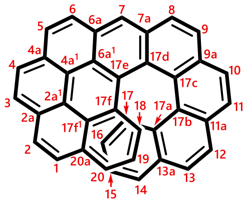

Starting from 23.9 mg of **9H** (0.05 mmol), 12.2 mg of **O9H** was obtained as orange solid (51%). Single crystals suitable for X-ray diffraction were prepared by slow diffusion of pentane into a solution of **O9H** in CH_2Cl_2. ^{1}H NMR (500 MHz, $CDCl_3$, 298 K): δ 8.62 (s, 1H, *H*-7), 8.34 (d, *J* = 8.1 Hz, 1H, *H*-4), 8.30 (d, *J* = 8.1 Hz, 1H, *H*-3), 8.22 (d, *J* = 8.3 Hz, 1H, *H*-8), 8.21 (d, *J* = 8.8 Hz, 1H, *H*-6), 8.12 (d, *J* = 8.8 Hz, 1H, *H*-5), 8.05 (d, *J* = 8.3 Hz, 1H, *H*-9), 8.02 (d, *J* = 8.1 Hz, 1H, *H*-10), 7.95 (d, *J* = 8.1 Hz, 1H, *H*-11), 7.93 (d, *J* = 8.7 Hz, 1H, *H*-2), 7.82 (4d, *J* = 7.9 Hz, *J* = 0.9 Hz 1H, *H*-18), 7.74 (d, *J* = 8.7 Hz, 1H, *H*-2), 7.72 (d, *J* = 8.5 Hz, 1H, *H*-12), 7.50 (d, *J* = 7.5 Hz, 1H, *H*-20), 7.46 (d, *J* = 8.5 Hz, 1H, *H*-13), 7.25 (d, *J* = 8.5 Hz, 1H, *H*-17), 7.11 (d, *J* = 7.9 Hz, 1H, *H*-14), 6.78 (dd, *J* = 7.9 Hz, *J* = 7.5 Hz, 1H, *H*-19), 6.42 (ddd, *J* = 7.9 Hz, *J* = 6.9 Hz, *J* = 1.1 Hz, 1H, *H*-15), 5.63 (ddd, *J* = 8.5 Hz, *J* = 6.9 Hz, *J* = 1.4 Hz, 1H, *H*-16). ^{13}C{^{1}H} NMR (126 MHz, $CDCl_3$, 298 K): δ 132.03 (*C*-9a), 131.87 (*C*-13a), 131.37 (*C*-7a), 131.27 (*C*-11a), 130.76 (*C*-6a), 130.63 (*C*-20a), 129.66

(C-17a), 129.39 (C-2a), 129.06 (C-4a), 129.04 (C-17f), 128.18 (C-17e), 128.14 (C-17b), 128.10 (C-11), 127.90 (C-5), 127.39 (C-8), 127.23 (C-13), 127.21 (C-1, C-9 or C-10), 127.19 (2C, C-1, C-9 or C-10), 127.13 (C-6), 126.69 (C-14), 126.21 (C-2), 125.64 (C-12), 125.61 (C-4), 125.47 (C-3), 125.18 (C-6a^1), 125.17 (C-17f^1), 125.04 (C-20), 124.98 (C-15), 124.81 (C-7), 124.69 (C-17c), 124.19 (C-18), 124.12 (C-4a^1), 124.00 (C-17), 123.72 (C-2a^1), 123.61 (C-19), 123.59 (C-16), 122.92 (C-17d). MALDI-HR-MS for $C_{38}H_{20}^{\cdot+}$ ($M^{\cdot+}$): m/z = 476.15595 (calculated), 476.15661 (found).

***P*- and *M*-O9H**

Starting from enantiomerically pure *P*- and *M*-**9H**, enantiomerically pure *P*-and *M*-**O9H** were obtained as orange solid (34% and 42%, respectively) with *e.e.* values higher than 99% according to chiral HPLC.

O8H$_{CH3}$

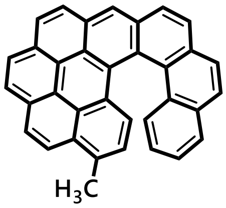

Starting from 22.1 mg of **8H$_{CH3}$** (0.05 mmol), 13.8 mg of **O8H$_{CH3}$** was obtained as orange solid (63%). Single crystals suitable for X-ray diffraction were prepared by slow diffusion of pentane into a solution of **O8H$_{CH3}$** in CH_2Cl_2. ^{1}H NMR (500 MHz, benzene-d_6, 298 K): δ 8.34 (s, 1H), 8.30 (d, J = 8.1 Hz, 1H), 8.23 (d, J = 8.2 Hz, 1H), 8.21 (d, J = 8.2 Hz, 1H), 8.10 (d, J = 8.6 Hz, 1H), 8.02 (d, J = 8.8 Hz, 1H), 8.02 (d, J = 9.0 Hz, 1H), 8.01 (d, J = 9.0 Hz, 1H), 7.97 (d, J = 8.8 Hz, 1H), 7.96 (dd, J = 8.4 Hz, J = 0.5 Hz, 1H), 7.79 (d, J = 8.4 Hz, 1H), 7.78 (s, 2H), 7.64 (ddd, J = 8.0 Hz, J = 1.4 Hz, J = 0.5 Hz, 1H), 6.94 (ddd, J = 8.0 Hz, J = 6.8 Hz, J = 1.1 Hz, 1H), 6.73 (dd, J = 8.1 Hz, J = 0.7 Hz, 1H), 6.25 (ddd, J = 8.6 Hz, J = 6.8 Hz, J = 1.4 Hz, 1H) 2.37 (s, 3H). ^{13}C{^{1}H} NMR (126 MHz, benzene-d_6, 298 K): δ 132.92, 132.82, 132.48, 131.67, 130.84, 130.07, 130.04, 129.82, 129.46, 128.95, 128.75, 128.58, 128.56, 128.46, 128.06, 127.81, 127.79, 127.66, 127.54, 127.53, 126.97, 126.80, 126.71, 126.37, 126.09, 125.89, 125.74, 125.60, 125.49, 125.19, 125.12, 124.78, 124.11, 121.81, 19.53. MALDI-HR-MS for $C_{35}H_{20}^{\cdot+}$ ($M^{\cdot+}$): m/z = 440.15595 (calculated), 440.15087 (found).

O8H$_{CF3}$

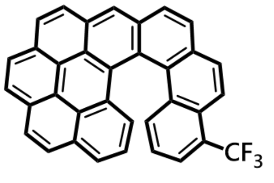

Starting from 24.8 mg of **8H$_{CF3}$** (0.05 mmol), 16.1 mg of **O8H$_{CF3}$** was obtained as orange solid (65%).

Single crystals suitable for X-ray diffraction were prepared by slow diffusion of pentane into a solution of **O8H$_{CF3}$** in CH_2Cl_2. ^{1}H NMR (500 MHz, $CDCl_3$, 298 K): δ 8.03 (dq, J = 8.9 Hz, $J_{H\text{-}F}$ = 1.8 Hz, 1H), 8.31 (s, 1H), 8.17 (s, 2H), 8.09 (d, J = 8.0 Hz, 1H), 8.09 (d, J = 8.4 Hz, 1H), 8.00 (d, J = 8.8 Hz, 1H), 7.95 (d, J = 8.8 Hz, 1H), 7.92 (d, J = 8.4 Hz, 1H), 7.90 (d, J = 8.7 Hz, 1H), 7.77 (d, J = 8.7 Hz, 1H), 7.76 (d, J = 8.9 Hz, 1H), 7.66 (d, J = 8.4 Hz, 1H), 7.52 (d, J = 7.6 Hz, 1H), 7.25 (d, J = 7.3 Hz, 1H), 6.74 (dd, J = 8.0 Hz, J = 7.6 Hz, 1H), 5.90 (dd, J = 8.4 Hz, J = 7.3 Hz, 1H). ^{13}C{^{1}H} NMR (126 MHz, $CDCl_3$, 298 K): δ 132.78, 132.71, 131.87, 131.28, 131.08, 130.22, 130.16, 129.84, 129.38, 128.70, 128.64, 128.56 (2C), 128.31, 127.90, 127.83, 127.50, 127.20 (2C), 126.44, 126.34, 126.14, 125.84, 125.82, 125.60, 125.04, 124.75, 124.38, 124.29(q, $J_{C\text{-}F}$ = 6.1 Hz), 124.05 (q, $J_{C\text{-}F}$ = 2.1 Hz), 123.74, 121.71. ^{19}F{^{1}H} NMR (471 MHz, $CDCl_3$, 298 K): δ −58.1. MALDI-HR-MS for $C_{35}H_{17}F_3^{\cdot+}$ ($M^{\cdot+}$): m/z = 494.12769 (calculated), 494.12881 (found).

O8H$_F$

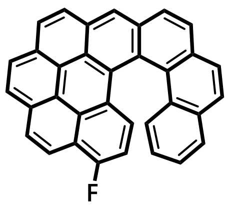

O8H$_F$-α

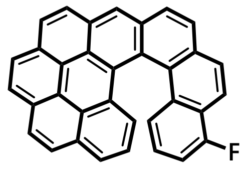

O8H$_F$-β

Starting from 22.3 mg of **8H$_{CF3}$** (0.05 mmol), 13.2 mg of **O8H$_F$** (a mixture of two positional isomers **O8H$_F$-α** and **O8H$_F$-β** at a ratio of 9 : 1 according to the ^{1}H NMR spectrum) was obtained as orange solid (59%). Single crystals suitable for X-ray diffraction were prepared by slow diffusion of pentane into a solution of **O8H$_F$-α** and **O8H$_F$-β** mixture in CH_2Cl_2, showing a mixed-crystal feature with **O8H$_F$-α** and **O8H$_F$-β** with a ratio of 87 : 13. ^{1}H NMR (500 MHz, $CDCl_3$, 298 K): δ 8.67 (s, 0.9H, *H*-β), 8.65 (s, 0.1H, *H*-α), 8.49 – 8.44 (m, 2H), 8.31 – 8.15 (m, 5H), 8.12 (dd, J = 8.0 Hz, J = 1.1 Hz, 0.1H, *H*-β), 8.10 – 7.96 (m, 3.9H), 7.88 – 7.83 (m, 1.9H), 7.67 (dd, J = 8.6 Hz, 0.1H, *H*-β), 7.21 (ddd, J = 8.0 Hz, J = 6.9 Hz, J = 1.1 Hz, 0.9H, *H*-α), 7.13 (dd, J = 8.0 Hz, J = 7.6 Hz, 0.1H, *H*-β), 6.85 (ddd, $J_{H\text{-}F}$ = 10.2 Hz, J = 7.6 Hz, J = 8.6 Hz, J = 0.8 Hz, 0.1H, *H*-β), 6.78 (dd, $J_{H\text{-}F}$ = 9.8 Hz, J = 8.8 Hz, 0.9H, *H*-α), 6.54 (ddd, J = 8.6 Hz, J = 6.9 Hz, J = 1.3 Hz, 0.9H, *H*-α), 6.31 (dd, J = 8.6 Hz, J = 7.6 Hz, $J_{H\text{-}F}$ = 6.0 Hz, 0.1H, *H*-β). ^{19}F{^{1}H} NMR (471 MHz, $CDCl_3$, 298 K): δ −122.6 (0.9F, *F*-α), −123.1 (0.1F, *F*-β). MALDI-HR-MS for $C_{34}H_{17}F^{\cdot+}$ ($M^{\cdot+}$): m/z = 444.13088 (calculated), 444.13080 (found).

O8H$_{Br}$

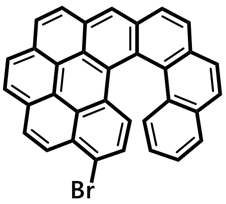

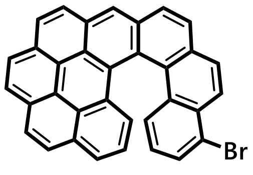

O8H$_{Br}$-α **O8H$_{Br}$-β**

Starting from 25.4 mg of **8H$_{Br}$** (0.05 mmol), 14.9 mg of **O8H$_{Br}$** (a mixture of two positional isomers **O8H$_{Br}$-α** and **O8H$_{Br}$-β** at a ratio of 3 : 1 according to the ^{1}H NMR spectrum) was obtained as orange solid (59%). Single crystals suitable for X-ray diffraction were prepared by slow diffusion of pentane into a solution of **O8H$_{Br}$-α** and **O8H$_{Br}$-β** mixture in CH_2Cl_2, showing a mixed-crystal feature with **O8H$_{Br}$-α** and **O8H$_{Br}$-β** with a ratio of 90 : 10. ^{1}H NMR (500 MHz, $CDCl_3$, 298 K): *δ* 8.70 (s, 0.75H, *H*-α), 8.67 (s, 0.25H, *H*-β), 8.51 – 8.44 (m, 3H), 8.30 – 8.17 (m, 4H), 8.12 – 7.92 (m, 4.25H), 7.87 – 7.83 (m, 1.75H), 7.45 (dd, *J* = 7.4 Hz, *J* = 1.1 Hz, 0.25H, *H*-β), 7.28 (d, *J* = 8.6 Hz, 0.75H, *H*-α), 7.22 (ddd, *J* = 8.0 Hz, *J* = 6.9 Hz, *J* = 1.1 Hz, 0.75H, *H*-α), 7.12 (dd, *J* = 8.0 Hz, *J* = 7.4 Hz, 0.25H, *H*-β), 6.54 (ddd, *J* = 8.6 Hz, *J* = 6.9 Hz, *J* = 1.3 Hz, 0.75H, *H*-α), 6.31 (dd, *J* = 8.6 Hz, *J* = 7.4 Hz, 0.25H, *H*-β). MALDI-HR-MS for $C_{34}H_{17}{}^{79}Br^{\cdot+}$ ($M^{\cdot+}$): *m*/*z* = 504.05081 (calculated), 504.05325 (found).

O8H$_{2py}$

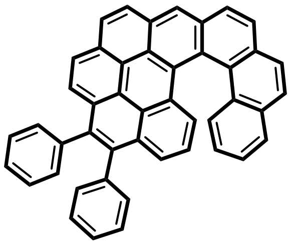

Starting from 29.0 mg of **8H$_{2py}$** (0.05 mmol), 17.2 mg of **O8H$_{2py}$** was obtained as orange solid (60%). Single crystals suitable for X-ray diffraction were prepared by slow diffusion of pentane into a solution of **O8H$_{2py}$** in CH_2Cl_2. ^{1}H NMR (500 MHz, $CDCl_3$, 298 K): *δ* 8.71 (s, 1H), 8.35 (d, *J* = 8.5 Hz, 1H), 8.28 (d, *J* = 8.8 Hz, 1H), 8.24 (d, *J* = 8.4 Hz, 1H), 8.19 (dd, *J* = 7.9 Hz, *J* = 0.9 Hz, 1H), 8.17 (d, *J* = 8.8 Hz, 1H), 8.16 (d, *J* = 8.5 Hz, 1H), 8.05 (d, *J* = 8.4 Hz, 1H), 8.01 (d, *J* = 8.5 Hz, 1H), 7.98 (d, *J* = 8.5 Hz, 1H), 7.98 (d, *J* = 8.5 Hz, 1H), 7.82 (dd, *J* = 8.0 Hz, *J* = 1.3 Hz, 1H), 7.52 (dd, *J* = 7.9 Hz, *J* = 0.9 Hz, 1H), 7.47 (d, *J* = 7.6 Hz, 1H), 7.40 – 4.28 (m, 6H), 7.26 – 7.16 (m, 4H), 6.96 (t, *J* = 7.9 Hz, 1H), 6.60 (ddd, *J* = 8.5 Hz, *J* = 6.9 Hz, *J* = 1.3 Hz, 1H). ^{13}C{^{1}H} NMR (126 MHz, $CDCl_3$, 298 K): *δ* 139.81, 139.79, 138.02, 137.37, 132.39, 132.09, 131.67, 131.57, 131.42, 131.39, 131.36, 131.25, 130.41, 129.59, 129.23, 129.22, 128.88, 128.66, 128.03, 127.99, 127.94, 127.86, 127.84, 127.80, 127.66, 127.63, 127.59, 127.56, 127.51, 127.49, 127.34, 126.80, 126.71, 126.49, 126.05, 125.57, 125.50, 125.27, 125.22 (2C), 125.15, 124.94, 124.68, 123.94, 123.80, 121.84. MALDI-HR-MS for $C_{46}H_{26}{}^{\cdot+}$ ($M^{\cdot+}$): *m*/*z* = 578.20290 (calculated), 578.20585 (found).

O8H$_{mt}$ and OO8H$_{mt}$

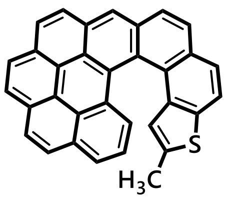

O8H$_{mt}$

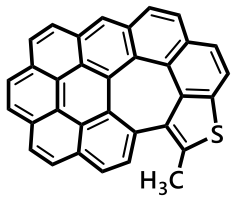

OO8H$_{mt}$

Starting from 22.4 mg of **8H$_{mt}$** (0.05 mmol) and 11.4 mg of DDQ (0.05 mmol, 1.0 equiv.), 4.6 mg of **O8H$_{mt}$** and 2.9 mg of **OO8H$_{mt}$** was obtained as orange solid (21% and 13%, respectively). *Notably, in the beginning of the reaction, the formation of **OO8H$_{mt}$** was soon detected by TLC along with the presence of **O8H$_{mt}$**, indicative of a high tendency of a secondary oxidation.* Single crystals suitable for X-ray diffraction were prepared by slow diffusion of pentane into a solution of **O8H$_{mt}$** or **OO8H$_{mt}$** in CH_2Cl_2, respectively. ^{1}H NMR of **O8H$_{mt}$** (500 MHz, $CDCl_3$, 298 K): δ 8.62 (s, 1H), 8.47 (d, J = 8.2 Hz, 1H), 8.46 (d, J = 8.2 Hz, 1H), 8.42 (dd, J = 7.9 Hz, J = 0.9 Hz, 1H), 8.25 (d, J = 8.7 Hz, 1H), 8.22 (d, J = 8.7 Hz, 1H), 8.19 (d, J = 8.7 Hz, 1H), 8.14 (d, J = 8.7 Hz, 1H), 8.07 (d, J = 8.5 Hz, 1H), 8.02 (d, J = 8.5 Hz, 1H), 8.00 (d, J = 7.7 Hz, 1H), 7.97 (dd, J = 8.4 Hz, J = 0.5 Hz, 1H), 7.88 (d, J = 8.4 Hz, 1H), 7.33 (dd, J = 7.9 Hz, J = 7.7 Hz, 1H), 6.40 (s, 1H), 1.91 (s, 3H). ^{13}C{^{1}H} NMR spectrum of **O8H$_{mt}$** could not be recorded due to the pour solubility. MALDI-HR-MS for **O8H$_{mt}$** $C_{33}H_{18}S^{\cdot+}$ ($M^{\cdot+}$): *m*/*z* = 446.11237 (calculated), 446.11326 (found). ^{1}H NMR of **OO8H$_{mt}$** (500 MHz, $CDCl_3$, 298 K): δ 8.35 (d, J = 8.2 Hz, 1H), 8.34 (s, 1H), 8.31 (d, J = 8.2 Hz, 1H), 8.23 (d, J = 8.1 Hz, 1H), 8.09 (d, J = 8.6 Hz, 1H), 8.06 (d, J = 8.6 Hz, 1H), 8.05 (d, J = 8.6 Hz, 1H), 7.98 (d, J = 8.6 Hz, 1H), 7.82 (d, J = 8.6 Hz, 1H), 7.78 (d, J = 8.6 Hz, 1H), 7.72 (d, J = 8.3 Hz, 1H), 7.68 (d, J = 8.3 Hz, 1H), 7.68 (d, J = 8.1 Hz, 1H), 2.52 (s, 3H). ^{13}C{^{1}H} NMR spectrum of **OO8H$_{mt}$** could not be recorded due to the pour solubility. MALDI-HR-MS for **OO8H$_{mt}$** $C_{33}H_{16}S^{\cdot+}$ ($M^{\cdot+}$): *m*/*z* = 444.09672 (calculated), 444.09770 (found).

OO7H

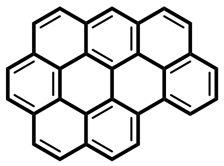

Starting from 18.8 mg of **O7H** (0.05 mmol), 10.6 mg of **OO7H** was obtained as orange solid (57%). Note: CS_2 was used as the eluent for the silica column chromatography. Single crystals suitable for X-ray diffraction were prepared by slow diffusion of pentane into a solution of **OO7H** in CS_2. ^{1}H NMR (500 MHz, CS_2, 298 K): δ 9.72 (d, J = 8.5 Hz, 1H), 9.54 (d, J = 7.9 Hz, 1H), 9.25 (s, 1H), 8.90 (d, J = 8.5 Hz, 1H), 8.85 (d, J = 8.6 Hz, 1H), 8.78 (s, 2H), 8.73 (d, J = 8.6 Hz, 1H), 8.71 (d, J = 8.2 Hz, 1H), 8.70 (d, J = 8.2 Hz, 1H), 8.48 (d, J = 8.7 Hz, 1H), 8.44 (d, J = 7.4 Hz, 1H), 8.34 (dd, J = 7.9

Hz, J = 7.4 Hz, 1H), 8.30 (d, J = 8.7 Hz, 1H). $^{13}C\{^1H\}$ NMR spectrum could not be recorded due to the pour solubility. MALDI-HR-MS for $C_{30}H_{14}^{\cdot+}$ ($M^{\cdot+}$): m/z = 374.10900 (calculated), 374.10946 (found).

One-pot reaction starting directly from **7H**: 18.9 mg of **7H** (0.05 mmol), 27.2 mg of 2,3-dichloro-5,6-dicyano-1,4-benzoquinone (DDQ) (0.12 mmol, 2.4 equiv.) were suspended in 1 mL of dried CH_2Cl_2 under argon at 20 °C, and 0.2 mL of CF_3SO_3H was added to the suspension dropwise with vigorous stirring. The reaction was stirred for 3 hours and then was quenched by 5 mL of saturated $NaHCO_3$ solution. The mixture was extracted by CS_2 and the organic layer was washed by water twice and was dried over Na_2SO_4. The solvent was removed at reduced pressure, and the residue was purified by silica column chromatography (CS_2) to afford **OO7H** as orange solid (8.6 mg, 46%).

OO8H

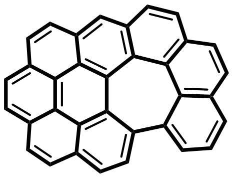

Starting from 21.3 mg of **O8H** (0.05 mmol), 9.7 mg of **OO8H** was obtained as orange solid (46%). Note: the reaction time was prolonged to 3 hours for a higher conversion. Single crystals suitable for X-ray diffraction were prepared by slow diffusion of pentane into a solution of **OO8H** in CH_2Cl_2. ^1H NMR (500 MHz, CS_2, 298 K): δ 8.34 (d, J = 8.1 Hz, 1H), 8.28 (d, J = 8.1 Hz, 1H), 8.24 (s, 1H), 8.24 (d, J = 8.1 Hz, 1H), 8.09 (d, J = 8.6 Hz, 1H), 8.04 (d, J = 8.6 Hz, 1H), 8.02 (d, J = 8.7 Hz, 1H), 7.91 (d, J = 8.7 Hz, 1H), 7.81 (d, J = 8.5 Hz, 1H), 7.75 (d, J = 8.5 Hz, 1H), 7.74 (d, J = 8.5 Hz, 1H), 7.73 (d, J = 8.5 Hz, 1H), 7.72 (dd, J = 7.8 Hz, J = 1.3 Hz, 1H), 7.58 (dd, J = 7.8 Hz, J = 7.2 Hz, 1H), 7.46 (d, J = 8.1 Hz, 1H), 6.97 (dd, J = 7.2 Hz, J = 1.3 Hz, 1H). $^{13}C\{^1H\}$ NMR (126 MHz, $CDCl_3$, 298 K): δ 142.32, 142.22, 139.11, 134.53, 134.29, 132.76, 131.97, 131.86, 131.29, 131.12, 130.93, 130.68, 129.38, 129.34, 128.97, 128.84, 128.29, 128.21, 128.00, 127.47, 127.34, 127.04, 126.89, 126.84, 126.80, 126.76, 125.85, 125.61, 125.51, 125.17, 124.84, 123.20. MALDI-HR-MS for $C_{34}H_{16}^{\cdot+}$ ($M^{\cdot+}$): m/z = 424.12465 (calculated), 424.12441 (found).

One-pot reaction starting directly from **8H**: 21.4 mg of **8H** (0.05 mmol), 27.2 mg of 2,3-dichloro-5,6-dicyano-1,4-benzoquinone (DDQ) (0.12 mmol, 2.4 equiv.) were suspended in 1 mL of dried CH_2Cl_2 under argon at 20 °C, and 0.2 mL of CF_3SO_3H was added to the suspension dropwise with vigorous stirring. The reaction was stirred for 3 hours and then quenched by 5 mL of saturated $NaHCO_3$ solution. The mixture was extracted by CH_2Cl_2 and the organic layer was washed by water twice and was dried over Na_2SO_4. The solvent was removed at reduced pressure, and the residue was purified

by silica column chromatography (heptane : CH_2Cl_2 = 10 : 1, v/v) to afford **OO8H** as orange solid (8.5 mg, 40%).

OO9H

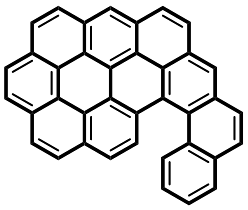

Starting from 23.8 mg of **O9H** (0.05 mmol), 11.4 mg of **OO9H** was obtained as orange solid (48%). Note: CH_3SO_3H was used instead of CF_3SO_3H for the reaction and the reaction time was prolonged to 3 hours for a higher conversion. CS_2 was used as the eluent for the silica column chromatography. Single crystals suitable for X-ray diffraction were prepared by slow diffusion of pentane into a solution of **OO9H** in CS_2. ^{1}H NMR (500 MHz, CS_2, 298 K): δ 9.68 (d, J = 8.6 Hz, 1H), 9.23 (s, 1H), 8.89 (d, J = 8.5 Hz, 1H), 8.79 (s, 2H), 8.78 (d, J = 8.5 Hz, 1H), 8.74 (d, J = 8.3 Hz, 1H), 8.73 (d, J = 8.5 Hz, 1H), 8.68 (d, J = 8.5 Hz, 1H), 8.65 (s, 1H), 8.48 (d, J = 8.6 Hz, 1H), 8.38 (d, J = 8.8 Hz, 1H), 8.28 (d, J = 8.8 Hz, 1H), 8.20 (d, J = 8.4 Hz, 1H), 8.05 (d, J = 8.4 Hz, 1H), 7.98 (d, J = 7.9 Hz, 1H), 7.51 (ddd, J = 7.9 Hz, J = 6.9 Hz, J = 0.9 Hz, 1H), 7.18 (ddd, J = 8.3 Hz, J = 6.9 Hz, J = 1.3 Hz, 1H). $^{13}C\{^1H\}$ NMR spectrum could not be recorded due to the pour solubility. MALDI-HR-MS for $C_{38}H_{18}^{\cdot+}$ ($M^{\cdot+}$): m/z = 474.14030 (calculated), 474.12441 (found).

Oxidative Cyclo-Rearrangement of Nitrogen-Containing Helicenes

General Method

0.05 mmol of helicene substrate was suspended in 20 mL of dried CH_2Cl_2 under argon at 20 °C, and 0.1 mL of CF_3SO_3H was added to the suspension dropwise under stirring. After 5 min, 13.6 mg of DDQ (0.06 mmol, 1.2 equiv.) was added, and the reaction was stirred vigorously for 12 hours. After, 20 mL of NaOH water solution (0.1 mol·L^{-1}) was added dropwise into the reaction, and the mixture was extracted by CH_2Cl_2. The organic layer was washed by water twice and dried over Na_2SO_4. The solvent was removed at reduced pressure, and the residue was purified by silica column chromatography (heptane/ethyl acetate = 1/1, v/v) to afford corresponding nanographene.

O8H$_a$

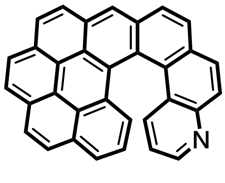

Starting from 21.6 mg of **8H$_a$** (0.05 mmol), 5.6 mg of **O8H$_a$** was obtained as orange solid (26%).

Single crystals suitable for X-ray diffraction were prepared by slow diffusion of pentane into a solution of **O8H$_a$** in CS_2. ^{1}H NMR (500 MHz, benzene-d_6, 298 K): δ 8.47 (dd, J = 4.1 Hz, J = 1.6 Hz, 1H), 8.47 (dd, J = 8.7 Hz, J = 0.6 Hz, 1H), 8.33 (s, 1H), 8.22 (dd, J = 8.0 Hz, J = 1.0 Hz, 1H). 8.18 (d, J = 8.2 Hz, 1H), 8.17(d, J = 8.2 Hz, 1H), 8.02 (ddd, J = 8.6 Hz, J = 1.6 Hz, J = 0.8 Hz, 1H), 8.00 (d, J = 8.8 Hz, 1H), 7.95 (d, J = 8.8 Hz, 1H), 7.93 (d, J = 8.4 Hz, 1H), 7.90 (d, J = 8.8 Hz, 1H), 7.86 (d, J = 8.7 Hz, 1H), 7.78 (d, J = 8.8 Hz, 1H), 7.70 (d, J = 8.4 Hz, 1H), 7.54 (d, J = 7.6 Hz, 1H), 6.78 (dd, J = 8.0 Hz, J = 7.6 Hz, 1H), 5.86 (dd, J = 8.6 Hz, J = 4.1 Hz, 1H). ^{13}C{^{1}H} NMR (126 MHz, benzene-d_6, 298 K): δ 149.37, 148.26, 135.19, 132.60, 131.92, 131.53, 130.99, 130.87, 130.23, 128.24, 130.18, 129.85, 129.38, 128.56, 128.21, 128.11, 127.87, 127.62, 127.58, 127.53, 127.24, 126.43, 126.29, 126.11, 125.95, 125.90, 125.79, 125.30, 124.79, 124.56, 124.41, 121.77, 119.85. MALDI-HR-MS for $C_{33}H_{17}N^{\cdot+}$ ($M^{\cdot+}$): m/z = 427.13555 (calculated), 427.13526 (found).

P- and ***M*-O8H$_a$**

Starting from enantiomerically pure *P*- and *M*-**8H$_a$**, enantiomerically pure *P*-and *M*-**O8H$_a$** was obtained as orange solid (22% and 17%, respectively) with *e.e.* values higher than 99% according to chiral HPLC.

O8H$_{ta}$

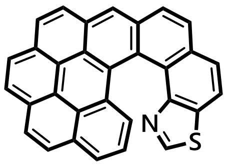

Starting from 21.9 mg of **8H$_{ta}$** (0.05 mmol), 9.9 mg of **O8H$_{ta}$** was obtained as orange solid (46%). Single crystals suitable for X-ray diffraction were prepared by slow diffusion of pentane into a solution of **O8H$_{ta}$** in CH_2Cl_2. ^{1}H NMR (500 MHz, $CDCl_3$, 298 K): δ 8.62 (s, 1H), 8.45 (d, J = 8.1 Hz, 1H), 8.42 (d, J = 8.1 Hz, 1H), 8.35 (dd, J = 7.9 Hz, J = 0.9 Hz, 1H), 8.23 (dd, J = 8.9 Hz, J = 0.3 Hz, 1H), 8.20 (d, J = 8.8 Hz, 1H), 8.19 (dd, J = 8.4 Hz, J = 0.5 Hz, 1H), 8.18 (d, J = 8.9 Hz, 1H), 8.15 (s, 1H), 8.14 (d, J = 8.4 Hz, 1H), 8.12 (d, J = 8.8 Hz, 1H), 8.08 (d, J = 8.4 Hz, 1H), 8.07 (d, J = 8.4 Hz, 1H), 7.94 (d, J = 7.6 Hz, 1H), 7.20 (dd, J = 7.9 Hz, J = 7.6 Hz, 1H). ^{13}C{^{1}H} NMR spectrum could not be recorded due to the pour solubility. MALDI-HR-MS for $C_{31}H_{15}NS^{\cdot+}$ ($M^{\cdot+}$): m/z = 433.09197 (calculated), 433.09228 (found).

1.5 Synthesis of More Complicated Chiral Nanographenes

Synthetic Routes for O6H$_{DPT}$ and CO8H'$_{PDI}$

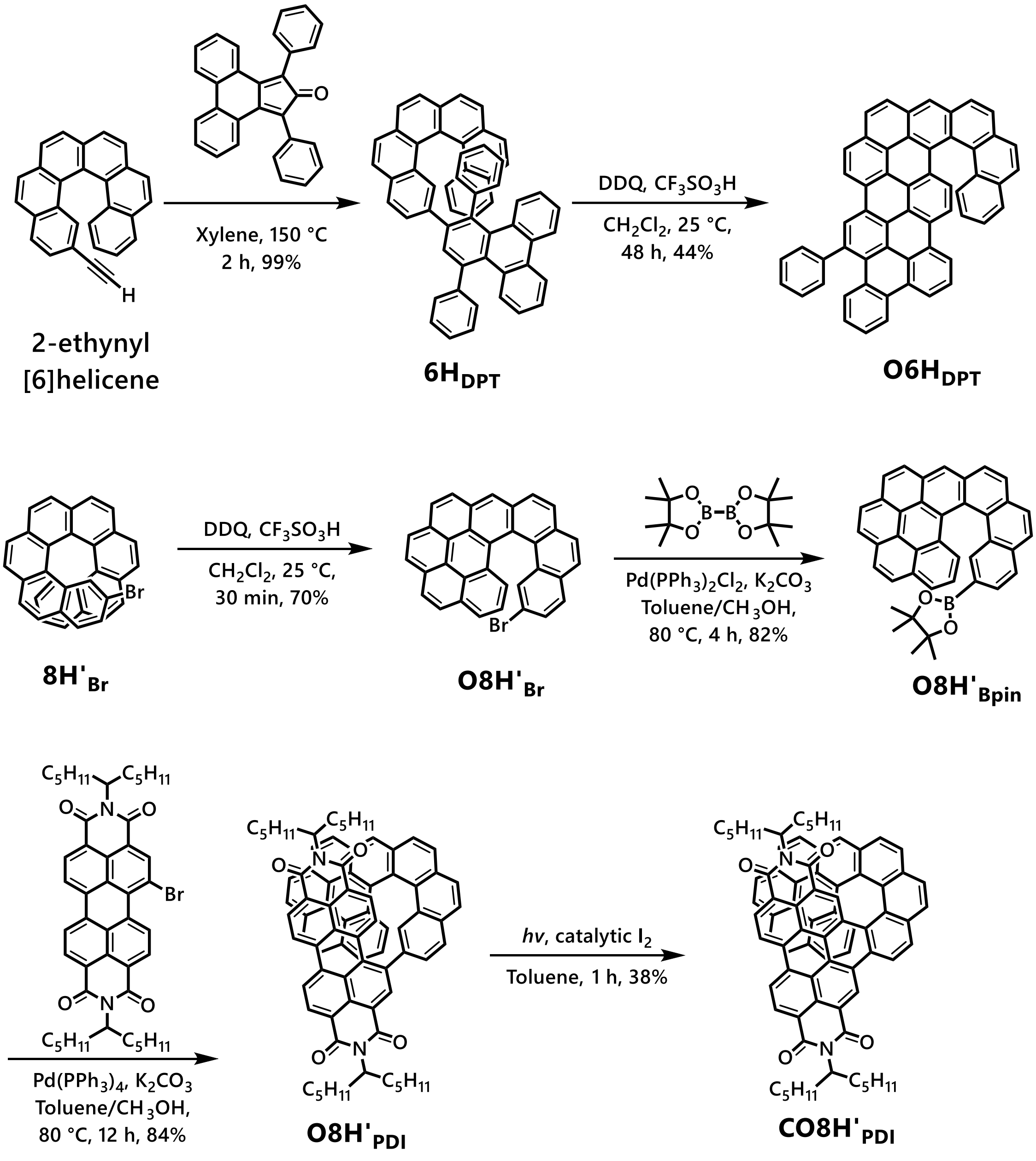

Synthesis of 6H$_{DPT}$

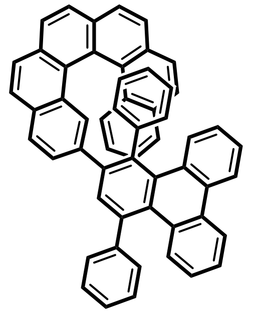

35.2 mg of 2-ethynyl[6]helicene (0.1 mmol) and 38.2 mg of phencyclone (0.1 mmol) were dissolved in 2 mL of xylene under argon. The mixture was then stirred at 140 °C for 3 hours. During the reaction,

the solution color turned from blackish green to colorless with heavy white precipitate. Subsequently, the suspension was cooled to room temperature and the solvent was removed at reduced pressure. The residue was purified by flash silica column chromatography (heptane : CH_2Cl_2 = 1 : 1, v/v) to afford **6H$_{DPT}$** as paled white solid (70.0 mg, 99%). ^{1}H NMR (500 MHz, $CDCl_3$, 298 K): δ ^{1}H NMR (700 MHz, 1,1,2,2-tetrachloroethane-*d*$_2$, 373 K): δ 8.43 (d, *J* = 8.0 Hz, 1H), 8.39 (d, *J* = 7.9 Hz, 1H), 8.03 (s, 2H), 8.00 (d, *J* = 7.9 Hz, 1H), 7.97 (d, *J* = 8.1 Hz, 1H), 7.92 (d, *J* = 7.9 Hz, 1H), 7.90 (d, *J* = 8.2 Hz, 1H), 7.87 (s, 1H), 7.77 – 7.73 (m, 2H), 7.72 (d, *J* = 8.1 Hz, 1H), 7.68 – 7.61 (m, 3H), 7.52 – 7.44 (m, 4H), 7.39 – 7.28 (m, 5H), 7.13 (t, *J* = 7.8 Hz, 1H), 7.03 (t, *J* = 7.3 Hz, 1H), 6.98 – 6.86 (m, 4H), 6.67 (broad, 2H), 6.57 (d, *J* = 8.1 Hz, 1H), 6.42 (s, 1H). ^{13}C{^{1}H} NMR spectrum could not be recorded due to the fast epimerization between different conformers. MALDI-HR-MS for $C_{56}H_{34}^{\cdot+}$ ($M^{\cdot+}$): *m*/*z* = 706.26550 (calculated), 706.26594 (found).

Synthesis of O6H$_{DPT}$

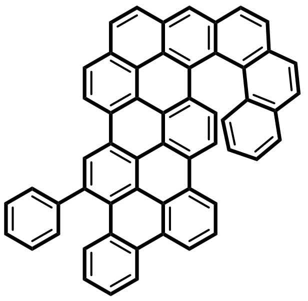

14.1 mg of **6H$_{DPT}$** (0.02 mmol), 15.9 mg of DDQ (0.07 mmol, 3.5 equiv.) were suspended in 20 mL of dried CH_2Cl_2 under argon at 20 °C, and 0.1 mL of CF_3SO_3H was added to the suspension dropwise with vigorous stirring. The reaction was stirred for 48 h and then quenched by 5 mL of saturated $NaHCO_3$ solution. The mixture was extracted by CH_2Cl_2, and the organic layer was washed by water twice and was dried over Na_2SO_4. The solvent was removed at reduced pressure, and the residue was purified by silica column chromatography (heptane/CH_2Cl_2 = 1/1, v/v) to afford **O6H$_{DPT}$** as orange solid (6.1 mg, 44%). Single crystals suitable for X-ray diffraction were prepared by slow diffusion of hexane into a solution of **O6H$_{DPT}$** in CS_2. ^{1}H NMR (500 MHz, $CDCl_3$/CS_2 = 1/1, v/v, 298 K): δ 9.35 (d, *J* = 8.2 Hz, 1H), 9.27 (s, 1H), 8.86 (d, *J* = 8.2 Hz, 1H), 8.73 (d, *J* = 8.1 Hz, 1H), 8.71 (d, *J* = 8.2 Hz, 1H), 8.68 (s, 1H), 8.54 (d, *J* = 8.1 Hz, 1H), 8.35 (d, *J* = 8.7 Hz, 1H), 8.29 – 8.23 (m, 3H), 8.19 (d, *J* = 8.7 Hz, 1H), 8.07 (d, *J* = 8.3 Hz, 1H), 8.05 – 7.97 (m, 4H), 7.79 (d, *J* = 8.5 Hz, 2H), 7.72 (broad, 2H), 7.60 – 7.48 (m, 4H), 7.16 (t, *J* = 7.6Hz, 1H), 6.97 (t, *J* = 7.3Hz, 1H), 6.25 (t, *J* = 7.7Hz, 1H). ^{13}C{^{1}H} NMR spectrum could not be recorded due to the pour solubility. MALDI-HR-MS for $C_{56}H_{28}^{\cdot+}$ ($M^{\cdot+}$): *m*/*z* = 700.21855 (calculated), 700.21736 (found).

Synthesis of 2-bromo[8]helicene 8H'$_{Br}$

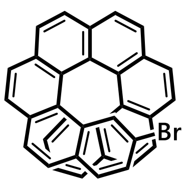

1.03 g of (4-bromobenzyl)triphenylphosphonium bromide 356 mg (2.0 mmol) was suspended in 20 mL of dried THF under argon and cooled to −78 °C. 1.4 mL of *n*-butyllithium (1.6 mol·L^{-1} in hexane, 2.2 mmol, 1.1 eq.) was added, and the reaction mixture was firstly stirred at −78 °C for 5 min and then at room temperature for 30 min. The reaction mixture turned red during the procedure. Then the reaction was cooled to −78 °C again and 0.71 g of 2-formyl[6]helicene (2.0 mmol) dissolved in 5 mL of dried THF was added dropwise. The reaction was stirred at −78 °C for 5 min and then at room temperature for 2 hours. The solvent was removed in vacuum and the residue was purified by flash silica column chromatography (CH_2Cl_2) to afford crude 2-(4-bromostyryl)[6]helicene (a mixture of *cis* and *trans* isomers) as paled yellow solid (0.82 g, 81%). Without further purification, the crude 2-(4-bromostyryl)[6]helicene was then dissolved in toluene with a concentration of 1 mmol·L^{-1}, and catalytic amount of I_2 was added into the solution. The mixture was irradiated under high-pressure mercury lamp (900 W) for 4 hours. Afterwards, the solvent was removed, and the residue was purified by silica column chromatography (heptane : CH_2Cl_2 = 10 : 1, v/v) to afford **8H'$_{Br}$** as yellow solid (622 mg, 76%). ^{1}H NMR (500 MHz, $CDCl_3$, 298 K): δ 8.06 (d, J = 8.2 Hz, 1H), 8.05 (s, 2H), 7.99 (d, J = 8.2 Hz, 1H), 7.89 (d, J = 8.1 Hz, 1H), 7.82 (d, J = 8.1 Hz, 1H), 7.56 (d, J = 8.1 Hz, 1H), 7.41 (d, J = 8.1 Hz, 1H), 7.29 (d, J = 7.9 Hz, 1H), 7.19 – 7.14 (m, 4H), 7.11 (d, J = 8.5 Hz, 1H), 7.09 (d, J = 8.5 Hz, 1H), 7.07 (dd, J = 8.6 Hz, J = 1.9 Hz, 1H), 7.01 (d, J = 8.6 Hz, 1H), 6.96 (ddd, J = 7.9 Hz, J = 6.9 Hz, J = 1.1 Hz, 1H), 6.39 (ddd, J = 8.5 Hz, J = 6.9 Hz, J = 1.3 Hz, 1H). ^{13}C{^{1}H} NMR (126 MHz, $CDCl_3$, 298 K): δ 132.66, 132.14, 131.67, 131.19, 131.14, 130.24, 129.10, 128.27, 128.00, 127.95, 127.48, 127.17, 127.13, 127.07, 127.05, 127.04, 127.02, 127.00, 126.98, 126.85, 126.84, 126.79, 126.78, 126.15, 126.08, 125.89, 125.48, 125.38, 125.31, 124.61, 123.96, 123.47, 118.43. MALDI-HR-MS for $C_{34}H_{19}{}^{79}Br^{\cdot+}$ ($M^{\cdot+}$): m/z = 506.06646 (calculated), 506.06595 (found).

Synthesis of O8H'$_{Br}$

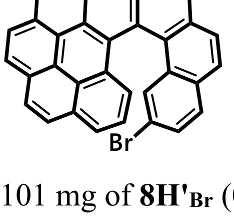

101 mg of **8H'$_{Br}$** (0.2 mmol) , 19.9 mg of DDQ (0.22 mmol, 1.1 equiv.) were suspended in 50 mL of dried CH_2Cl_2 under argon at 20 °C, and 0.5 mL of CF_3SO_3H was added to the suspension dropwise with vigorous stirring. The reaction was stirred for 30 min and then was quenched by 10 mL of

saturated $NaHCO_3$ solution. The mixture was extracted by CH_2Cl_2, and the organic layer was washed by water twice and dried over Na_2SO_4. The solvent was removed at reduced pressure, and the residue was purified by silica column chromatography (heptane/CH_2Cl_2 = 1/10, v/v) to afford **O8H'$_{\mathbf{Br}}$** as orange solid (70.4 mg, 70%). Single crystals suitable for X-ray diffraction were prepared by slow diffusion of pentane into a solution of **O8H'$_{\mathbf{Br}}$** in CH_2Cl_2. ^{1}H NMR (500 MHz, DMSO-d_6, 298 K): δ 8.98 (s, 1H), 8.64 (s, 2H), 8.44 (d, J = 8.1 Hz, 1H), 8.43 (d, J = 8.8 Hz, 1H), 8.38 (d, J = 8.8 Hz, 1H), 8.35 (d, J = 8.8 Hz, 1H), 8.26 (d, J = 8.8 Hz, 1H), 8.24 (d, J = 8.1 Hz, 1H), 8.22 (d, J = 8.5 Hz, 1H), 8.15 (d, J = 8.5 Hz, 1H), 8.10 (d, J = 7.6 Hz, 1H), 7.95 (d, J = 8.0 Hz, 1H), 7.92 (d, J = 8.5 Hz, 1H), 7.86 (d, J = 1.8 Hz, 1H), 7.36 (dd, J = 8.5 Hz, J = 1.8 Hz, 1H), 7.16 (dd, J = 8.0 Hz, J = 7.6 Hz, 1H). ^{13}C{^{1}H} NMR (126 MHz, DMSO-d_6, 298 K): δ 131.87, 131.66, 131.28, 130.17, 129.95, 129.88, 129.30, 129.27, 129.00, 128.98, 128.41, 128.37, 128.24, 128.24, 127.93, 127.59, 127.57, 127.25, 127.14, 127.00, 126.45, 126.43, 126.36, 126.14, 125.86, 125.58, 125.05, 124.80, 124.68, 124.42, 123.08, 122.70, 120.38, 118.81. HR-MS for $C_{34}H_{17}{}^{79}Br^{\cdot+}$ ($M^{\cdot+}$): m/z = 504.05081 (calculated), 504.04954 (found).

Synthesis of O8H'$_{\mathbf{Bpin}}$

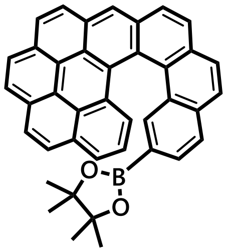

50.4 mg of **O8H'$_{\mathbf{Br}}$** (0.1 mmol), 50.8 mg of bis(pinacolato)diboron (Bpin-Bpin, 0.2 mmol) and 28 mg of K_2CO_3 (0.2 mmol), together with 7.0 mg of $Pd(PPh_3)_2Cl_2$ (10 mol %), were suspended in 5 mL of dried mixed solvent (toluene/CH_3OH = 1/1, v/v) under argon. The reaction was stirred at 80 °C for 4 hours. Subsequently, the solvent was removed, and the residue was purified by silica column chromatography (heptane : ethyl acetate = 5 : 1, v/v) to afford **O8H'$_{\mathbf{Bpin}}$** as orange solid (45.5 mg, 82%). Single crystals suitable for X-ray diffraction were prepared by slow diffusion of pentane into a solution of **O8H'$_{\mathbf{Bpin}}$** in CH_2Cl_2. ^{1}H NMR (500 MHz, $CDCl_3$, 298 K): δ 8.66 (s, 1H), 8.43 (s, 1H), 8.42 (d, J = 8.2 Hz, 1H), 8.41 (d, J = 8.2 Hz, 1H), 8.26 (d, J = 8.7 Hz, 1H), 8.21 (d, J = 8.3 Hz, 1H), 8.18 (d, J = 8.7 Hz, 1H), 8.13 (d, J = 8.7 Hz, 1H), 8.09 (d, J = 7.9 Hz, 1H), 8.03 (d, J = 8.5 Hz, 1H), 8.01 (d, J = 8.2 Hz, 1H), 7.99 (d, J = 8.7 Hz, 1H), 7.96 (d, J = 8.5 Hz, 1H), 7.81 (d, J = 7.9 Hz, 1H), 7.79 (d, J = 7.6 Hz, 1H), 7.52 (d, J = 7.9 Hz, 1H), 7.01 (dd, J = 7.9 Hz, J = 7.6 Hz, 1H), 0.86 (s, 6H), 0.68 (s, 6H). ^{13}C{^{1}H} NMR (126 MHz, $CDCl_3$, 298 K): δ 136.13, 133.75, 132.34, 131.45, 131.22, 130.63, 130.04, 129.74, 129.45, 129.30, 128.36, 128.27, 128.05, 127.83, 127.79, 127.68, 127.62, 127.58, 127.56, 127.35, 127.28, 126.80, 126.66, 125.96, 125.70, 125.62, 125.57, 125.47, 125.10,

124.59, 124.48, 124.22, 121.82, 83.17, 24.35, 24.32. MALDI-HR-MS for $C_{40}H_{29}{}^{11}BO_2{}^{\cdot+}$ ($M^{\cdot+}$): *m*/*z* = 552.22551 (calculated), 552.22479 (found).

Synthesis of O8H'$_{\mathbf{PDI}}$

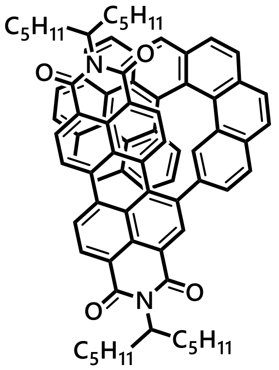

55.2 mg of **O8H'$_{\mathbf{Bpin}}$** (0.1 mmol), 93.3 mg of 1-bromo-perylene-3,4,9,10-tetracarboxylic diimides (1-bromo-PDI, 0.12 mmol, 1.2 equiv.), 11.6 mg of $Pd(PPh_3)_4$ (10 mol %) and 27.6 mg of K_2CO_3 (0.2 mmol) were suspended in 3 mL of mixed solvent (toluene/CH_3OH = 1/1, v/v) under argon. The reaction was stirred at 80 °C for 12 hours. Subsequently, the mixture was extracted by CH_2Cl_2 and the organic layer was collected and dried over Na_2SO_4. The solvent was then removed and the residue was purified by silica column chromatography (heptane : CH_2Cl_2 = 1 : 1, v/v) to afford **O8H'$_{\mathbf{PDI}}$** as dark perple solid (94.1 mg, 84%). **O8H'$_{\mathbf{PDI}}$** presented several distinct rotamers causing from the non-rigid structure, thus providing complicated NMR spectra for assignment. ^{1}H NMR (500 MHz, $CDCl_3$, 298 K): δ 8.73 – 6.90 (m, 24H, from aromatic region), 5.50 – 4.76 (m, 2H, -N-C*H*R_2), 2.72 – 063 (m, 44H, from alkyl chains). MALDI-HR-MS for $C_{80}H_{70}N_2O_4{}^{\cdot+}$ ($M^{\cdot+}$): *m*/*z* = 1122.53301 (calculated), 1122.51677 (found).

Synthesis of CO8H'$_{\mathbf{PDI}}$

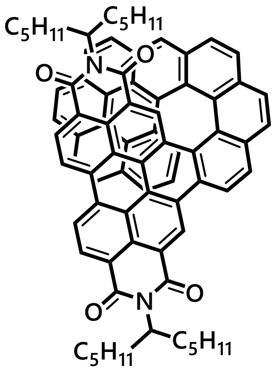

22.5 mg of **O8H'$_{\mathbf{PDI}}$** (0.02 mmol) was dissolved in 20 mL of toluene (1 mmol·L^{-1}), and catalytic amount of I_2 was added into the solution. The mixture was irradiated by a high-pressure mercury lamp (900 W) for 1 hour. Afterwards, the solvent was removed, and the residue was purified by silica column chromatography (heptane : CH_2Cl_2 = 1 : 1, v/v) to afford **CO8H'$_{\mathbf{PDI}}$** as purple solid (8.6 mg,

38%). Single crystals suitable for X-ray diffraction were prepared by slow diffusion of pentane into a solution of **CO8H'PDI** in $CHCl_3$.

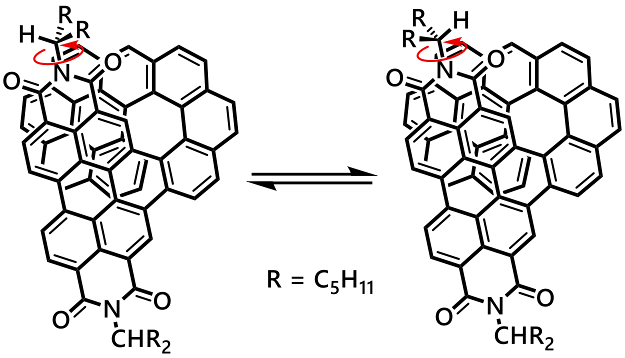

According to the NMR analysis, **CO8H'PDI** presented two distinct rotamers with a ratio of ca. 1:1. This was probably caused by the partially restricted rotation of the alkyl chains. ^{1}H NMR (500 MHz, $CDCl_3$, 298 K): δ 9.61 (broad, 0.5H), 9.58 (broad, 0.5H), 9.02 – 8.92 (m, 1H), 8.74 (d, J = 8.2 Hz, 1H), 8.53 (d, J = 8.6 Hz, 1H), 8.51 – 8.44 (m, 2H), 8.34 (s, 0.5 H), 8.30 (s, 0.5H), 8.28 – 8.18 (m, 6H), 7.69 (d, J = 8.7 Hz, 1H), 7.59 (d, J = 8.1 Hz, 1H), 7.50 (d, J = 8.1 Hz, 1H), 7.44 (d, J = 8.7 Hz, 1H), 7.43 (d, J = 7.9 Hz, 1H), 7.21 (d, J = 7.4 Hz, 1H), 6.71 (dd, J = 7.9 Hz, J = 7.4 Hz, 1H), 6.61 (d, J = 8.8 Hz, 1H), 6.59 (d, J = 8.8 Hz, 1H), 5.45 (broad, 1H), 5.13 (broad, 0.5H), 5.06 (broad, 0.5H), 2.62 (broad, 1H), 2.37 (broad, 2.5H), 2.25 (broad, 0.5H), 2.03 (broad, 3H), 1.89 – 1.62 (m, 8H), 1.52 – 1.16 (m, 13H), 1.11 – 0.81 (m, 13H), 0.60 (broad, 3H). ^{13}C{^{1}H} NMR spectrum could not be recorded due to the fast epimerization between different conformers. MALDI-HR-MS for $C_{80}H_{68}N_2O_4^{\cdot+}$ ($M^{\cdot+}$): m/z = 1120.51736 (calculated), 1120.50106 (found).

1.6 NMR Spectra

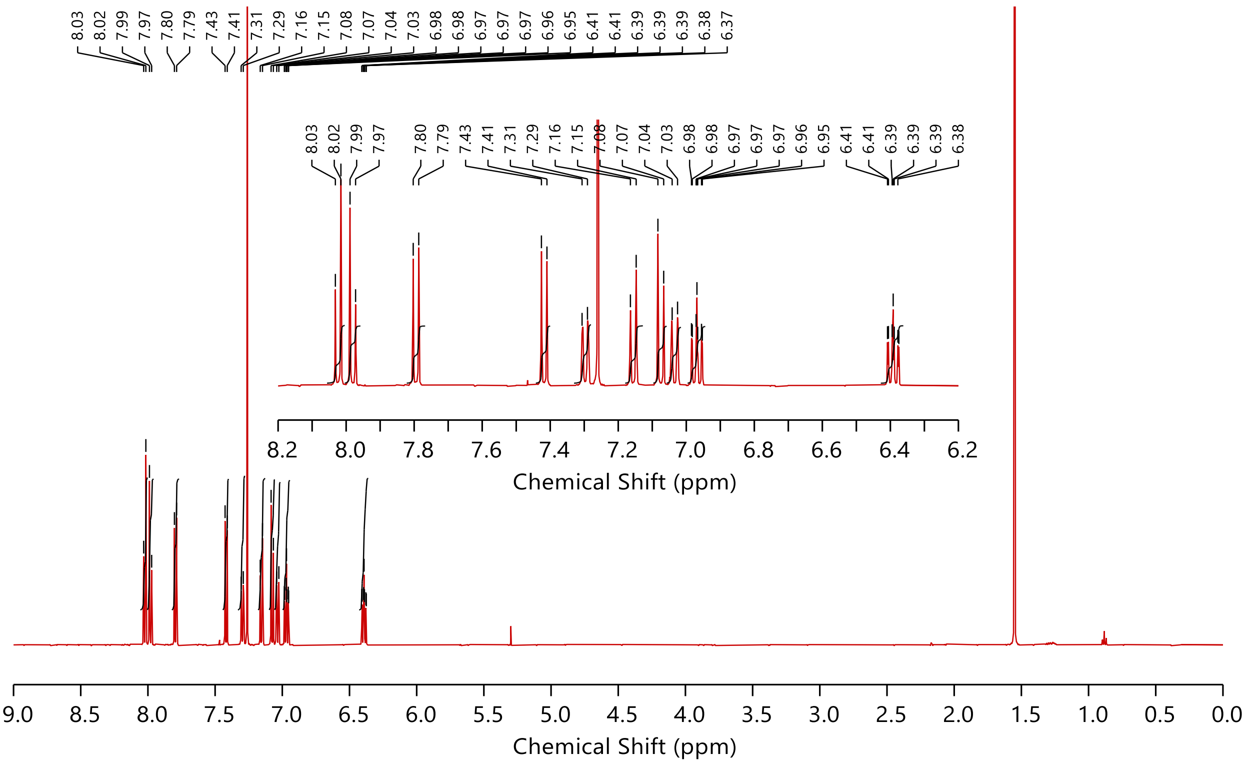

Supplementary Figure 2. ^{1}H NMR spectrum of **8H** (500 MHz, $CDCl_3$, 298 K).

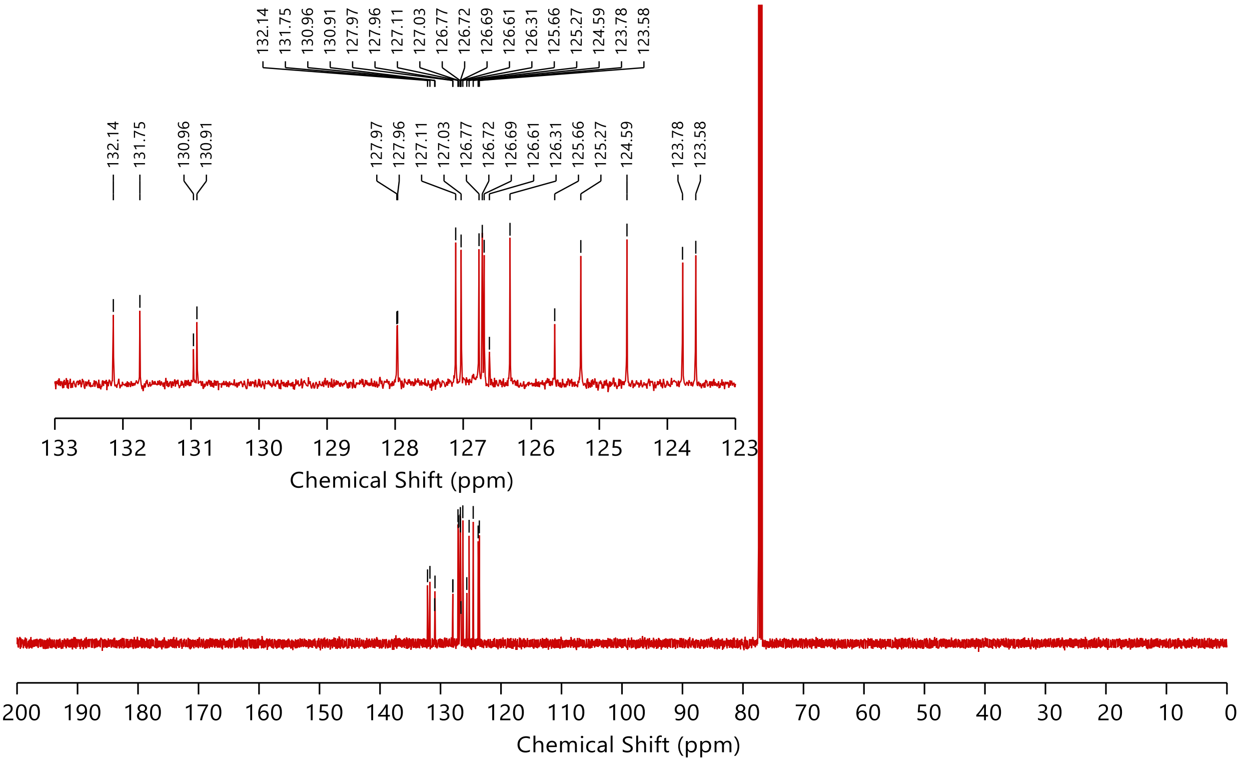

Supplementary Figure 3. ^{13}C{^{1}H} NMR spectrum of **8H** (126 MHz, $CDCl_3$, 298 K).

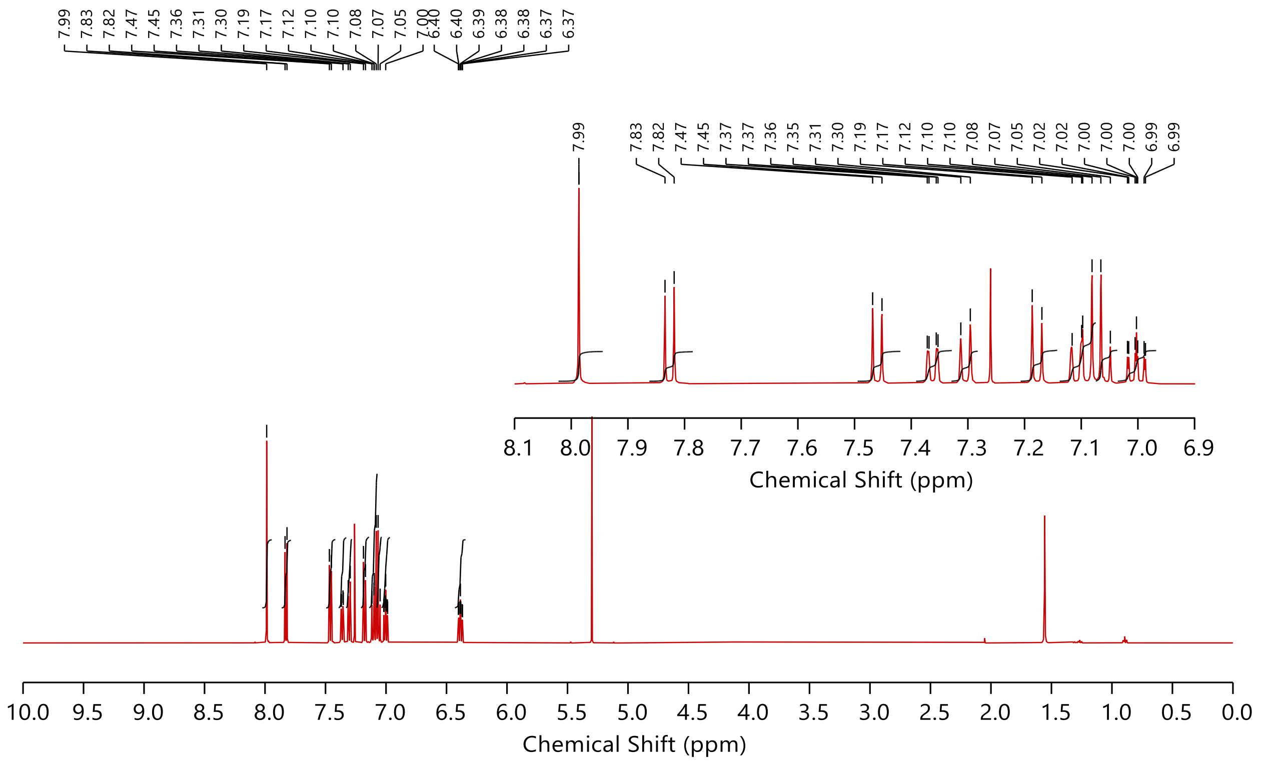

Supplementary Figure 4. ^{1}H NMR spectrum of **9H** (500 MHz, $CDCl_3$, 298 K).

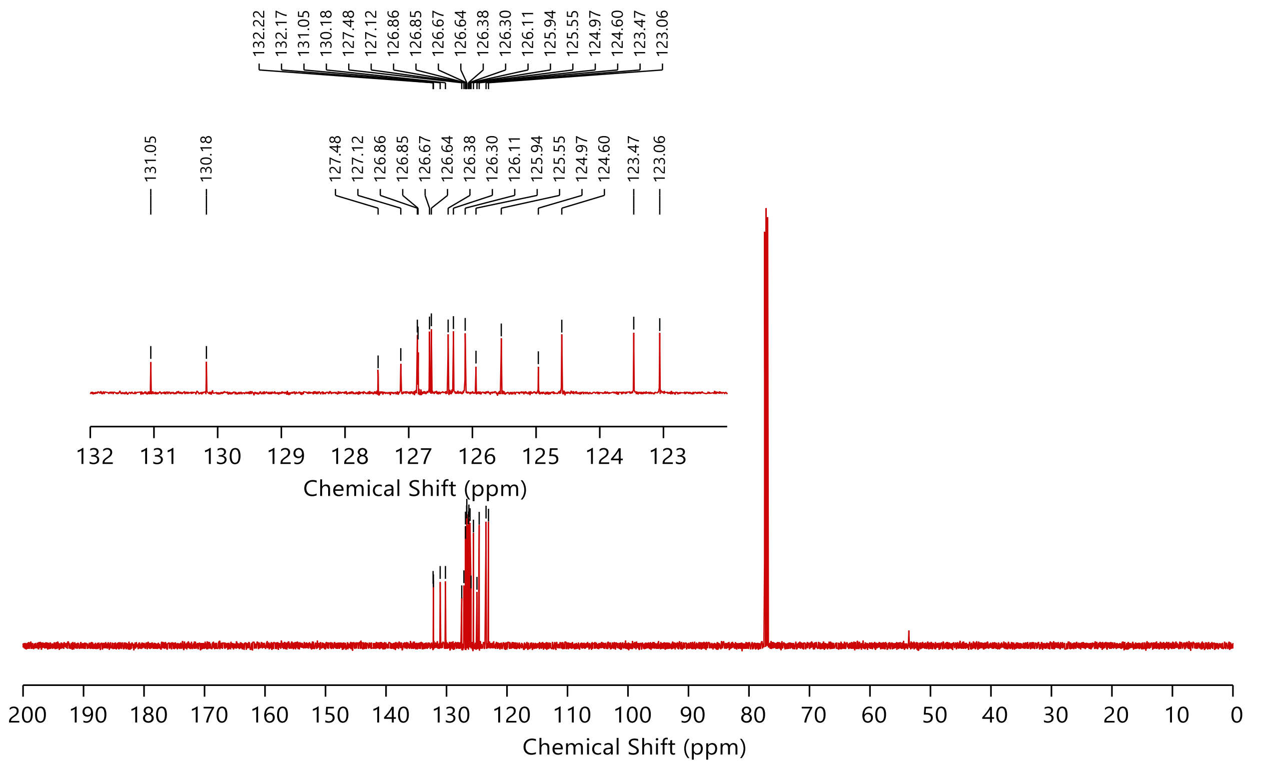

Supplementary Figure 5. $^{13}C\{^{1}H\}$ NMR spectrum of **9H** (126 MHz, $CDCl_3$, 298 K).

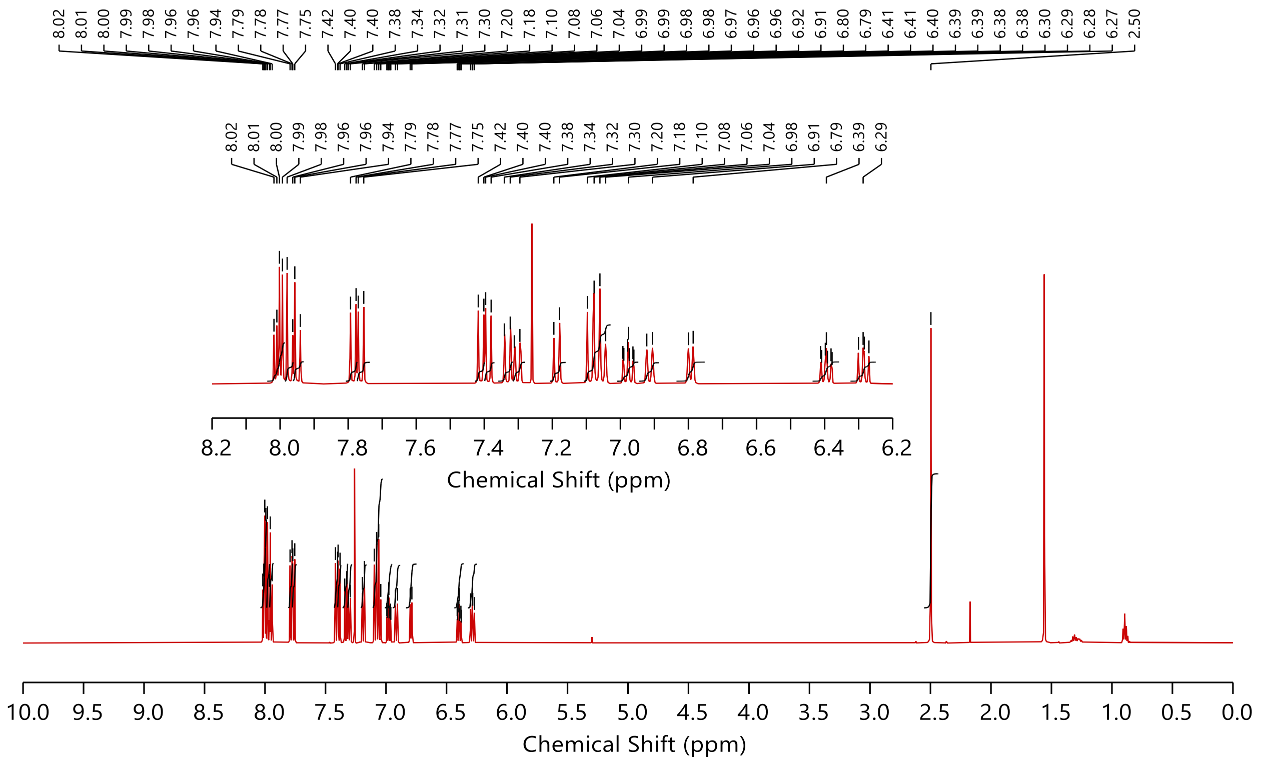

Supplementary Figure 6. ^{1}H NMR spectrum of **8H$_{CH3}$** (500 MHz, $CDCl_3$, 298 K).

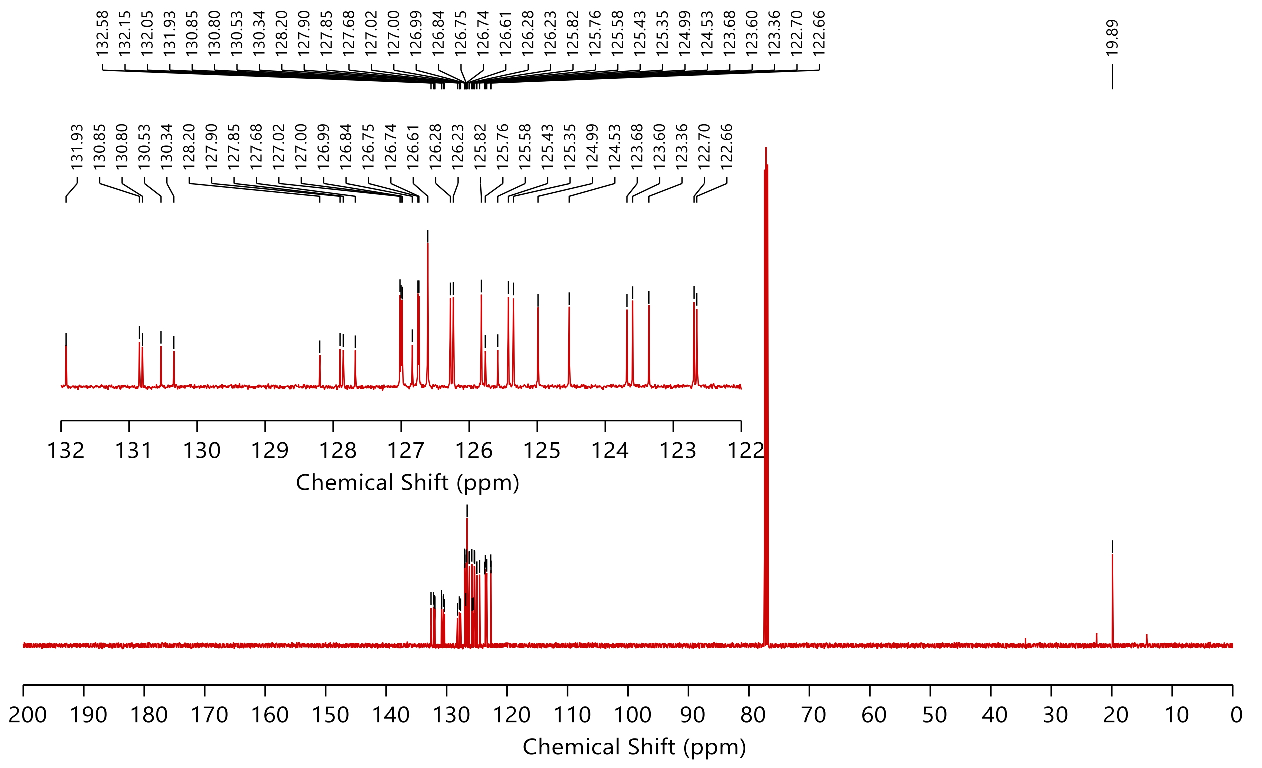

Supplementary Figure 7. $^{13}C\{^{1}H\}$ NMR spectrum of **8H$_{CH3}$** (126 MHz, $CDCl_3$, 298 K).

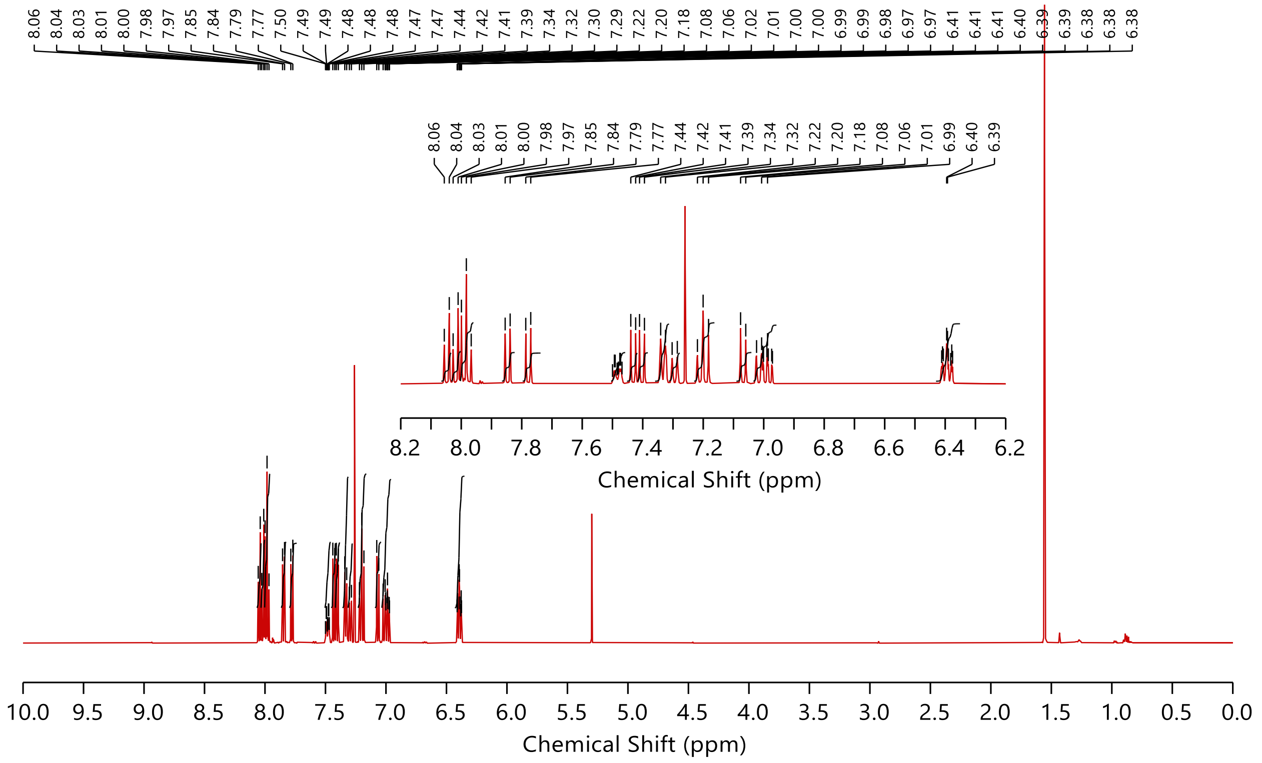

Supplementary Figure 8. ^{1}H NMR spectrum of **8H$_{CF3}$** (500 MHz, $CDCl_3$, 298 K).

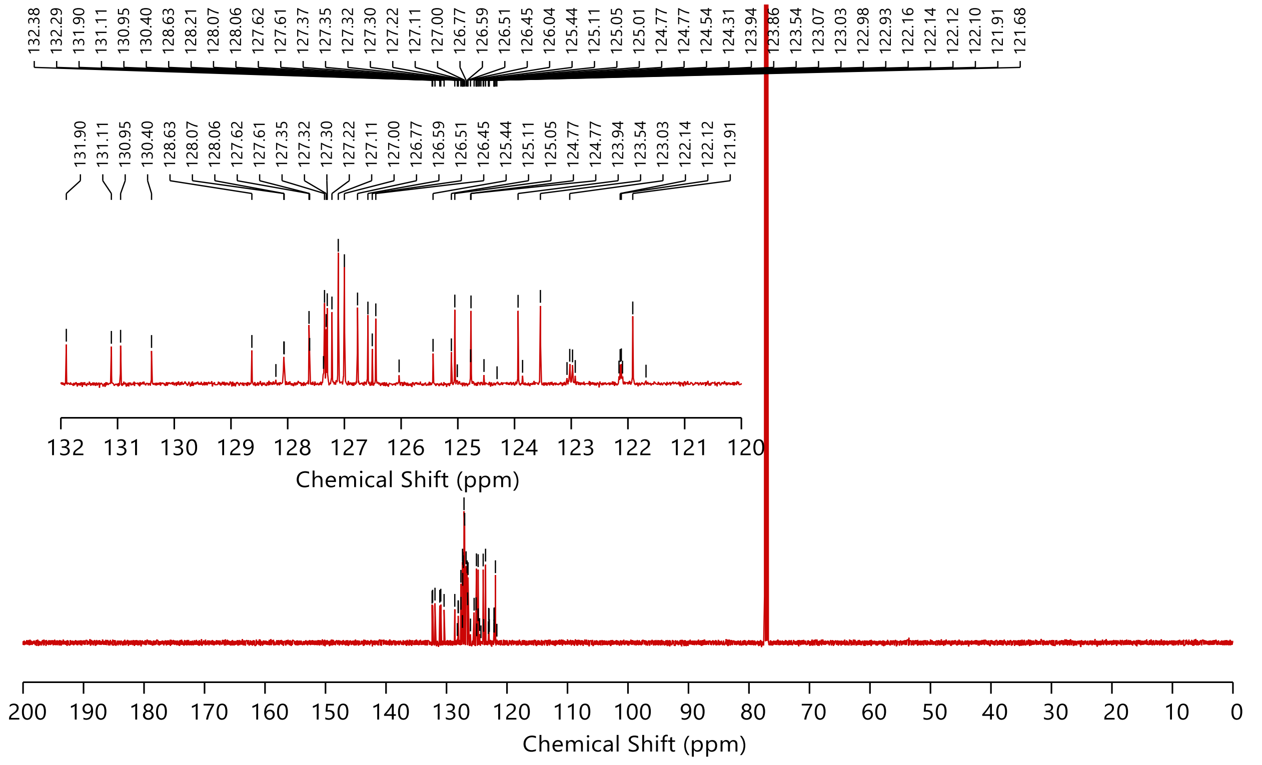

Supplementary Figure 9. $^{13}C\{^1H\}$ NMR spectrum of **8H$_{CF3}$** (126 MHz, $CDCl_3$, 298 K).

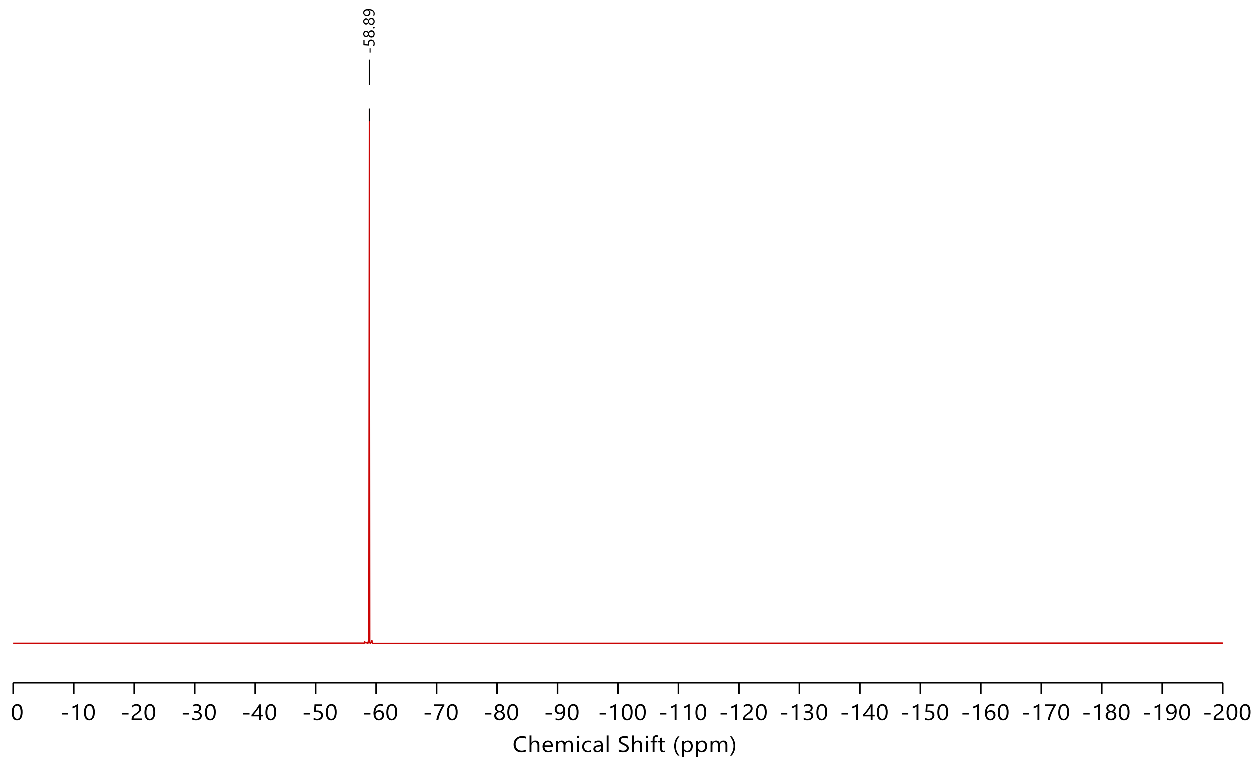

Supplementary Figure 10. $^{19}F\{^{1}H\}$ NMR spectrum of **8H$_{CF3}$** (471 MHz, $CDCl_3$, 298 K).

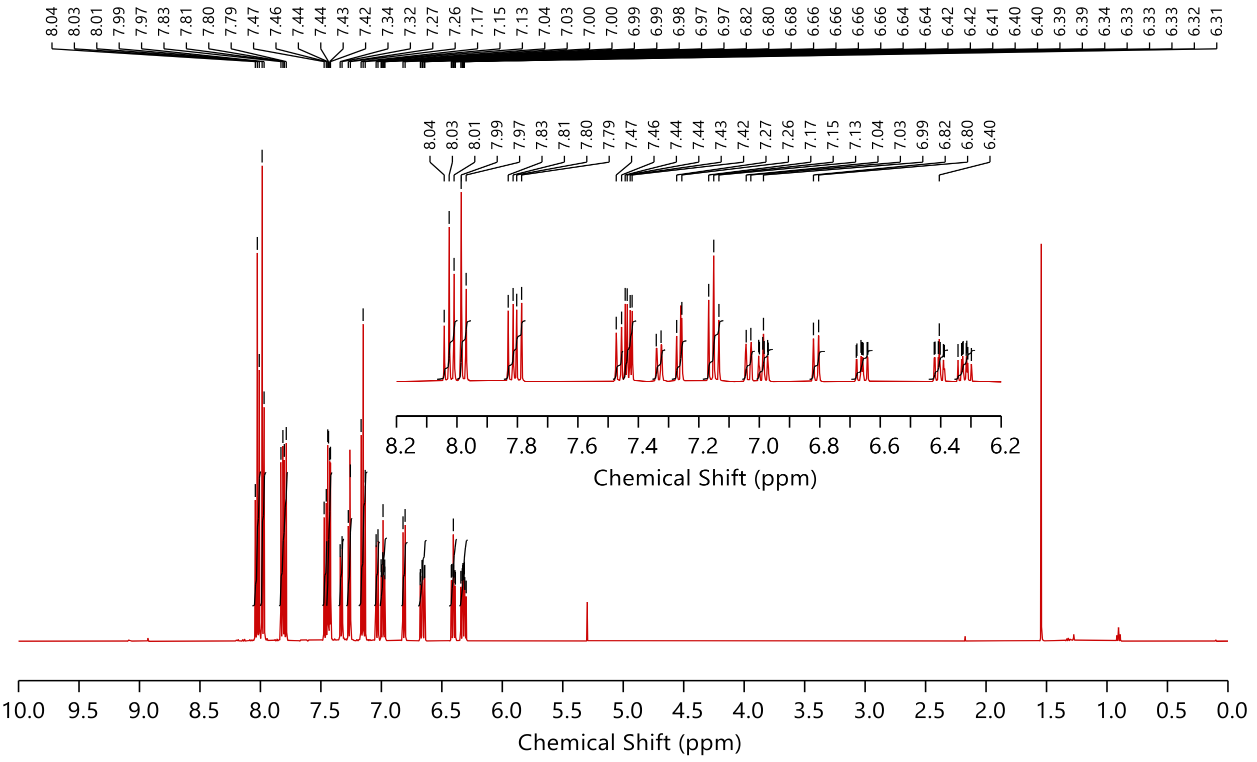

Supplementary Figure 11. ^{1}H NMR spectrum of **8H$_F$** (500 MHz, $CDCl_3$, 298 K).

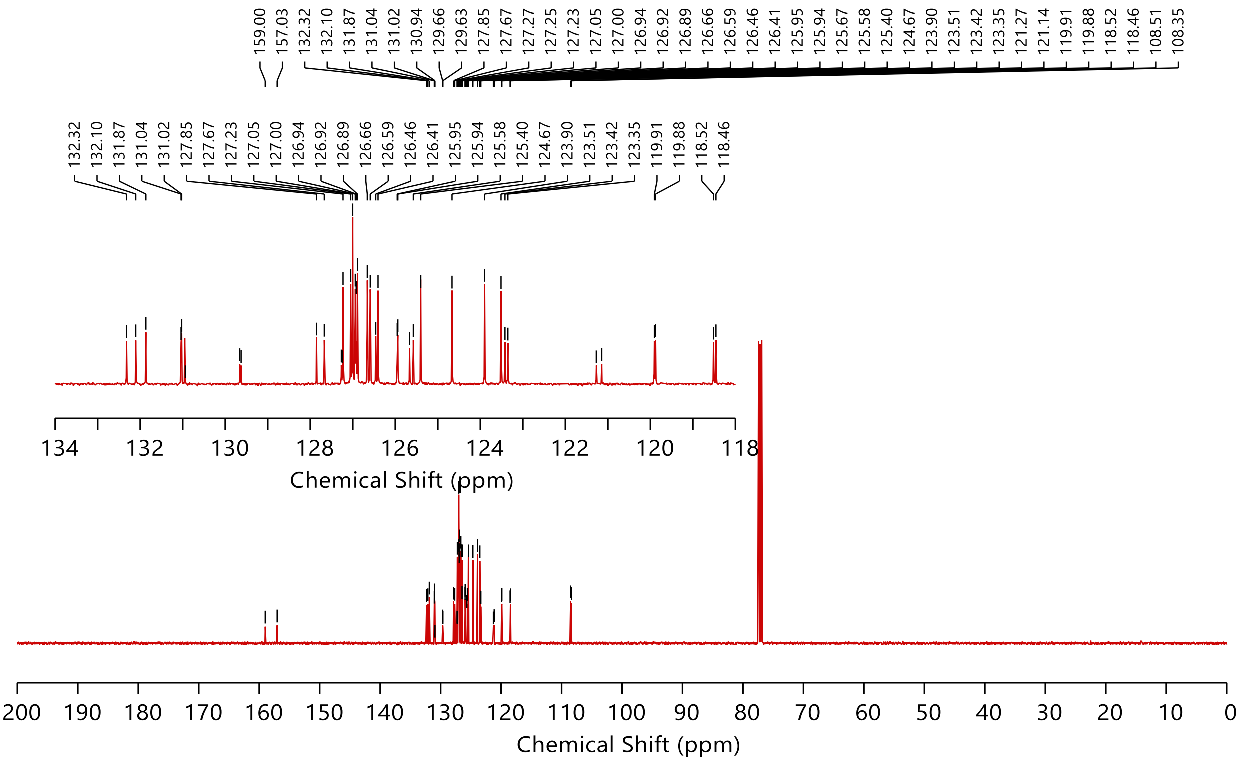

Supplementary Figure 12. $^{13}C\{^{1}H\}$ NMR spectrum of **8H$_F$** (126 MHz, $CDCl_3$, 298 K).

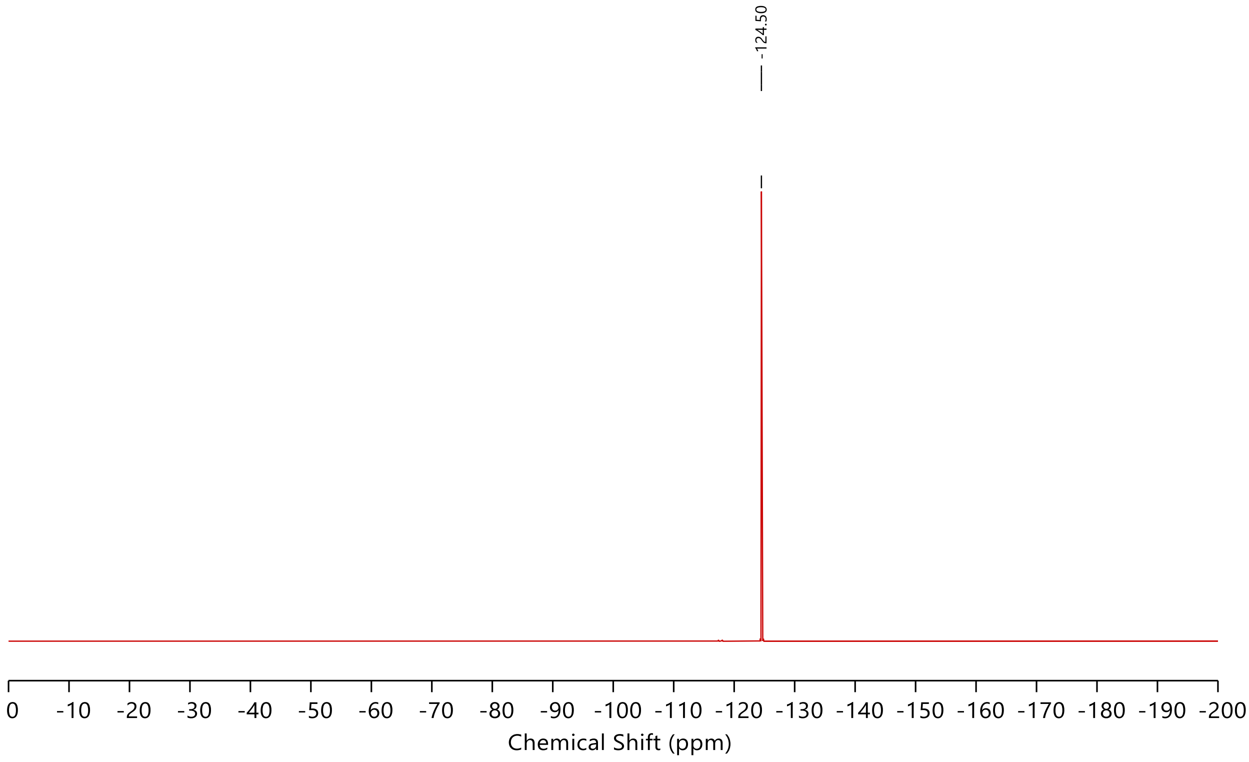

Supplementary Figure 13. $^{19}F\{^{1}H\}$ NMR spectrum of **8H$_F$** (471 MHz, $CDCl_3$, 298 K).

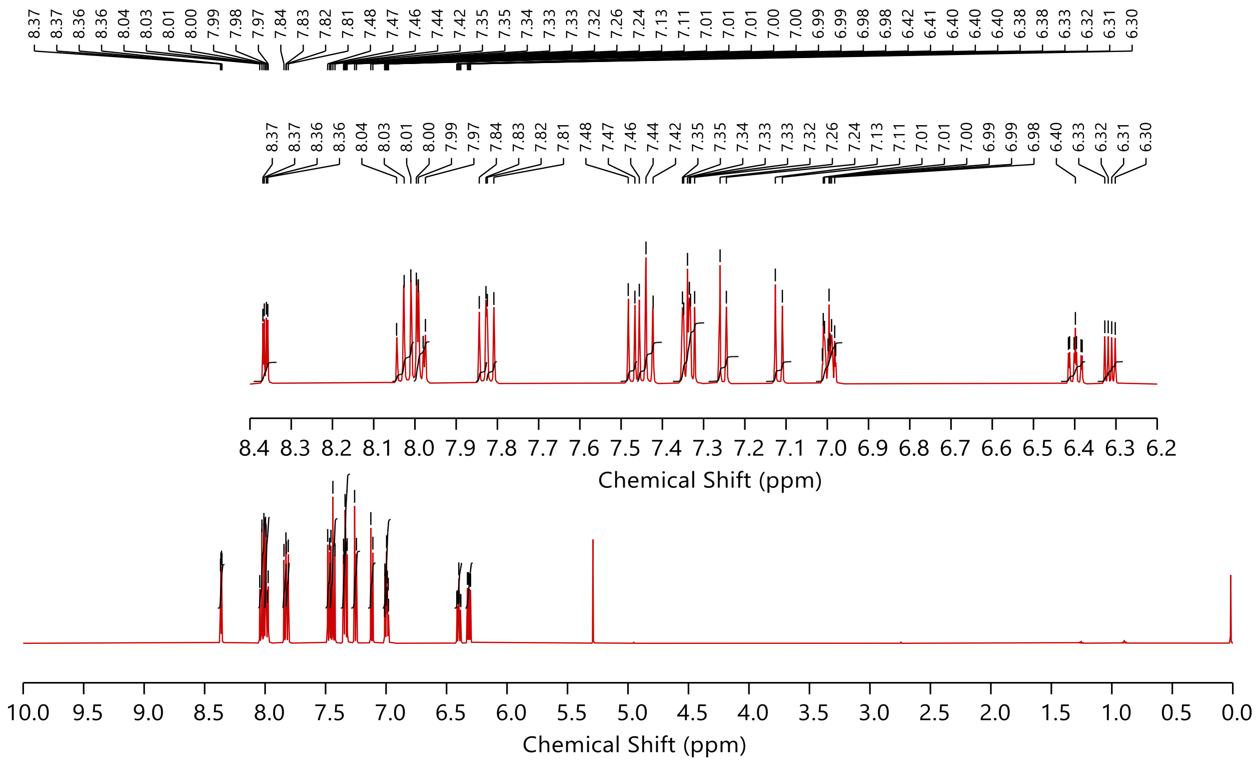

Supplementary Figure 14. ^{1}H NMR spectrum of **8Ha** (500 MHz, $CDCl_3$, 298 K).

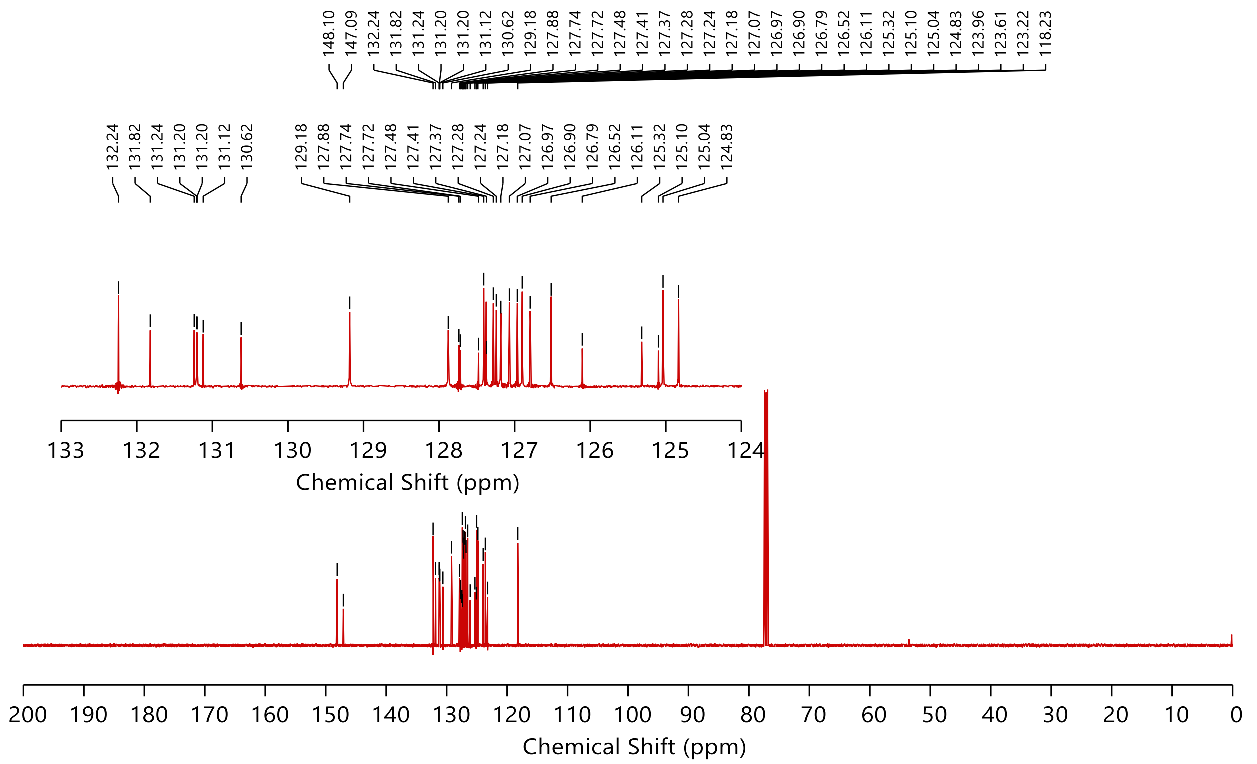

Supplementary Figure 15. $^{13}C\{^{1}H\}$ NMR spectrum of **8Ha** (126 MHz, $CDCl_3$, 298 K).

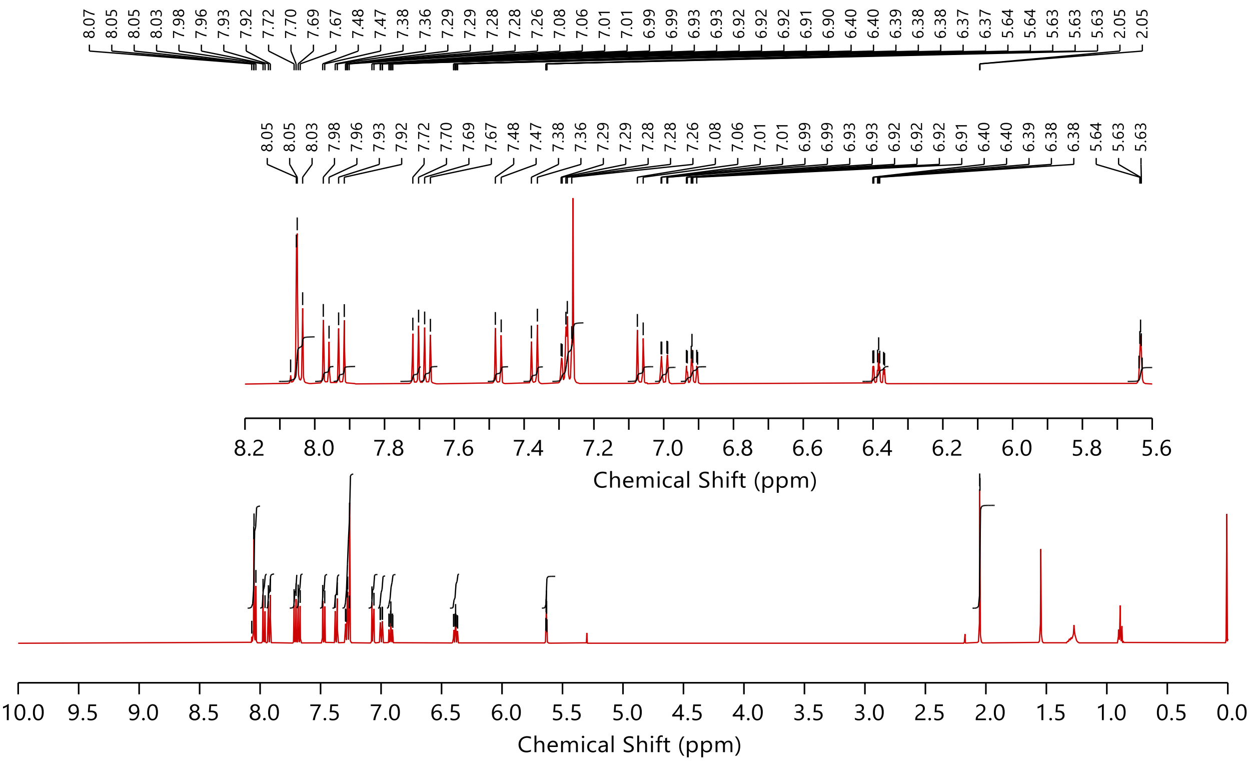

Supplementary Figure 16. ^{1}H NMR spectrum of **8H$_{mt}$** (500 MHz, $CDCl_3$, 298 K).

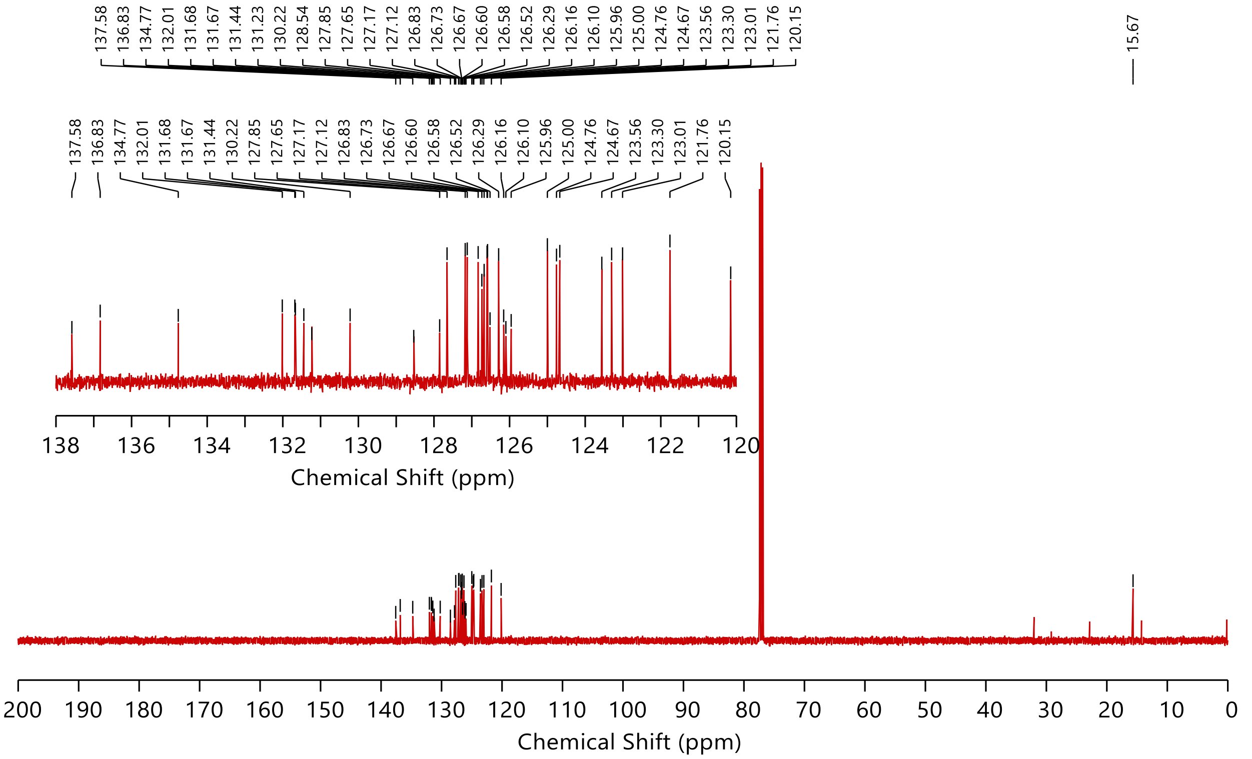

Supplementary Figure 17. ^{13}C{^{1}H} NMR spectrum of **8H$_{mt}$** (126 MHz, $CDCl_3$, 298 K).

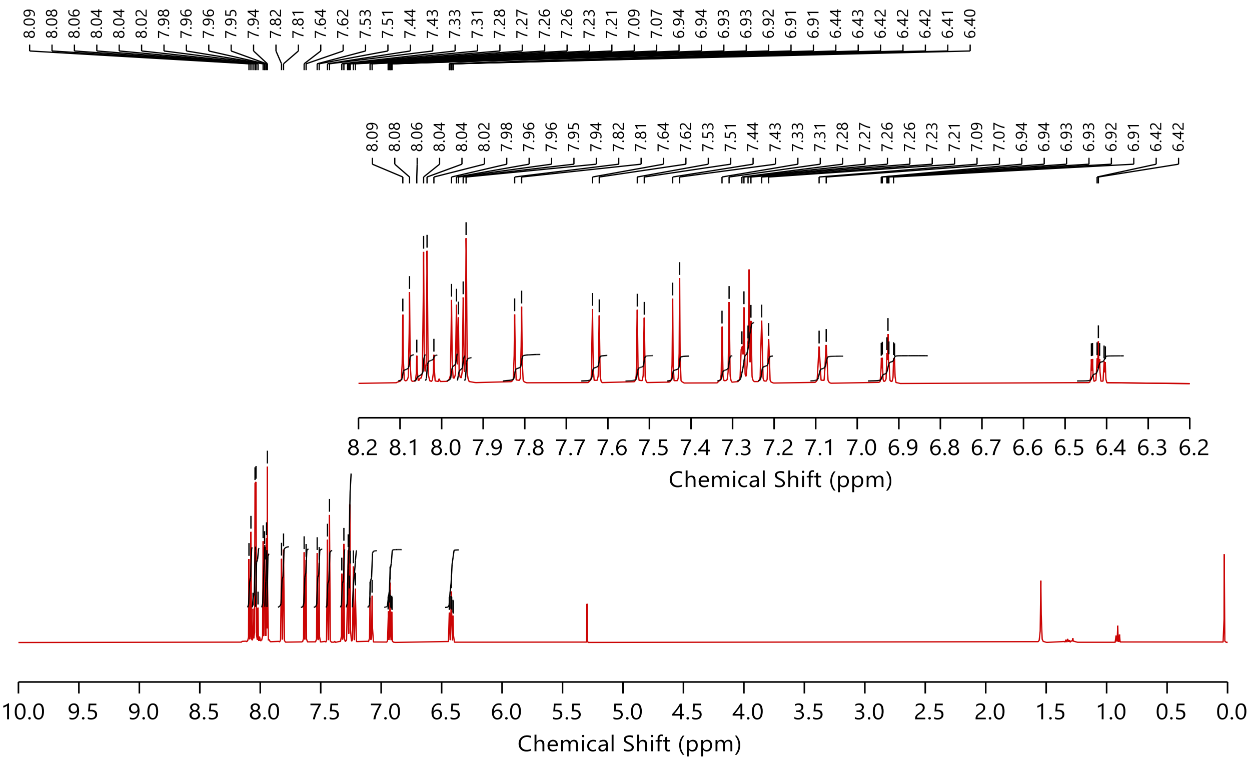

Supplementary Figure 18. ^{1}H NMR spectrum of **8H$_{ta}$** (500 MHz, $CDCl_3$, 298 K).

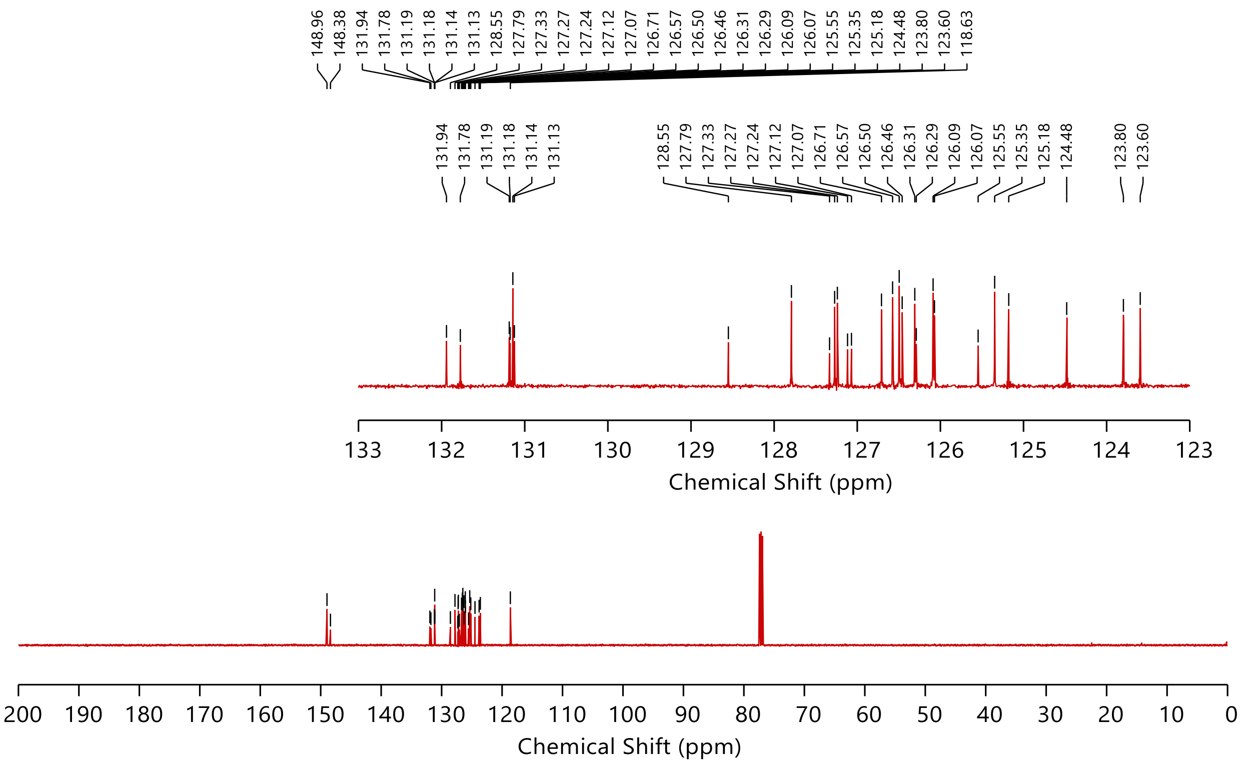

Supplementary Figure 19. ^{13}C{^{1}H} NMR spectrum of **8H$_{ta}$** (126 MHz, $CDCl_3$, 298 K).

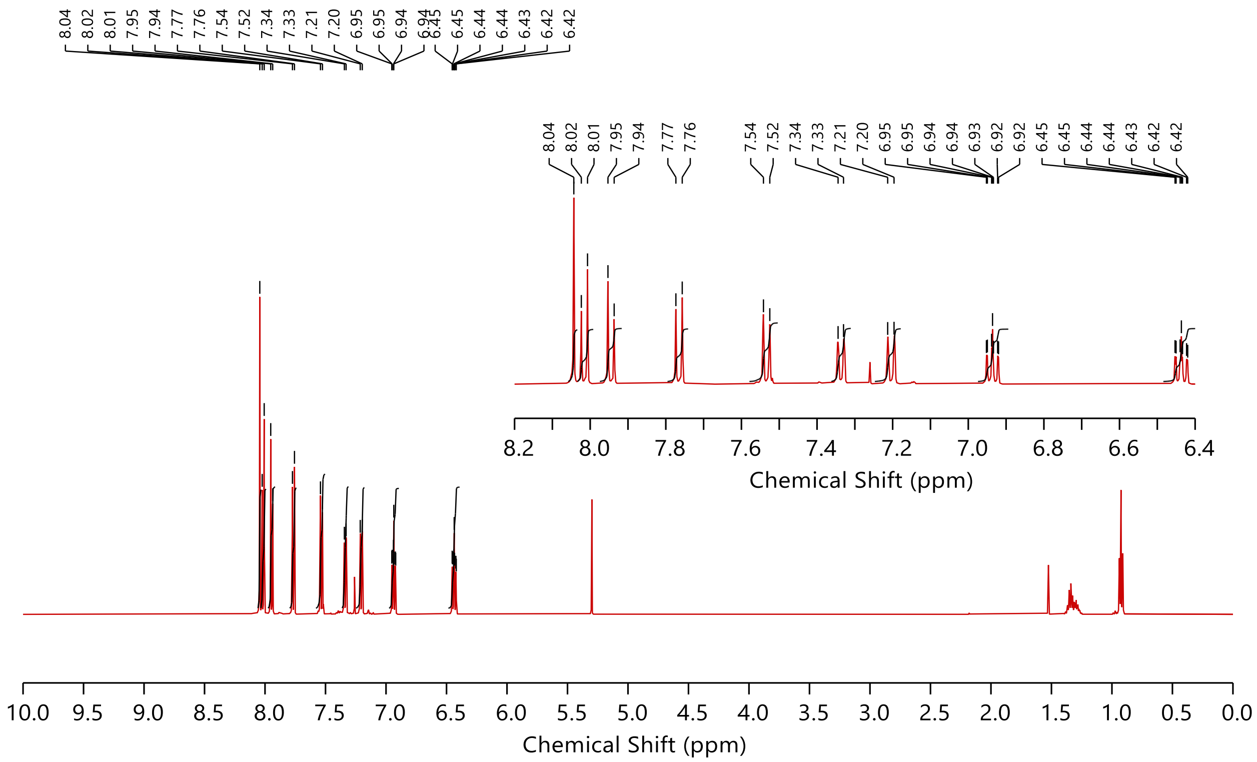

Supplementary Figure 20. ^{1}H NMR spectrum of **7H** (500 MHz, $CDCl_3$, 298 K).

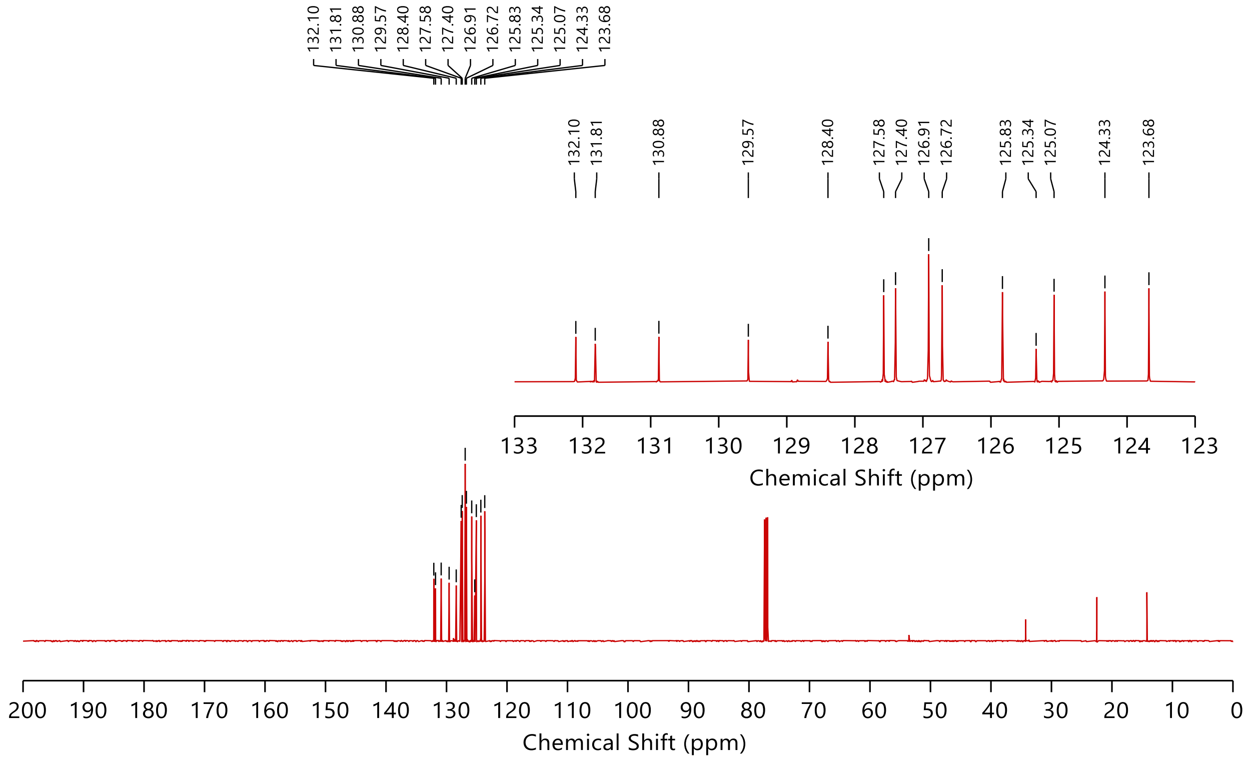

Supplementary Figure 21. $^{13}C\{^{1}H\}$ NMR spectrum of **7H** (126 MHz, $CDCl_3$, 298 K).

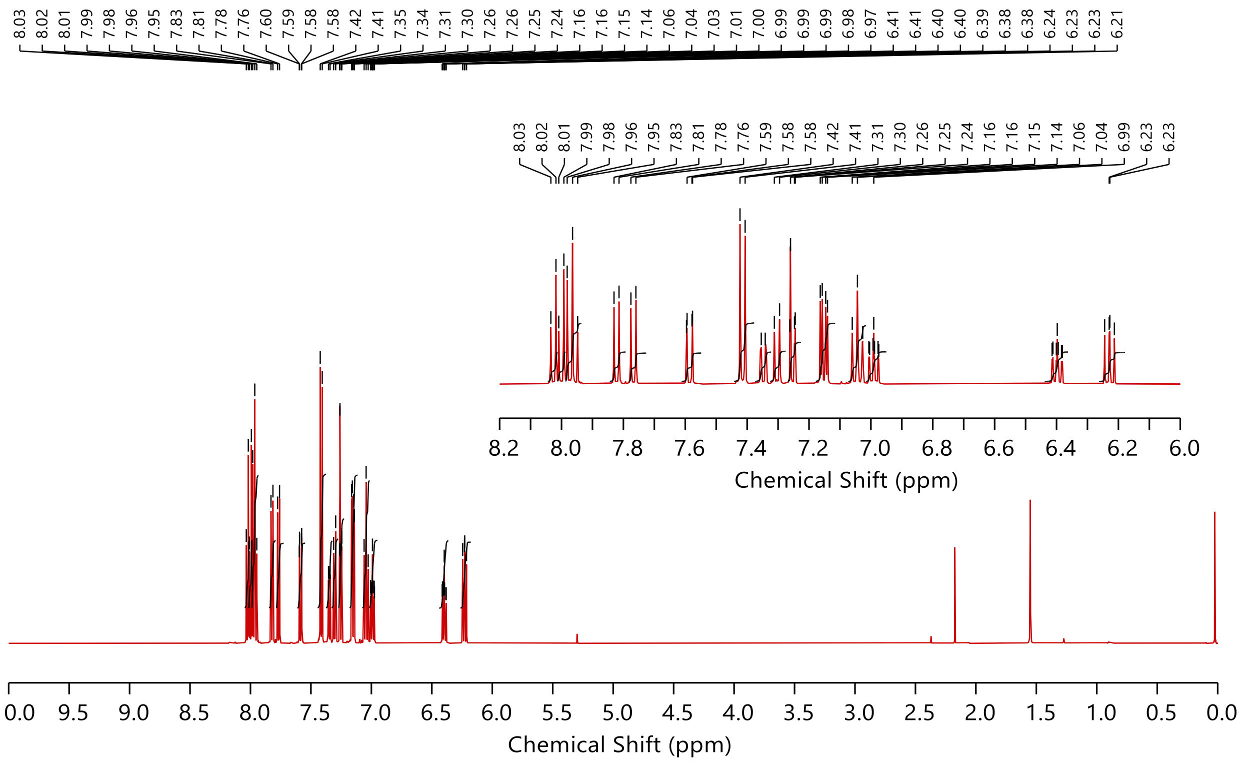

Supplementary Figure 22. ^{1}H NMR spectrum of **8H$_{Br}$** (500 MHz, $CDCl_3$, 298 K).

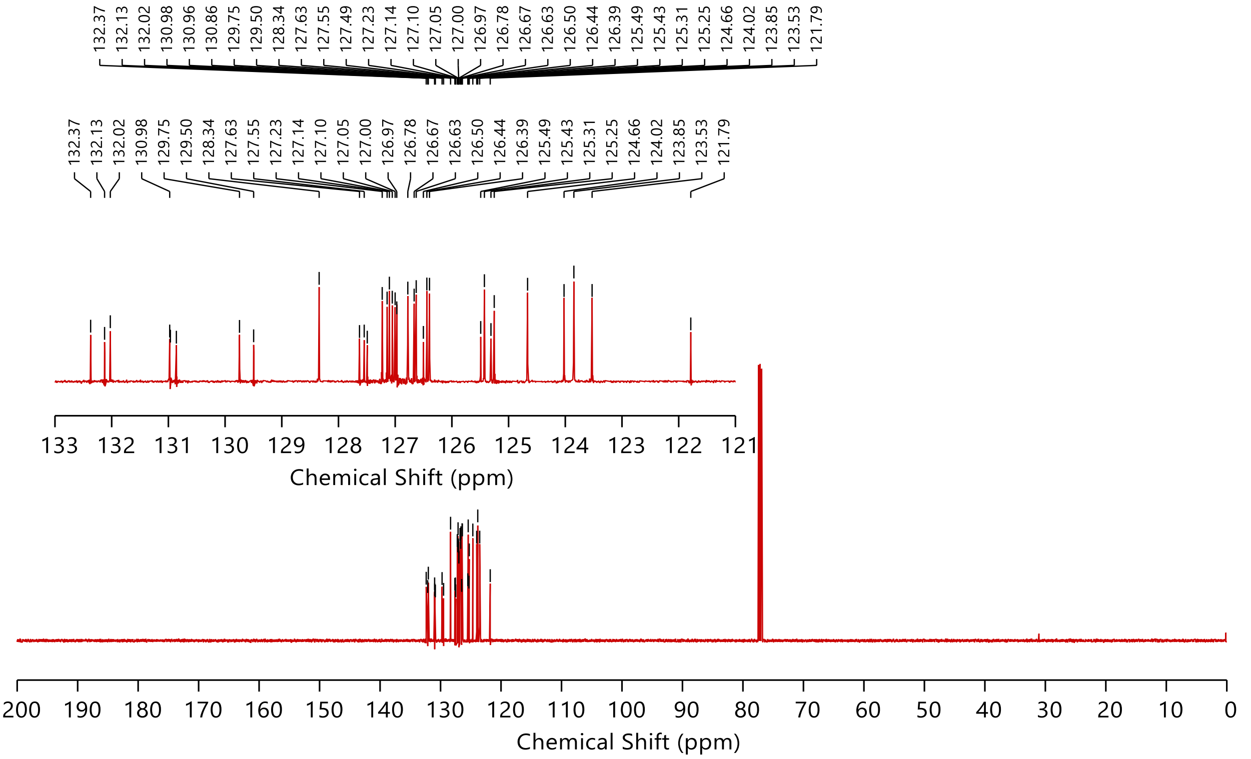

Supplementary Figure 23. $^{13}C\{^{1}H\}$ NMR spectrum of **8H$_{Br}$** (126 MHz, $CDCl_3$, 298 K).

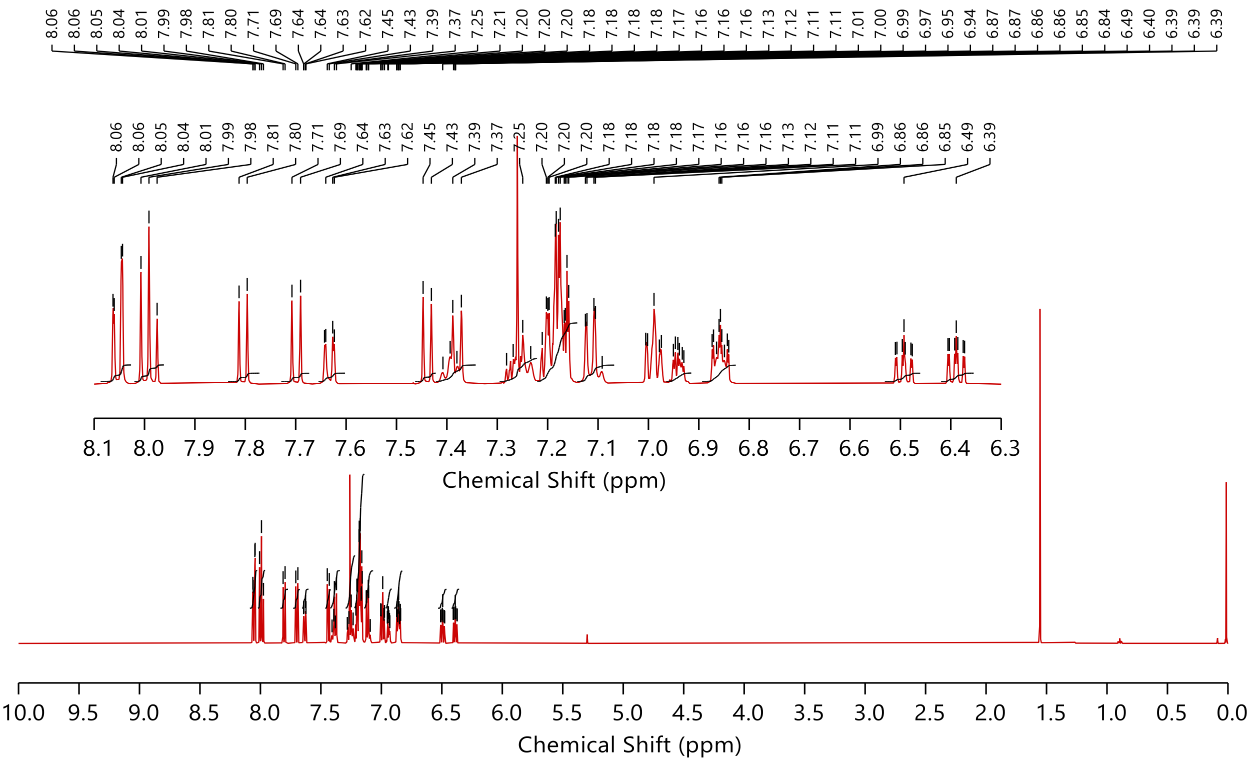

Supplementary Figure 24. ^{1}H NMR spectrum of **8H2py** (500 MHz, $CDCl_3$, 298 K).

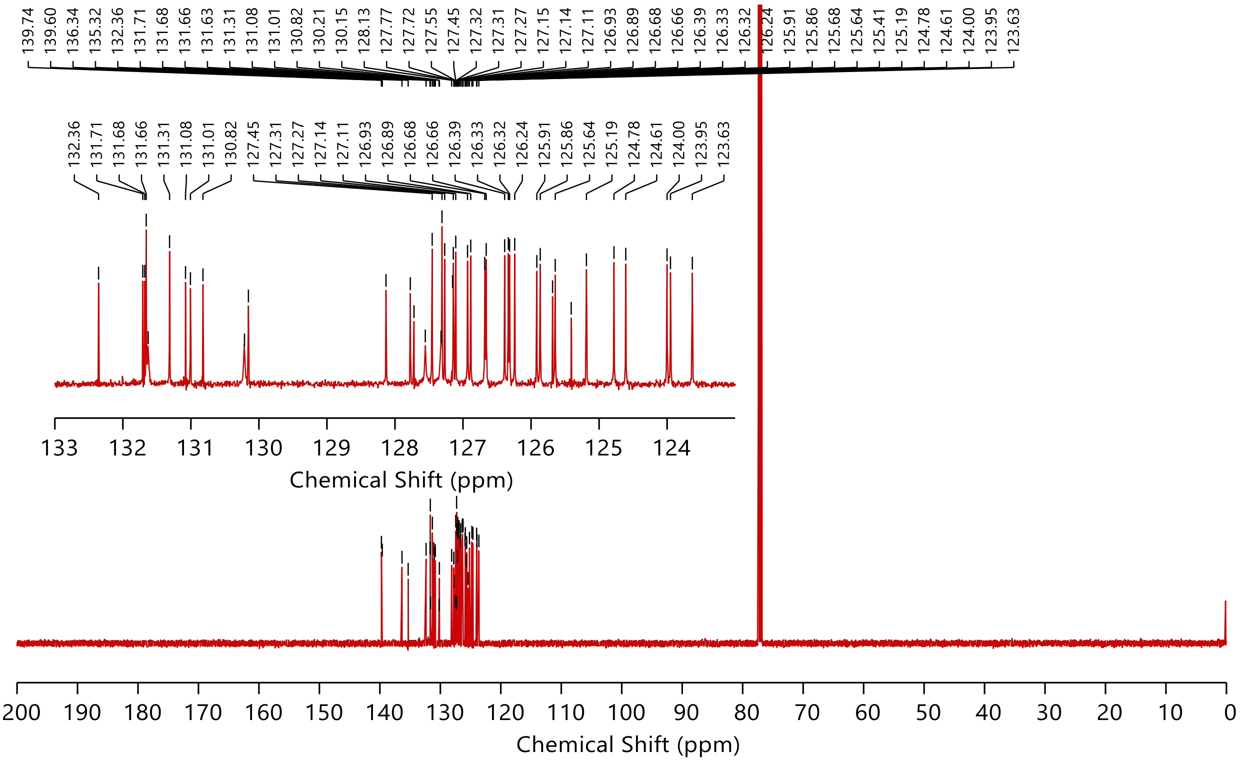

Supplementary Figure 25. $^{13}C\{^{1}H\}$ NMR spectrum of **8H2py** (126 MHz, $CDCl_3$, 298 K).

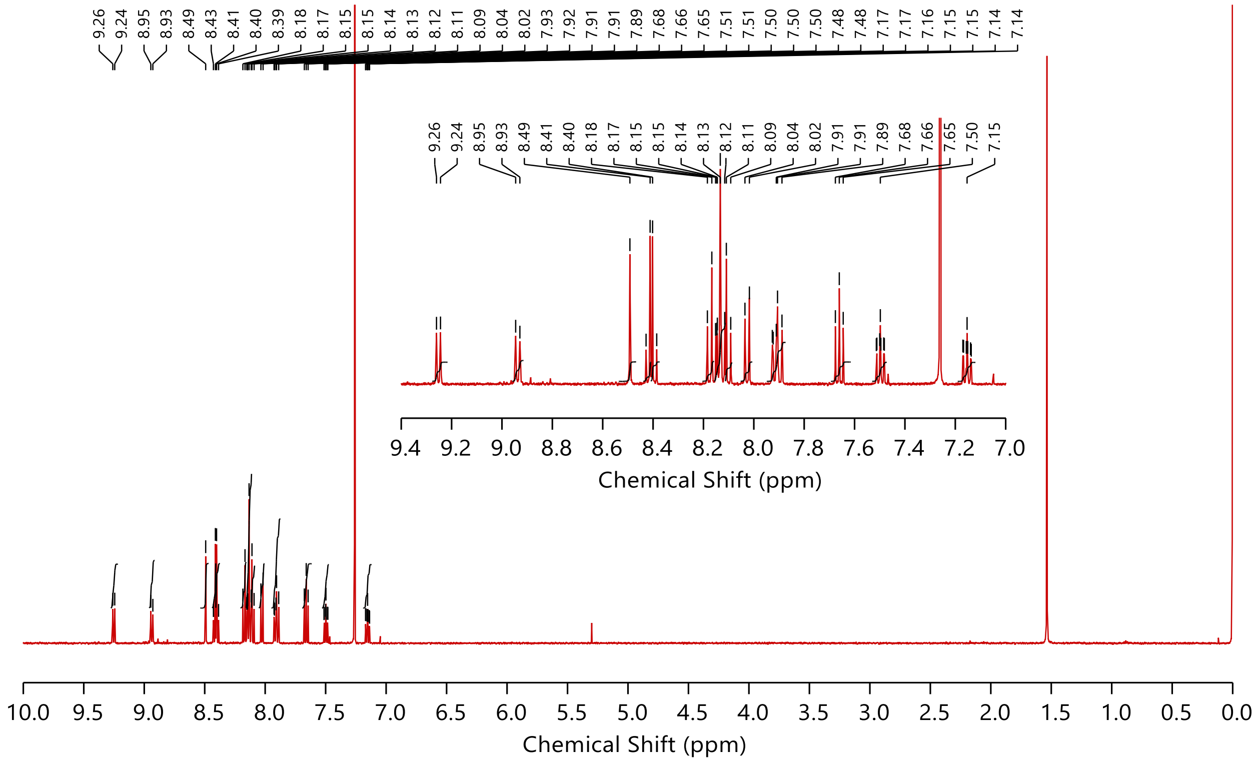

Supplementary Figure 26. ^{1}H NMR spectrum of **O7H** (500 MHz, $CDCl_3$, 298 K).

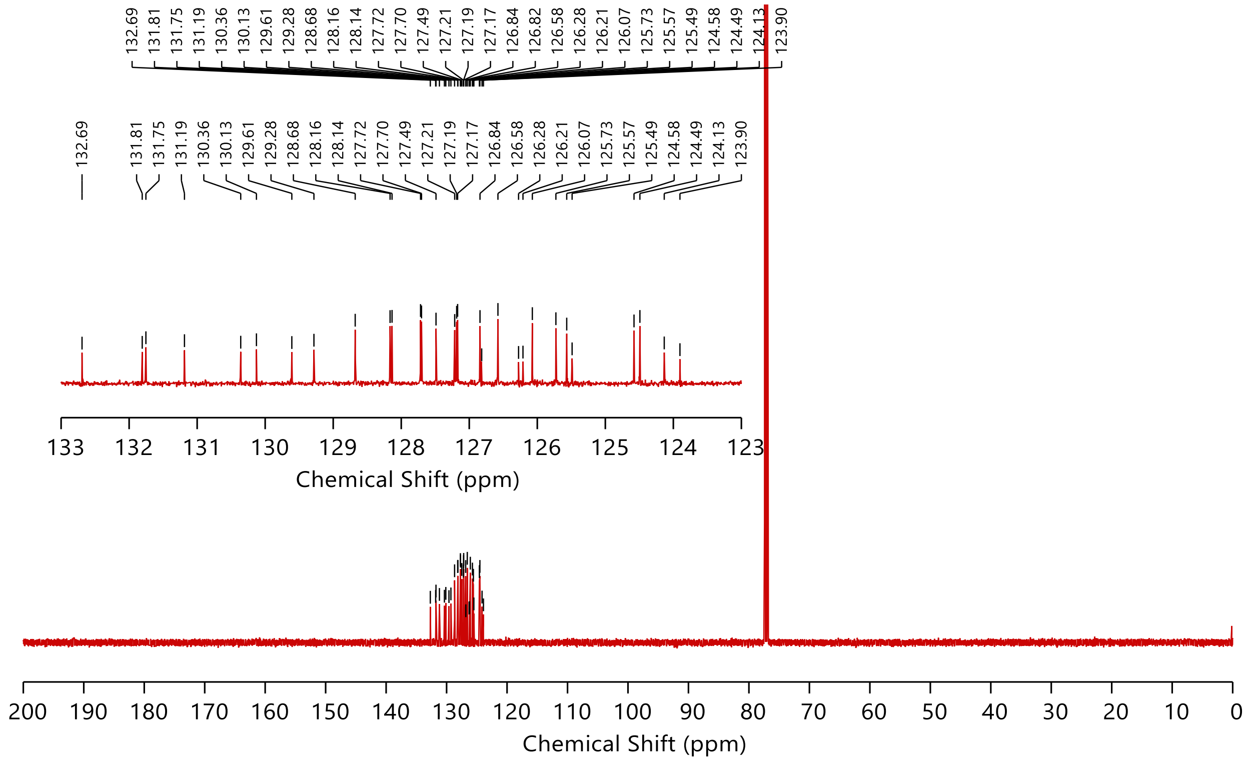

Supplementary Figure 27. $^{13}C\{^{1}H\}$ NMR spectrum of **O7H** (126 MHz, $CDCl_3$, 298 K).

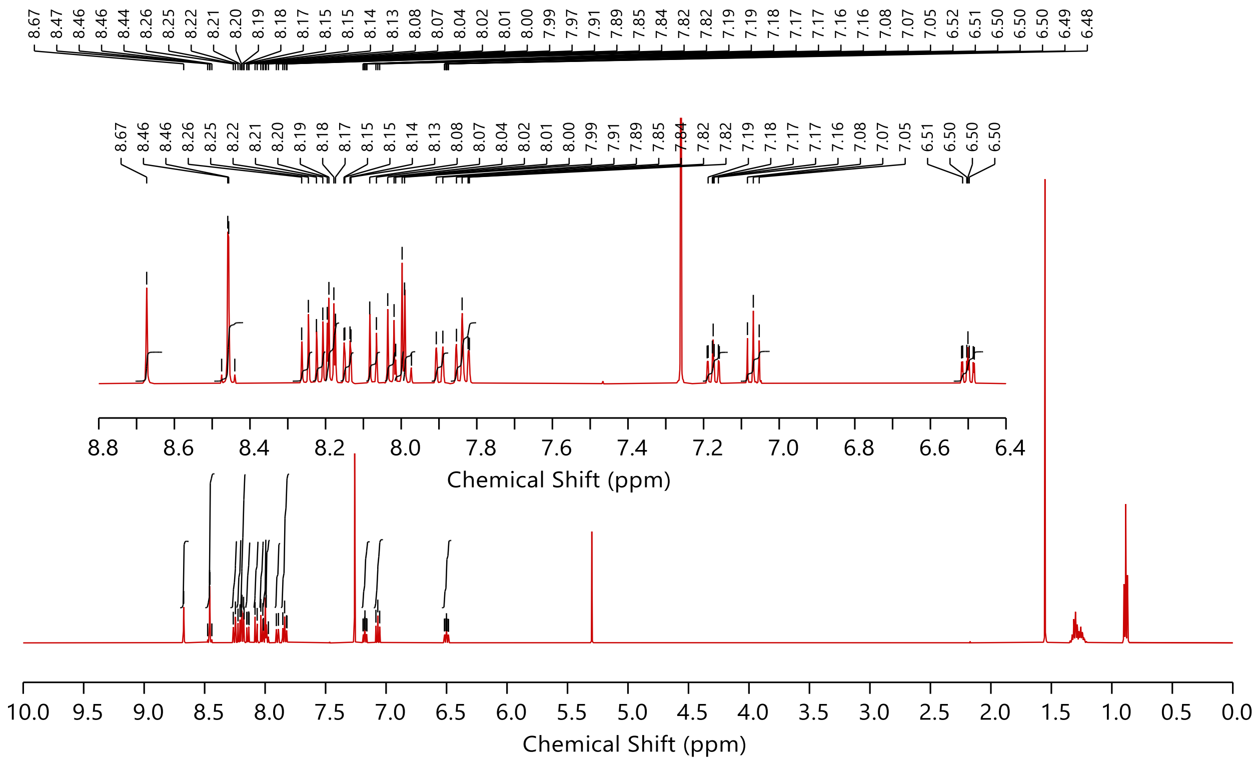

Supplementary Figure 28. ^{1}H NMR spectrum of **O8H** (500 MHz, $CDCl_3$, 298 K).

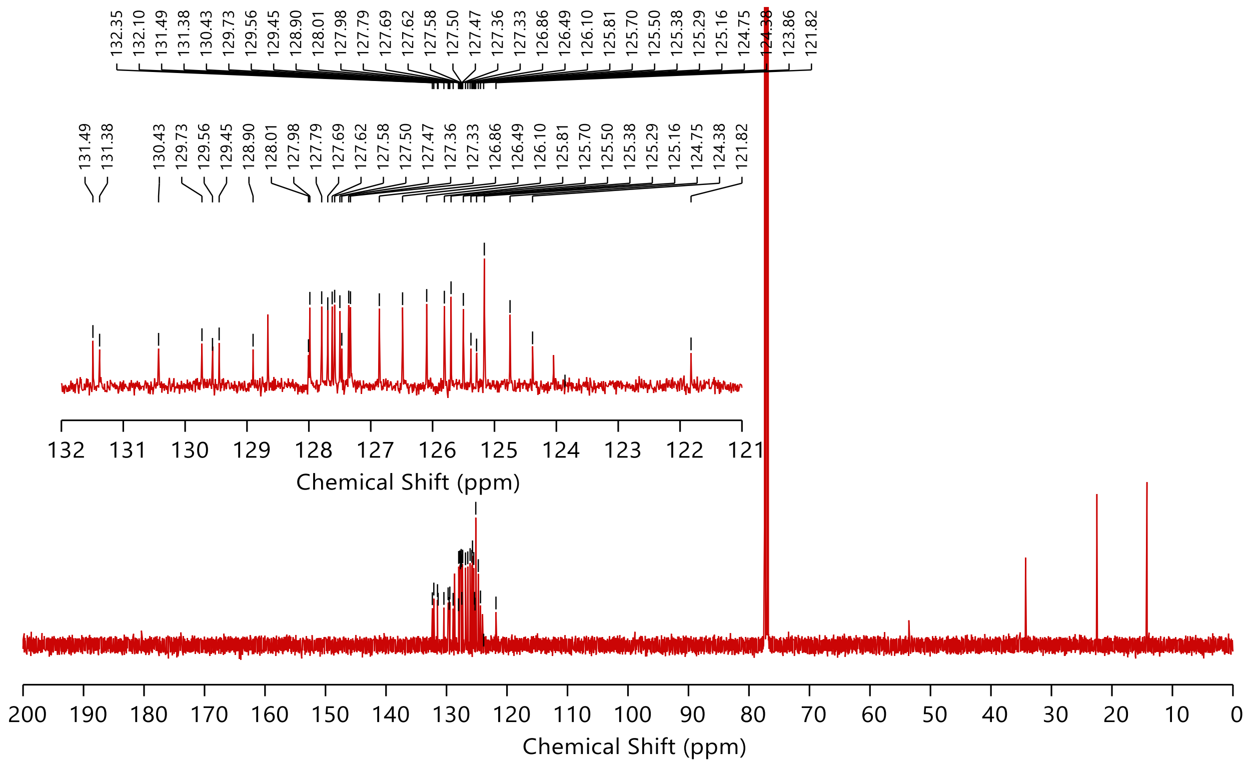

Supplementary Figure 29. $^{13}C\{^{1}H\}$ NMR spectrum of **O8H** (126 MHz, $CDCl_3$, 298 K).

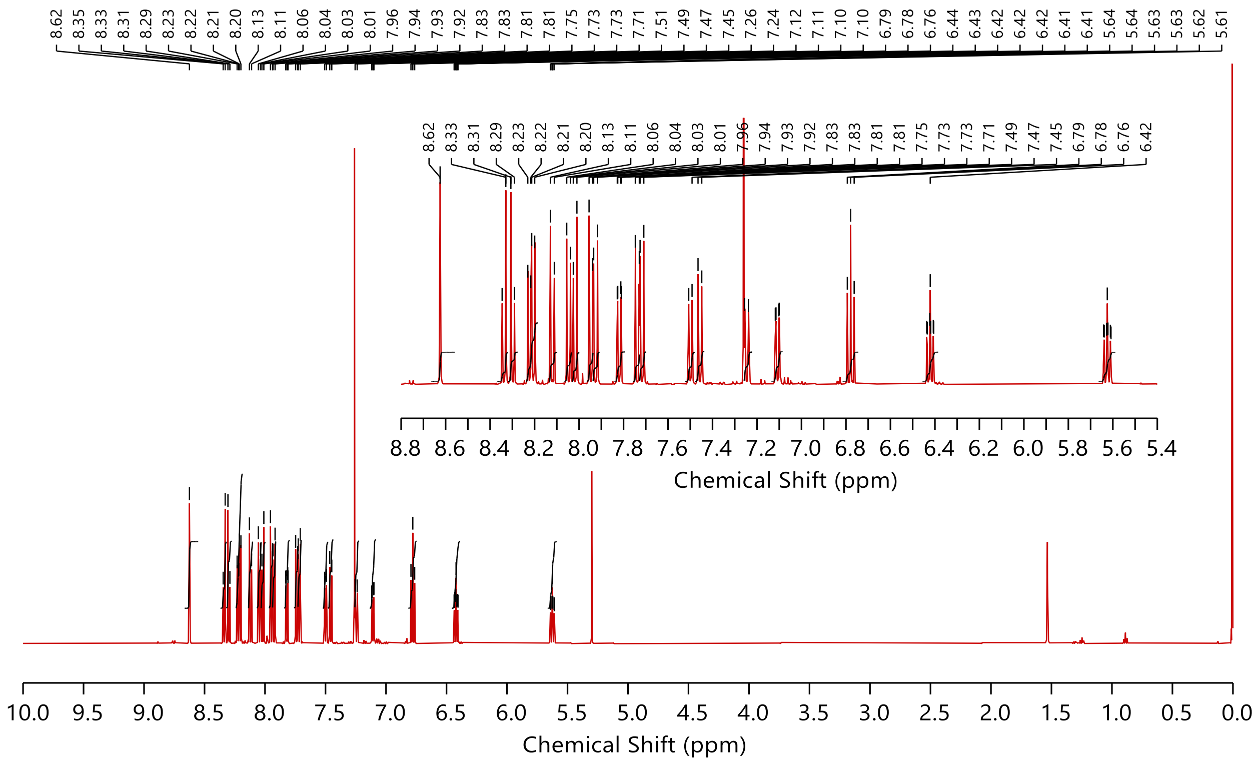

Supplementary Figure 30. ^{1}H NMR spectrum of **O9H** (500 MHz, $CDCl_3$, 298 K).

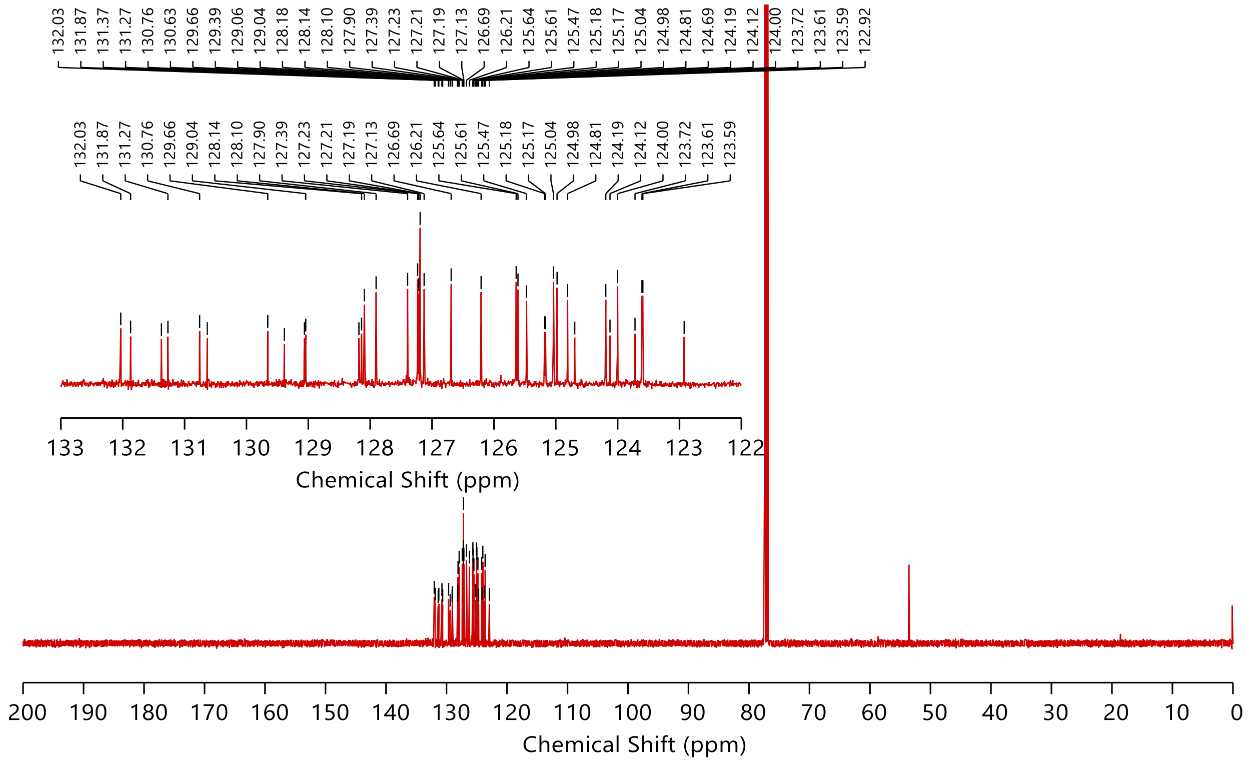

Supplementary Figure 31. ^{13}C{^{1}H} NMR spectrum of **O9H** (126 MHz, $CDCl_3$, 298 K).

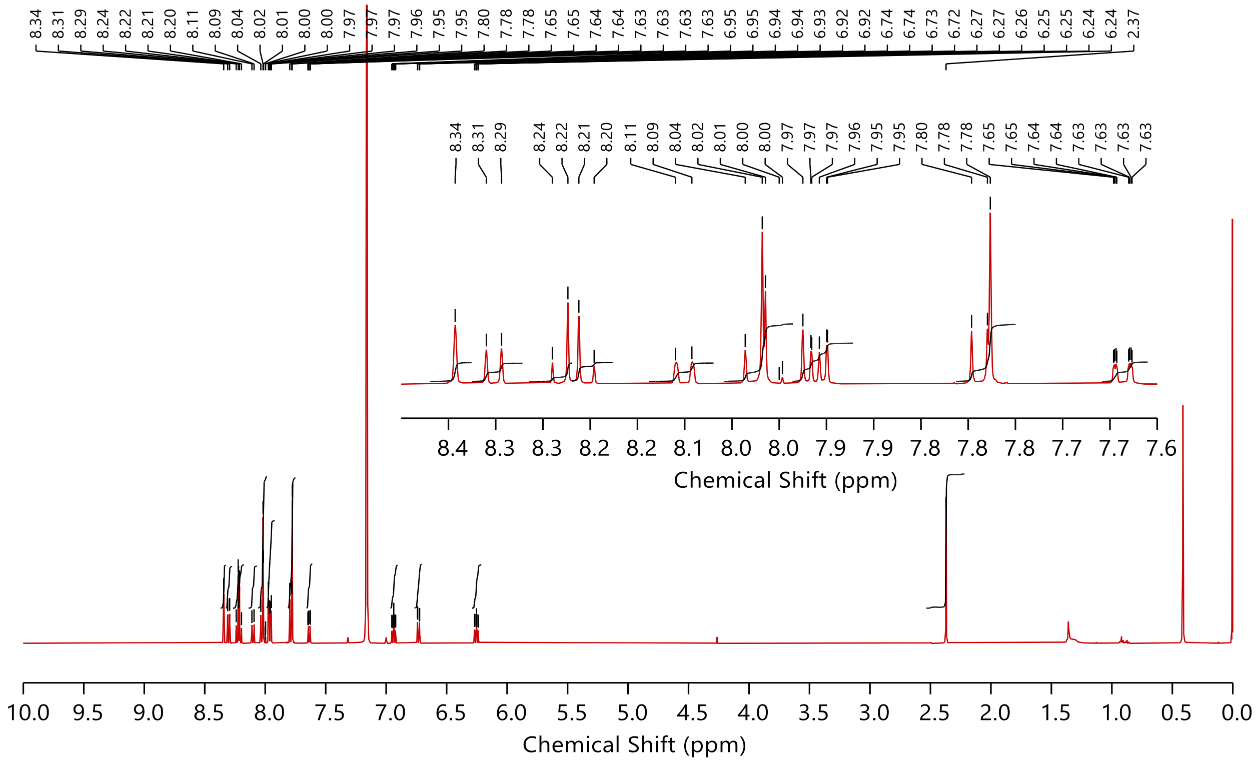

Supplementary Figure 32. ^{1}H NMR spectrum of **O8H$_{CH3}$** (500 MHz, benzene-d_6, 298 K).

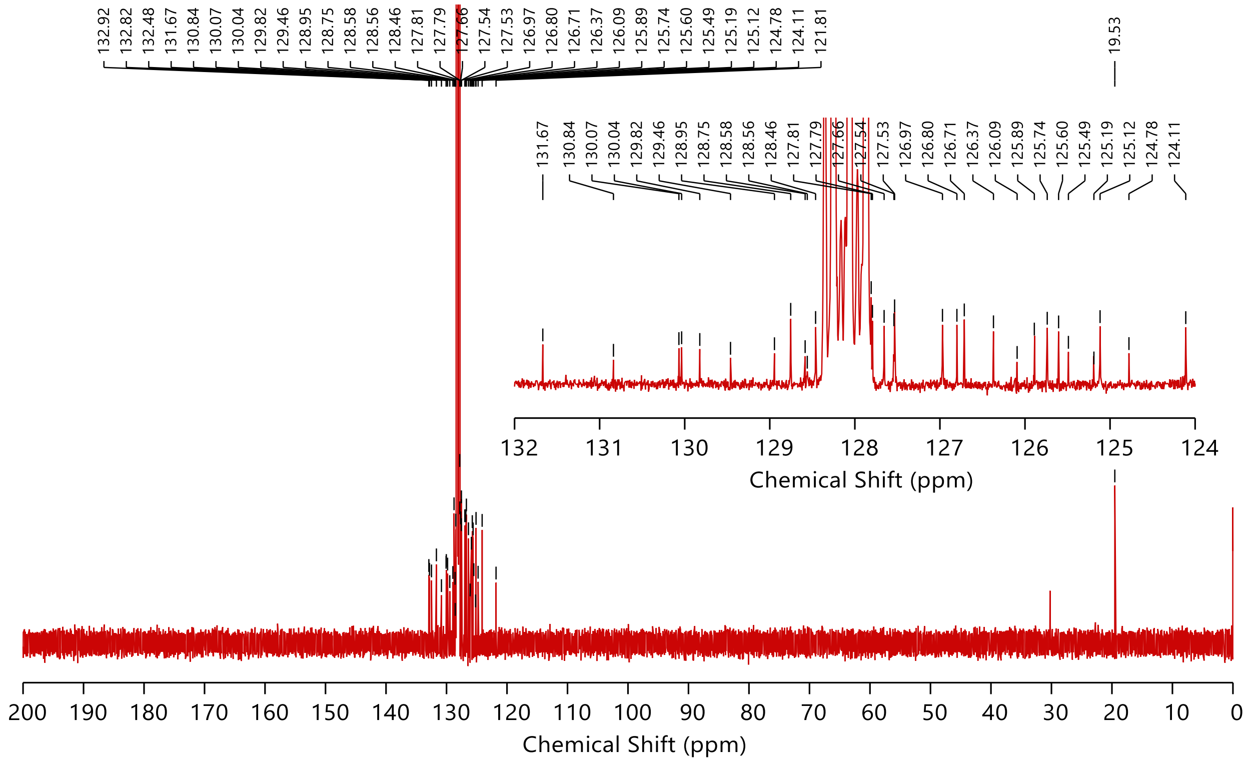

Supplementary Figure 33. ^{13}C{^{1}H} NMR spectrum of **O8H$_{CH3}$** (126 MHz, benzene-d_6, 298 K).

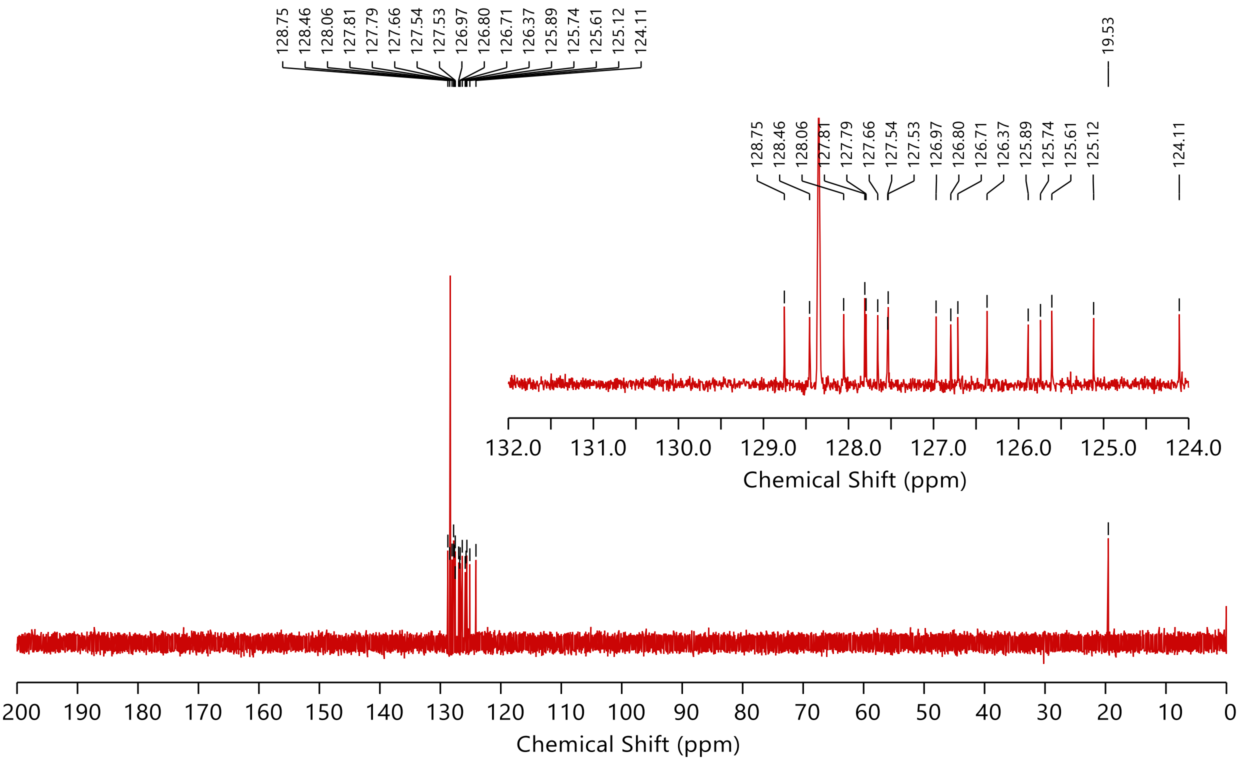

Supplementary Figure 34. DEPT-135 NMR spectrum of **O8H**$_{\mathbf{CH3}}$ (126 MHz, benzene-d_6, 298 K).

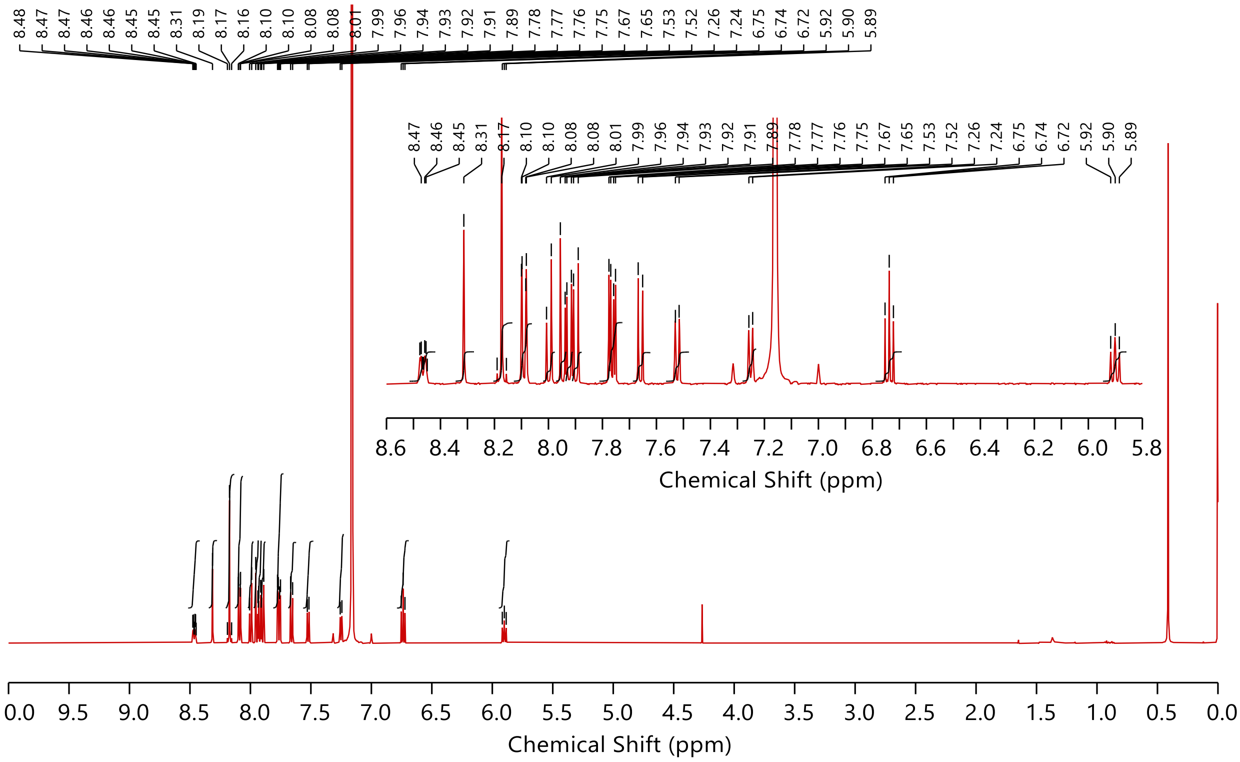

Supplementary Figure 35. ^{1}H NMR spectrum of **O8H**$_{\mathbf{CF3}}$ (500 MHz, $CDCl_3$, 298 K).

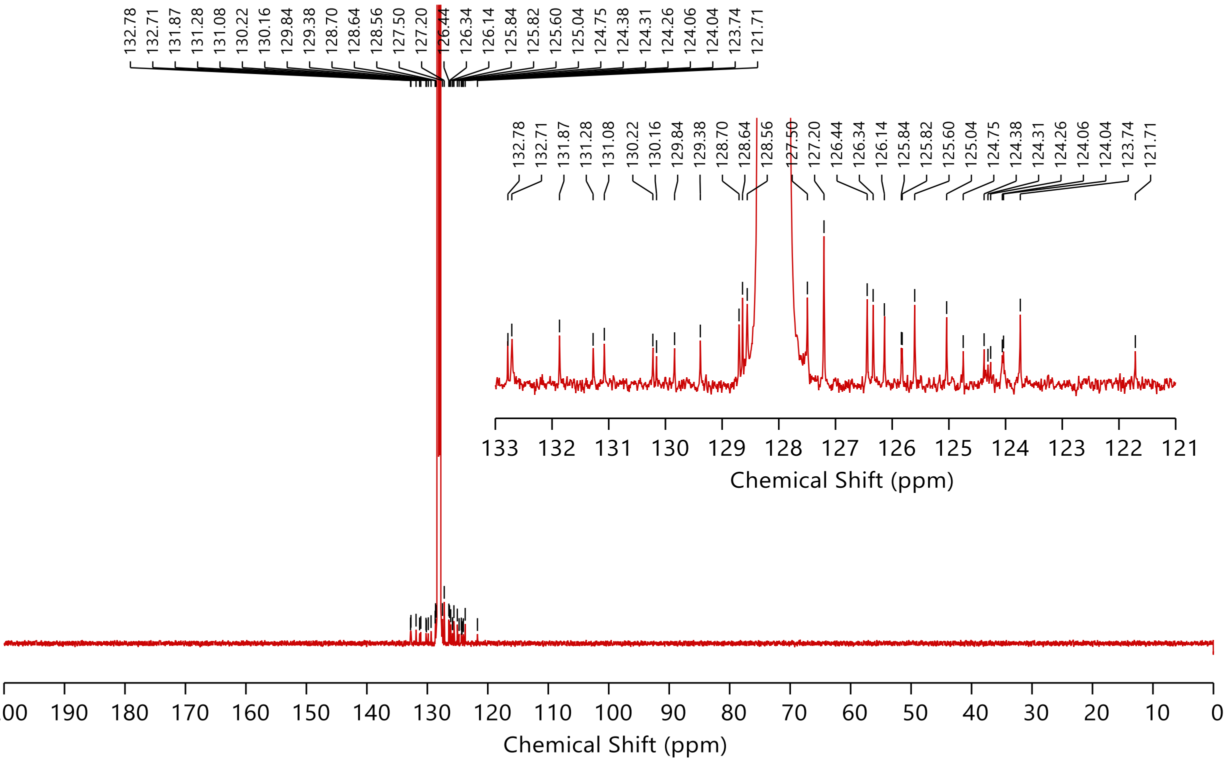

Supplementary Figure 36. $^{13}C\{^{1}H\}$ NMR spectrum of **O8H$_{CF3}$** (126 MHz, $CDCl_3$, 298 K).

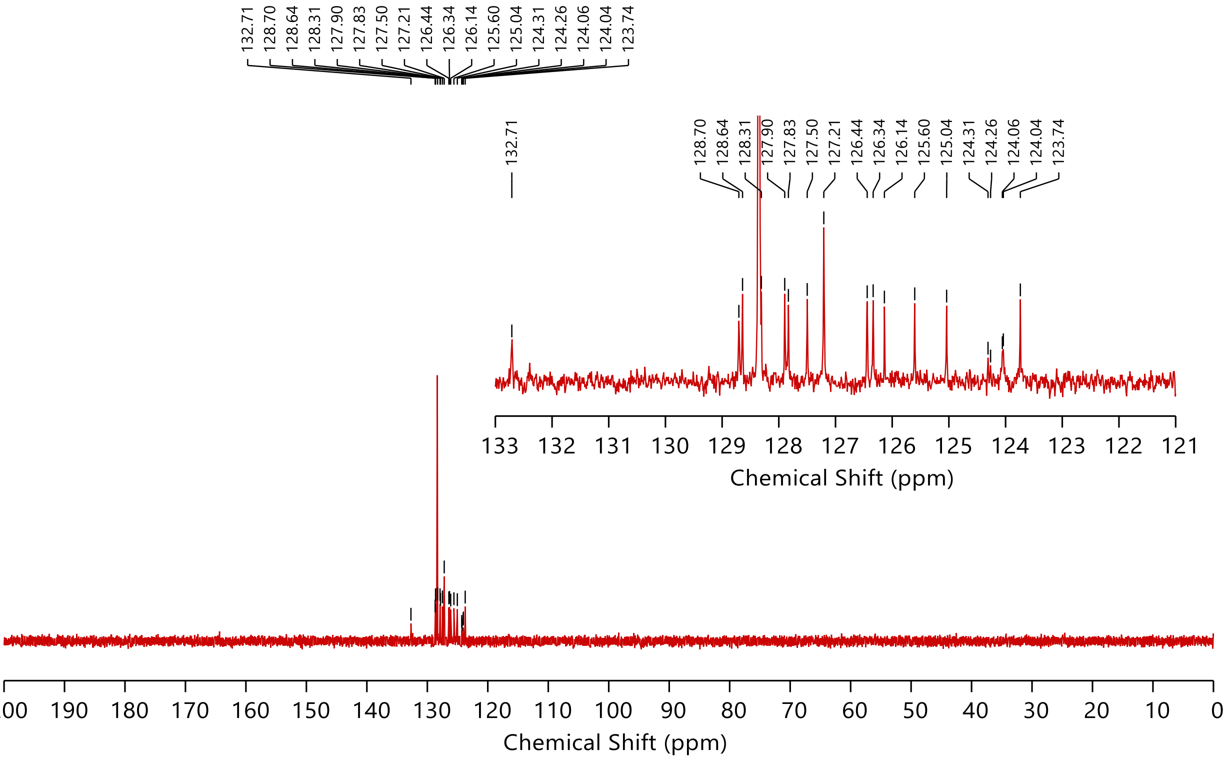

Supplementary Figure 37. DEPT-135 NMR spectrum of **O8H$_{CF3}$** (126 MHz, $CDCl_3$, 298 K).

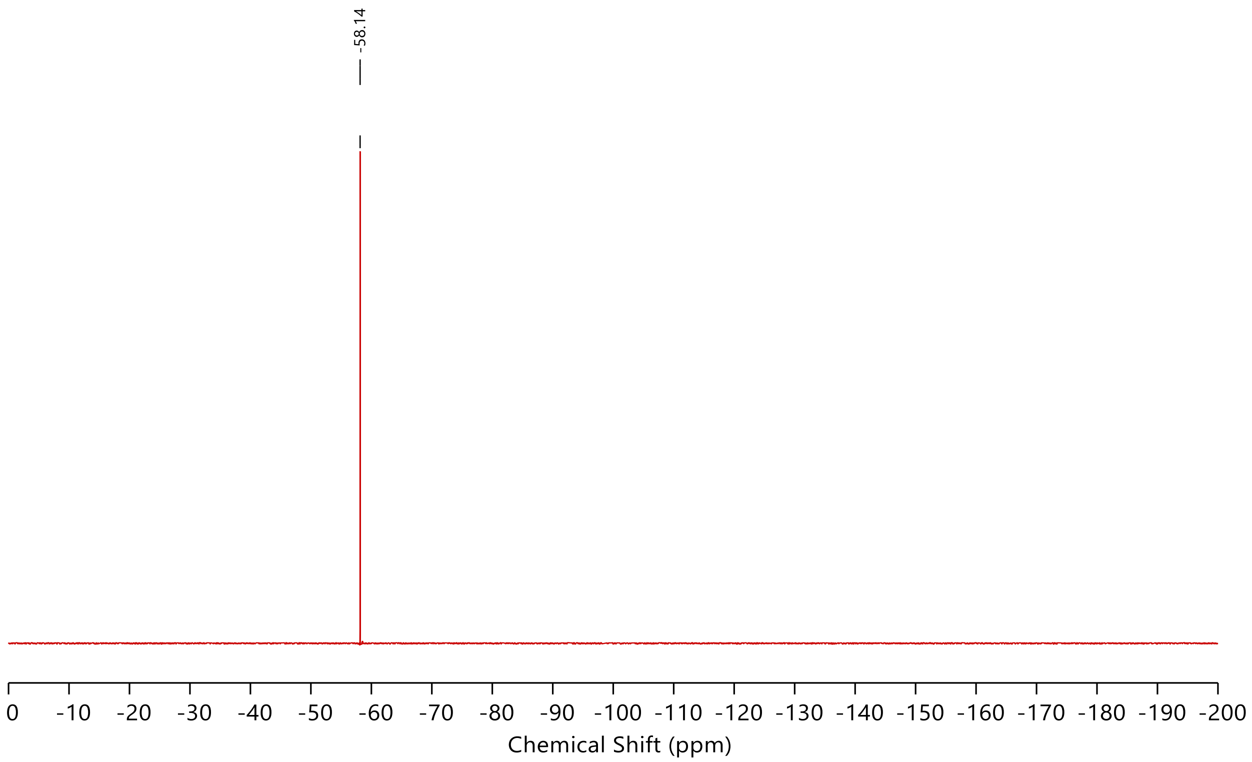

Supplementary Figure 38. $^{19}F\{^{1}H\}$ NMR spectrum of **O8H$_{CF3}$** (471 MHz, $CDCl_3$, 298 K).

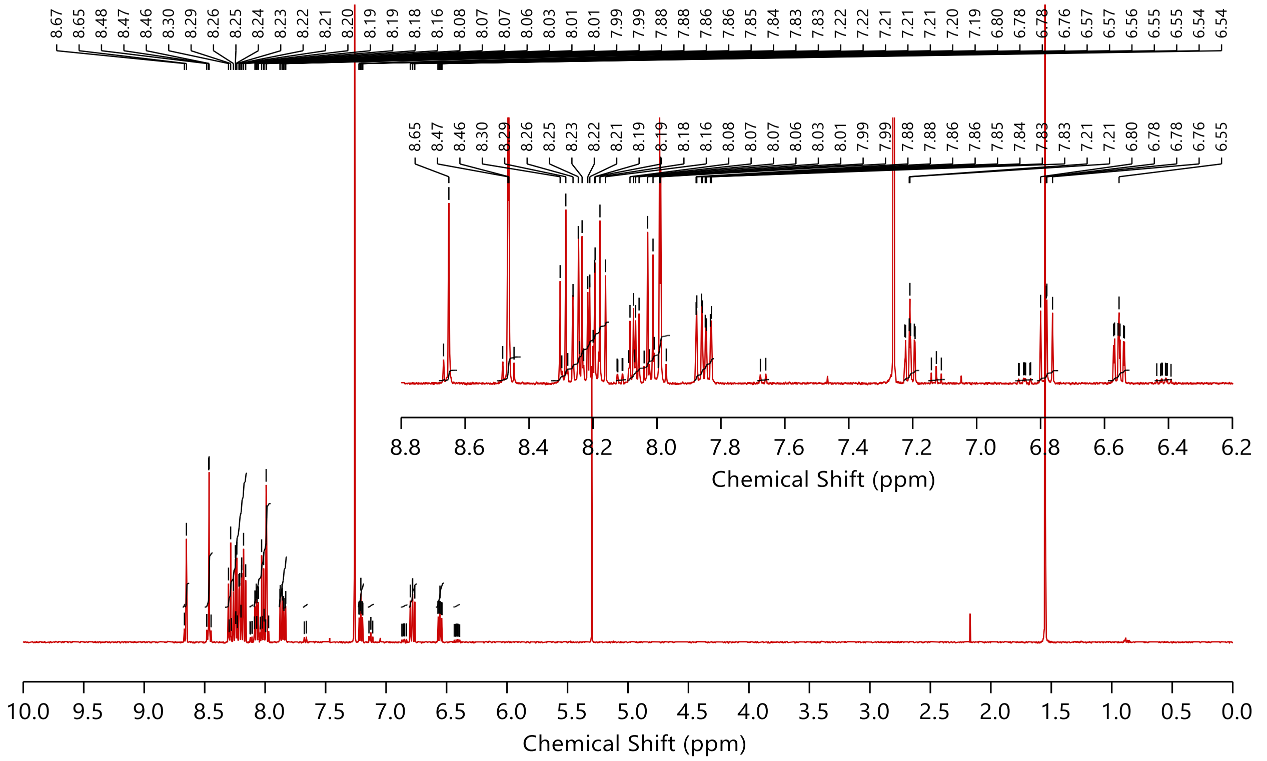

Supplementary Figure 39. ^{1}H NMR spectrum of **O8H$_F$-α** and **O8H$_F$-β** mixture (500 MHz, $CDCl_3$, 298 K).

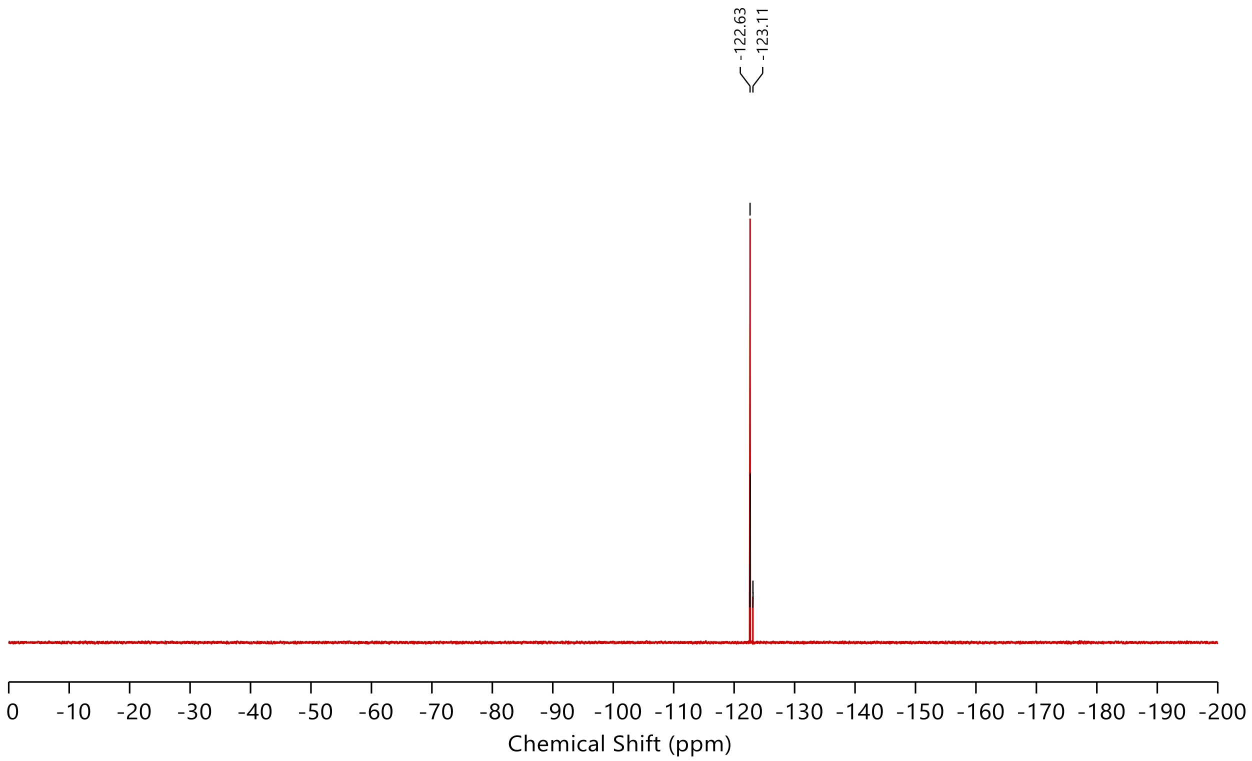

Supplementary Figure 40. $^{19}F\{^{1}H\}$ NMR spectrum of **O8H$_F$-α** and **O8H$_F$-β** mixture (471 MHz, $CDCl_3$, 298 K).

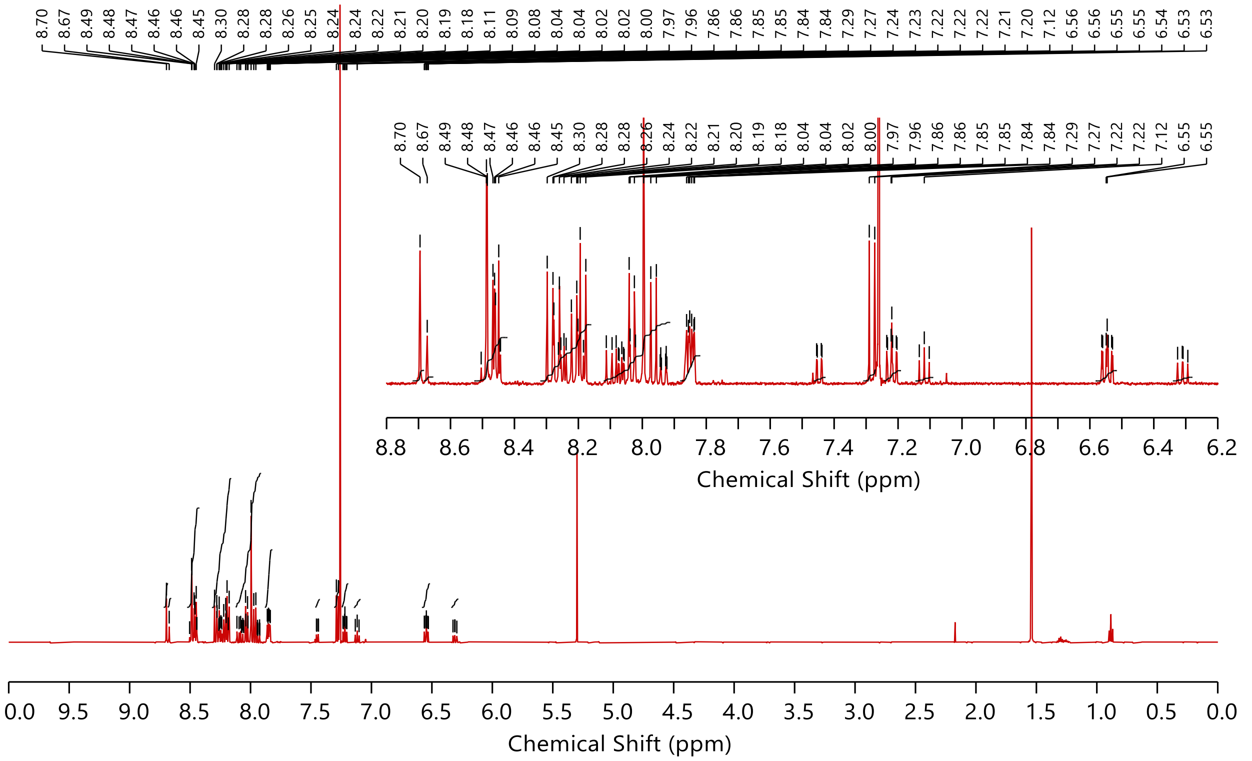

Supplementary Figure 41. ^{1}H NMR spectrum of **O8H$_{Br}$-α** and **O8H$_{Br}$-β** mixture (500 MHz, $CDCl_3$, 298 K).

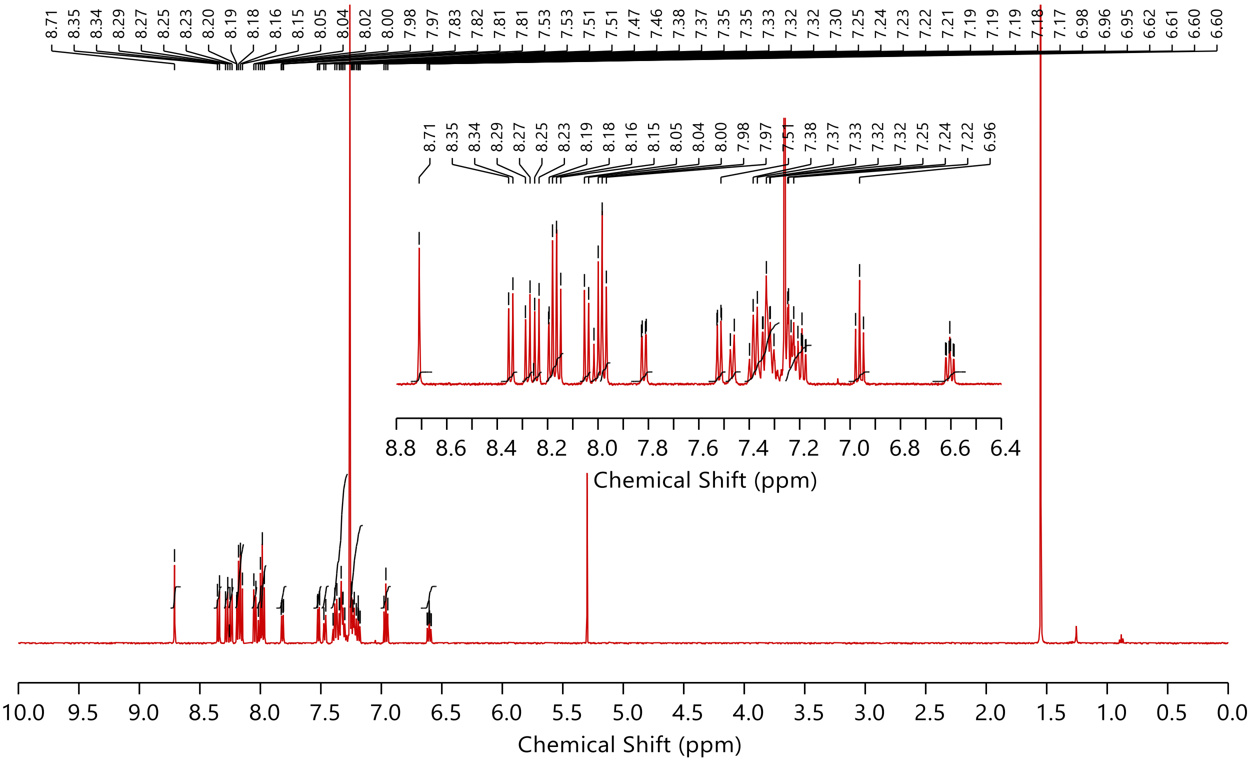

Supplementary Figure 42. ^{1}H NMR spectrum of **O8H$_{2py}$** (500 MHz, $CDCl_3$, 298 K).

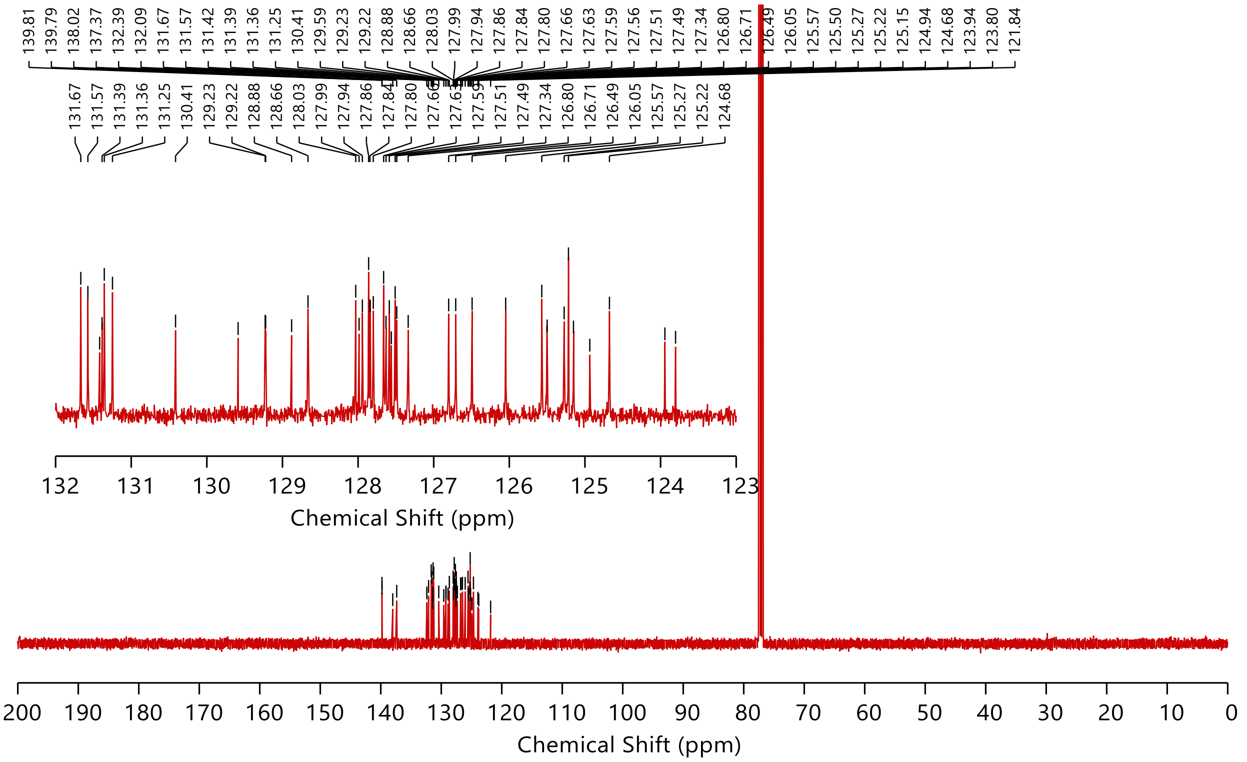

Supplementary Figure 43. $^{13}C\{^{1}H\}$ NMR spectrum of **O8H$_{2py}$** (126 MHz, $CDCl_3$, 298 K).

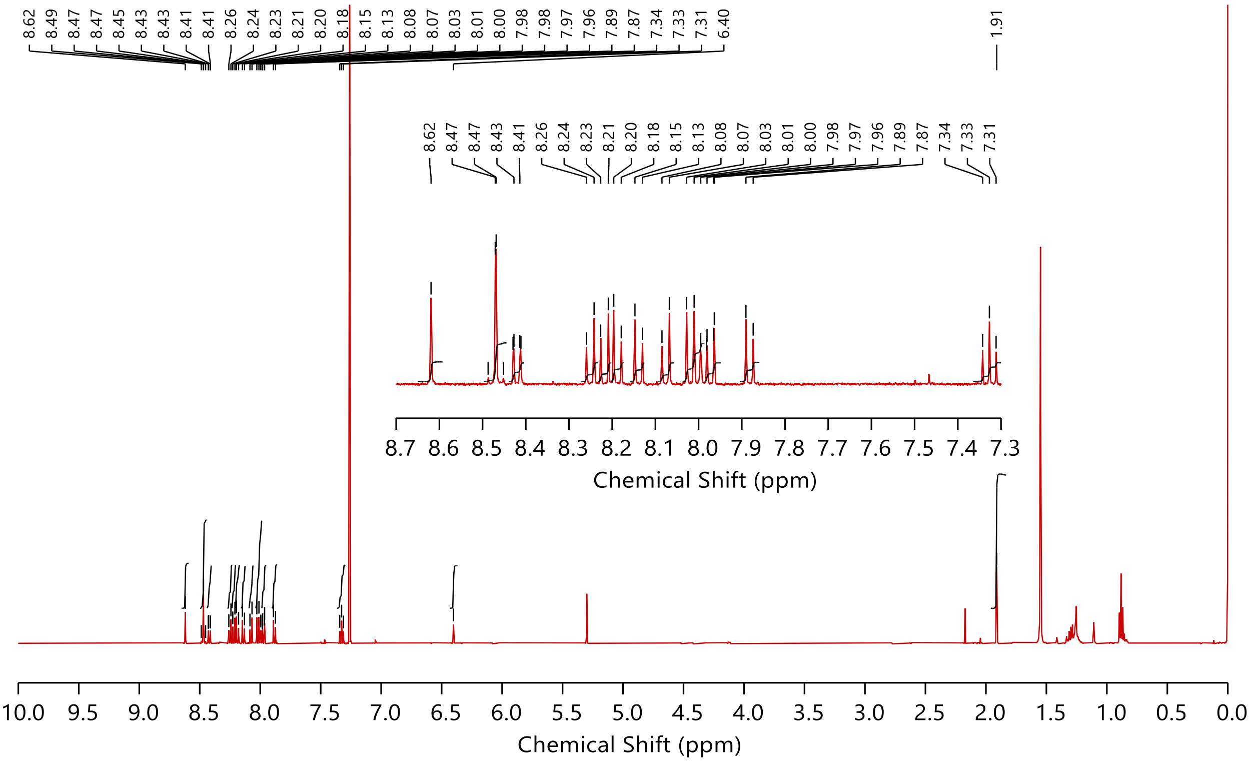

Supplementary Figure 44. ^{1}H NMR spectrum of **O8H$_{mt}$** (500 MHz, $CDCl_3$, 298 K).

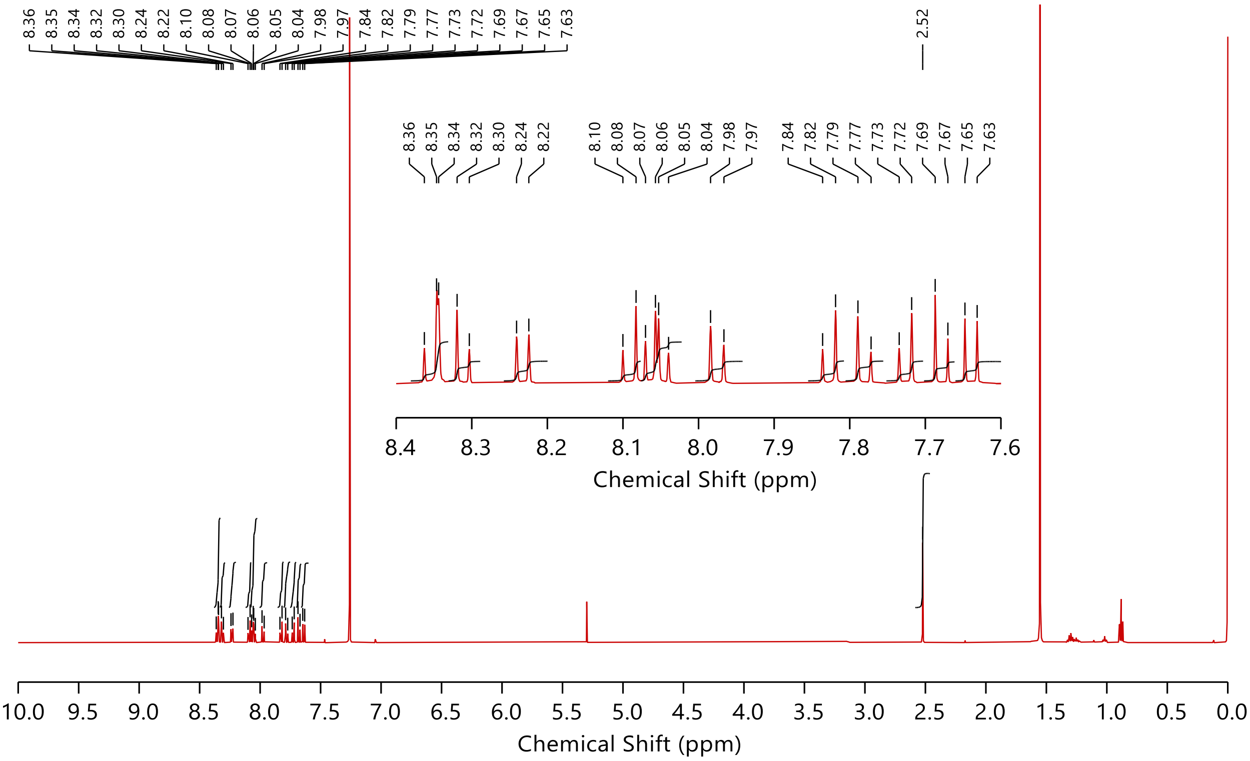

Supplementary Figure 45. ^{1}H NMR spectrum of **OO8H$_{mt}$** (500 MHz, $CDCl_3$, 298 K).

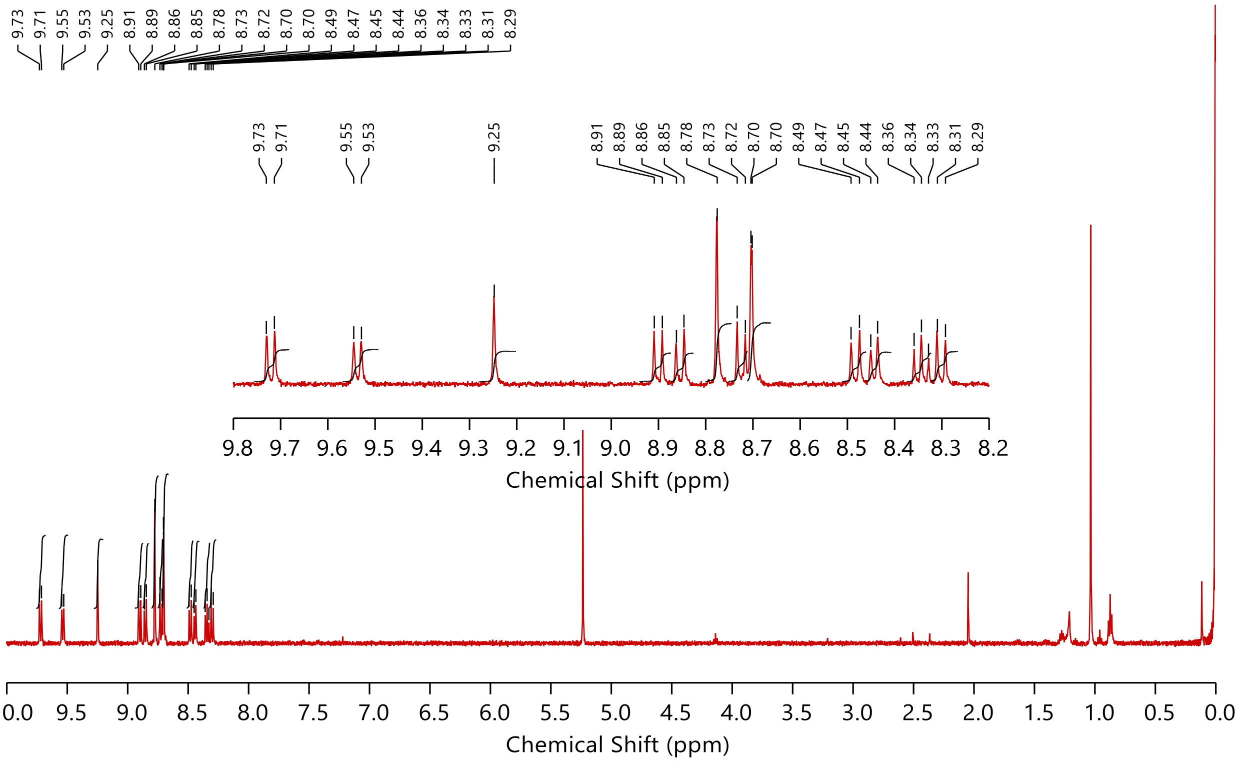

Supplementary Figure 46. ^{1}H NMR spectrum of **OO7H** (500 MHz, CS_2, 298 K).

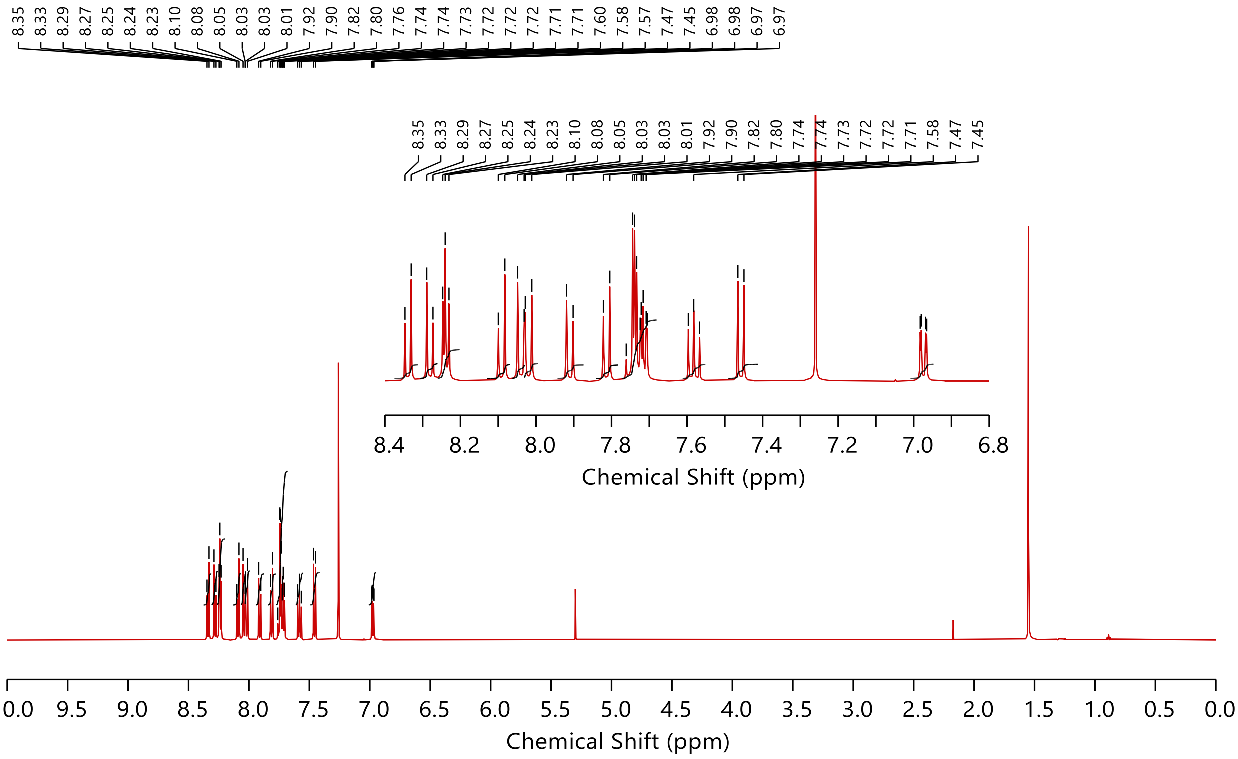

Supplementary Figure 47. ^{1}H NMR spectrum of **OO8H** (500 MHz, $CDCl_3$, 298 K).

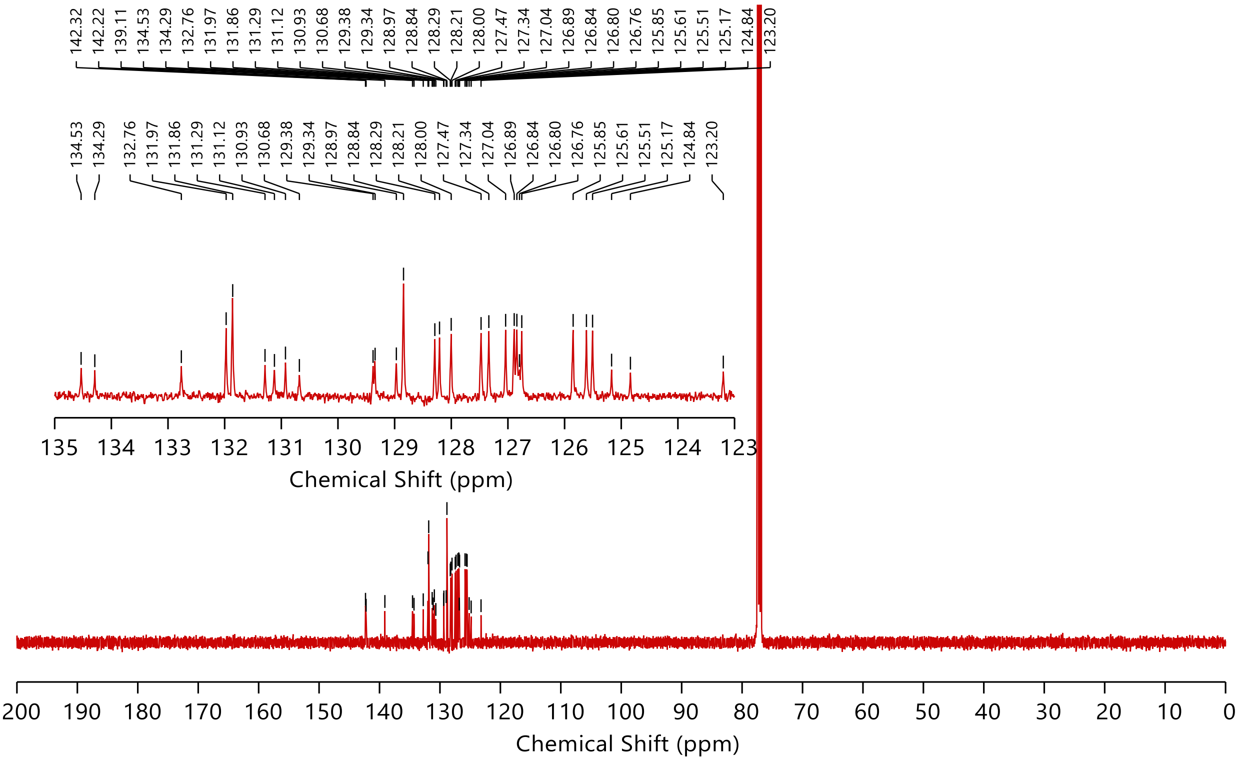

Supplementary Figure 48. $^{13}C\{^{1}H\}$ NMR spectrum of **OO8H** (126 MHz, $CDCl_3$, 298 K).

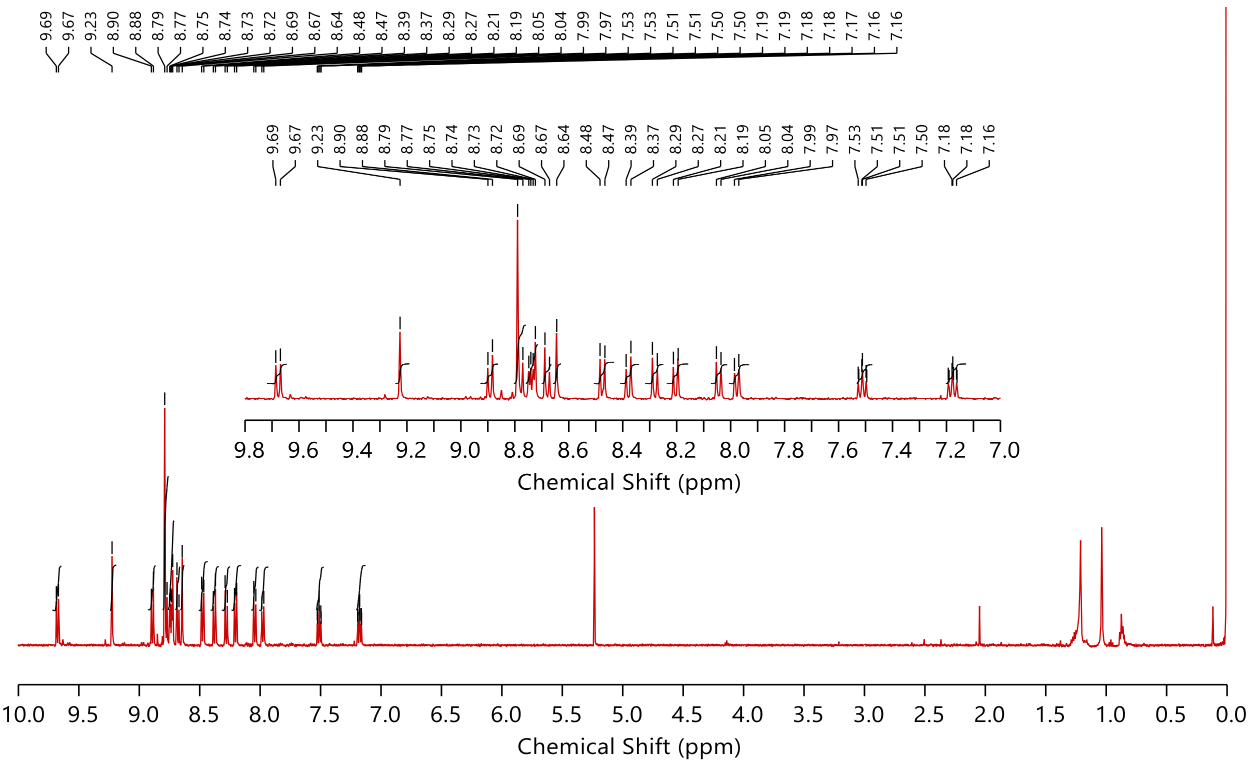

Supplementary Figure 49. ^{1}H NMR spectrum of **OO9H** (500 MHz, CS_2, 298 K).

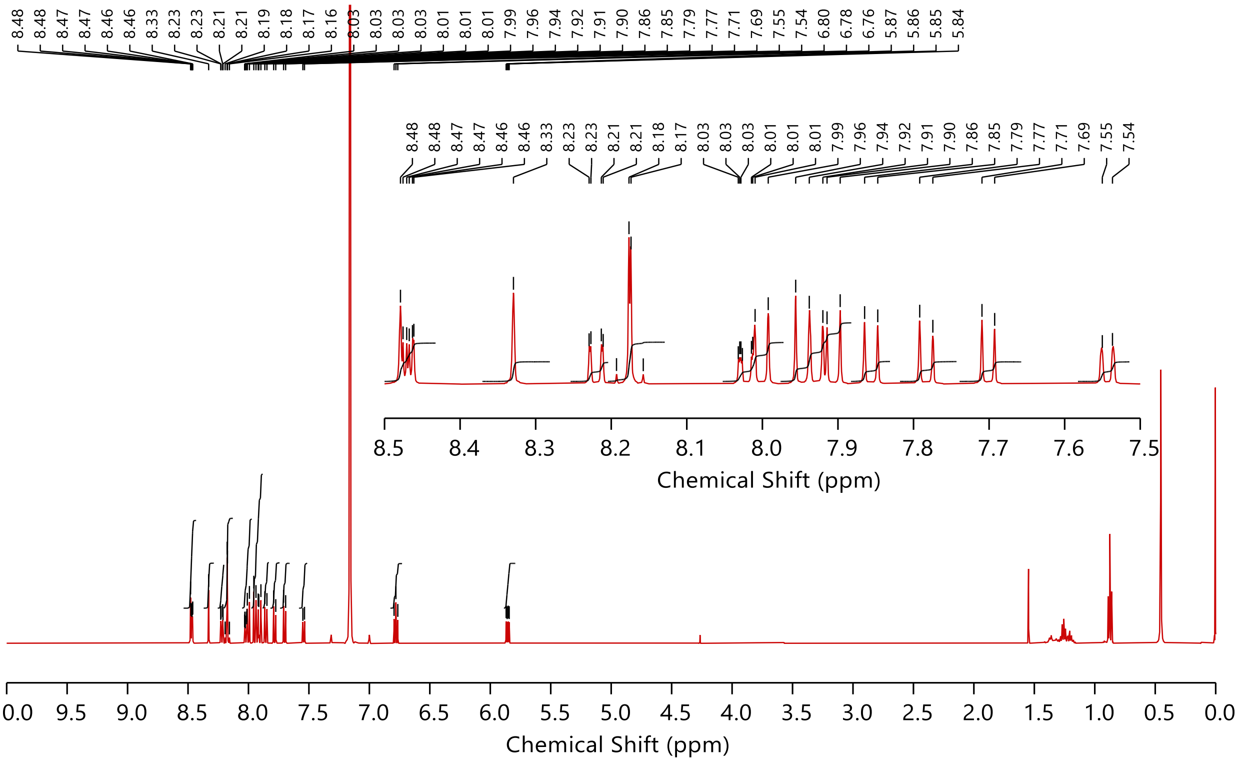

Supplementary Figure 50. ^{1}H NMR spectrum of **O8H$_a$** (500 MHz, benzene-d_6, 298 K).

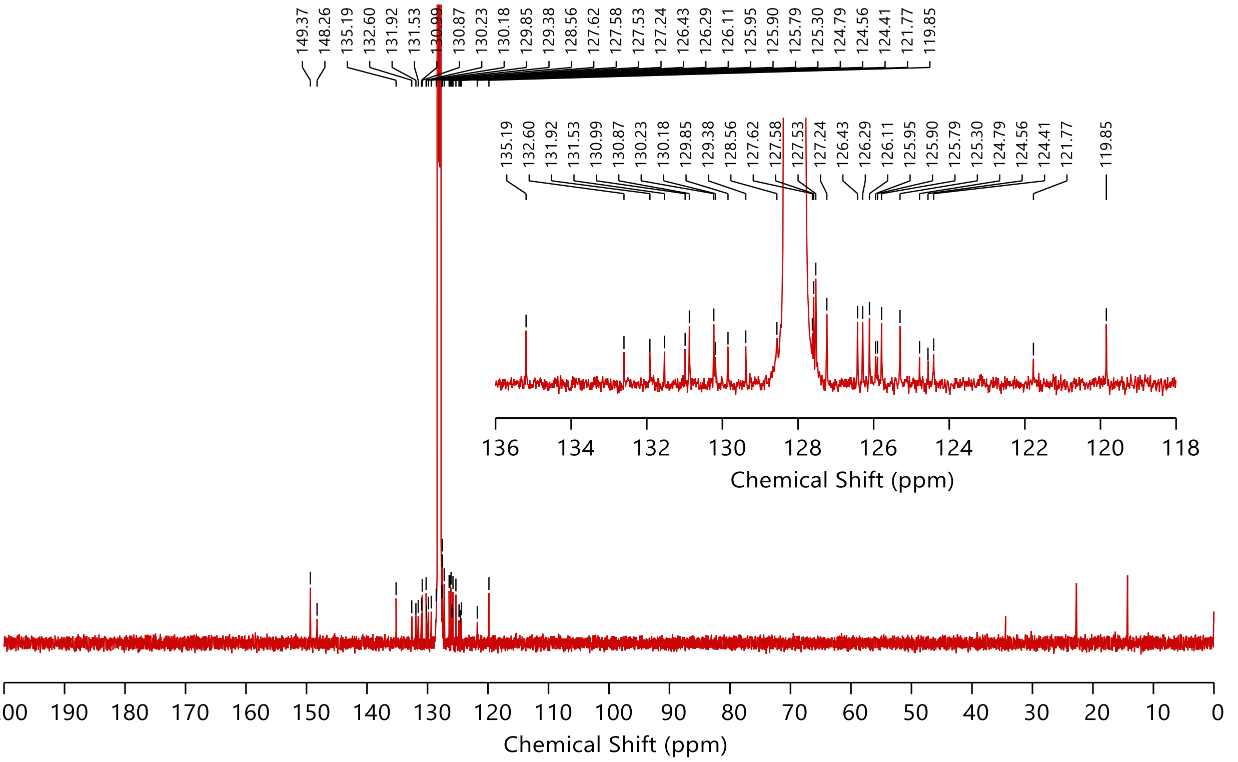

Supplementary Figure 51. $^{13}C\{^{1}H\}$ NMR spectrum of **O8H$_a$** (126 MHz, benzene-d_6, 298 K).

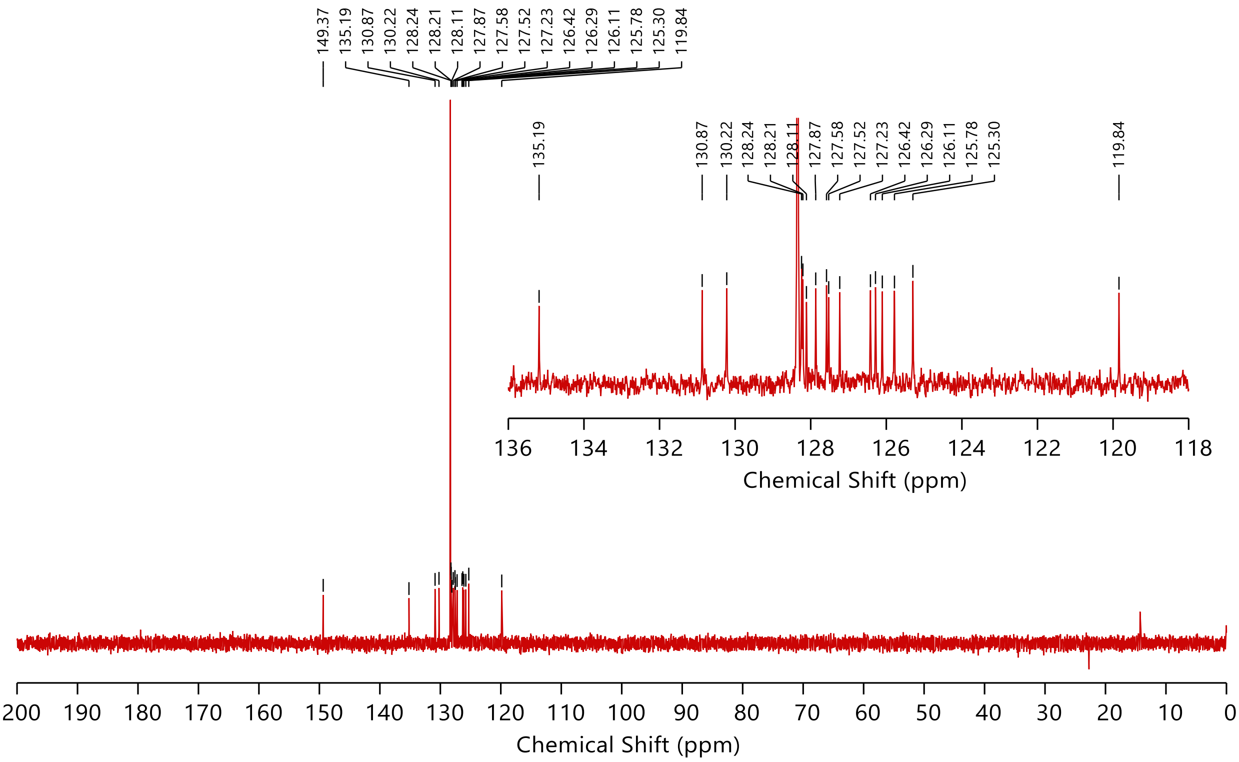

Supplementary Figure 52. DEPT-135 NMR spectrum of **O8Ha** (126 MHz, $CDCl_3$, 298 K).

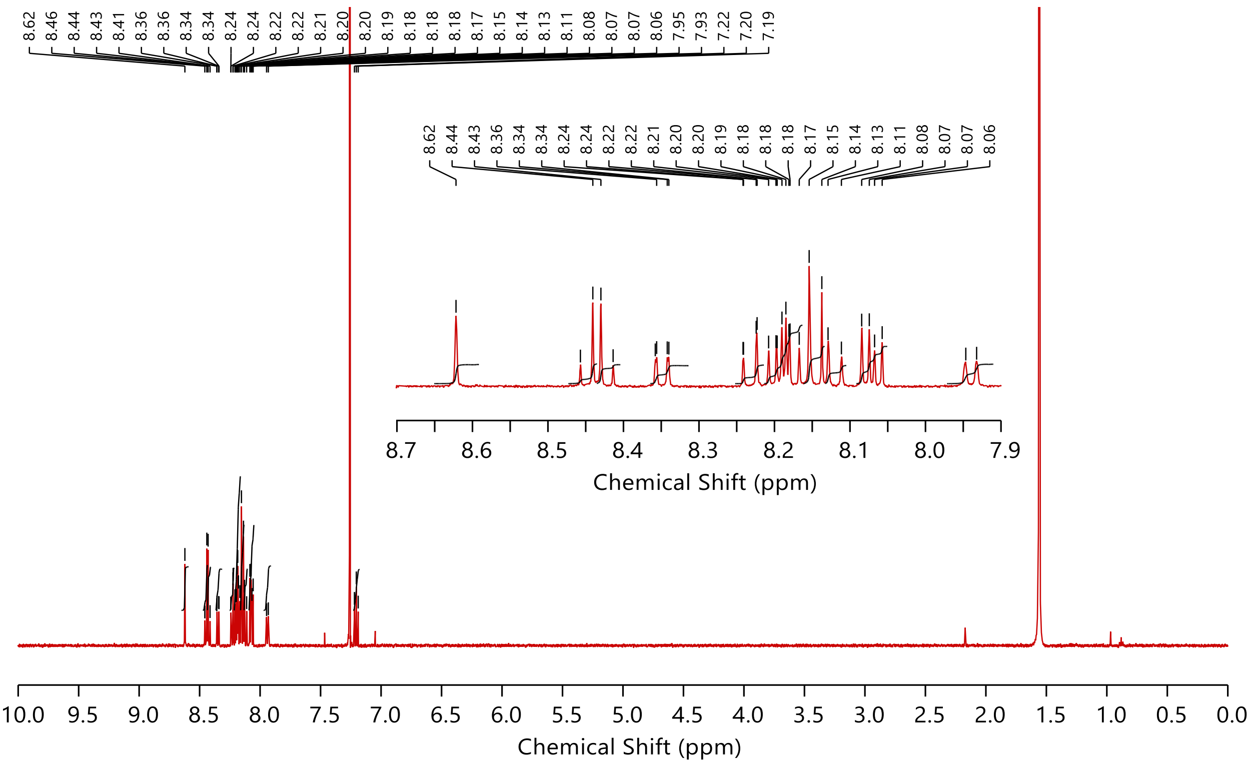

Supplementary Figure 53. ^{1}H NMR spectrum of **O8Hta** (500 MHz, $CDCl_3$, 298 K).

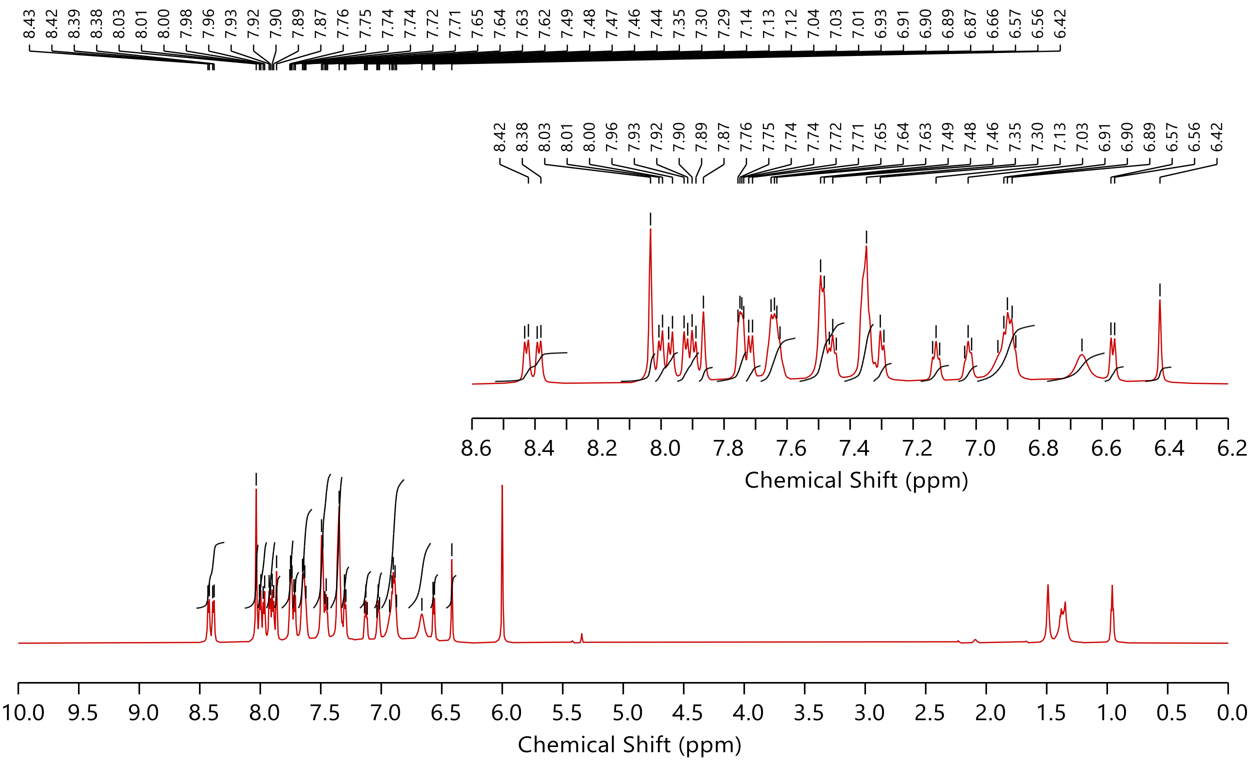

Supplementary Figure 54. ^{1}H NMR spectrum of **6H$_{DPT}$** (700 MHz, 1,1,2,2-tetrachloroethane-d_2, 298 K).

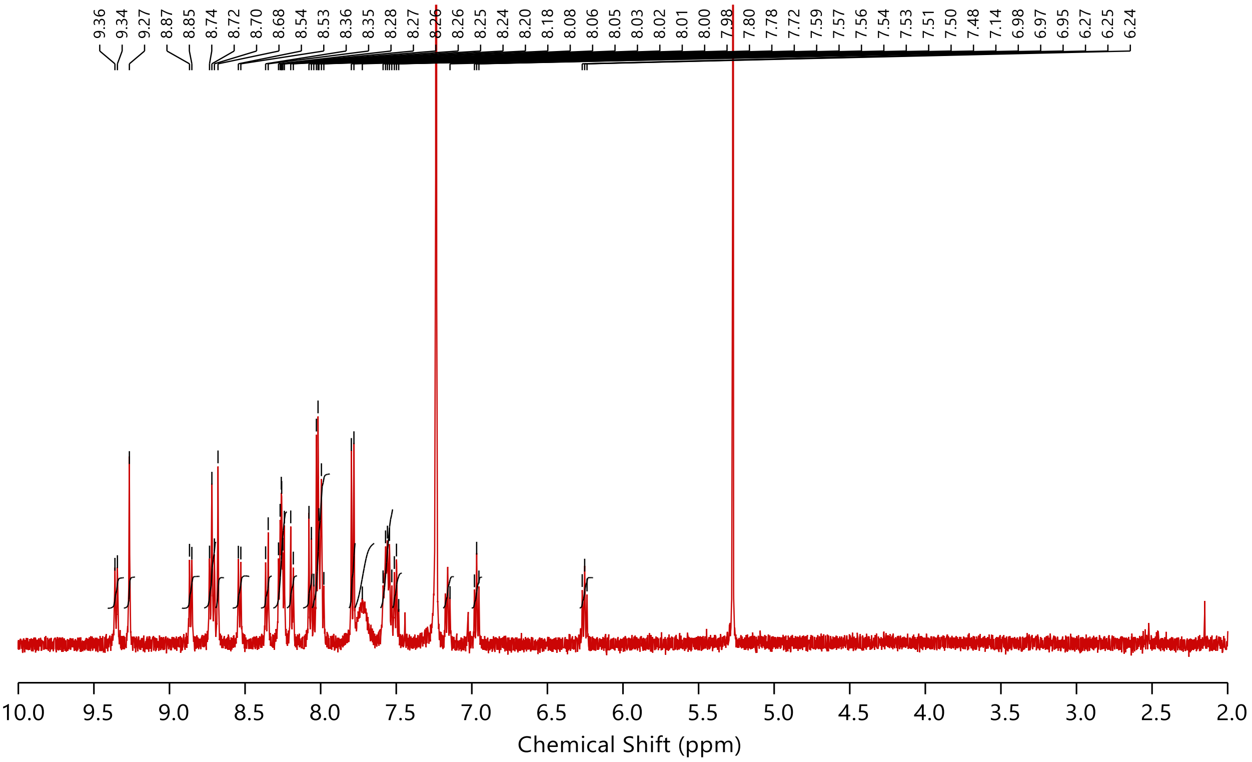

Supplementary Figure 55. ^{1}H NMR spectrum of **O6H$_{DPT}$** (500 MHz, $CDCl_3/CS_2$ = 1/1, v/v, 298 K).

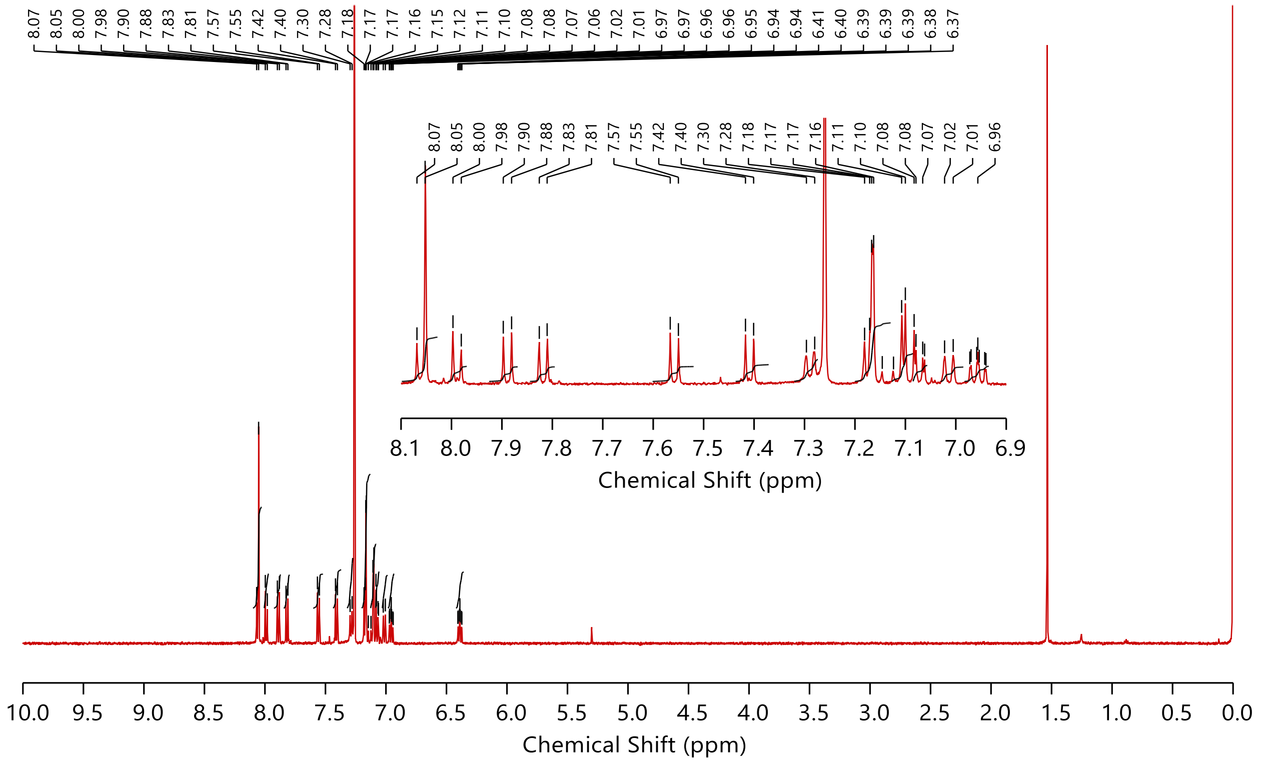

Supplementary Figure 56. ^{1}H NMR spectrum of **8H'$_{Br}$** (500 MHz, $CDCl_3$, 298 K).

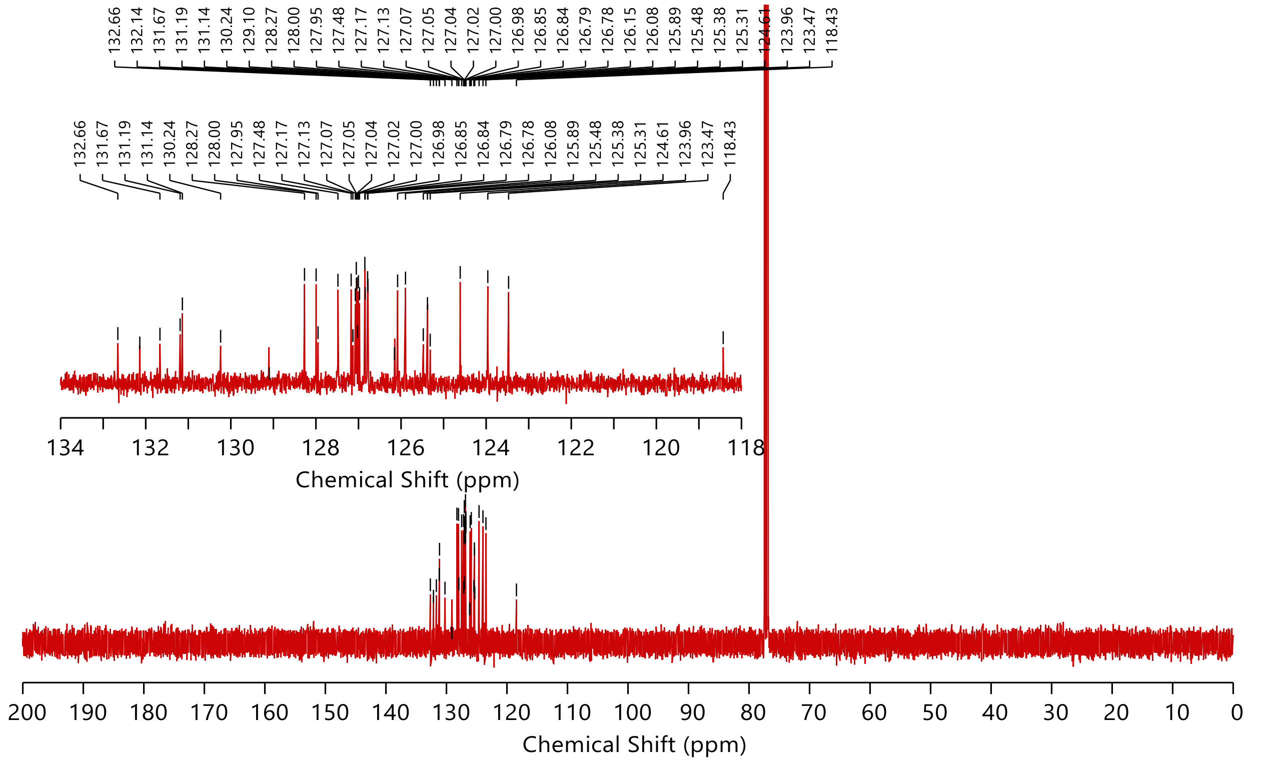

Supplementary Figure 57. $^{13}C\{^{1}H\}$ NMR spectrum of **8H'$_{Br}$** (126 MHz, $CDCl_3$, 298 K).

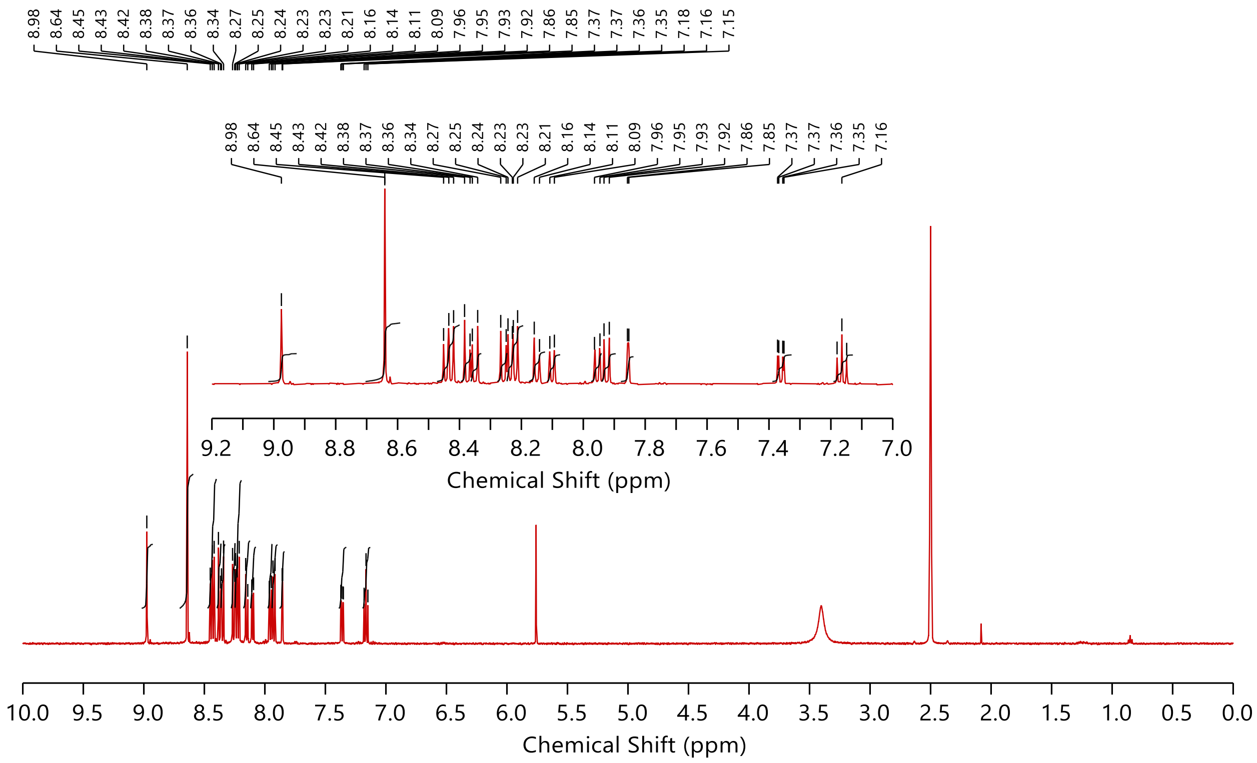

Supplementary Figure 58. ^{1}H NMR spectrum of **O8H'$_{Br}$** (500 MHz, DMSO-d_6, 298 K).

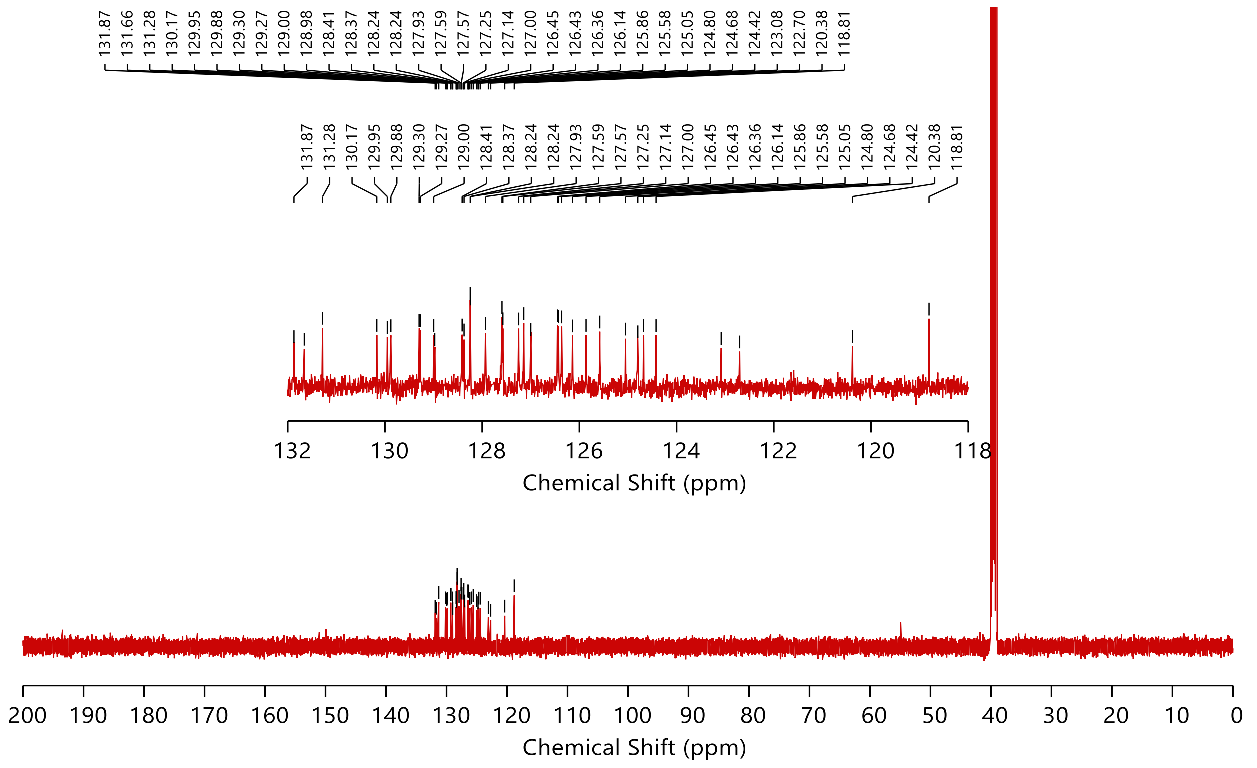

Supplementary Figure 59. $^{13}C\{^{1}H\}$ NMR spectrum of **O8H'$_{Br}$** (126 MHz, DMSO-d_6, 298 K).

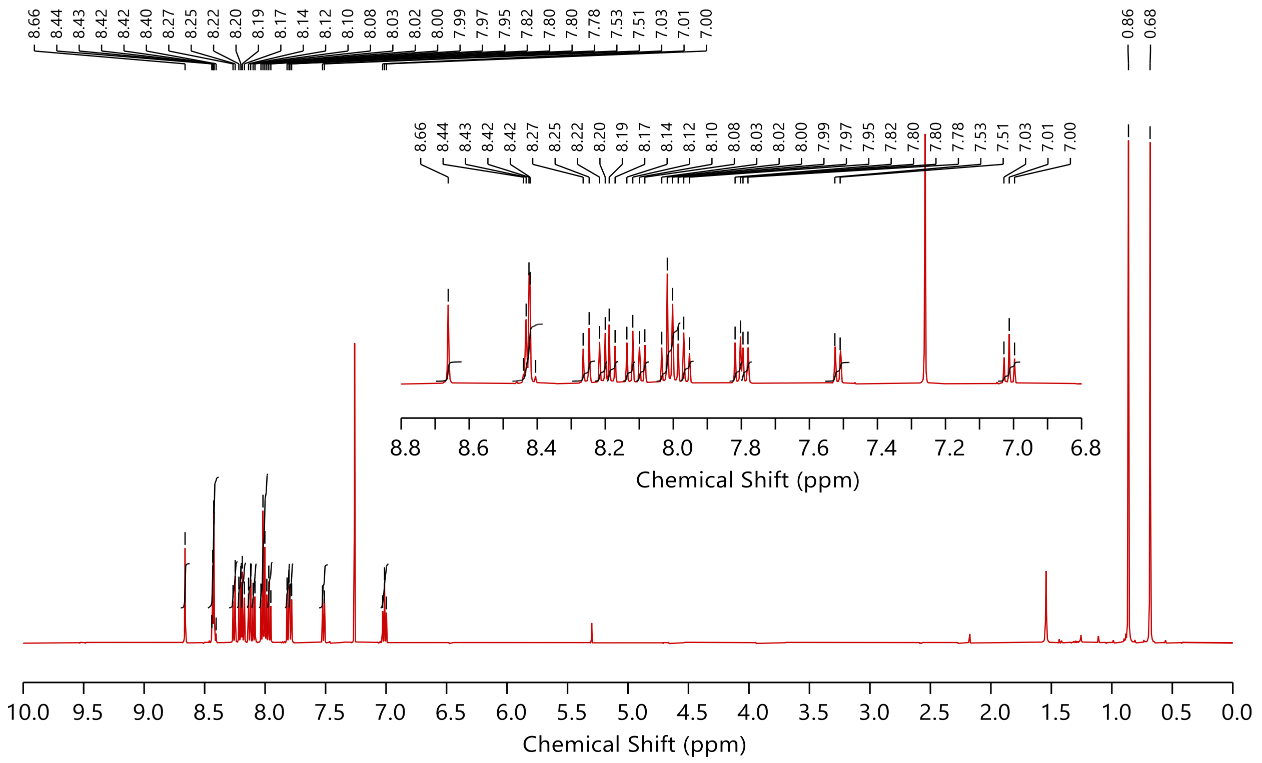

Supplementary Figure 60. ^{1}H NMR spectrum of **O8H'$_{Bpin}$** (500 MHz, $CDCl_3$, 298 K).

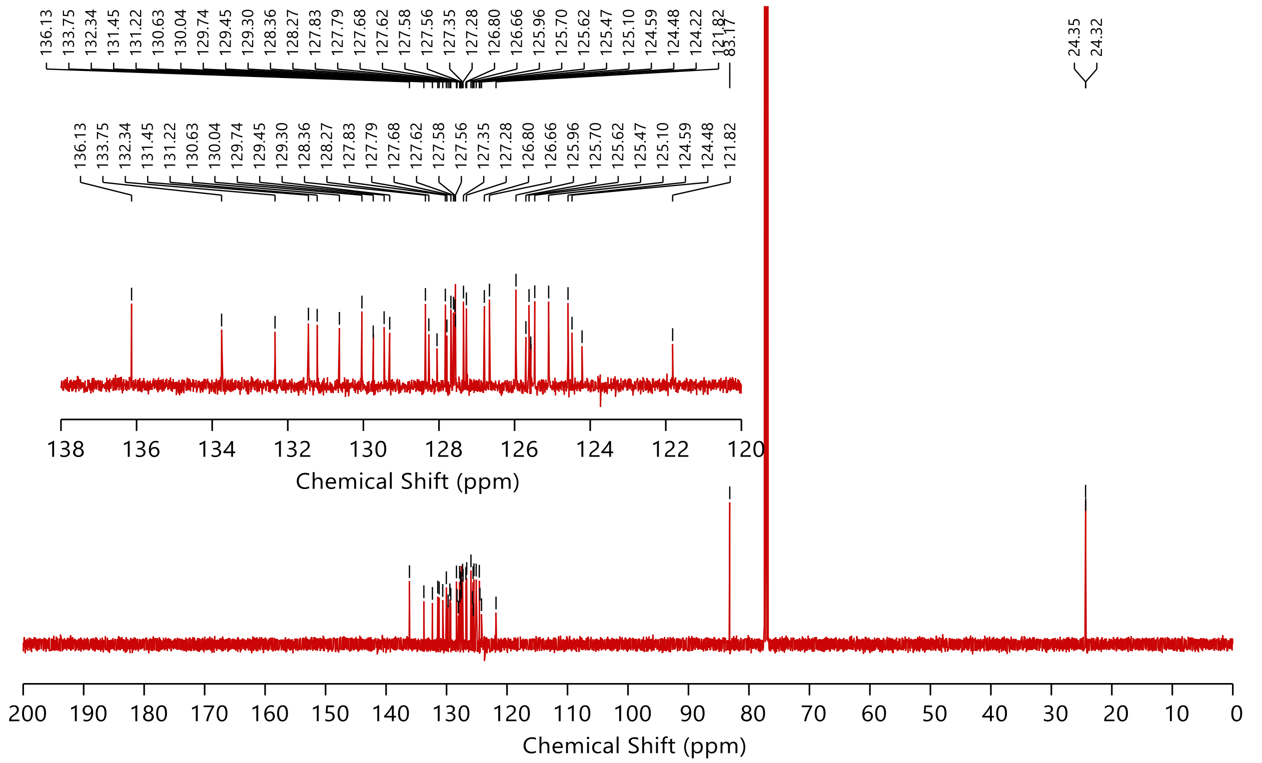

Supplementary Figure 61. $^{13}C\{^{1}H\}$ NMR spectrum of **O8H'$_{Bpin}$** (126 MHz, $CDCl_3$, 298 K).

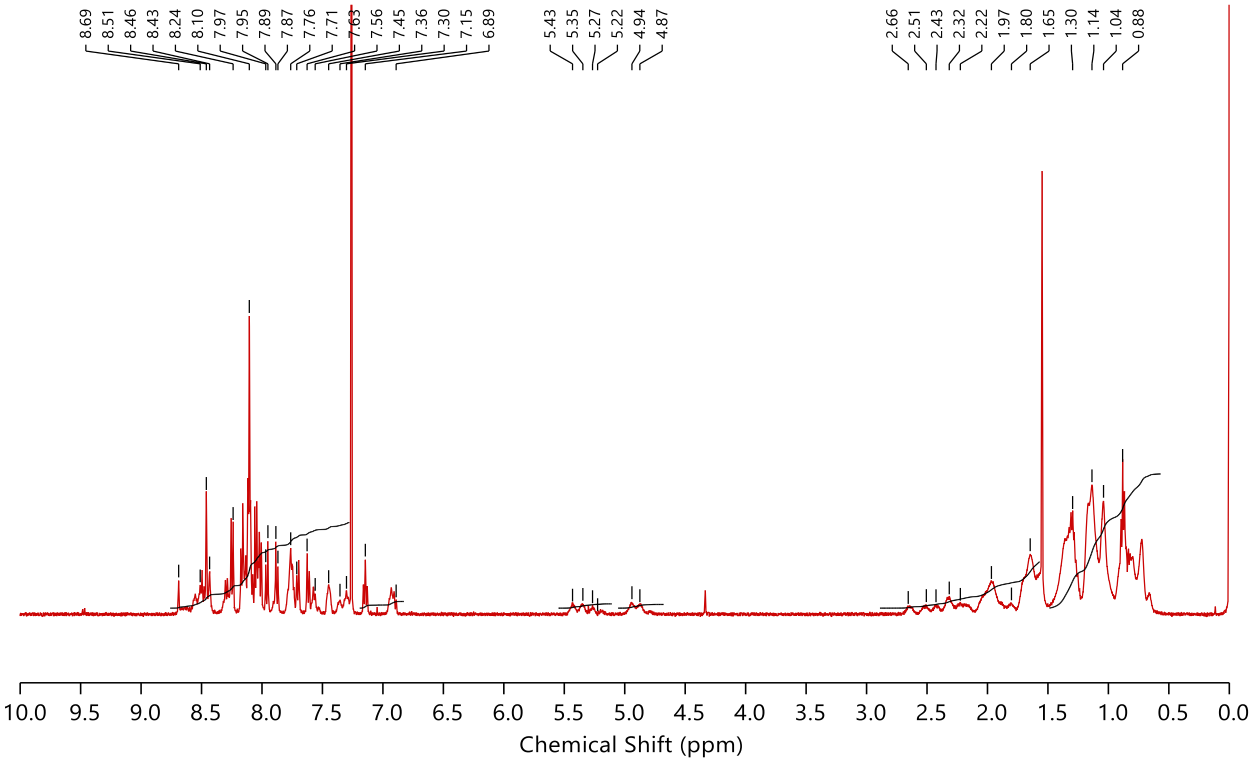

Supplementary Figure 62. ^{1}H NMR spectrum of **O8H'$_{PDI}$** (500 MHz, $CDCl_3$, 298 K).

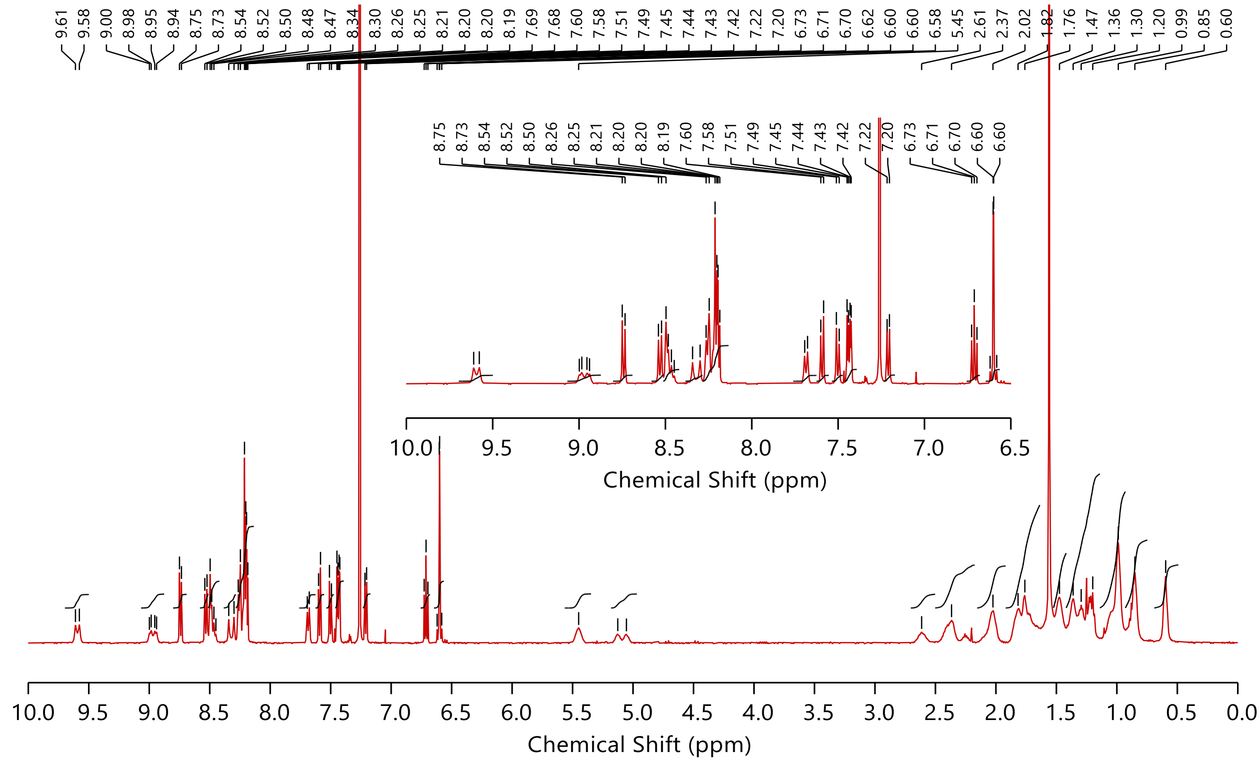

Supplementary Figure 63. ^{1}H NMR spectrum of **CO8H'$_{PDI}$** (500 MHz, $CDCl_3$, 298 K).

1.7 Crystal Structures

Supplementary Table 3. Crystallographic data and structure refinement for **9H**·CH_2Cl_2.

Empirical formula	$C_{39}H_{24}Cl_2$
Formula weight	563.53
Temperature (K)	150.01
Crystal system	Monoclinic
Space group	$P2_1/c$
a (Å)	11.4982(6)
b (Å)	13.2325(7)
c (Å)	18.3650(9)
α (°)	90
β (°)	102.256(2)
γ (°)	90
Volume (Å^3)	2730.5(2)
Z	4
$\rho_{calc.}$ (g·cm^{-3})	1.3707
μ (mm^{-1})	0.267
F(000)	1169.6
Crystal size (mm^3)	0.05 × 0.02 × 0.01
Radiation	Mo K_α (λ = 0.71073)
2Θ range for data collection (°)	4.76 to 52.82
Index ranges	$-14 \leq h \leq 14$, $-16 \leq k \leq 16$, $-22 \leq l \leq 22$
Reflections collected	72610
Independent reflection	5609 [R_{int} = 0.0953, R_σ = 0.0351]
Data/restraints/parameters	5609/0/370
Goodness-of-fit on F^2	1.148
Final R indexes [$I \geq 2\sigma(I)$]	R_1 = 0.0907, wR_2 = 0.2496
Final R indexes [all data]	R_1 = 0.1281, wR_2 = 0.3097
Largest diff. peak/hole (e·Å^{-3})	0.80/−1.38

Supplementary Table 4. Crystallographic data and structure refinement for **8H$_{CF3}$**.

Empirical formula	$C_{35}H_{19}F_3$
Formula weight	496.536
Temperature (K)	172.99
Crystal system	Triclinic
Space group	$P\bar{1}$
a (Å)	9.8273(3)
b (Å)	10.3670(3)
c (Å)	25.7500(7)
α (°)	93.595(1)
β (°)	92.245(1)
γ (°)	113.800(1)
Volume (Å^3)	2389.60(12)
Z	4
$\rho_{calc.}$ (g·cm^{-3})	1.380
μ (mm^{-1})	0.779
F(000)	1027.5
Crystal size (mm^3)	0.1 × 0.1 × 0.1
Radiation	Cu K$_\alpha$ (λ = 1.54178)
2Θ range for data collection (°)	6.9 to 136.5
Index ranges	$-11 \leq h \leq 11$, $-12 \leq k \leq 12$, $-29 \leq l \leq 30$
Reflections collected	29713
Independent reflection	8724 [R_{int} = 0.0434, R_σ = 0.0378]
Data/restraints/parameters	8724/0/722
Goodness-of-fit on F^2	1.088
Final R indexes [$I \geq 2\sigma(I)$]	R_1 = 0.0579, wR_2 = 0.1277
Final R indexes [all data]	R_1 = 0.0762, wR_2 = 0.1388
Largest diff. peak/hole (e·Å^{-3})	0.43/−0.28

Supplementary Table 5. Crystallographic data and structure refinement for **8H$_F$**.

Empirical formula	$C_{34}H_{19}F$
Formula weight	446.528
Temperature (K)	297.32
Crystal system	Monoclinic
Space group	$P2_1/c$
a (Å)	15.1263(18)
b (Å)	9.7528(10)
c (Å)	32.469(5)
α (°)	90
β (°)	111.694(12)
γ (°)	90
Volume (Å^3)	4450.7(11)
Z	8
$\rho_{calc.}$ (g·cm^{-3})	1.333
μ (mm^{-1})	0.641
F(000)	1861.6
Crystal size (mm^3)	0.1 × 0.1 × 0.1
Radiation	Cu K$_\alpha$ (λ = 1.54178)
2Θ range for data collection (°)	5.86 to 136.74
Index ranges	$-17 \leq h \leq 18$, $-10 \leq k \leq 11$, $-39 \leq l \leq 39$
Reflections collected	28191
Independent reflection	8071 [R_{int} = 0.0433, R_σ = 0.0339]
Data/restraints/parameters	8071/4/641
Goodness-of-fit on F^2	1.079
Final R indexes [$I \geq 2\sigma(I)$]	R_1 = 0.0546, wR_2 = 0.1328
Final R indexes [all data]	R_1 = 0.0727, wR_2 = 0.1460
Largest diff. peak/hole (e·Å^{-3})	0.50/−0.26

Supplementary Table 6. Crystallographic data and structure refinement for **7H**.

Empirical formula	$C_{30}H_{18}$
Formula weight	378.477
Temperature (K)	150.01
Crystal system	Monoclinic
Space group	$P2_1$
a (Å)	9.2826(5)
b (Å)	14.9039(7)
c (Å)	13.9050(8)
α (°)	90
β (°)	92.972(2)
γ (°)	90
Volume (Å^3)	1921.13(18)
Z	4
$\rho_{calc.}$ (g·cm^{-3})	1.309
μ (mm^{-1})	0.074
F(000)	792.3
Crystal size (mm^3)	0.03 × 0.03 × 0.01
Radiation	Mo K_α (λ = 0.71073)
2Θ range for data collection (°)	4.4 to 53.12
Index ranges	$-11 \leq h \leq 11, -18 \leq k \leq 18, -17 \leq l \leq 17$
Reflections collected	42191
Independent reflection	7870 [R_{int} = 0.0428, R_σ = 0.0300]
Data/restraints/parameters	7870/1/541
Goodness-of-fit on F^2	1.040
Final R indexes [$I \geq 2\sigma(I)$]	R_1 = 0.0604, wR_2 = 0.1619
Final R indexes [all data]	R_1 = 0.0642, wR_2 = 0.1644
Largest diff. peak/hole (e·Å^{-3})	0.32/−0.28
Flack parameter	−2(11) [a]

[a] Large standard uncertainty can be ascribed to the weak diffraction and the vacancy of heavy atoms.

Supplementary Table 7. Crystallographic data and structure refinement for **O7H**.

Empirical formula	$C_{30}H_{16}$
Formula weight	376.461
Temperature (K)	172.98
Crystal system	Monoclinic
Space group	$P2_1/c$
a (Å)	16.2553(2)
b (Å)	5.8139(1)
c (Å)	20.2242(3)
α (°)	90
β (°)	110.7525(5)
γ (°)	90
Volume (Å^3)	1787.32(5)
Z	4
$\rho_{calc.}$ (g·cm^{-3})	1.399
μ (mm^{-1})	0.606
F(000)	786.2
Crystal size (mm^3)	0.1 × 0.1 × 0.1
Radiation	Cu K_α (λ = 1.54178)
2Θ range for data collection (°)	5.82 to 136.5
Index ranges	$-19 \leq h \leq 19$, $-6 \leq k \leq 6$, $-24 \leq l \leq 24$
Reflections collected	17139
Independent reflection	3242 [R_{int} = 0.0263, R_σ = 0.0190]
Data/restraints/parameters	3242/0/271
Goodness-of-fit on F^2	1.067
Final R indexes [$I \geq 2\sigma(I)$]	R_1 = 0.0337, wR_2 = 0.0844
Final R indexes [all data]	R_1 = 0.0380, wR_2 = 0.0888
Largest diff. peak/hole (e·Å^{-3})	0.15/−0.14

Supplementary Table 8. Crystallographic data and structure refinement for **O8H**.

Empirical formula	$C_{34}H_{18}$
Formula weight	426.522
Temperature (K)	173.0
Crystal system	Monoclinic
Space group	$P2_1/c$
a (Å)	11.0500(16)
b (Å)	8.6712(6)
c (Å)	21.654(3)
α (°)	90
β (°)	100.803(9)
γ (°)	90
Volume (Å^3)	2038.0(4)
Z	4
$\rho_{calc.}$ (g·cm^{-3})	1.390
μ (mm^{-1})	0.602
F(000)	890.5
Crystal size (mm^3)	0.05 × 0.04 × 0.02
Radiation	Cu K$_\alpha$ (λ = 1.54178)
2Θ range for data collection (°)	8.32 to 136.76
Index ranges	$-13 \leq h \leq 13$, $-10 \leq k \leq 10$, $-25 \leq l \leq 21$
Reflections collected	29555
Independent reflection	3706 [R_{int} = 0.0278, R_σ = 0.0158]
Data/restraints/parameters	3706/0/307
Goodness-of-fit on F^2	1.053
Final R indexes [$I \geq 2\sigma(I)$]	R_1 = 0.0363, wR_2 = 0.0947
Final R indexes [all data]	R_1 = 0.0382, wR_2 = 0.0965
Largest diff. peak/hole (e·Å^{-3})	0.17/−0.16

Supplementary Table 9. Crystallographic data and structure refinement for **O9H**.

Empirical formula	$C_{38}H_{20}$
Formula weight	476.582
Temperature (K)	173.0
Crystal system	Monoclinic
Space group	$P2_1/c$
a (Å)	9.6847(5)
b (Å)	15.6667(7)
c (Å)	15.5544(7)
α (°)	90
β (°)	90.666(3)
γ (°)	90
Volume (Å^3)	2359.87(19)
Z	4
$\rho_{calc.}$ (g·cm^{-3})	1.341
μ (mm^{-1})	0.581
F(000)	994.8
Crystal size (mm^3)	0.05 × 0.05 × 0.02
Radiation	Cu K$_\alpha$ (λ = 1.54178)
2Θ range for data collection (°)	8 to 137.02
Index ranges	$-11 \le h \le 11$, $-18 \le k \le 18$, $-18 \le l \le 18$
Reflections collected	17251
Independent reflection	4295 [R_{int} = 0.0720, R_σ = 0.0646]
Data/restraints/parameters	4295/0/343
Goodness-of-fit on F^2	1.070
Final R indexes [$I \ge 2\sigma(I)$]	R_1 = 0.1205, wR_2 = 0.3399
Final R indexes [all data]	R_1 = 0.1529, wR_2 = 0.3719
Largest diff. peak/hole (e·Å^{-3})	0.40/−0.44

Note: Due to the high difficulty to grow qualified crystals, one CheckCIF alert in level B was found as a result of the high wR_2 value.

Supplementary Table 10. Crystallographic data and structure refinement for **O8H$_{CH3}$**.

Empirical formula	$C_{35}H_{20}$
Formula weight	440.549
Temperature (K)	149.99
Crystal system	Monoclinic
Space group	$P2_1/c$
a (Å)	12.3532(6)
b (Å)	8.6104(4)
c (Å)	20.8267(10)
α (°)	90
β (°)	104.742(2)
γ (°)	90
Volume (Å^3)	2142.33(18)
Z	4
$\rho_{calc.}$ (g·cm^{-3})	1.366
μ (mm^{-1})	0.077
F(000)	920.4
Crystal size (mm^3)	0.05 × 0.02 × 0.02
Radiation	Cu K$_\alpha$ (λ = 1.54178)
2Θ range for data collection (°)	4.58 to 54.36
Index ranges	$-15 \le h \le 15$, $-11 \le k \le 11$, $-26 \le l \le 26$
Reflections collected	50135
Independent reflection	4745 [R_{int} = 0.0625, R_σ = 0.0266]
Data/restraints/parameters	4745/0/317
Goodness-of-fit on F^2	1.090
Final R indexes [$I \ge 2\sigma(I)$]	R_1 = 0.0514, wR_2 = 0.1283
Final R indexes [all data]	R_1 = 0.0749, wR_2 = 0.1520
Largest diff. peak/hole (e·Å^{-3})	0.58/−0.33

Supplementary Table 11. Crystallographic data and structure refinement for **O8H$_{CF3}$**.

Empirical formula	$C_{35}H_{17}F_3$
Formula weight	494.520
Temperature (K)	296.92
Crystal system	Monoclinic
Space group	*C*2/*c*
a (Å)	19.355(7)
b (Å)	9.806(4)
c (Å)	25.605(10)
α (°)	90
β (°)	111.300(17)
γ (°)	90
Volume (Å^3)	4528(3)
Z	8
$\rho_{calc.}$ (g·cm^{-3})	1.451
μ (mm^{-1})	0.822
F(000)	2038.9
Crystal size (mm^3)	0.1 × 0.1 × 0.1
Radiation	Cu K_α (λ = 1.54178)
2Θ range for data collection (°)	11.58 to 127.36
Index ranges	$-22 \leq h \leq 22$, $-11 \leq k \leq 11$, $-29 \leq l \leq 29$
Reflections collected	17518
Independent reflection	3544 [R_{int} = 0.0976, R_σ = 0.0823]
Data/restraints/parameters	3544/1/343
Goodness-of-fit on F^2	1.114
Final *R* indexes [$I \geq 2\sigma(I)$]	R_1 = 0.0604, wR_2 = 0.1906
Final *R* indexes [all data]	R_1 = 0.1516, wR_2 = 0.2725
Largest diff. peak/hole (e·Å^{-3})	0.50/−0.50

Note: Due to the high difficulty to grow qualified crystals, one CheckCIF alert in level B was found as a result of the low Θ value.

Supplementary Table 12. Crystallographic data and structure refinement for the mixed crystal of **O8H$_F$-α** and **O8H$_F$-β** (at a ratio of 87:13).

Empirical formula	$C_{34}H_{17}F$
Formula weight	444.512
Temperature (K)	173.0
Crystal system	Monoclinic
Space group	$P2_1/c$
a (Å)	22.8654(8)
b (Å)	8.4179(3)
c (Å)	22.3166(7)
α (°)	90
β (°)	102.3557(16)
γ (°)	90
Volume (Å^3)	4196.0(3)
Z	8
$\rho_{calc.}$ (g·cm^{-3})	1.407
μ (mm^{-1})	0.680
F(000)	1845.6
Crystal size (mm^3)	0.1 × 0.1 × 0.1
Radiation	Cu K$_\alpha$ (λ = 1.54178)
2Θ range for data collection (°)	8.12 to 136.88
Index ranges	$-27 \le h \le 26, 0 \le k \le 10, 0 \le l \le 26$
Reflections collected	7622
Independent reflection	7622 [R_{int} = N/A, R_σ = N/A]
Data/restraints/parameters	7622/0/652
Goodness-of-fit on F^2	1.062
Final R indexes [$I \ge 2\sigma(I)$]	R_1 = 0.0674, wR_2 = 0.1850
Final R indexes [all data]	R_1 = 0.0819, wR_2 = 0.1943
Largest diff. peak/hole (e·Å^{-3})	0.34/−0.37

Note: Twinning was found in the crystal and this has been addressed in the refinement using Olex2.

Supplementary Table 13. Crystallographic data and structure refinement for the mixed crystal of **O8H$_{Br}$-α** and **O8H$_{Br}$-β** (at a ratio of 90:10).

Empirical formula	$C_{34}H_{17}Br$
Formula weight	505.417
Temperature (K)	297.45
Crystal system	Monoclinic
Space group	$P2_1/c$
a (Å)	12.499(2)
b (Å)	8.4972(14)
c (Å)	21.513(3)
α (°)	90
β (°)	103.562(13)
γ (°)	90
Volume (Å^3)	2221.1(7)
Z	4
$\rho_{calc.}$ (g·cm^{-3})	1.511
μ (mm^{-1})	2.678
F(000)	1023.9
Crystal size (mm^3)	0.1 × 0.1 × 0.1
Radiation	Cu K$_\alpha$ (λ = 1.54178)
2Θ range for data collection (°)	7.28 to 137.02
Index ranges	$-14 \leq h \leq 15$, $-9 \leq k \leq 10$, $-25 \leq l \leq 20$
Reflections collected	15985
Independent reflection	4064 [R_{int} = 0.0460, R_σ = 0.0366]
Data/restraints/parameters	4064/0/326
Goodness-of-fit on F^2	1.059
Final R indexes [$I \geq 2\sigma(I)$]	R_1 = 0.0639, wR_2 = 0.1730
Final R indexes [all data]	R_1 = 0.0816, wR_2 = 0.1902
Largest diff. peak/hole (e·Å^{-3})	0.96/−0.98

Supplementary Table 14. Crystallographic data and structure refinement for **2O8H$_{2py}$**·CH_2Cl_2.

Empirical formula	$C_{93}H_{54}Cl_2$
Formula weight	1242.37
Temperature (K)	150.0
Crystal system	Triclinic
Space group	$P\bar{1}$
a (Å)	11.4726(5)
b (Å)	16.9881(7)
c (Å)	17.1711(7)
α (°)	104.422(2)
β (°)	104.691(2)
γ (°)	96.160(2)
Volume (Å^3)	3082.8(2)
Z	2
$\rho_{calc.}$ (g·cm^{-3})	1.3383
μ (mm^{-1})	0.160
F(000)	1293.1
Crystal size (mm^3)	0.3 × 0.3 × 0.25
Radiation	Mo K$_\alpha$ (λ = 0.71073)
2Θ range for data collection (°)	4.58 to 52.98
Index ranges	$-14 \le h \le 14$, $-21 \le k \le 21$, $-21 \le l \le 21$
Reflections collected	52253
Independent reflection	12691 [R_{int} = 0.0601, R_σ = 0.0476]
Data/restraints/parameters	12691/0/856
Goodness-of-fit on F^2	1.089
Final R indexes [$I \ge 2\sigma(I)$]	R_1 = 0.0551, wR_2 = 0.1316
Final R indexes [all data]	R_1 = 0.0984, wR_2 = 0.1670
Largest diff. peak/hole (e·Å^{-3})	0.57/−0.73

Supplementary Table 15. Crystallographic data and structure refinement for **OO7H**.

Empirical formula	$C_{30}H_{14}$
Formula weight	374.445
Temperature (K)	173.0
Crystal system	Monoclinic
Space group	$P2_1/c$
a (Å)	18.4652(6)
b (Å)	4.6554(1)
c (Å)	19.6479(6)
α (°)	90
β (°)	93.7066(17)
γ (°)	90
Volume (Å^3)	1685.46(8)
Z	4
$\rho_{calc.}$ (g·cm^{-3})	1.476
μ (mm^{-1})	0.642
F(000)	778.2
Crystal size (mm^3)	0.1 × 0.1 × 0.05
Radiation	Cu K_α (λ = 1.54178)
2Θ range for data collection (°)	4.8 to 136.42
Index ranges	$-22 \leq h \leq 21$, $-5 \leq k \leq 5$, $-23 \leq l \leq 20$
Reflections collected	20975
Independent reflection	3089 [R_{int} = 0.0963, R_σ = 0.0469]
Data/restraints/parameters	3089/0/271
Goodness-of-fit on F^2	1.069
Final R indexes [$I \geq 2\sigma(I)$]	R_1 = 0.0758, wR_2 = 0.2186
Final R indexes [all data]	R_1 = 0.1349, wR_2 = 0.2709
Largest diff. peak/hole (e·Å^{-3})	0.51/−0.36

Supplementary Table 16. Crystallographic data and structure refinement for **OO8H**.

Empirical formula	$C_{34}H_{16}$
Formula weight	424.506
Temperature (K)	173.0
Crystal system	Orthorhombic
Space group	$P2_12_12_1$
a (Å)	7.4471(2)
b (Å)	12.5355(4)
c (Å)	21.0318(8)
α (°)	90
β (°)	90
γ (°)	90
Volume (Å^3)	1963.38(11)
Z	4
$\rho_{calc.}$ (g·cm^{-3})	1.436
μ (mm^{-1})	0.625
F(000)	882.5
Crystal size (mm^3)	0.1 × 0.1 × 0.1
Radiation	Cu K$_\alpha$ (λ = 1.54178)
2Θ range for data collection (°)	8.22 to 137.02
Index ranges	$-8 \le h \le 8$, $-15 \le k \le 14$, $-25 \le l \le 25$
Reflections collected	37415
Independent reflection	3598 [R_{int} = 0.1208, R_σ = 0.0458]
Data/restraints/parameters	3598/0/307
Goodness-of-fit on F^2	1.028
Final R indexes [$I \ge 2\sigma(I)$]	R_1 = 0.0660, wR_2 = 0.1754
Final R indexes [all data]	R_1 = 0.0994, wR_2 = 0.2070
Largest diff. peak/hole (e·Å^{-3})	0.22/−0.24
Flack parameter	−1(3) [a]

[a] Large standard uncertainty can be ascribed to the weak diffraction and the vacancy of heavy atoms.

Supplementary Table 17. Crystallographic data and structure refinement for **OO9H**.

Empirical formula	$C_{38}H_{18}$
Formula weight	474.566
Temperature (K)	173.0
Crystal system	Orthorhombic
Space group	$P2_12_12_1$
a (Å)	4.7250(3)
b (Å)	19.3324(13)
c (Å)	23.9090(14)
α (°)	90
β (°)	90
γ (°)	90
Volume (Å^3)	2184.0(2)
Z	4
$\rho_{calc.}$ (g·cm^{-3})	1.443
μ (mm^{-1})	0.628
F(000)	986.8
Crystal size (mm^3)	0.5 × 0.1 × 0.05
Radiation	Cu K$_\alpha$ (λ = 1.54178)
2Θ range for data collection (°)	5.88 to 127.98
Index ranges	$-4 \le h \le 5$, $-22 \le k \le 22$, $-27 \le l \le 27$
Reflections collected	12748
Independent reflection	3582 [R_{int} = 0.1452, R_σ = 0.0952]
Data/restraints/parameters	3582/0/343
Goodness-of-fit on F^2	1.012
Final R indexes [$I \ge 2\sigma(I)$]	R_1 = 0.0744, wR_2 = 0.1821
Final R indexes [all data]	R_1 = 0.1077, wR_2 = 0.2141
Largest diff. peak/hole (e·Å^{-3})	0.30/−0.33
Flack parameter	0(5) [a]

[a] Large standard uncertainty can be ascribed to the weak diffraction and the vacancy of heavy atoms.

Note: Due to the high difficulty to grow qualified crystals, one CheckCIF alert in level B was found as a result of the low Θ value.

Supplementary Table 18. Crystallographic data and structure refinement for **O8H$_{mt}$**.

Empirical formula	$C_{33}H_{18}S$
Formula weight	446.577
Temperature (K)	173.01
Crystal system	Monoclinic
Space group	$P2_1/n$
a (Å)	12.4404(14)
b (Å)	8.8519(9)
c (Å)	19.933(2)
α (°)	90
β (°)	103.479(10)
γ (°)	90
Volume (Å^3)	2134.6(4)
Z	4
$\rho_{calc.}$ (g·cm^{-3})	1.390
μ (mm^{-1})	1.490
F(000)	931.8
Crystal size (mm^3)	0.1 × 0.1 × 0.1
Radiation	Cu K$_\alpha$ (λ = 1.54178)
2Θ range for data collection (°)	7.66 to 136.82
Index ranges	$-14 \leq h \leq 15, -10 \leq k \leq 10, -19 \leq l \leq 24$
Reflections collected	25710
Independent reflection	3908 [R_{int} = 0.0349, R_σ = 0.0200]
Data/restraints/parameters	3908/0/308
Goodness-of-fit on F^2	1.055
Final R indexes [$I \geq 2\sigma(I)$]	R_1 = 0.0355, wR_2 = 0.0901
Final R indexes [all data]	R_1 = 0.0401, wR_2 = 0.0943
Largest diff. peak/hole (e·Å^{-3})	0.19/−0.31

Supplementary Table 19. Crystallographic data and structure refinement for **OO8H$_{mt}$**.

Empirical formula	$C_{33}H_{16}S$
Formula weight	444.561
Temperature (K)	173.0
Crystal system	Monoclinic
Space group	$P2_1/c$
a (Å)	11.866(7)
b (Å)	7.278(5)
c (Å)	25.823(17)
α (°)	90
β (°)	116.28(6)
γ (°)	90
Volume (Å^3)	2000(2)
Z	4
$\rho_{calc.}$ (g·cm^{-3})	1.477
μ (mm^{-1})	1.590
F(000)	923.8
Crystal size (mm^3)	0.1 × 0.1 × 01
Radiation	Cu K$_\alpha$ (λ = 1.54178)
2Θ range for data collection (°)	7.64 to 122.72
Index ranges	$-13 \le h \le 13$, $-8 \le k \le 8$, $-28 \le l \le 29$
Reflections collected	9251
Independent reflection	3060 [R_{int} = 0.0688, R_σ = 0.0668]
Data/restraints/parameters	3060/0/308
Goodness-of-fit on F^2	1.042
Final R indexes [$I \geq 2\sigma(I)$]	R_1 = 0.0849, wR_2 = 0.2267
Final R indexes [all data]	R_1 = 0.1112, wR_2 = 0.2630
Largest diff. peak/hole (e·Å^{-3})	0.48/−0.40

Note: Due to the high difficulty to grow qualified crystals, one CheckCIF alert in level A and two in level B were found as a result of the low Θ value and high uncertainty of the β angle.

Supplementary Table 20. Crystallographic data and structure refinement for **O8H$_a$**.

Empirical formula	$C_{33}H_{17}N$
Formula weight	427.509
Temperature (K)	297.37
Crystal system	Triclinic
Space group	$P\bar{1}$
a (Å)	8.8314(1)
b (Å)	14.1848(2)
c (Å)	16.9692(2)
α (°)	87.3360(6)
β (°)	84.0065(6)
γ (°)	76.5503(6)
Volume (Å^3)	2055.62(5)
Z	4
$\rho_{calc.}$ (g·cm^{-3})	1.381
μ (mm^{-1})	0.613
F(000)	890.5
Crystal size (mm^3)	0.1 × 0.1 × 0.1
Radiation	Cu K$_\alpha$ (λ = 1.54178)
2Θ range for data collection (°)	5.24 to 136.44
Index ranges	$-10 \le h \le 10$, $-17 \le k \le 17$, $-20 \le l \le 20$
Reflections collected	37835
Independent reflection	7478 [R_{int} = 0.0468, R_σ = 0.0374]
Data/restraints/parameters	7478/0/613
Goodness-of-fit on F^2	1.071
Final R indexes [$I \ge 2\sigma(I)$]	R_1 = 0.0422, wR_2 = 0.1080
Final R indexes [all data]	R_1 = 0.0786, wR_2 = 0.1257
Largest diff. peak/hole (e·Å^{-3})	0.26/−0.33

Supplementary Table 21. Crystallographic data and structure refinement for **O8H$_{ta}$**.

Empirical formula	$C_{31}H_{15}NS$
Formula weight	433.537
Temperature (K)	172.98
Crystal system	Monoclinic
Space group	$P2_1/c$
a (Å)	9.6926(8)
b (Å)	18.059(2)
c (Å)	11.9231(12)
α (°)	90
β (°)	110.242(6)
γ (°)	90
Volume (Å^3)	1958.1(4)
Z	4
$\rho_{calc.}$ (g·cm^{-3})	1.471
μ (mm^{-1})	1.622
F(000)	899.8
Crystal size (mm^3)	0.05 × 0.05 × 0.05
Radiation	Cu K$_\alpha$ (λ = 1.54178)
2Θ range for data collection (°)	9.3 to 136.6
Index ranges	$-11 \leq h \leq 11$, $-21 \leq k \leq 21$, $-14 \leq l \leq 14$
Reflections collected	27944
Independent reflection	3577 [R_{int} = 0.0286, R_σ = 0.0153]
Data/restraints/parameters	3577/0/298
Goodness-of-fit on F^2	1.055
Final R indexes [$I \geq 2\sigma(I)$]	R_1 = 0.0313, wR_2 = 0.0803
Final R indexes [all data]	R_1 = 0.0339, wR_2 = 0.0827
Largest diff. peak/hole (e·Å^{-3})	0.24/−0.36

Supplementary Table 22. Crystallographic data and structure refinement for **O6H$_{\mathbf{DPT}}$**·1/2hexane.

Empirical formula	$C_{59}H_{35}$
Formula weight	743.935
Temperature (K)	297.3
Crystal system	Triclinic
Space group	$P\bar{1}$
a (Å)	12.1965(14)
b (Å)	12.896(2)
c (Å)	13.1874(14)
α (°)	81.517(8)
β (°)	82.012(6)
γ (°)	62.031(5)
Volume (Å^3)	1806.1(4)
Z	2
$\rho_{calc.}$ (g·cm^{-3})	1.368
μ (mm^{-1})	0.590
F(000)	780.2
Crystal size (mm^3)	0.1 × 0.1 × 0.1
Radiation	Cu K$_\alpha$ (λ = 1.54178)
2Θ range for data collection (°)	6.8 to 130.58
Index ranges	$-14 \leq h \leq 13$, $-15 \leq k \leq 15$, $-15 \leq l \leq 15$
Reflections collected	25199
Independent reflection	6189 [R_{int} = 0.0880, R_σ = 0.0659]
Data/restraints/parameters	6189/0/533
Goodness-of-fit on F^2	1.046
Final R indexes [$I \geq 2\sigma(I)$]	R_1 = 0.0769, wR_2 = 0.2035
Final R indexes [all data]	R_1 = 0.1396, wR_2 = 0.2615
Largest diff. peak/hole (e·Å^{-3})	0.31/−0.39

Supplementary Table 23. Crystallographic data and structure refinement for **O8H'$_{Br}$**.

Empirical formula	$C_{34}H_{17}Br$
Formula weight	505.417
Temperature (K)	173.0
Crystal system	Monoclinic
Space group	$P2_1/c$
a (Å)	14.7842(3)
b (Å)	11.5002(2)
c (Å)	13.8126(3)
α (°)	90
β (°)	110.842(1)
γ (°)	90
Volume (Å^3)	2194.77(8)
Z	4
$\rho_{calc.}$ (g·cm^{-3})	1.530
μ (mm^{-1})	2.710
F(000)	1023.9
Crystal size (mm^3)	0.1 × 0.1 × 0.02
Radiation	Cu K$_\alpha$ (λ = 1.54178)
2Θ range for data collection (°)	6.4 to 136.78
Index ranges	$-17 \le h \le 17$, $-13 \le k \le 13$, $-15 \le l \le 16$
Reflections collected	22574
Independent reflection	4020 [R_{int} = 0.0273, R_σ = 0.0183]
Data/restraints/parameters	4020/0/316
Goodness-of-fit on F^2	1.052
Final R indexes [$I \ge 2\sigma(I)$]	R_1 = 0.0388, wR_2 = 0.1103
Final R indexes [all data]	R_1 = 0.0411, wR_2 = 0.1131
Largest diff. peak/hole (e·Å^{-3})	0.42/−0.76

Supplementary Table 24. Crystallographic data and structure refinement for **O8H'$_{Bpin}$**.

Empirical formula	$C_{40}H_{29}BO_2$
Formula weight	552.499
Temperature (K)	172.99
Crystal system	Monoclinic
Space group	$P2_1/c$
a (Å)	15.855(3)
b (Å)	11.5894(18)
c (Å)	16.312(4)
α (°)	90
β (°)	101.197(11)
γ (°)	90
Volume (Å^3)	2940.1(10)
Z	4
$\rho_{calc.}$ (g·cm^{-3})	1.248
μ (mm^{-1})	0.580
F(000)	1163.4
Crystal size (mm^3)	0.1 × 0.1 × 0.1
Radiation	Cu K$_\alpha$ (λ = 1.54178)
2Θ range for data collection (°)	5.68 to 136.26
Index ranges	$-19 \leq h \leq 15$, $-13 \leq k \leq 13$, $-19 \leq l \leq 19$
Reflections collected	19697
Independent reflection	5324 [R_{int} = 0.0412, R_σ = 0.0351]
Data/restraints/parameters	5324/0/439
Goodness-of-fit on F^2	1.065
Final R indexes [$I \geq 2\sigma(I)$]	R_1 = 0.0488, wR_2 = 0.1096
Final R indexes [all data]	R_1 = 0.0686, wR_2 = 0.1247
Largest diff. peak/hole (e·Å^{-3})	0.21/−0.34

Supplementary Table 25. Crystallographic data and structure refinement for **CO8H'$_{DPT}$**·$CHCl_3$.

Empirical formula	$C_{81}Cl_3H_{69}N_2O_4$
Formula weight	1240.819
Temperature (K)	172.99
Crystal system	Monoclinic
Space group	$P2_1/c$
a (Å)	17.7544(5)
b (Å)	24.3910(6)
c (Å)	14.2516(4)
α (°)	90
β (°)	91.735(2)
γ (°)	90
Volume (Å^3)	6168.8(3)
Z	4
$\rho_{calc.}$ (g·cm^{-3})	1.336
μ (mm^{-1})	1.790
F(000)	2619.6
Crystal size (mm^3)	0.1 × 0.1 × 0.1
Radiation	Cu K$_\alpha$ (λ = 1.54178)
2Θ range for data collection (°)	6.16 to 127.44
Index ranges	$-20 \leq h \leq 20, -28 \leq k \leq 28, -16 \leq l \leq 16$
Reflections collected	54238
Independent reflection	10149 [R_{int} = 0.0783, R_σ = 0.0450]
Data/restraints/parameters	10149/32/800
Goodness-of-fit on F^2	1.056
Final R indexes [$I \geq 2\sigma(I)$]	R_1 = 0.0764, wR_2 = 0.2188
Final R indexes [all data]	R_1 = 0.1143, wR_2 = 0.2622
Largest diff. peak/hole (e·Å^{-3})	0.73/−0.44

Note: In each asymmetric unit, the possible existing $CHCl_3$ molecule in a disordered fashion was removed by solvent mask using Olex2.

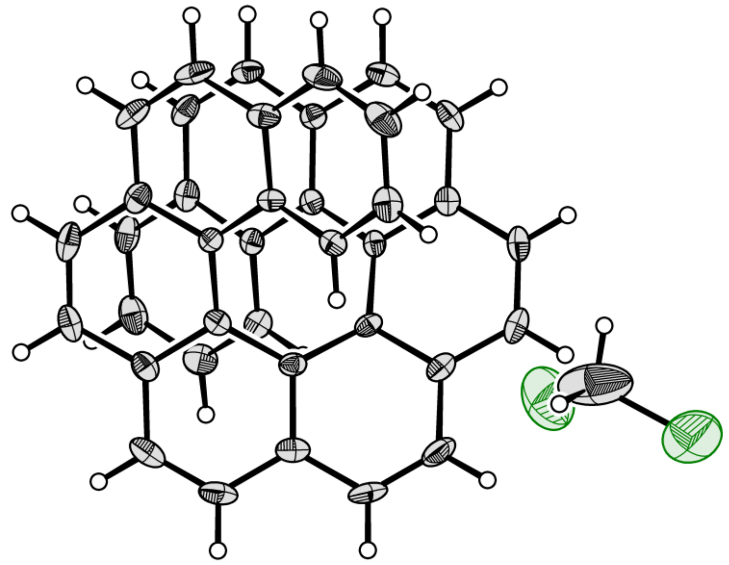

Supplementary Figure 64. X-ray crystallographic structure of racemic **9H**·CH_2Cl_2 (only *M* enantiomer is shown). Thermal ellipsoids are shown at a 50% probability level.

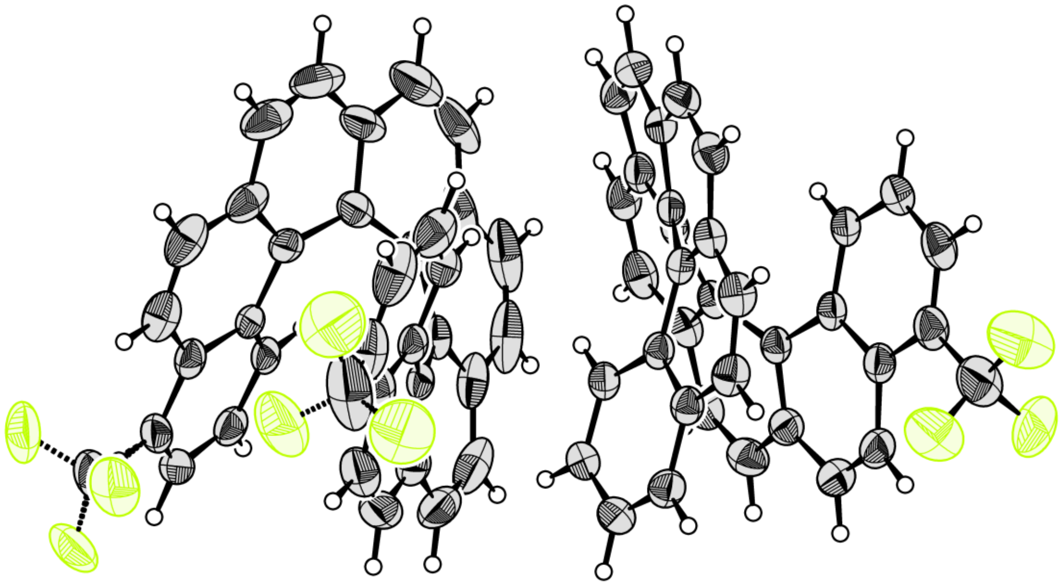

Supplementary Figure 65. X-ray crystallographic structure of racemic **8H**$_{\mathbf{CF3}}$ (only *M* enantiomers are shown). Orientational disorder was found in the crystal. Thermal ellipsoids are shown at a 50% probability level.

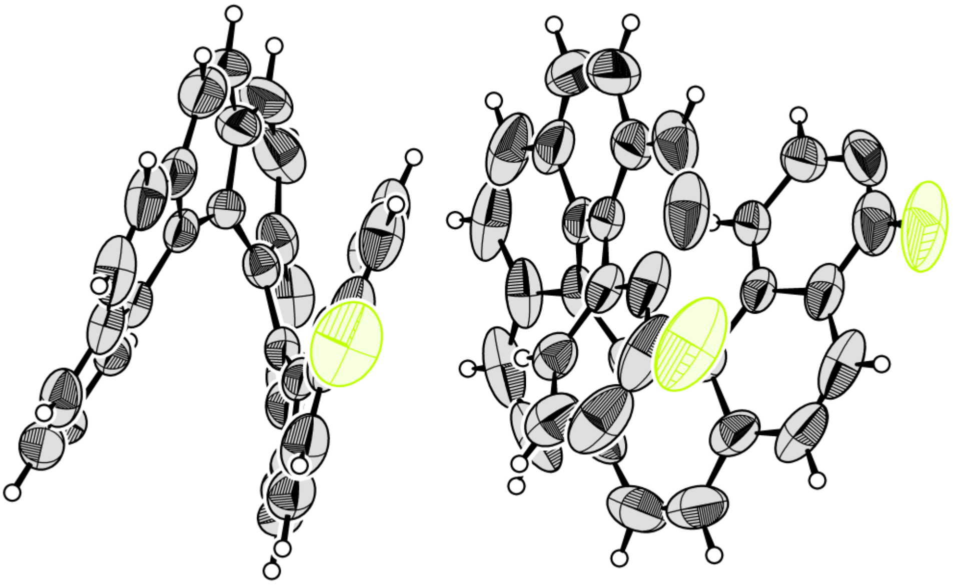

Supplementary Figure 66. X-ray crystallographic structure of racemic **8H**$_{\mathbf{F}}$ (only *M* enantiomer is shown). Orientational disorder was found in the crystal. Thermal ellipsoids are shown at a 50% probability level.

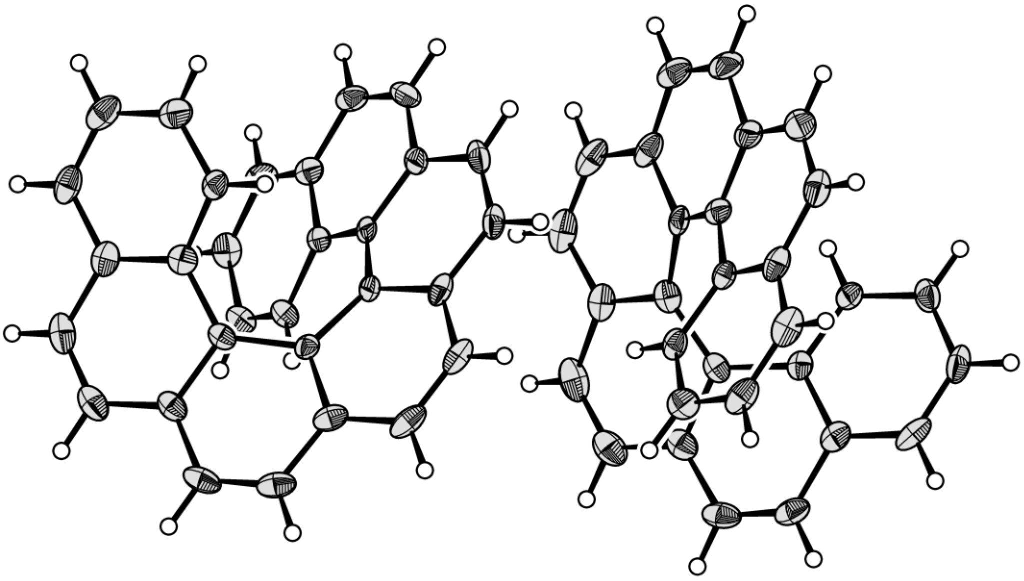

Supplementary Figure 67. X-ray crystallographic structure of **7H**. Thermal ellipsoids are shown at a 50% probability level.

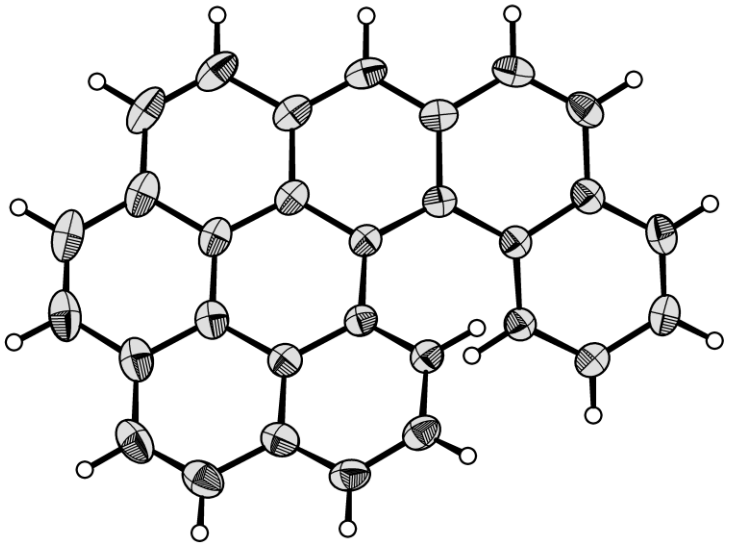

Supplementary Figure 68. X-ray crystallographic structure of racemic **O7H** (only *P* enantiomer is shown). Thermal ellipsoids are shown at a 50% probability level.

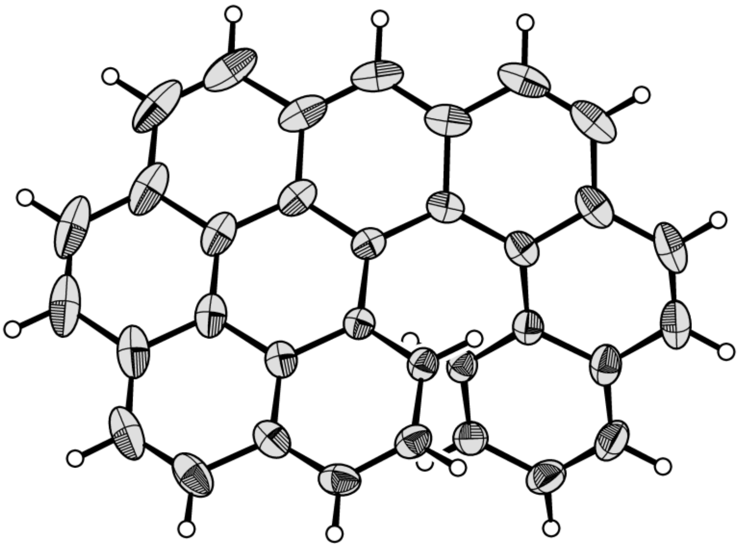

Supplementary Figure 69. X-ray crystallographic structure of racemic **O8H** (only *P* enantiomer is shown). Thermal ellipsoids are shown at the 50% probability level.

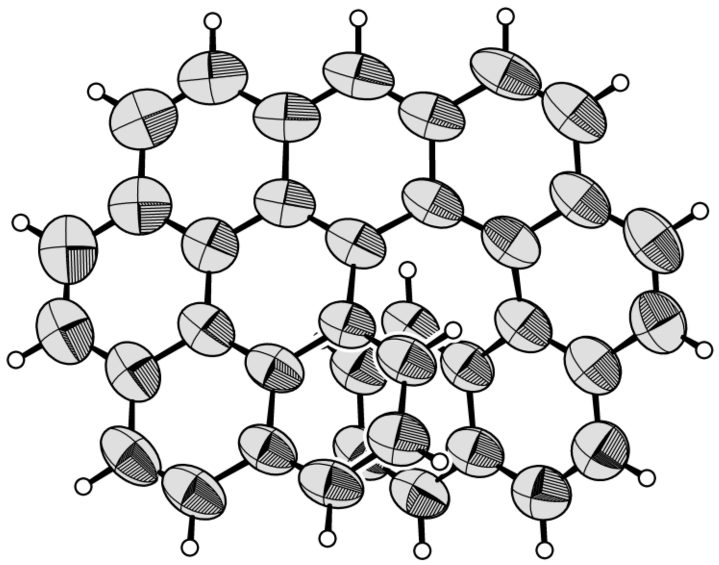

Supplementary Figure 70. X-ray crystallographic structure of racemic **O9H** (only *P* enantiomer is shown). Thermal ellipsoids are shown at a 50% probability level.

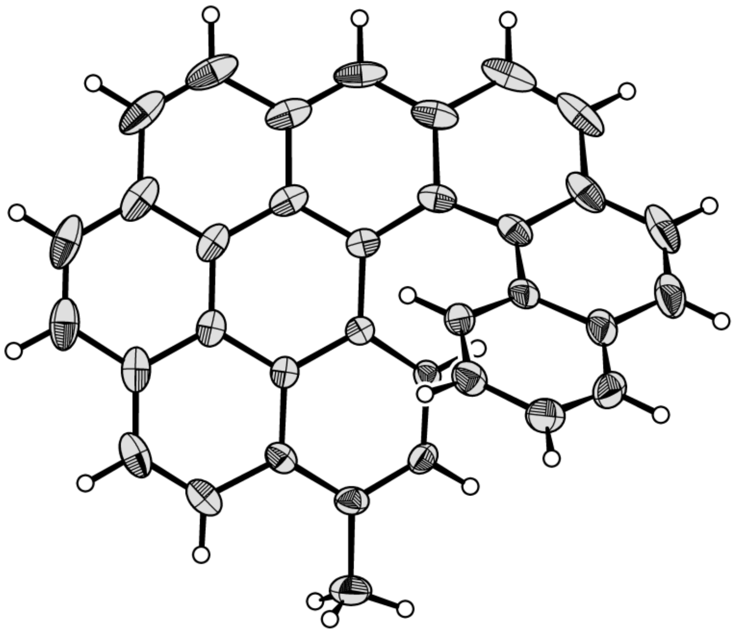

Supplementary Figure 71. X-ray crystallographic structure of racemic **O8H$_{CH3}$** (only *M* enantiomer is shown). Thermal ellipsoids are shown at a 50% probability level.

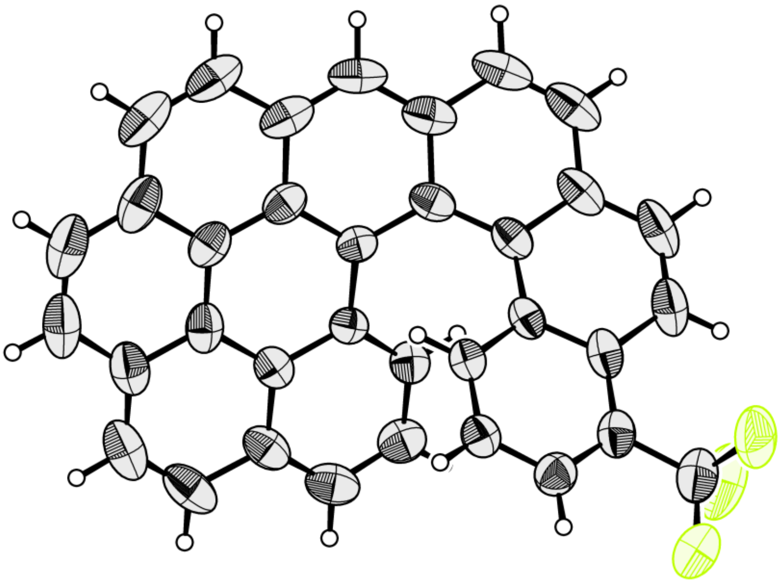

Supplementary Figure 72. X-ray crystallographic structure of racemic **O8H$_{\mathbf{CF3}}$** (only *M* enantiomer is shown). Thermal ellipsoids are shown at a 50% probability level.

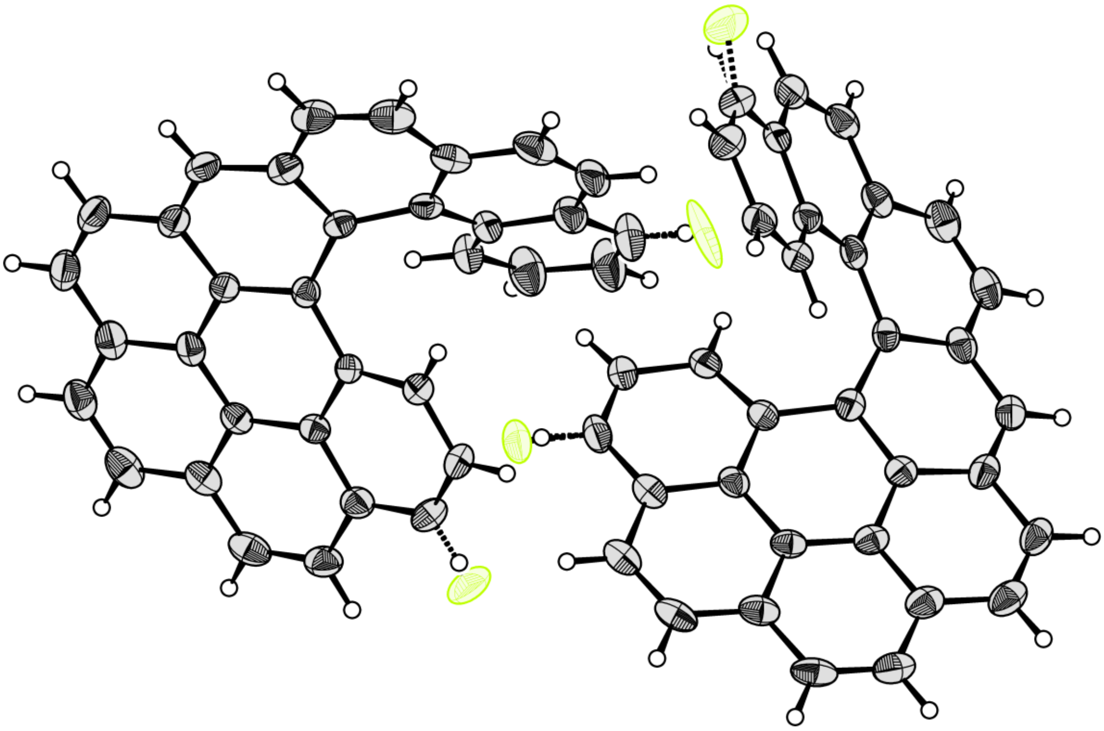

Supplementary Figure 73. X-ray crystallographic structure of the mixed crystal of racemic **O8H$_{\mathbf{F}}$-α** and **O8H$_{\mathbf{F}}$-β** at a ratio of 87:13. Thermal ellipsoids are shown at a 50% probability level.

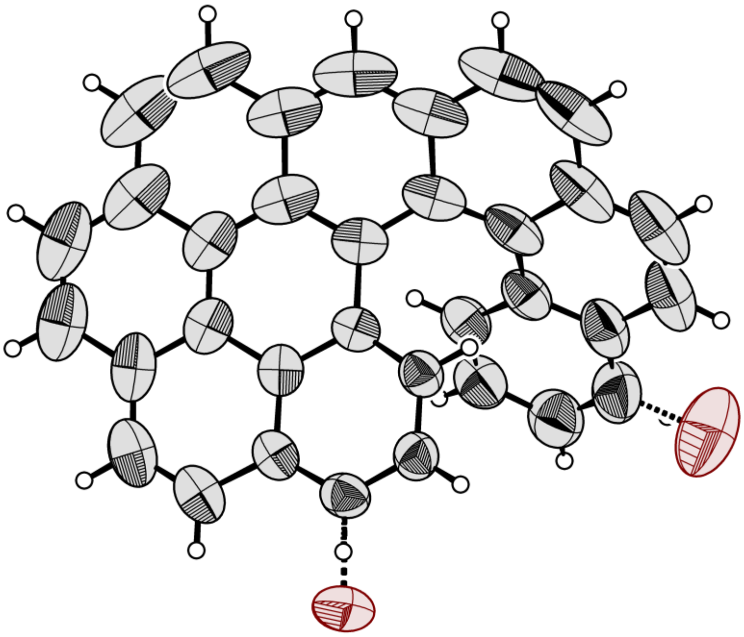

Supplementary Figure 74. X-ray crystallographic structure of the mixed crystal of racemic **O8H$_{Br}$-α** and **O8H$_{Br}$-β** at a ratio of 90:10 (only *P* enantiomer is shown). Thermal ellipsoids are shown at a 50% probability level.

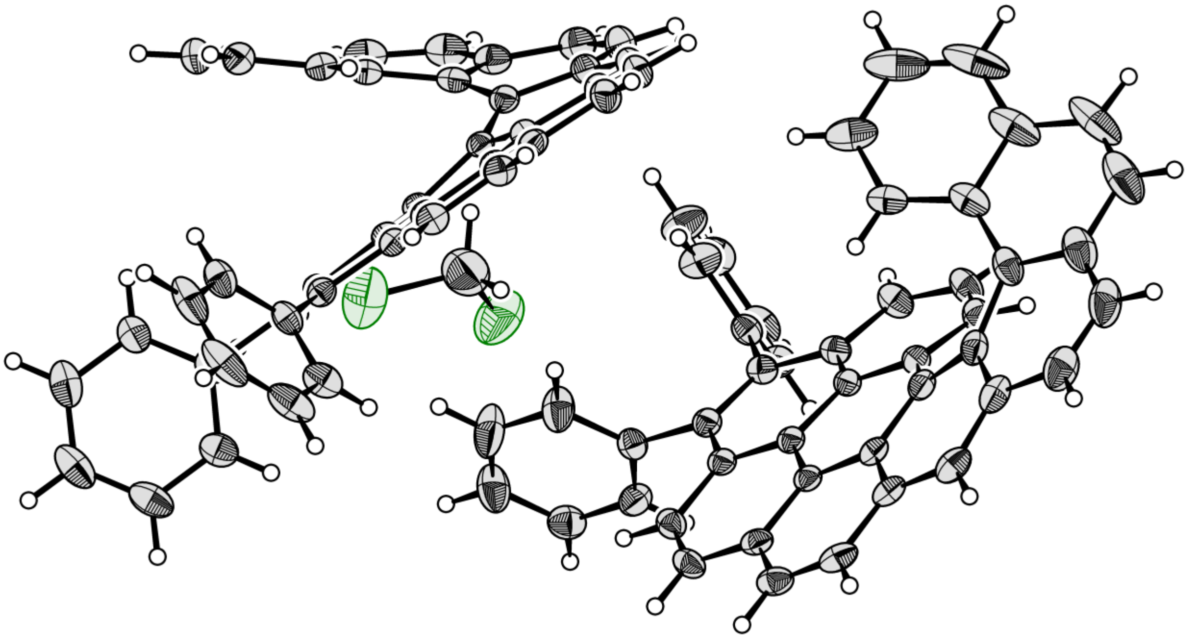

Supplementary Figure 75. X-ray crystallographic structure of racemic 2**O8H$_{2py}$**·CH_2Cl_2 (only *P* enantiomers are shown). Thermal ellipsoids are shown at a 50% probability level.

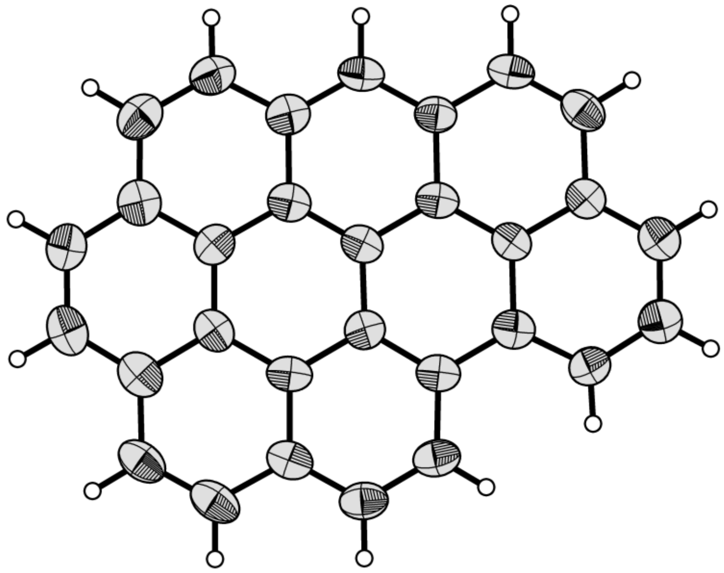

Supplementary Figure 76. X-ray crystallographic structure of **OO7H**. Thermal ellipsoids are shown at a 50% probability level.

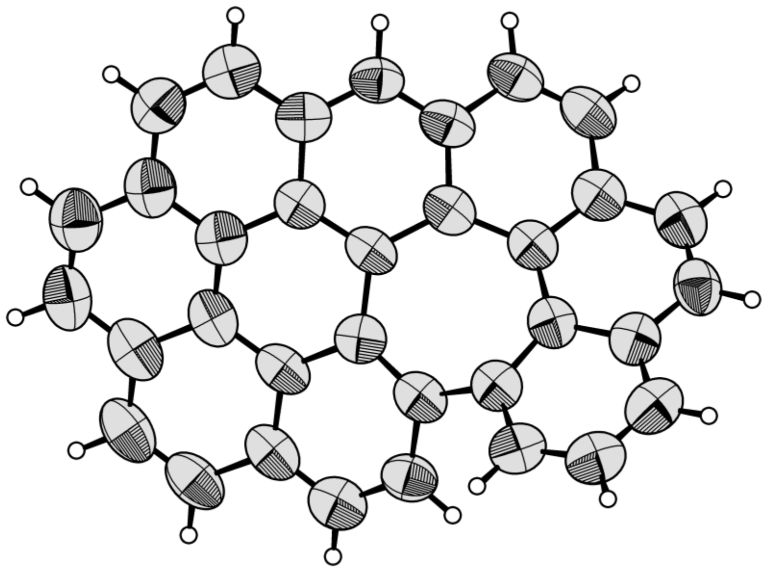

Supplementary Figure 77. X-ray crystallographic structure of **OO8H**. Thermal ellipsoids are shown at a 50% probability level.

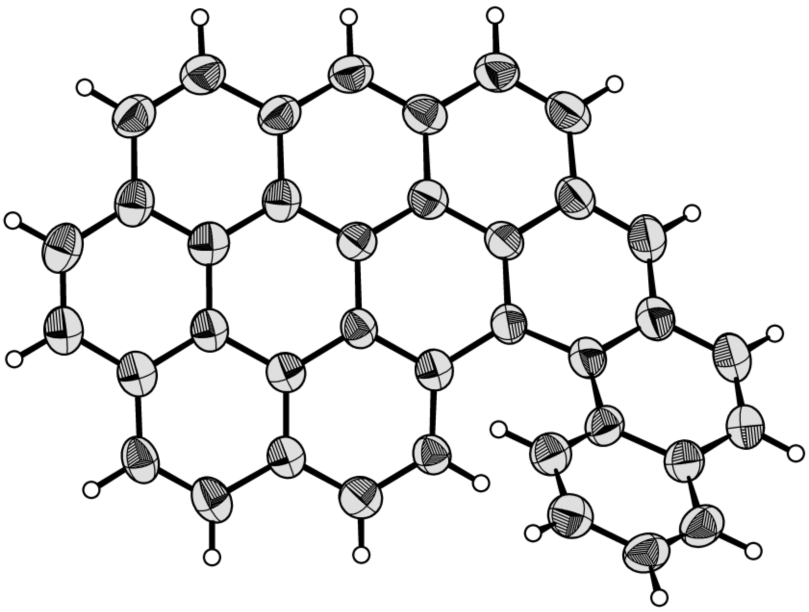

Supplementary Figure 78. X-ray crystallographic structure of **OO9H**. Thermal ellipsoids are shown at a 50% probability level.

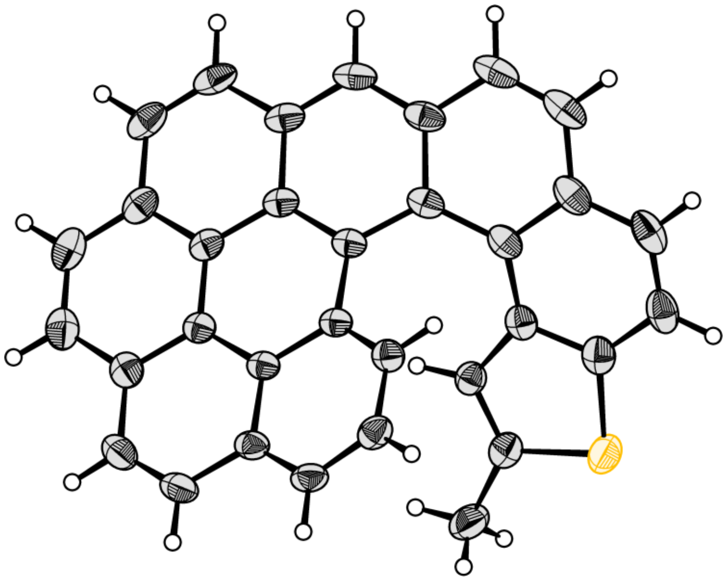

Supplementary Figure 79. X-ray crystallographic structure of racemic **O8H$_{\mathbf{mt}}$** (only *P* enantiomer is shown). Thermal ellipsoids are shown at a 50% probability level.

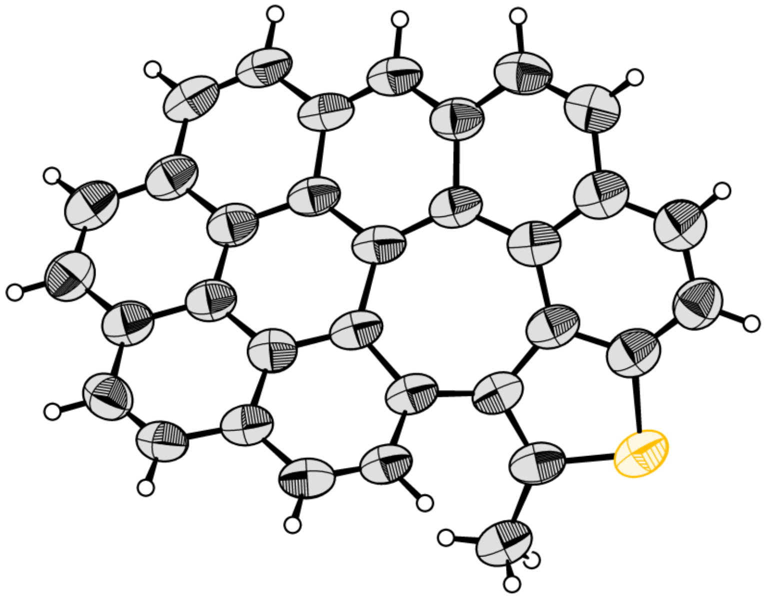

Supplementary Figure 80. X-ray crystallographic structure of racemic **OO8H$_{mt}$** (only *P* enantiomer is shown). Thermal ellipsoids are shown at a 50% probability level.

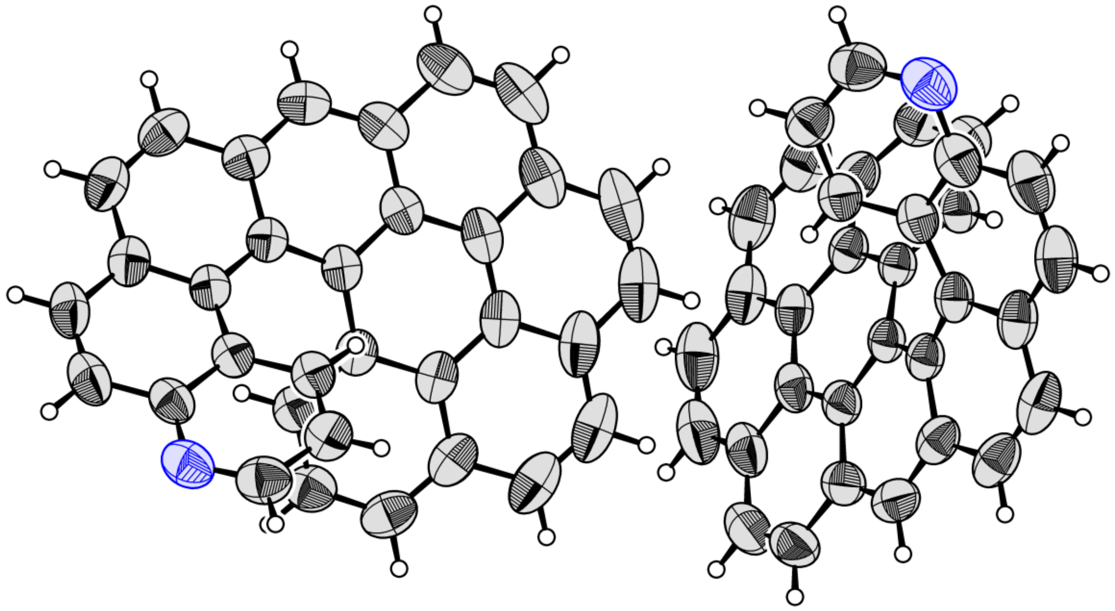

Supplementary Figure 81. X-ray crystallographic structure of racemic **O8H$_{a}$** (only *P* enantiomers are shown). Thermal ellipsoids are shown at a 50% probability level.

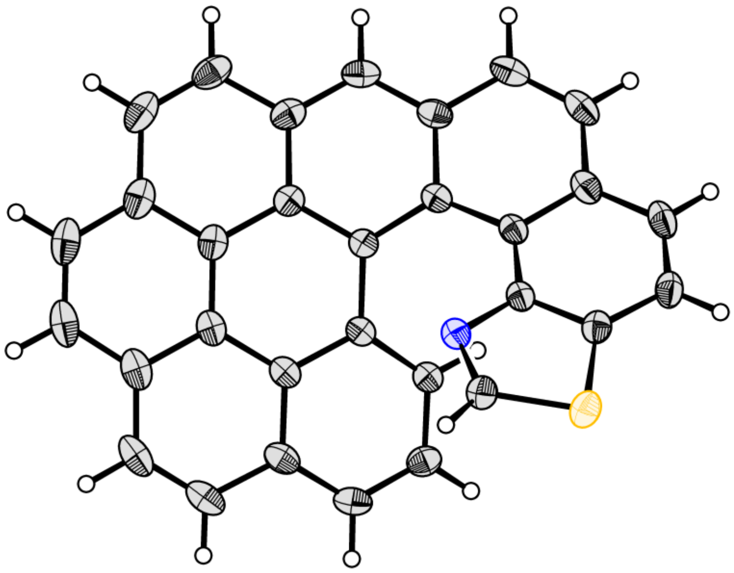

Supplementary Figure 82. X-ray crystallographic structure of racemic **O8H$_{ta}$** (only *M* enantiomers are shown). Thermal ellipsoids are shown at a 50% probability level.

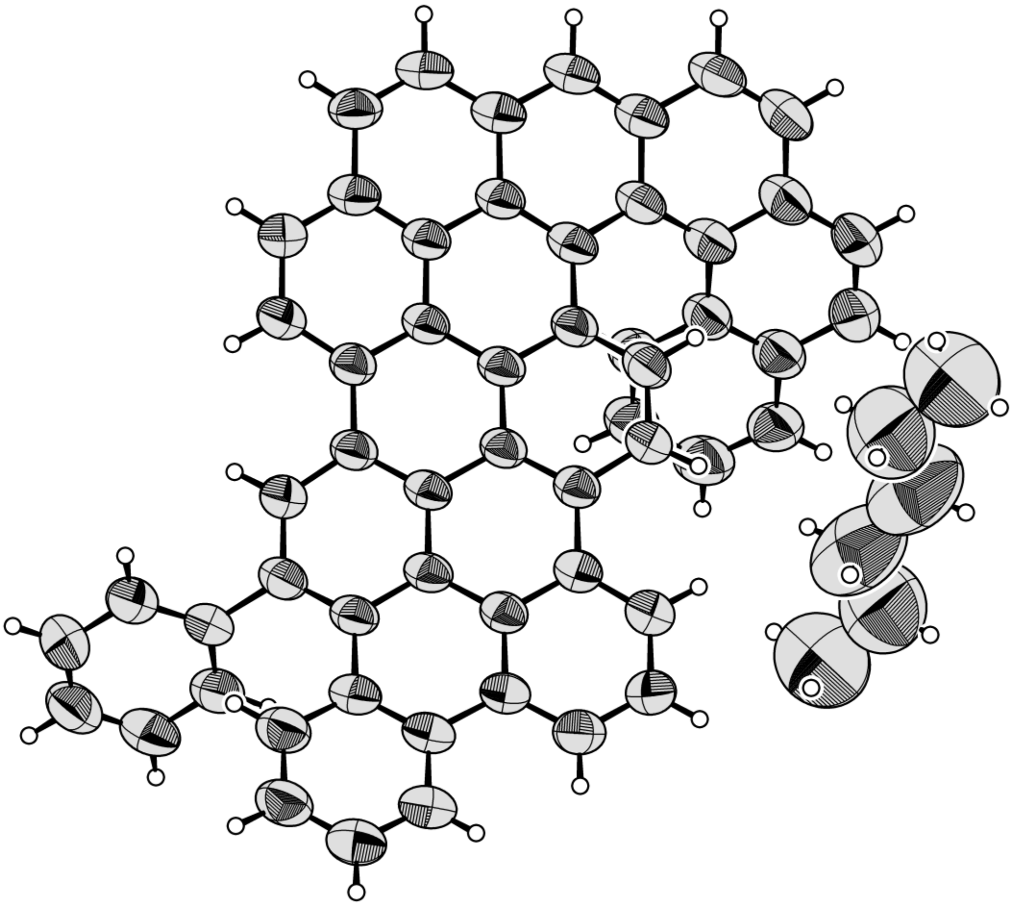

Supplementary Figure 83. X-ray crystallographic structure of racemic **O6H$_{DPT}$** (only *P* enantiomers are shown) with *n*-hexane. Thermal ellipsoids are shown at a 50% probability level.

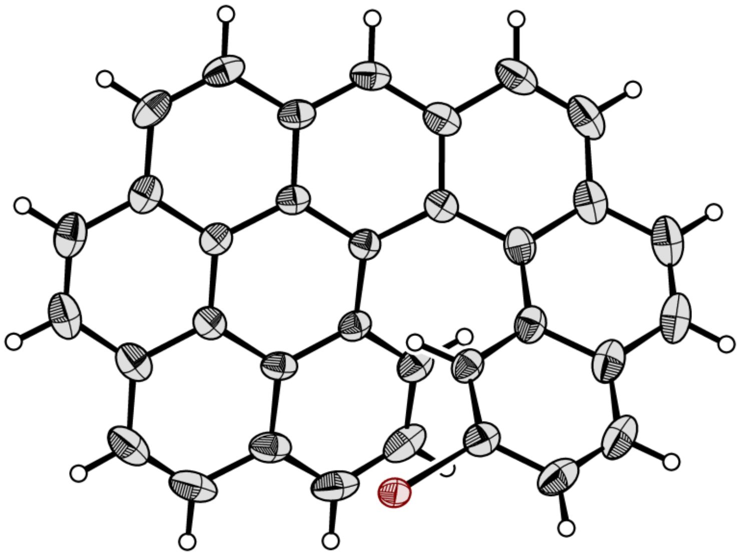

Supplementary Figure 84. X-ray crystallographic structure of racemic **O8H'$_{\mathbf{Br}}$** (only *M* enantiomers are shown). Thermal ellipsoids are shown at a 50% probability level.

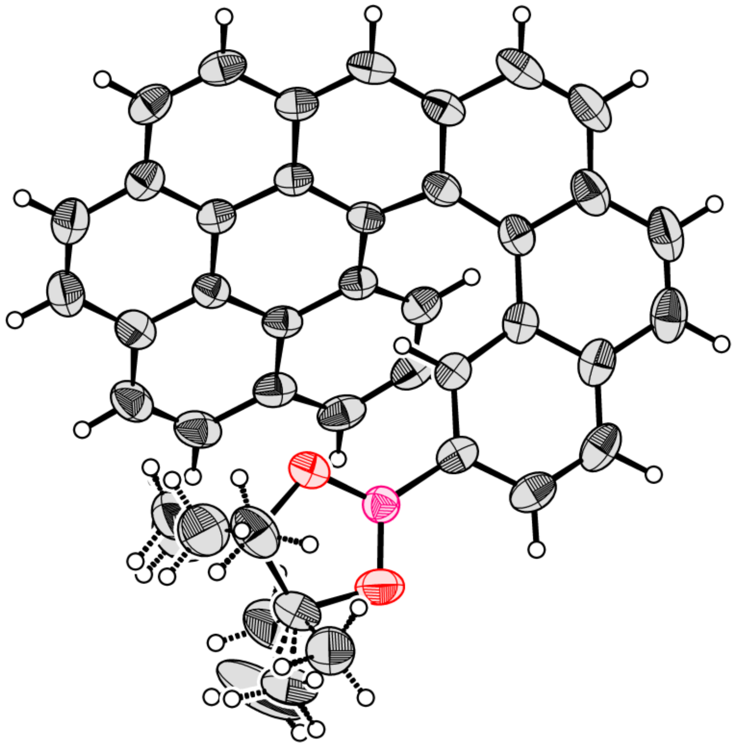

Supplementary Figure 85. X-ray crystallographic structure of racemic **O8H'$_{\mathbf{Bpin}}$** (only *M* enantiomers are shown). Thermal ellipsoids are shown at a 50% probability level.

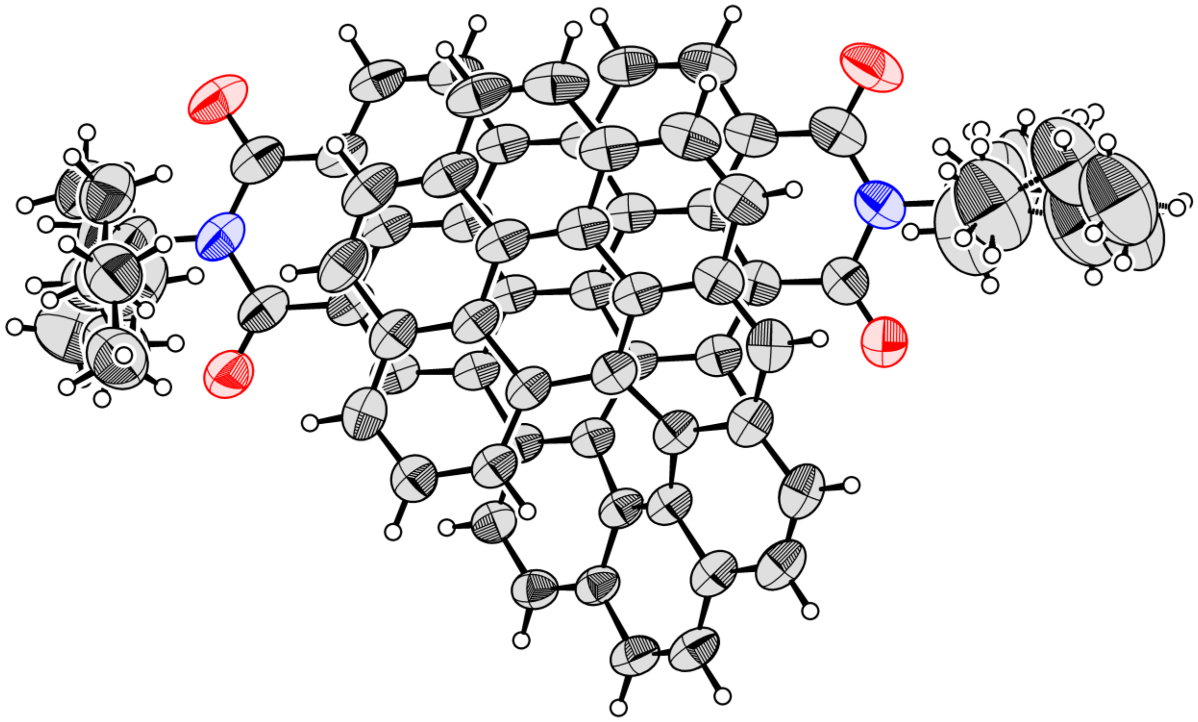

Supplementary Figure 86. X-ray crystallographic structure of racemic **CO8H'$_{\mathbf{PDI}}$** (only *P* enantiomers are shown). Thermal ellipsoids are shown at a 50% probability level.

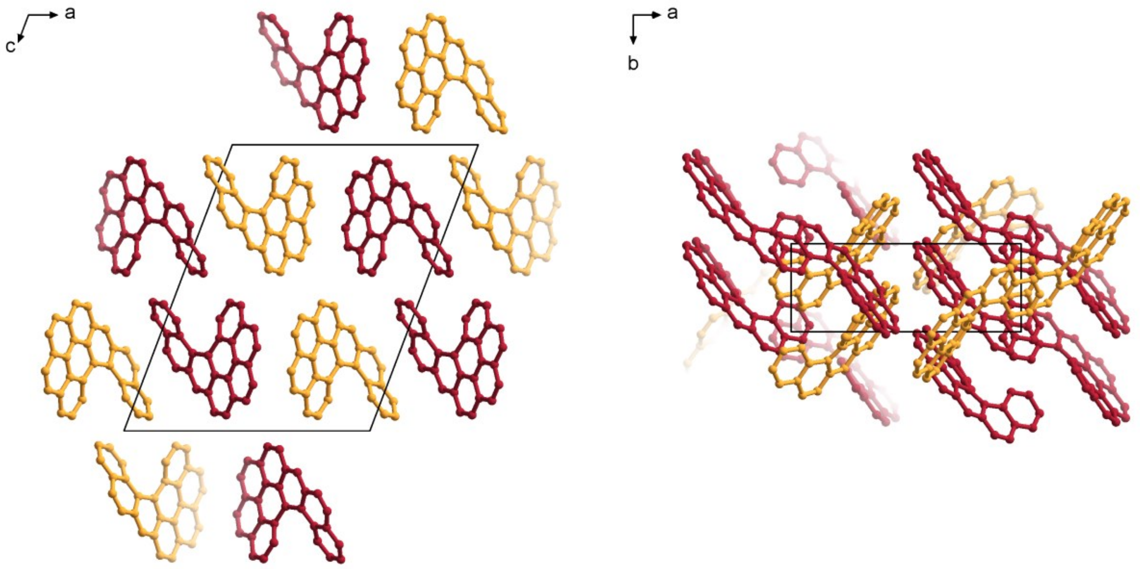

Supplementary Figure 87. Crystal packing diagrams of **O7H**. Hydrogen atoms are omitted for clarity.

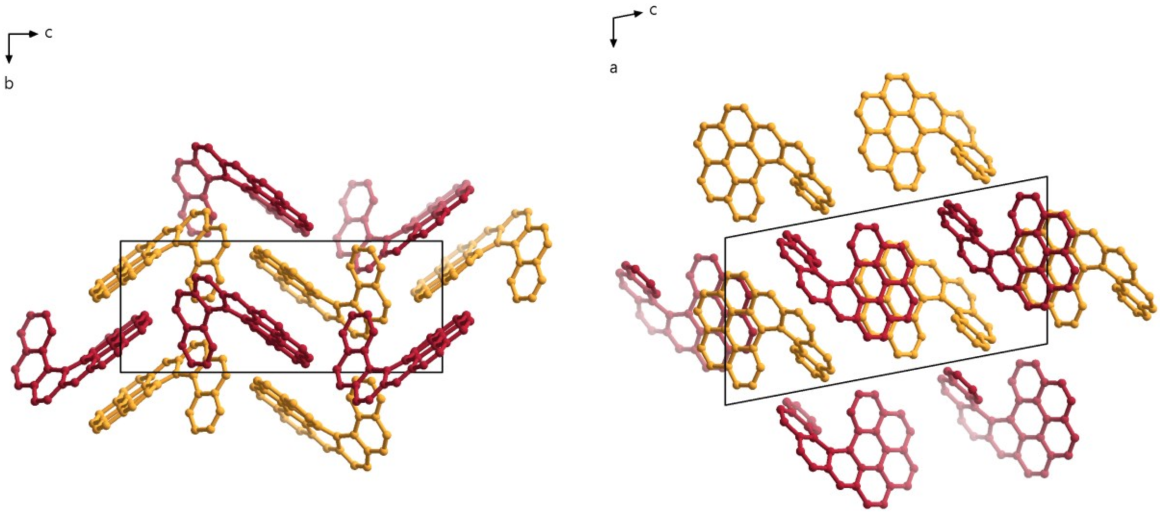

Supplementary Figure 88. Crystal packing diagrams of **O8H**. Hydrogen atoms are omitted for clarity.

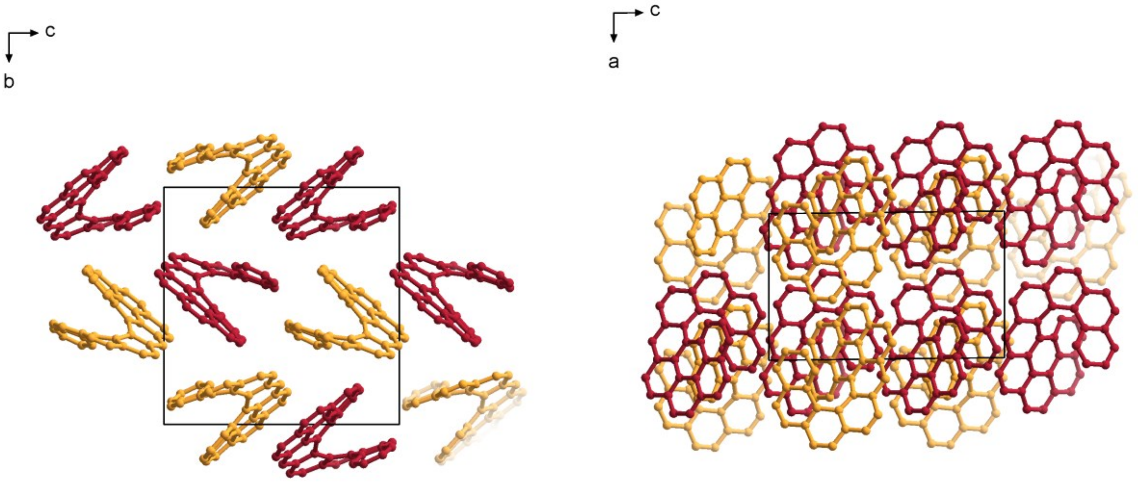

Supplementary Figure 89. Crystal packing diagrams of **O9H**. Hydrogen atoms are omitted for clarity.

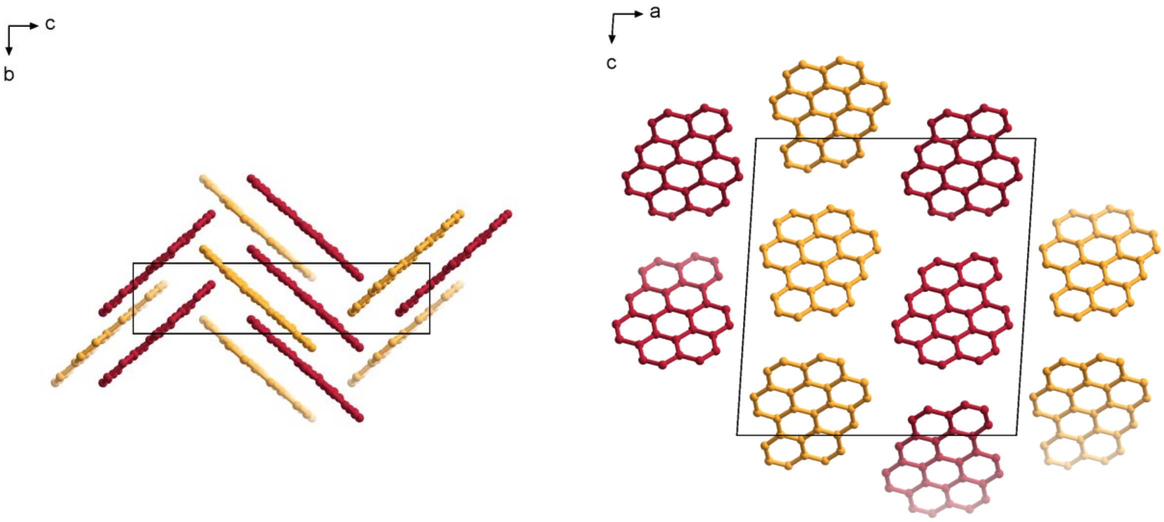

Supplementary Figure 90. Crystal packing diagrams of **OO7H**. Hydrogen atoms are omitted for clarity.

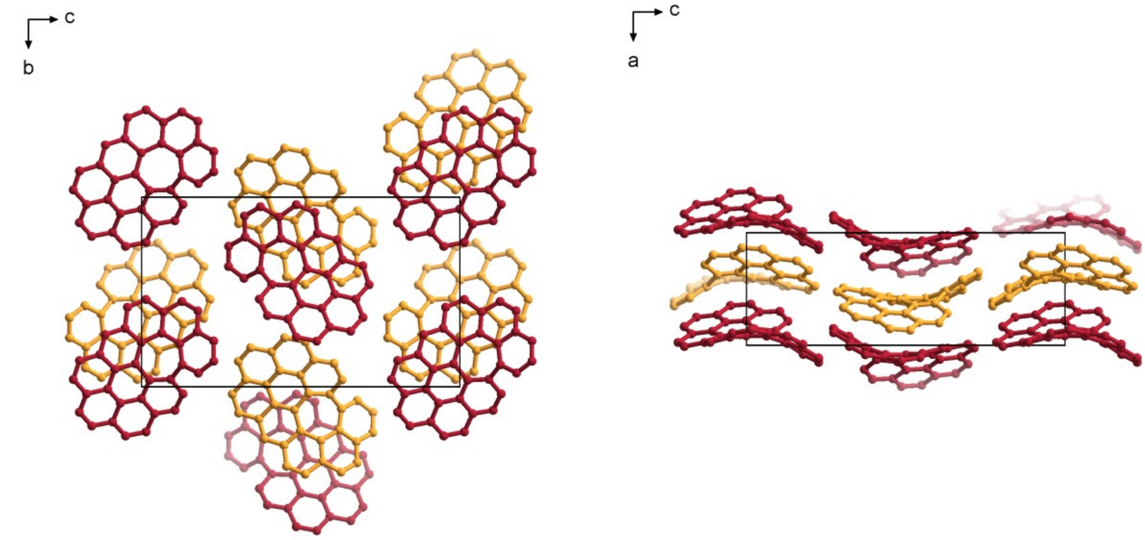

Supplementary Figure 91. Crystal packing diagrams of **OO8H**. Hydrogen atoms are omitted for clarity.

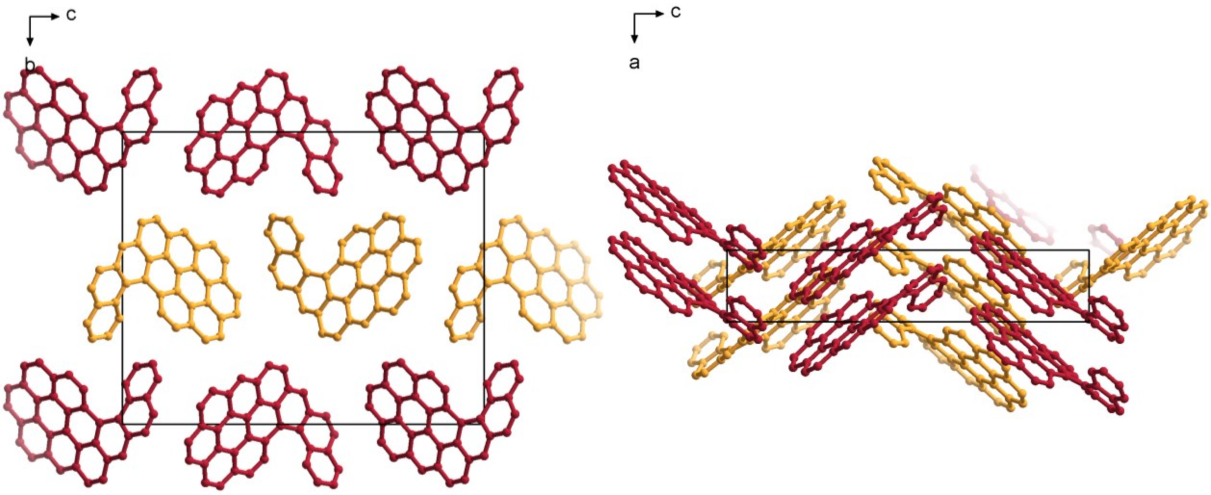

Supplementary Figure 92. Crystal packing diagrams of **OO9H**. Hydrogen atoms are omitted for clarity.

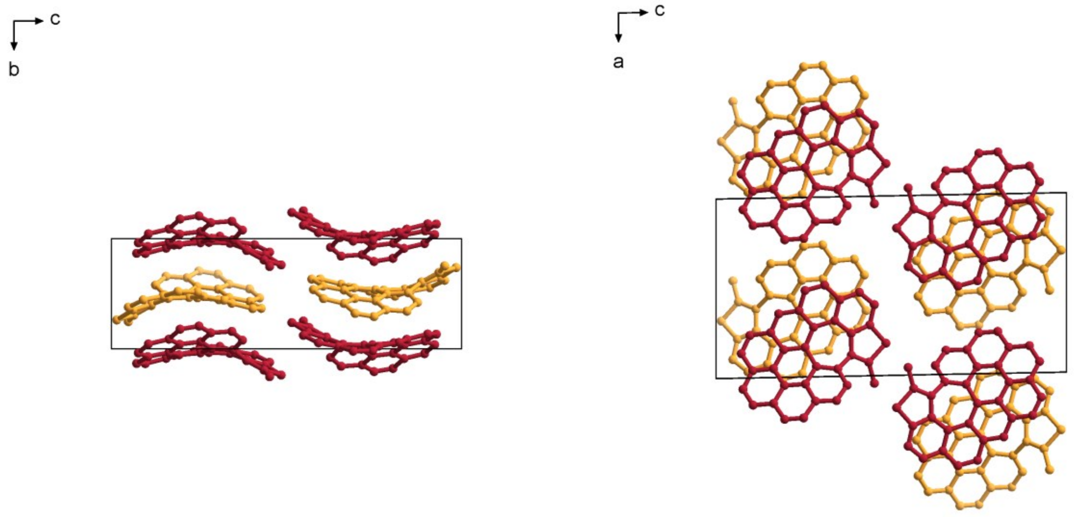

Supplementary Figure 93. Crystal packing diagrams of **OO8H**$_{\mathbf{mt}}$. Hydrogen atoms are omitted for clarity.

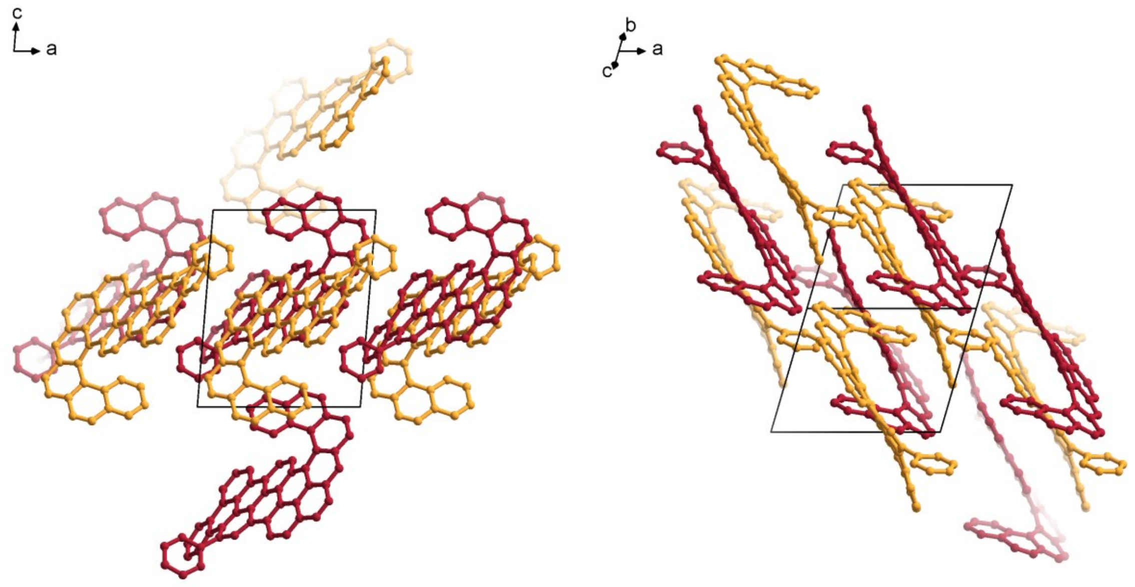

Supplementary Figure 94. Crystal packing diagrams of **O6H**$_{\mathbf{DPT}}$. Solvent molecules and hydrogen atoms are omitted for clarity.

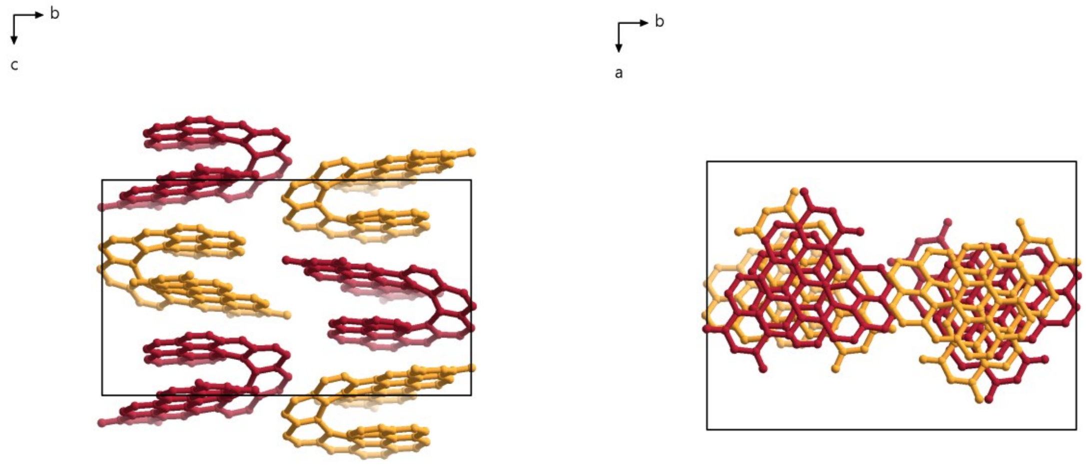

Supplementary Figure 95. Crystal packing diagrams of **O8H'$_{PDI}$**. Side alkyl chains and hydrogen atoms are omitted for clarity.

1.8 Chiral SFC Analysis

Analytical chiral supercritical fluid chromatography (SFC) separation for the rearranged products **O8H**, **O9H** and **O8Ha** obtained from racemic and enantiopure helicene precursors **8H**, **9H** and **8Ha** were performed on a Waters SFC200 system with a UV detector at 220 nm, using Waters AMY1 (2.1 mm × 150 mm) as the chromatography column and CO_2/CH_3OH = 6/4 as the mobile phase. The flowrate was set at 1.5 mL·min^{-1} and the column temperature was set at 35 °C. The samples were dissolved in chloroform before injection into the column.

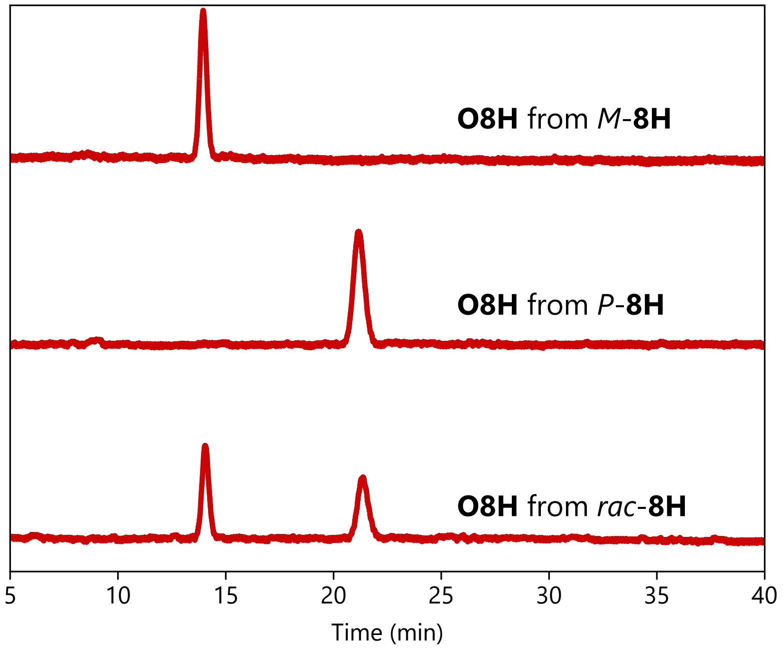

Supplementary Figure 96. Chromatograms of **O8H** obtained from *rac*-, *P*- or *M*-**8H** (Waters AMY1, CO_2/CH_3OH = 6/4).

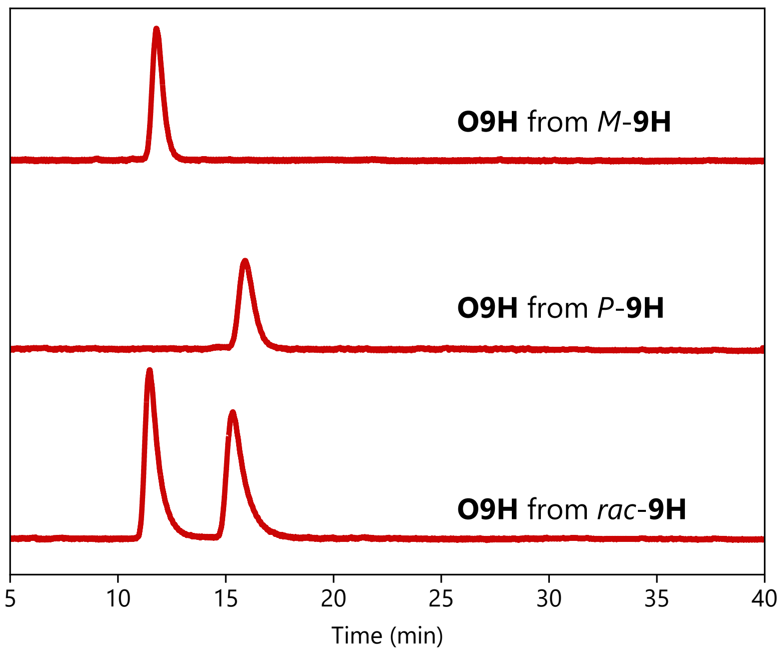

Supplementary Figure 97. Chromatograms of **O9H** obtained from *rac*-, *P*- or *M*-**9H** (Waters AMY1, CO_2/CH_3OH = 6/4).

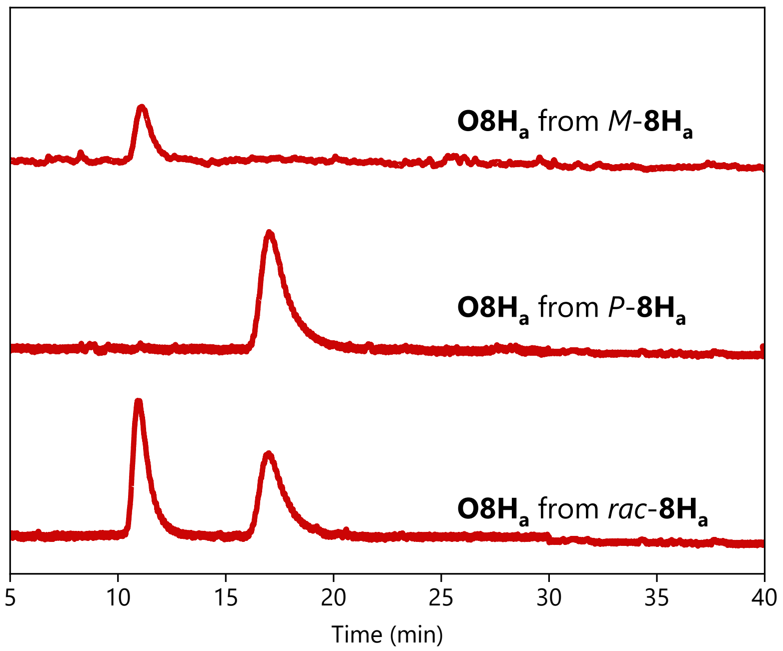

Supplementary Figure 98. Chromatograms of **O8Ha** obtained from *rac*-, *P*- or *M*-**8Ha** (Waters AMY1, CO_2/CH_3OH = 6/4).

2. Optical and Chiroptical Spectroscopy

2.1 General Information

UV-vis absorption spectra were recorded on a Shimazu UV-2600 UV-vis Spectrophotometer. Electronic circular dichroism (ECD) spectra were recorded on a JASCO T-815 Spectropolarimeter. Fluorescence spectra were recorded on a Shimazu RF-6000 Spectro Fluorophotometer. Circularly polarized luminescence (CPL) spectra were recorded on a JASCO CPL-300 Spectrophotometer. All spectra were recorded at 293 K in a 1 cm quartz cuvette unless otherwise mentioned.

2.2 Additional Optical Spectra

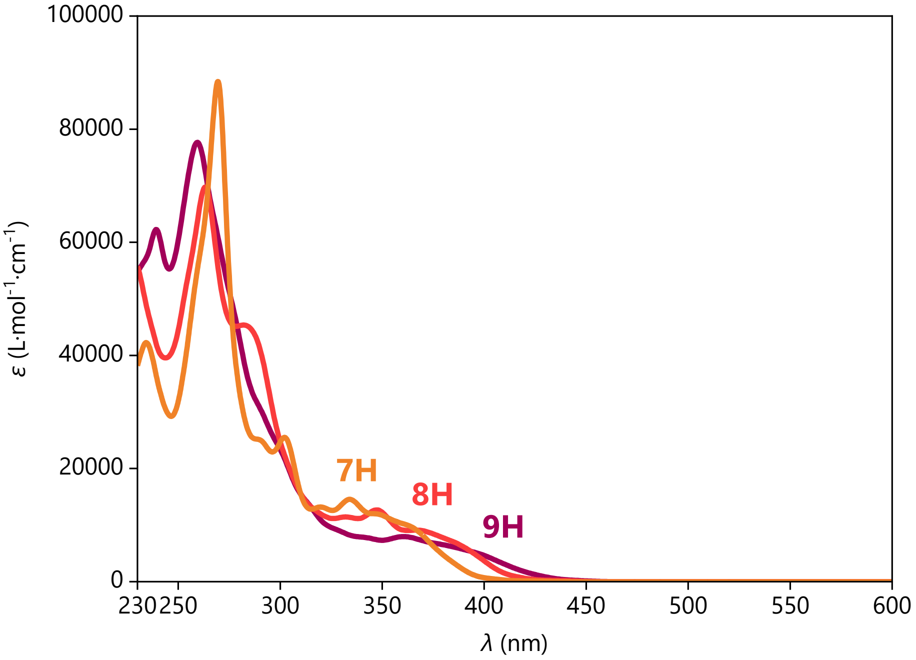

Supplementary Figure 99. UV-vis absorption spectra of **7H**, **8H** and **9H** (c = 2 × 10^{-5} mol·L^{-1}, in CH_2Cl_2).

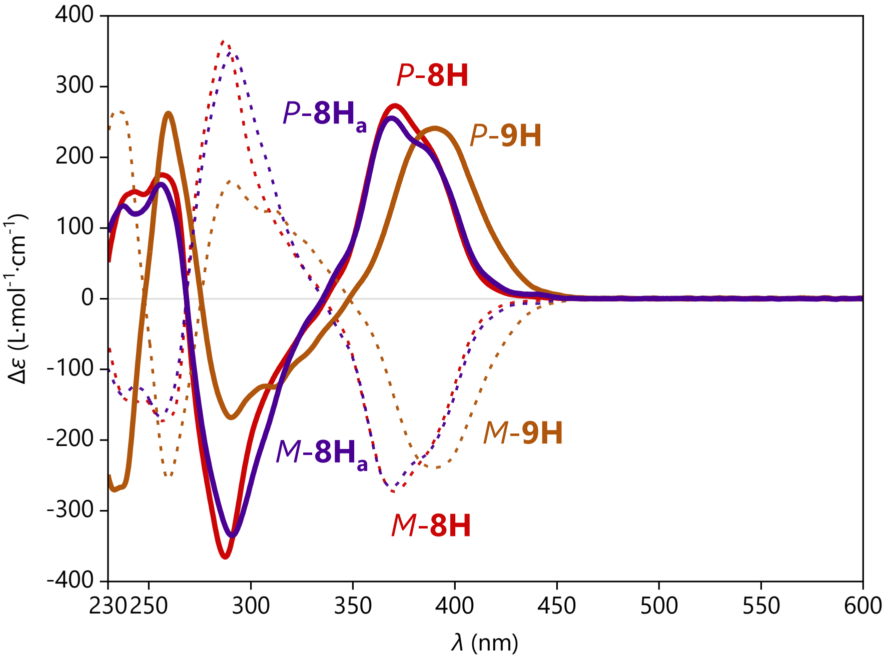

Supplementary Figure 100. ECD spectra of enantiopure **8H**, **9H** and **8Ha** (c = 2 × 10^{-5} mol·L^{-1}, in CH_2Cl_2).

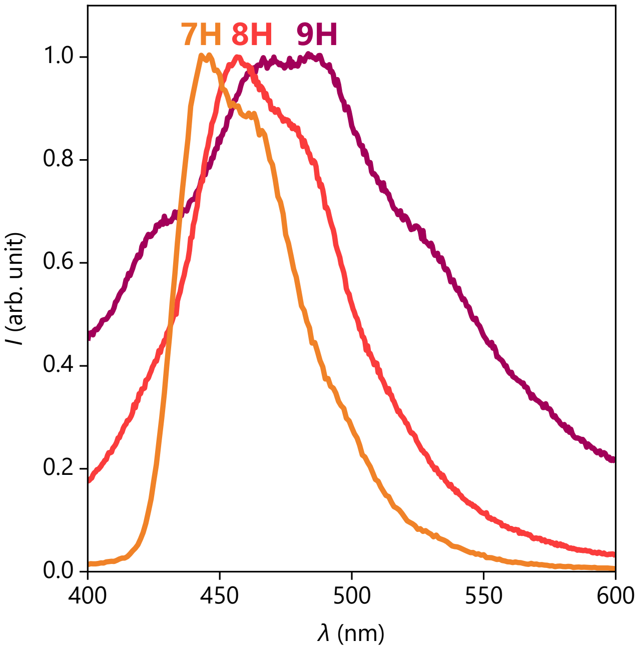

Supplementary Figure 101. Fluorescence spectra of **7H**, **8H** and **9H** excited at 365 nm (c = 2 × 10^{-5} mol·L^{-1}, in CH_2Cl_2).

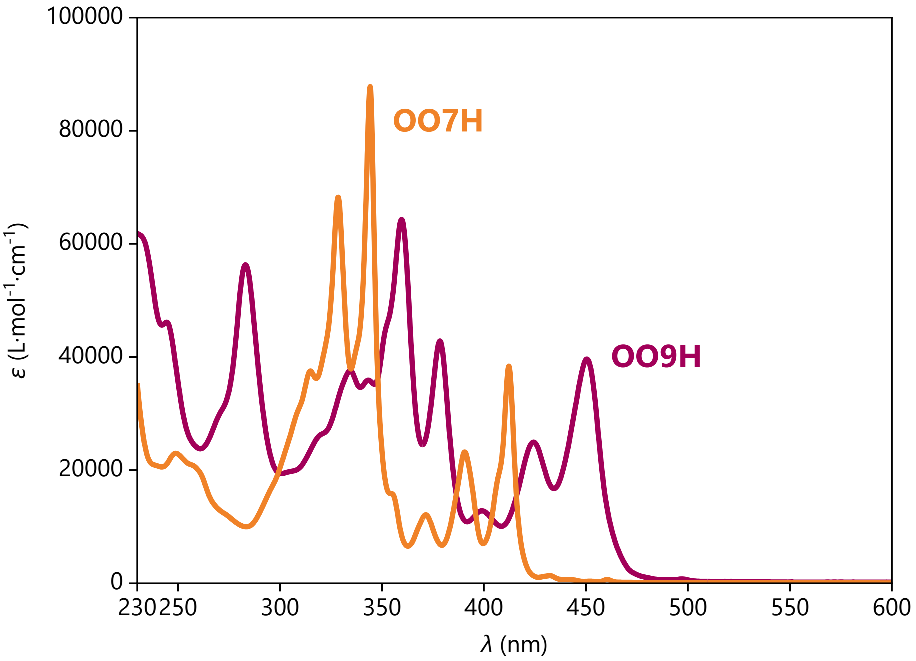

Supplementary Figure 102. UV-vis absorption spectra of **OO7H** and **OO9H** (c = 2 × 10^{-5} mol·L^{-1}, in CH_2Cl_2).

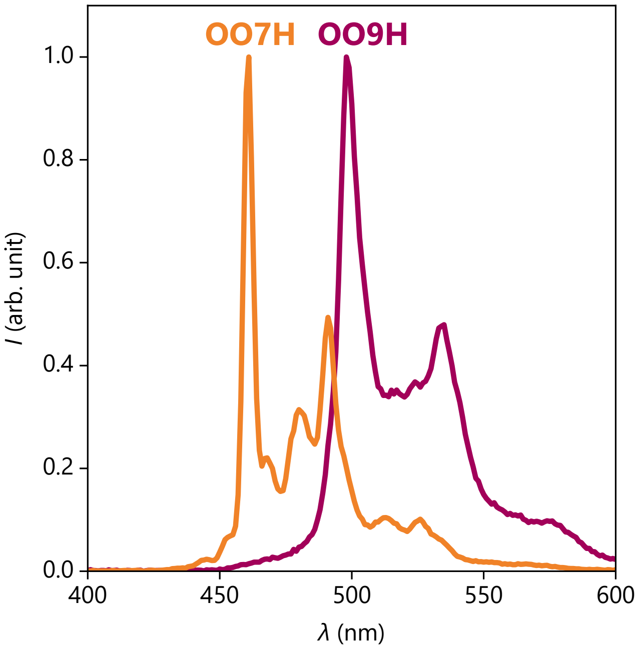

Supplementary Figure 103. Fluorescence spectra of **OO7H** and **OO9H** excited at 365 nm (c = 2 × 10^{-5} mol·L^{-1}, in CH_2Cl_2).

3. Theoretical Calculations

3.1 General Information

Density functional theory (DFT) calculations were carried out using Gaussian 09 program.[5] Geometrical optimization calculations were carried out at the PBE0-D3[6]/def2-SVP[7] level with the SMD continuum solvent model[8] for CH_2Cl_2 without any symmetry assumptions unless otherwise stated. Harmonic vibration frequency calculations were performed at the same level for verifying the resulting geometries as local minima (with all the frequencies real) or saddle points (with only one imaginary frequency). The assignment of the saddle points was performed using the intrinsic reaction coordinate (IRC) calculations.

Nucleus-independent chemical shifts (NICS)[9] values were calculated at the GIAO-PBE0/def2-SVP level on the optimized structures. Coordinates of the centroid of the corresponding six (or seven) benzenoid carbon atoms were used for NICS(0). Harmonic oscillator model of aromaticity (HOMA)[10] values were calculated according to the single-crystal structures using the equation as follows:

$$\mathrm{HOMA} = 1 - \frac{\alpha}{n}\sum_{i}^{n}(R_{\mathrm{opt}} - R_i)^2$$

Where α refers to an empirical constant which is 257.7, n refers to the number of carbon atoms in one cycle (for six-membered rings, n = 6 and for the seven-membered rings n = 7), R_{opt} refers to the optimal value for CC bond length, which is 1.388 (in Å), and R_i refers to individual CC bond length (in Å).

Time-dependent density functional theory (TD-DFT) calculations were performed at the PBE0-D3/def2-SVP level with the SMD continuum solvent model for CH_2Cl_2 on the optimized geometries. Absorption and electronic circular dichroism (ECD) spectra were simulated via TD-DFT calculations of the lowest 200 vertical singlet electronic excitations with Gaussian broadening for the oscillator strength f and rotatory strength R_{vel} (in velocity form), and half-width at half-height was set at 0.2 eV. No special shift or scaling was applied unless otherwise mentioned. Isosurfaces of molecular orbitals (MOs) were drawn using IQmol program with the isovalue set to 0.1.

For clarity and simplicity, all the chiral compounds were calculated on the *P*-enantiomers unless otherwise mentioned. For **CO8H'$_{\mathbf{PDI}}$**, the calculation model was simplified to **CO8H'$_{\mathbf{PDI\text{-}CH3}}$** using methyl groups to replace the long alkyl chains.

3.2 Calculations on Free Energies

3.2.1 Free Energy Comparison between [*n*]Helicenes and [*n*]Phenacenes

Supplementary Table 26. Free energy difference between [*n*]helicenes (n = 5 to 9) and [*n*]phenacenes ($\Delta G_i = G_{[i]\text{helicene}} - G_{[i]\text{phenacene}}$, in kcal·mol^{-1}, at 298.15 K).

ΔG_5	ΔG_6	ΔG_7	ΔG_8	ΔG_9
13.1	15.6	18.9	24.8	25.6

3.2.2 Calculation Results for the Oxidative Cyclo-rearrangement of 8H and 7H

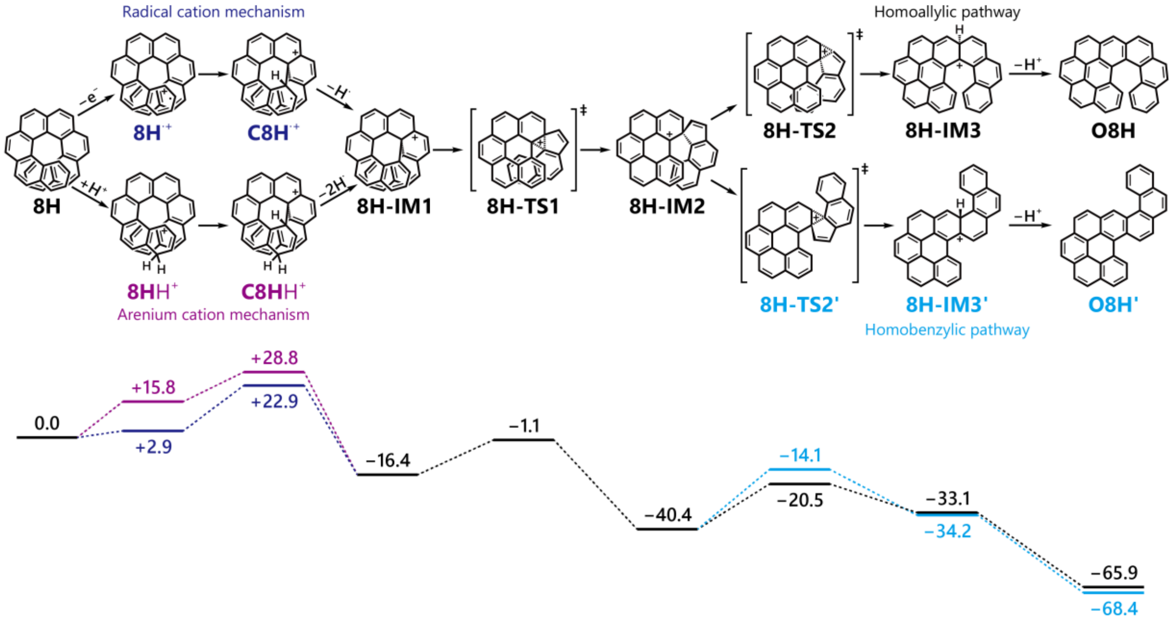

Supplementary Figure 104. Computed energy profiles (free energy in kcal·mol^{-1}) for the rearrangement mechanism of **8H**. Free energy of **8H** is set as 0.

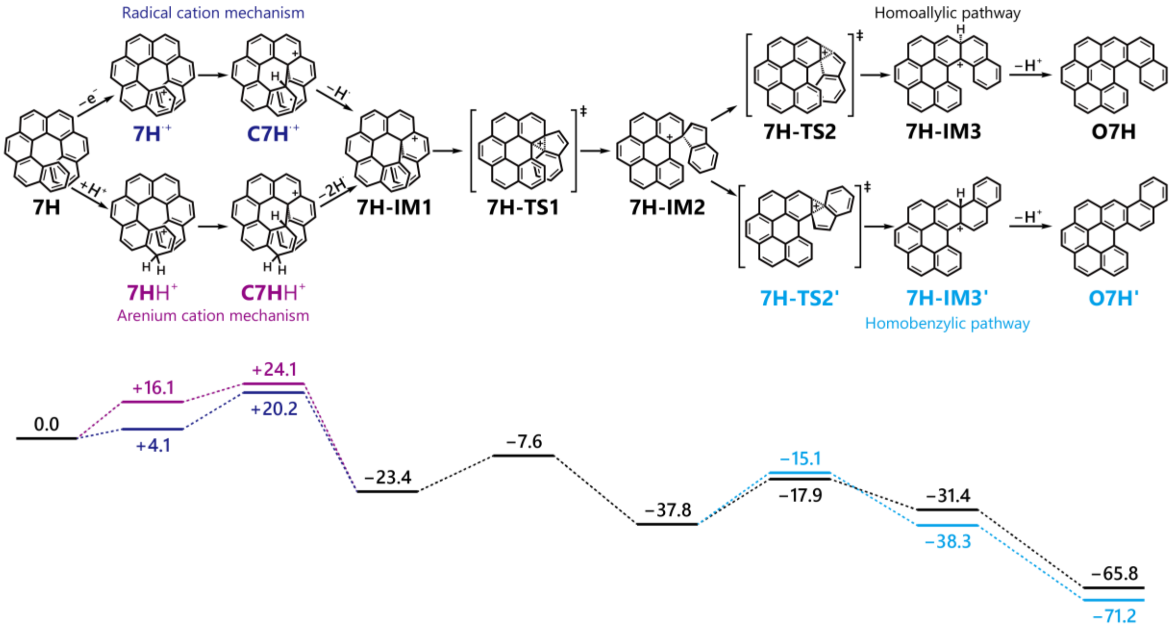

Supplementary Figure 105. Computed energy profiles (free energy in kcal·mol^{-1}) for the rearrangement mechanism of **7H**. Free energy of **7H** is set as 0.

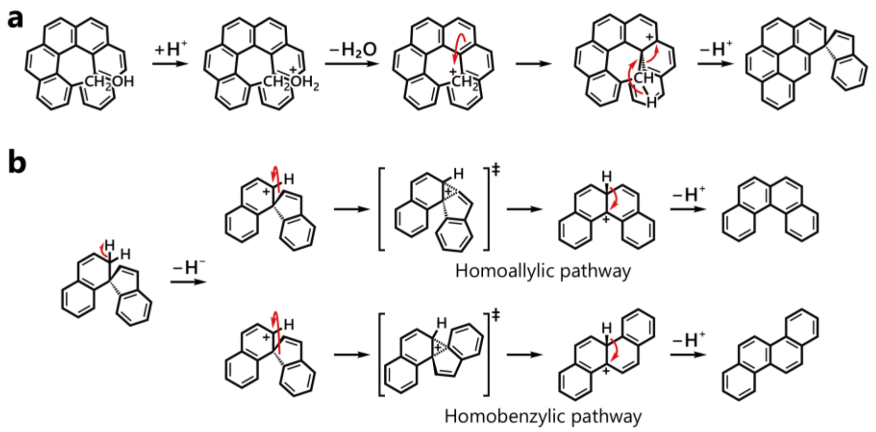

Supplementary Figure 106. Relevant migration reactions for the first and second 1,2-migrations according to the previous literatures.[11,12] (a) Rearrangement of (1-[6]helicenyl)methanol. (b) Rearrangement of 2'*H*-spiro[indene-1,1'-naphthalene] in homoallylic and homobenzylic pathways.

In the radical cation mechanism, we used (DDQ + CF_3SO_3H) to (DDQH$^{\cdot}$ + $CF_3SO_3^-$) as the e$^-$ acceptor, and DDQH$^{\cdot}$ to $DDQH_2$ as the H$^{\cdot}$ acceptor. In the arenium cation mechanism, we use CF_3SO_3H to $CF_3SO_3^-$ as the H$^+$ donor, and DDQ to $DDQH_2$ as the 2H$^{\cdot}$ acceptor. In the last deprotonation process, we use $CF_3SO_3^-$ to CF_3SO_3H as the H$^+$ acceptor. The calculations were at the PBE0-D3/ma-SVP[13] level of theory with SMD solvent model for CH_2Cl_2. The basis set was changed from def2-SVP to ma-SVP under the consideration that compared with def2-SVP, ma-SVP contains additional diffuse functions which could have a better description of the anionic $CF_3SO_3^-$.

The free energies for the cationic intermediates might be overestimated due to the formation of the intimate ion-pairs with the anionic $CF_3SO_3^-$ in solution, which might lower the total free energies. Therefore, we took the intermediate **8H**H^+ as the example to estimate this ion-pair complexation energy ΔG_{ip} using the following equation:

$$\begin{aligned}\Delta G_{\text{ip}} &= G_{\mathbf{8H}\text{H}^+\cdot\text{CF}_3\text{SO}_3^-} - G_{\mathbf{8H}\text{H}^+} - G_{\text{CF}_3\text{SO}_3^-}\\ &= (-2265.782580) - (-1305.382236) - (-960.395780)\ \text{Hartree}\\ &= -0.004564\ \text{Hartree}\\ &= -2.9\ \text{kcal}\cdot\text{mol}^{-1}\end{aligned}$$

Where $G_{\mathbf{8H}\text{H}^+\cdot\text{CF}_3\text{SO}_3^-}$, $G_{\mathbf{8H}\text{H}^+}$ and $G_{\text{CF}_3\text{SO}_3^-}$ are the free energies of the intimate ion-pair of **8H**$H^+\cdot CF_3SO_3^-$, the independent cationic **8H**H^+ and the independent anionic $CF_3SO_3^-$, respectively. The calculations were at the PBE0-D3/ma-SVP level of theory with the SMD solvent model for CH_2Cl_2. The result revealed that the complexation to form the intimate ion-pairs only has limited influence on the free energies of the cationic intermediates (ca. −2.9 kcal·mol^{-1}), and for this work, without consideration of this ion-pair complexation would be acceptable.

For the two 1,2-migrations, according to the relevant literature works was reported (Supplementary Figure 106),[11,12] the second 1,2-migration from **8H-IM2** (or **7H-IM2**) was also proposed in two plausible pathways (the homoallylic way vs. the homobenzylic way) to afford inward product **O8H** (or **O7H**) or outward product **O8H'** (or **O7H'**). Calculation results indicated that although the homobenzylic way is more thermodynamically favored, its transition state possesses higher energy than the transition state of homoallylic way. Therefore, in consideration of the kinetic concerns, the second 1,2-migration prefers to undergo through the homoallylic pathway.

3.2.3 Calculation Results for the Initial Cyclization Process of O7H, O8H and O9H

We studied the energy changes of the initial cyclization process of **O7H**, **O8H** and **O9H** to understand their cyclization behaviors. Radical cation mechanism was chosen for the calculation due to its simplicity. **O7H**$^{\cdot+}$ revealed a low activation energy of +20.9 kcal·mol^{-1}, which could interpret its easy cyclization even in the non-degassed solutions. We proposed two pathways for **O8H**: the heptagonal pathway and the hexagonal pathway, to understand whether it favors a formation of heptagon by Scholl reaction or a second oxidative cyclo-rearrangement by forming a hexagon in the initial stage. Activation energy indicated that forming a hexagonal is kinetically disfavored in the ambient temperature. For **O9H**, due to its unsymmetrical structures, the second oxidative cyclization was proposed two occur at both sides: the benzoperylene-side pathway and the helicene-side pathway. Calculation results indicated that the benzoperylene-side pathway is more kinetically favored than the helicene-side pathway.

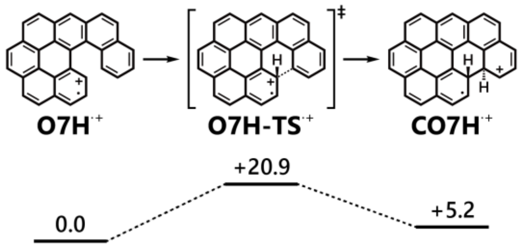

Supplementary Figure 107. Computed energy profiles (free energy in kcal·mol^{-1}) for the initial cyclization of **O7H**$^{\cdot+}$ through a radical cation mechanism. Free energy of **O7H**$^{\cdot+}$ is set as 0.

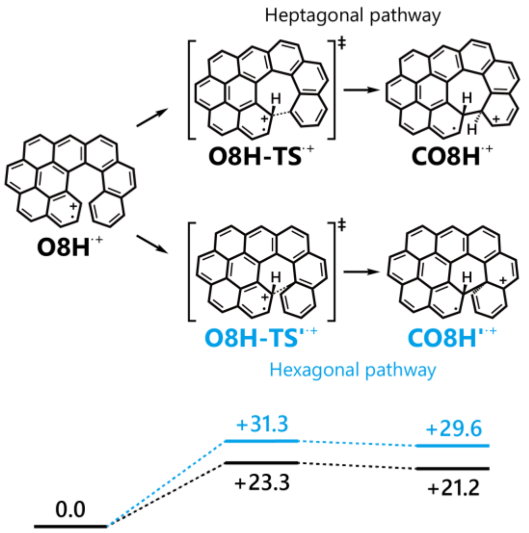

Supplementary Figure 108. Computed energy profiles (free energy in kcal·mol^{-1}) for the initial cyclization of **O8H**$^{\cdot+}$ through a radical cation mechanism. Free energy of **O8H**$^{\cdot+}$ is set as 0.

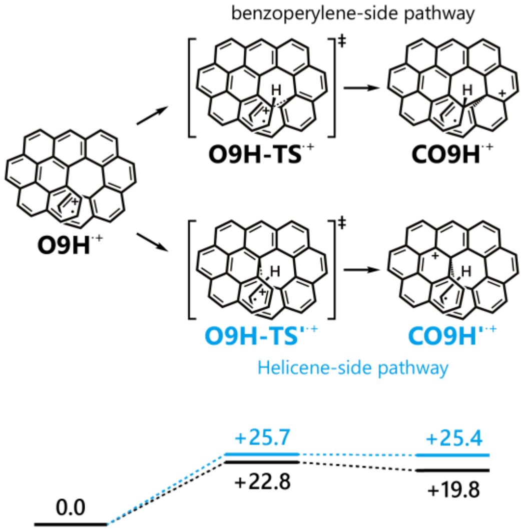

Supplementary Figure 109. Computed energy profiles (free energy in kcal·mol^{-1}) for the initial cyclization of **O9H**$^{\cdot+}$ through a radical cation mechanism. Free energy of **O9H**$^{\cdot+}$ is set as 0.

3.2.4 Racemization Barrier of O7H, O8H, OO8H, OO8H$_{mt}$ and OO9H

We studied the racemization barriers of the [5]helicene-cored **O7H** and **OO9H** with the comparison of primitive [5]helicene **5H**, and the [6]helicene-cored **O8H** with the comparison of primitive [6]helicene **6H**, and the negatively curved **OO8H** and **OO8H$_{mt}$**. The racemization transition states **O7H***, **O8H***, **OO8H***, **OO8H$_{mt}$***, **OO9H***, **5H*** and **6H*** were calculated with only one imaginary frequency, and were verified by IRC calculations.

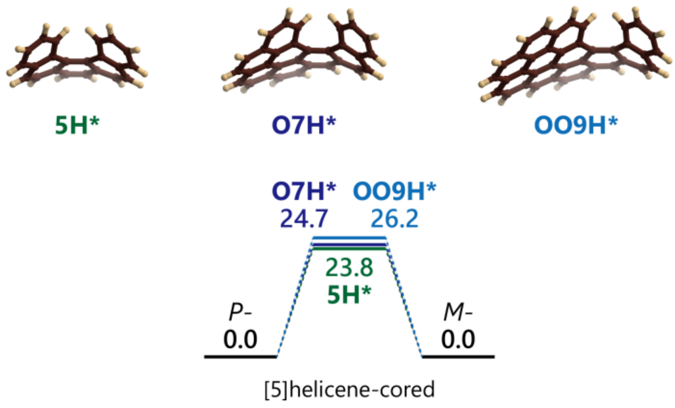

Supplementary Figure 110. Computed energy profiles (free energy in kcal·mol^{-1}) for the racemization of [5]helicene-cored **5H**, **O7H** and **OO9H**. Free energies of *P* or *M* configuration is set as 0.

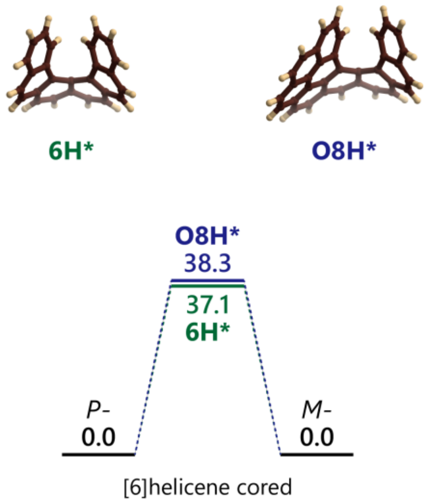

Supplementary Figure 111. Computed energy profiles (free energy in kcal·mol^{-1}) for the racemization of [6]helicene-cored **6H** and **O8H**. Free energies of *P* or *M* configuration is set as 0.

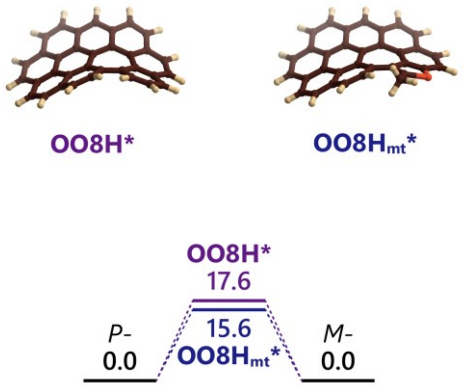

Supplementary Figure 112. Computed energy profiles (free energy in kcal·mol^{-1}) for the racemization of negatively curved **OO8H** and **OO8H$_{mt}$**. Free energies of *P* or *M* configuration is set as 0.

3.2.5 Energies of Stationary Points

Supplementary Table 27. Electronic energies (*E*), zero-point energy corrected energies (*E* + *ZPE*), electronic and thermal enthalpies (*H*), Gibbs free energies (*G*) of the stationary points calculated at the PBE0-D3/def2-SVP level of theory with the SMD continuum solvent model for CH_2Cl_2.

	E (Hartree)	*E* + *ZPE* (Hartree)	*H* (Hartree)	*G* (Hartree)
CF_3SO_3H	−960.778042	−960.738745	−960.730109	−960.771886
$CF_3SO_3^-$	−960.334808	−960.306890	−960.298833	−960.339512
CF_3SO_3H [a]	−960.814166	−960.775626	−960.766862	−960.808972
$CF_3SO_3^-$ [a]	−960.390552	−960.363261	−960.355170	−960.395780
DDQ	−1483.488579	−1483.423987	−1483.410939	−1483.463617
DDQH$^·$	−1484.108416	−1484.032829	−1484.019602	−1484.072974
$DDQH_2$	−1484.751723	−1484.663735	−1484.650121	−1484.703256
DDQ [a]	−1483.517024	−1483.453053	−1483.439901	−1483.492133
DDQH$^·$ [a]	−1484.138122	−1484.062908	−1484.049626	−1484.103126
$DDQH_2$ [a]	−1484.782593	−1484.694767	−1484.681107	−1484.734398
**8H**	−1305.354753	−1304.922810	−1304.899398	−1304.972902
**8H**$^{·+}$	−1305.150496	−1304.719416	−1304.695763	−1304.770541
**8H**H$^+$	−1305.752551	−1305.310488	−1305.286476	−1305.360900
**C8H**$^{·+}$	−1305.118513	−1304.687243	−1304.664005	−1304.738547
**C8H**H$^+$	−1305.735151	−1305.291300	−1305.268078	−1305.340265
**8H-IM1**	−1304.543345	−1304.121423	−1304.098840	−1304.169931
**8H-TS1**	−1304.517932	−1304.097308	−1304.074915	−1304.145556
**8H-IM2**	−1304.581864	−1304.158773	−1304.136140	−1304.208209
**8H-TS2**	−1304.549090	−1304.127617	−1304.105384	−1304.176514
**8H-TS2'**	−1304.538475	−1304.117382	−1304.095133	−1304.166355
**8H-IM3**	−1304.569009	−1304.147460	−1304.124854	−1304.196505
**8H-IM3'**	−1304.571260	−1304.149147	−1304.126667	−1304.198384
**O8H**	−1304.199190	−1303.787395	−1303.765284	−1303.835718
**O8H'**	−1304.203173	−1303.790927	−1303.768799	−1303.839696
**8H**H$^+$ [a]	−1305.772351	−1305.331661	−1305.307498	−1305.382236
**8H**H$^+$·$CF_3SO_3^-$ [a]	−2266.183604	−2265.714844	−2265.681192	−2265.782580
**7H**	−1151.991991	−1151.606921	−1151.586210	−1151.653855
**7H**$^{·+}$	−1151.785655	−1151.401644	−1151.380644	−1151.449544
**7H**H$^+$	−1152.388840	−1151.993757	−1151.972406	−1152.041316
**C7H**$^{·+}$	−1151.761580	−1151.377287	−1151.356801	−1151.423925
**C7H**H$^+$	−1152.380071	−1151.982696	−1151.962219	−1152.028634
**7H-IM1**	−1151.192011	−1150.816682	−1150.796787	−1150.862097
**7H-TS1**	−1151.165017	−1150.791620	−1150.771879	−1150.836967
**7H-IM2**	−1151.213711	−1150.838215	−1150.818127	−1150.884976

	E (Hartree)	$E + ZPE$ (Hartree)	H (Hartree)	G (Hartree)
**7H-TS2**	−1151.181860	−1150.807756	−1150.788175	−1150.853375
**7H-TS2'**	−1151.176729	−1150.803047	−1150.783461	−1150.848784
**7H-IM3**	−1151.203582	−1150.829109	−1150.809236	−1150.874796
**7H-IM3'**	−1151.214729	−1150.839714	−1150.819826	−1150.885876
**O7H**	−1150.835711	−1150.471108	−1150.451644	−1150.516400
**O7H'**	−1150.844605	−1150.479455	−1150.459973	−1150.525085
**O7H**$^{\cdot+}$	−1150.643719	−1150.279106	−1150.259530	−1150.325198
**O7H-TS**$^{\cdot+}$	−1150.610201	−1150.247093	−1150.228269	−1150.291816
**CO7H**$^{\cdot+}$	−1150.637037	−1150.272040	−1150.253112	−1150.316977
**O8H**$^{\cdot+}$	−1304.008454	−1303.596560	−1303.574366	−1303.645603
**O8H-TS**$^{\cdot+}$	−1303.971654	−1303.560494	−1303.538994	−1303.608514
**CO8H**$^{\cdot+}$	−1303.975935	−1303.563795	−1303.542180	−1303.611884
**O8H-TS'**$^{\cdot+}$	−1303.957434	−1303.548327	−1303.526813	−1303.595715
**CO8H'**$^{\cdot+}$	−1303.961182	−1303.550816	−1303.529116	−1303.598407
**O9H**$^{\cdot+}$	−1457.373119	−1456.914155	−1456.889333	−1456.965889
**O9H-TS**$^{\cdot+}$	−1457.336914	−1456.879383	−1456.855364	−1456.929529
**CO9H**$^{\cdot+}$	−1457.342328	−1456.883695	−1456.859457	−1456.934289
**O9H-TS'**$^{\cdot+}$	−1457.332246	−1456.875228	−1456.851303	−1456.925005
**CO9H'**$^{\cdot+}$	−1457.332434	−1456.874667	−1456.850312	−1456.925362
[5]phenacene	−845.285476	−844.994280	−844.978456	−845.037850
[6]phenacene	−998.653173	−998.314351	−998.295889	−998.360710
[7]phenacene	−1152.020759	−1151.634732	−1151.614264	−1151.683931
[8]phenacene	−1305.388458	−1304.954968	−1304.930870	−1305.012462
[9]phenacene	−1458.756064	−1458.274820	−1458.248176	−1458.332045
**5H**	−845.266637	−844.975950	−844.960498	−845.016963
**6H**	−998.629501	−998.291648	−998.273536	−998.335805
**9H**	−1458.718322	−1458.239248	−1458.213341	−1458.291158
**5H***	−845.227996	−844.938365	−844.923347	−844.978968
**6H***	−998.569463	−998.233389	−998.215831	−998.276661
**O7H***	−1150.796710	−1150.432743	−1150.413892	−1150.477114
**O8H***	−1304.138363	−1303.727731	−1303.706349	−1303.774675
**OO9H***	−1456.370850	−1455.933142	−1455.910170	−1455.981953
**OO8H***	−1302.971400	−1302.581412	−1302.561025	−1302.627269
**OO8H**$_{mt}$*****	−1662.876099	−1662.490976	−1662.469605	−1662.538036
**OO9H**	−1456.412692	−1455.974098	−1455.950564	−1456.023737
**OO8H**	−1302.998973	−1302.608470	−1302.587491	−1302.655243
**OO8H**$_{mt}$	−1662.899615	−1662.514652	−1662.492351	−1662.562855

[a] ma-SVP was used as the basis set.

3.3 Calculated UV-vis Absorption and ECD Spectra

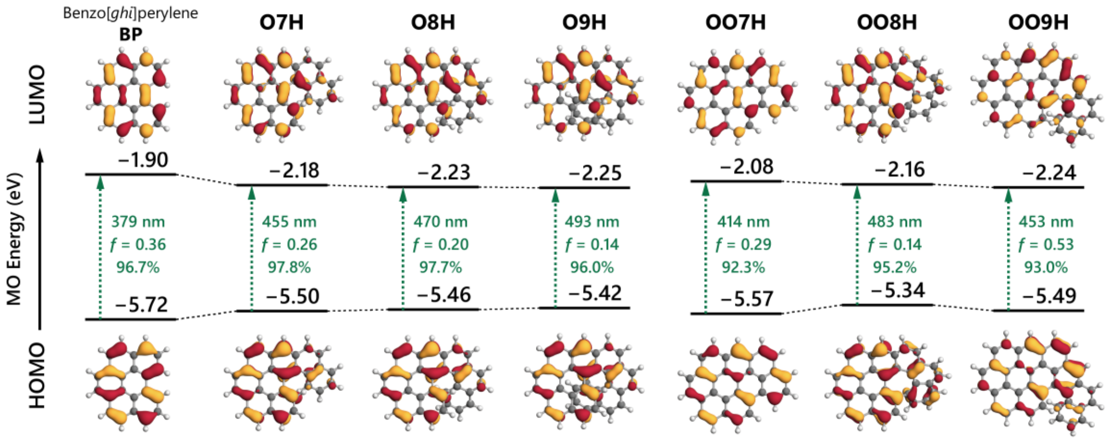

Supplementary Figure 113. Frontier molecular orbitals (MO) and energy diagram of benzo[*ghi*]perylene, **O7H**, **O8H**, **O9H**, **OO7H**, **OO8H** and **OO9H**. The *f* values refer to the oscillator strength and the values in percentage represents the contribution of HOMO→LUMO transition for the first excitation.

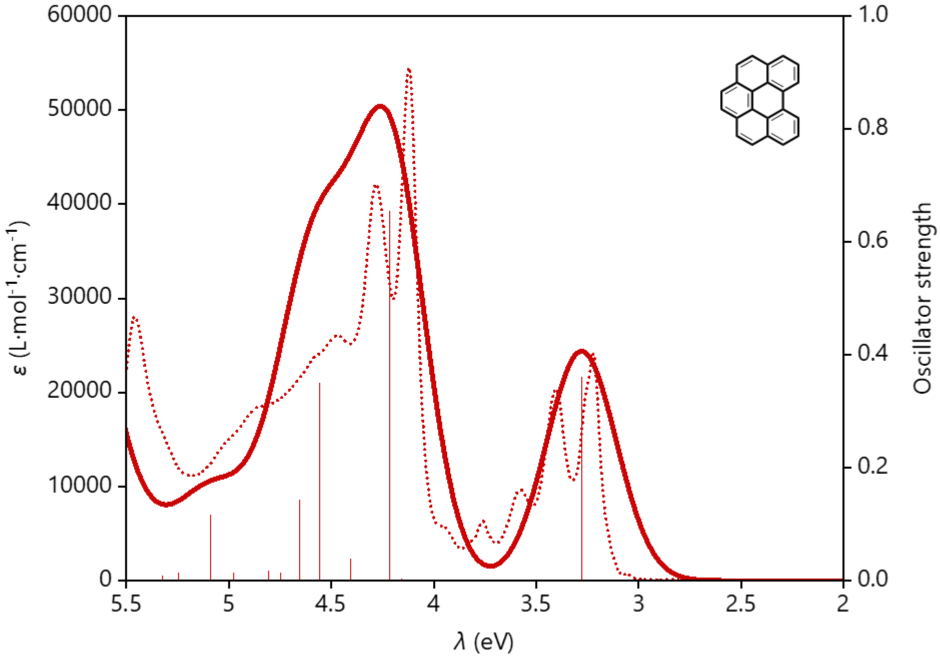

Supplementary Figure 114. Calculated (solid) and experimental UV-vis absorption spectra (dotted, *c* = 2 × 10^{-5} mol·L^{-1}, in CH_2Cl_2) of benzo[*ghi*]perylene. Calculated excitation energies and oscillator strengths *f* are displayed as stick spectrum.

Supplementary Table 28. Selected dominant excitations and occupied (occ) – unoccupied (unocc) MO pair contributions (greater than 10%) of benzo[*ghi*]perylene.

Excitation	*E* (eV)	λ (nm)	*f*	occ no.	unocc no.	%
1	3.27	379	0.3613	72	73	96.7
2	3.48	357	0.0002	72	74	49.7
				71	73	49.0
3	4.15	299	0.0039	72	75	88.3
4	4.21	295	0.6556	71	73	48.6
				72	74	47.5

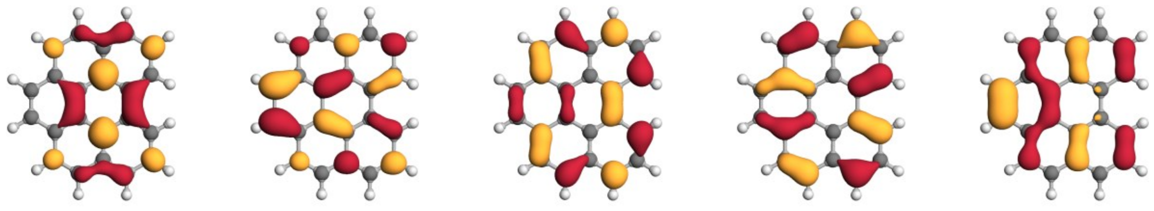

MO75 −0.55 eV | **MO74** −1.19 eV | **MO73** (LUMO) −1.90 eV | **MO72** (HOMO) −5.72 eV | **MO71** −6.41 eV

Supplementary Figure 115. Isosurfaces of MOs involved in the selected transitions of benzo[*ghi*]perylene.

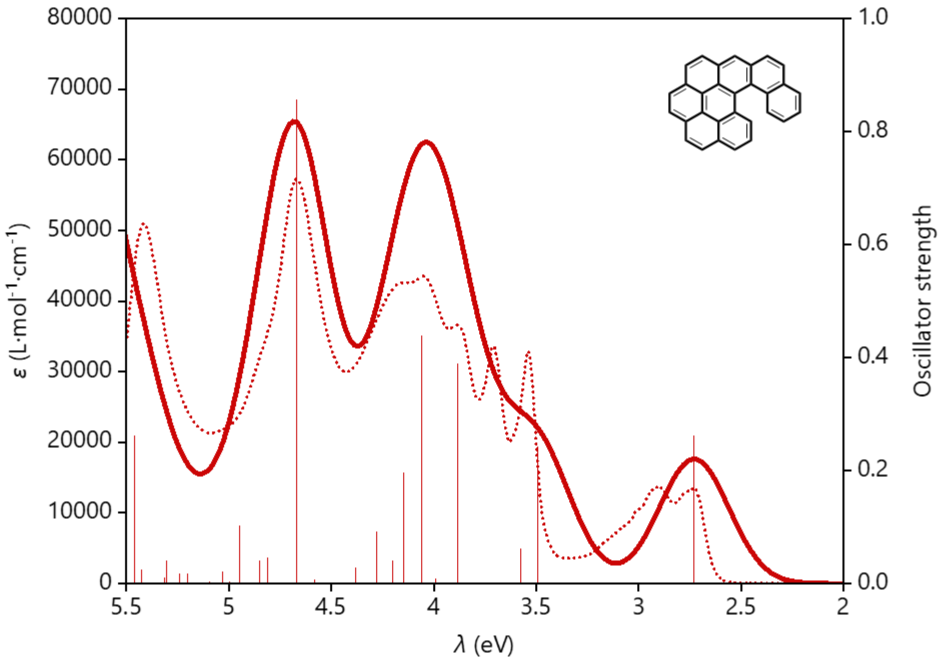

Supplementary Figure 116. Calculated (solid) and experimental UV-vis absorption spectra (dotted, $c = 2 \times 10^{-5}$ mol·L^{-1}, in CH_2Cl_2) of **O7H**. Calculated excitation energies and oscillator strengths *f* are displayed as stick spectrum.

Supplementary Table 29. Selected dominant excitations and occupied (occ) – unoccupied (unocc) MO pair contributions (greater than 10%) of **O7H**.

Excitation	E (eV)	λ (nm)	f	occ no.	unocc no.	%
1	2.73	455	0.2611	98	99	97.8
2	3.05	406	0.0004	97	99	50.1
				98	100	47.6
3	3.49	356	0.2412	97	99	37.8
				98	100	36.0
				96	99	21.7
4	3.57	348	0.0619	98	101	46.3
				96	99	38.1

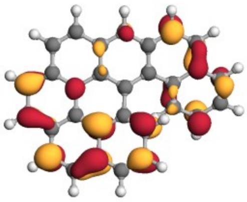

MO101
−0.98 eV

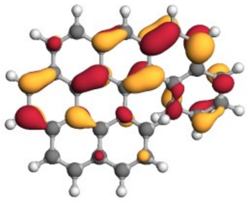

MO100
−1.47 eV

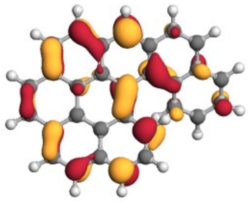

MO99 (LUMO)
−2.18 eV

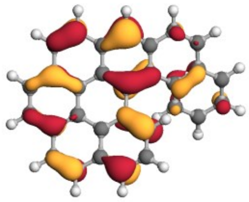

MO98 (HOMO)
−5.50 eV

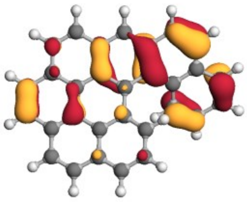

MO97
−6.18 eV

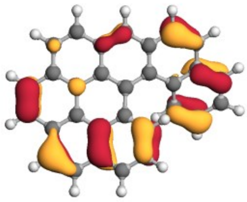

MO96
−6.59 eV

Supplementary Figure 117. Isosurfaces of MOs involved in the selected transitions of **O7H**.

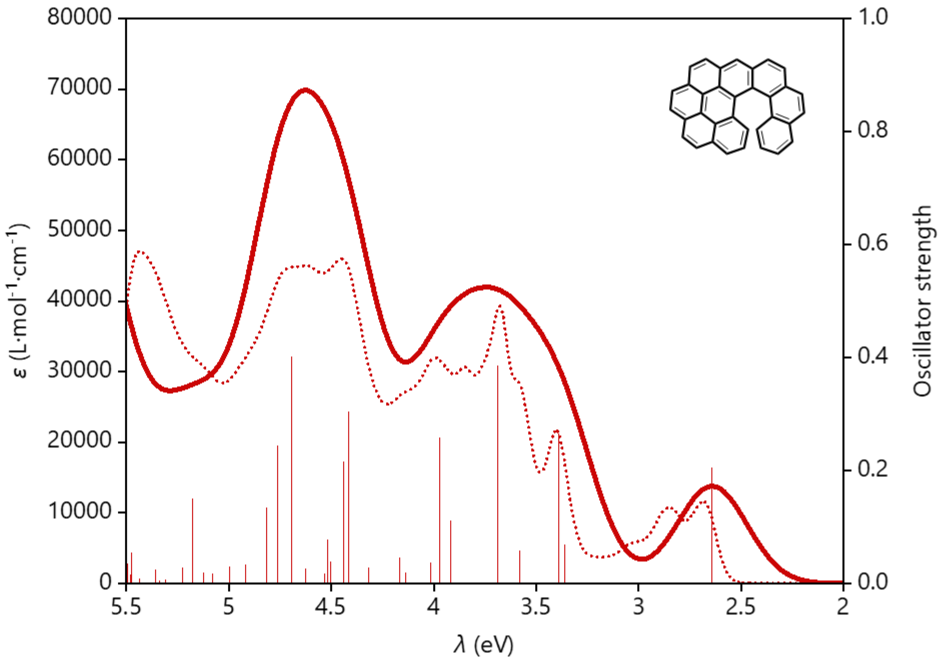

Supplementary Figure 118. Calculated (solid) and experimental UV-vis absorption spectra (dotted, $c = 2 \times 10^{-5}$ mol·L^{-1}, in CH_2Cl_2) of **O8H**. Calculated excitation energies and oscillator strengths f are displayed as stick spectrum.

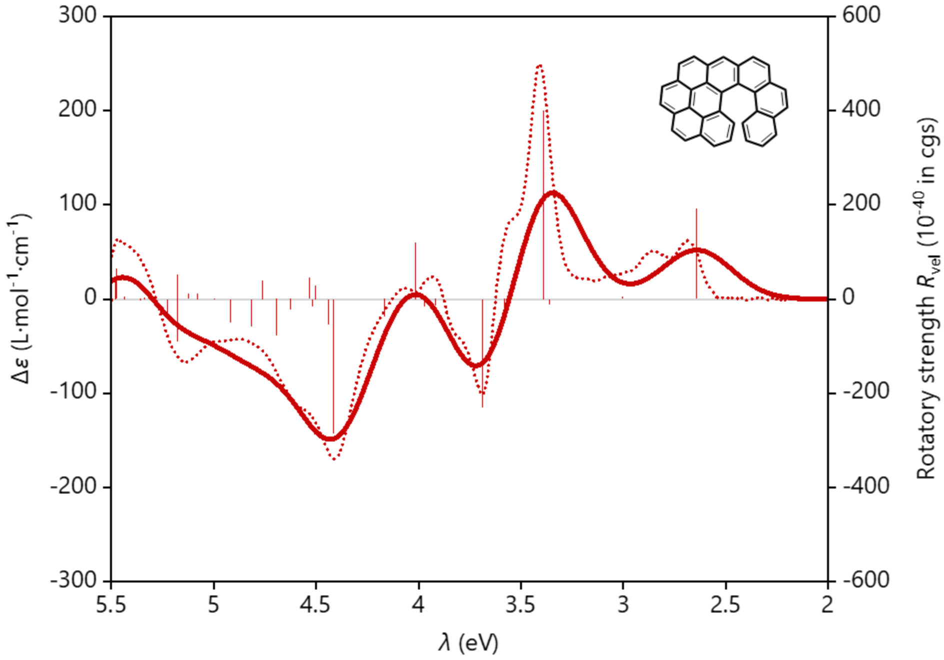

Supplementary Figure 119. Calculated (solid) and experimental ECD spectra (dotted, $c = 2 \times 10^{-5}$ mol·L^{-1}, in CH_2Cl_2) of *P*-**O8H**. Calculated excitation energies and rotatory strengths R_{vel} are displayed as stick spectrum.

Supplementary Table 30. Selected dominant excitations and occupied (occc) – unoccupied (unocc) MO pair contributions (greater than 10%) of *P*-**O8H**.

Excitation	*E* (eV)	*λ* (nm)	*f*	R_{vel} (10^{-40} in cgs)	occ no.	unocc no.	%
1	2.64	470	0.2042	192.4	111	112	97.7
2	3.00	414	0.0018	5.1	111	113	55.0
					110	112	37.7
3	3.35	370	0.0696	−11.1	109	112	70.0
					110	112	14.9
4	3.38	367	0.2661	401.0	111	113	37.2
					110	112	31.5
					109	112	18.2

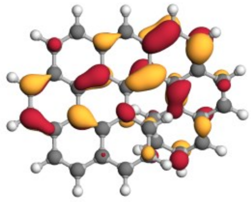

MO113
−1.53 eV

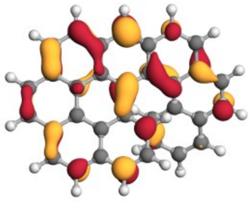

MO112 (LUMO)
−2.23 eV

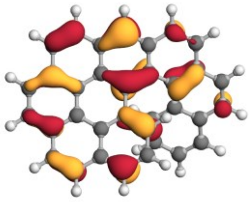

MO111 (HOMO)
−5.46 eV

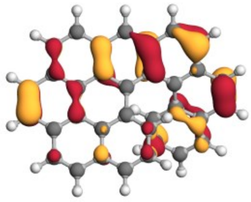

MO110
−6.19 eV

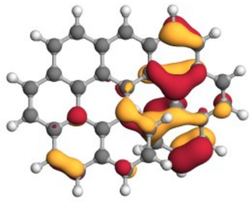

MO109
−6.33 eV

Supplementary Figure 120. Isosurfaces of MOs involved in the selected transitions of **O8H**.

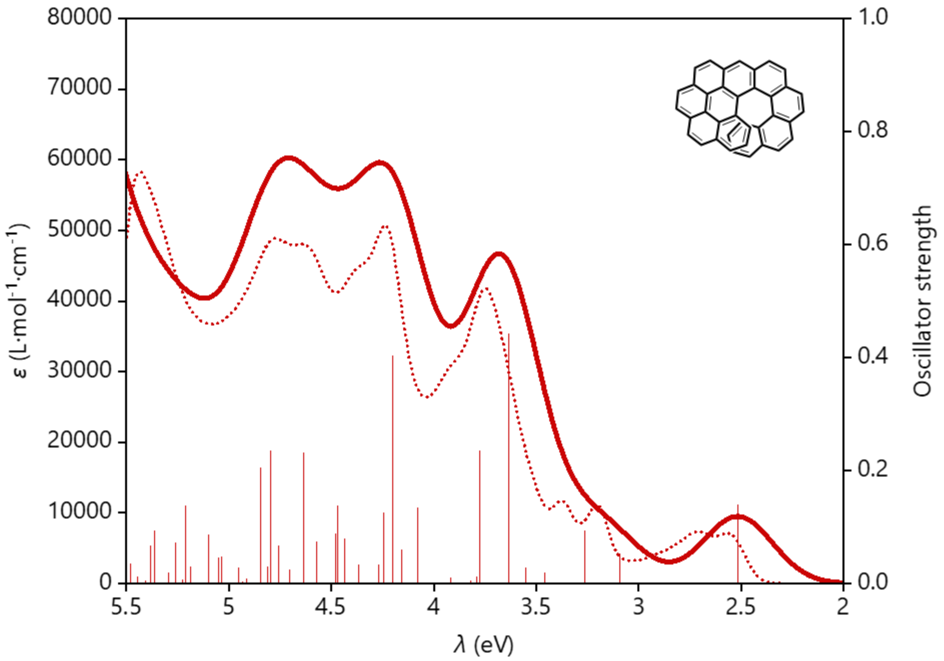

Supplementary Figure 121. Calculated (solid) and experimental UV-vis absorption spectra (dotted, $c = 2 \times 10^{-5}$ mol·L^{-1}, in CH_2Cl_2) of **O9H**. Calculated excitation energies and oscillator strengths *f* are displayed as stick spectrum.

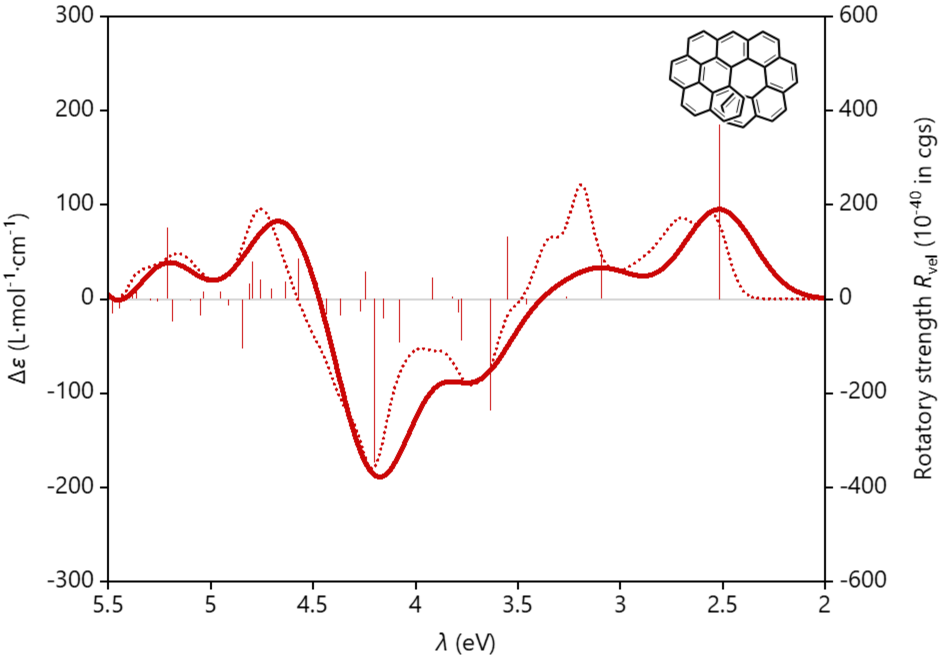

Supplementary Figure 122. Calculated (solid) and experimental ECD spectra (dotted, $c = 2 \times 10^{-5}$ mol·L^{-1}, in CH_2Cl_2) of *P*-**O9H**. Calculated excitation energies and rotatory strengths R_{vel} are displayed as stick spectrum.

Supplementary Table 31. Selected dominant excitations and occupied (occ) – unoccupied (unocc) MO pair contributions (greater than 10%) of *P*-**O9H**.

Excitation	*E* (eV)	*λ* (nm)	*f*	R_{vel} (10^{-40} in cgs)	occ no.	unocc no.	%
1	2.51	493	0.1406	370.5	124	125	96.0
2	2.83	438	0.0006	−0.1	123	125	48.3
					124	126	46.0
3	3.09	402	0.0527	101.3	123	125	45.2
					124	126	39.2
4	3.26	381	0.0928	4.1	122	125	62.5
					124	127	18.1

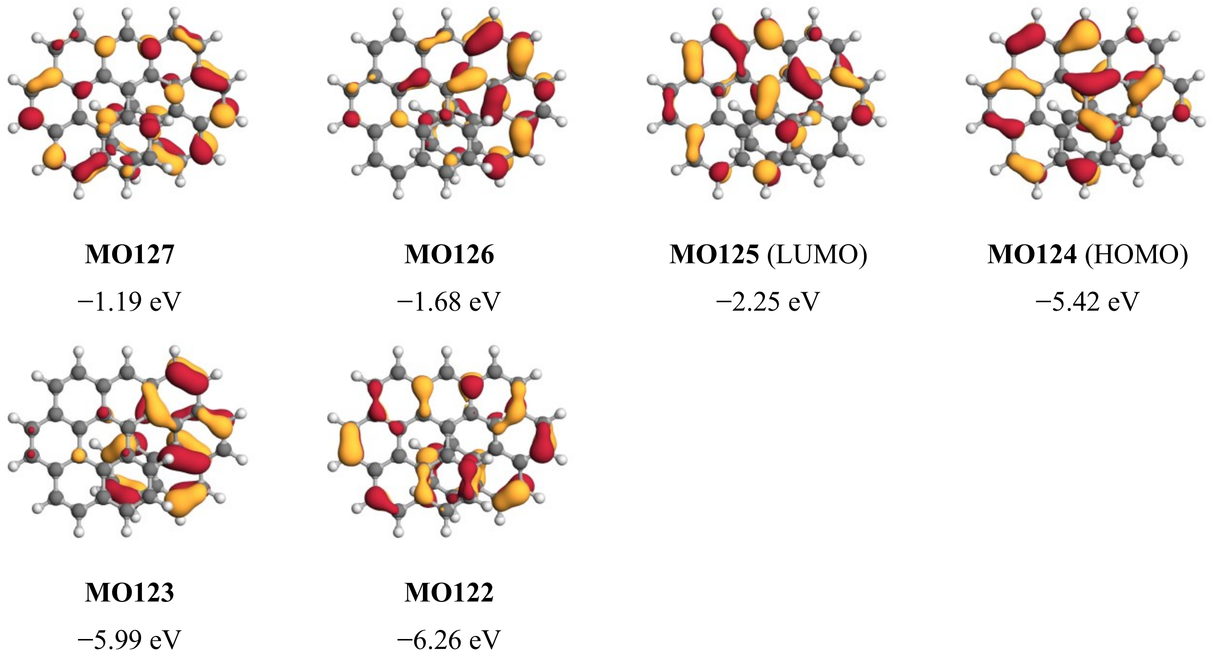

Supplementary Figure 123. Isosurfaces of MOs involved in the selected transitions of **O9H**.

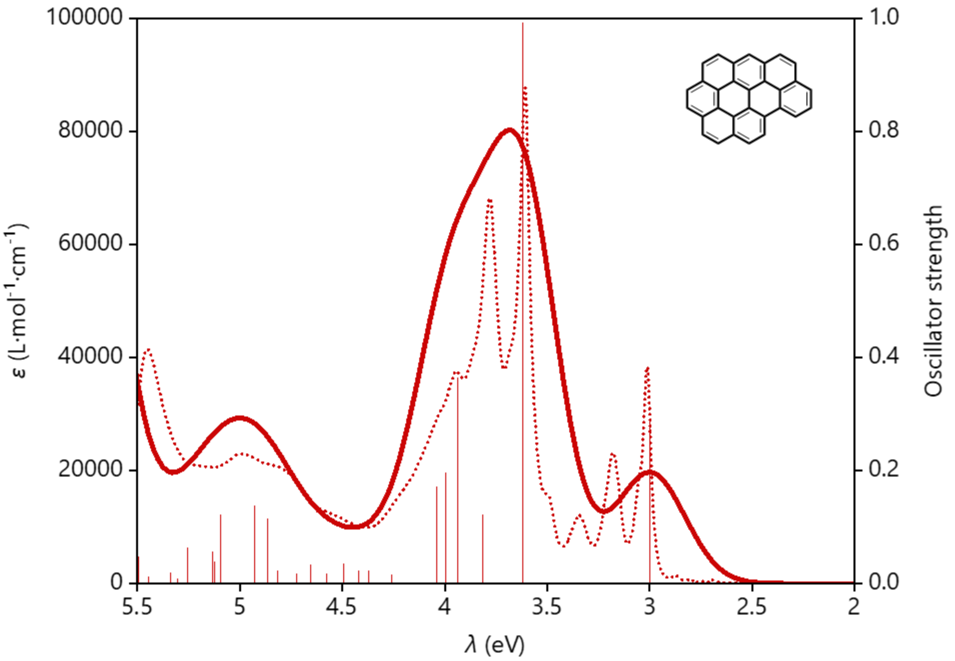

Supplementary Figure 124. Calculated (solid) and experimental UV-vis absorption spectra (dotted, $c = 2 \times 10^{-5}$ mol·L^{-1}, in CH_2Cl_2) of **OO7H**. Calculated excitation energies and oscillator strengths *f* are displayed as stick spectrum.

Supplementary Table 32. Selected dominant excitations and occupied (occ) – unoccupied (unocc) MO pair contributions (greater than 10%) of **OO7H**.

Excitation	*E* (eV)	*λ* (nm)	*f*	occ no.	unocc no.	%
1	2.99	414	0.2896	97	98	92.3
2	3.04	408	0.0011	97	99	51.8
				96	98	46.4
3	3.61	343	0.9930	96	98	46.5
				97	99	42.6

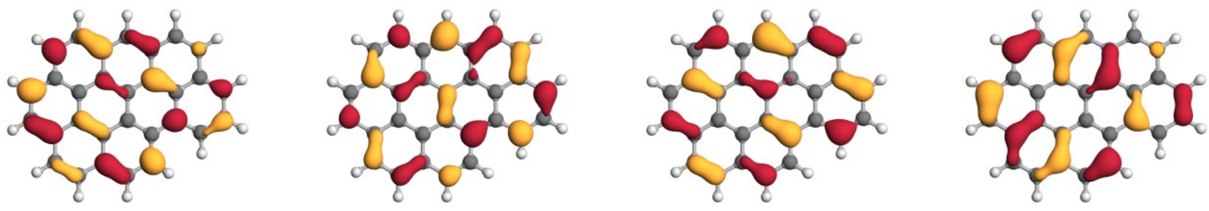

**MO99**	**MO98** (LUMO)	**MO97** (HOMO)	**MO96**
−1.61 eV	−2.08 eV	−5.57 eV	−6.07 eV

Supplementary Figure 125. Isosurfaces of MOs involved in the selected transitions of **OO7H**.

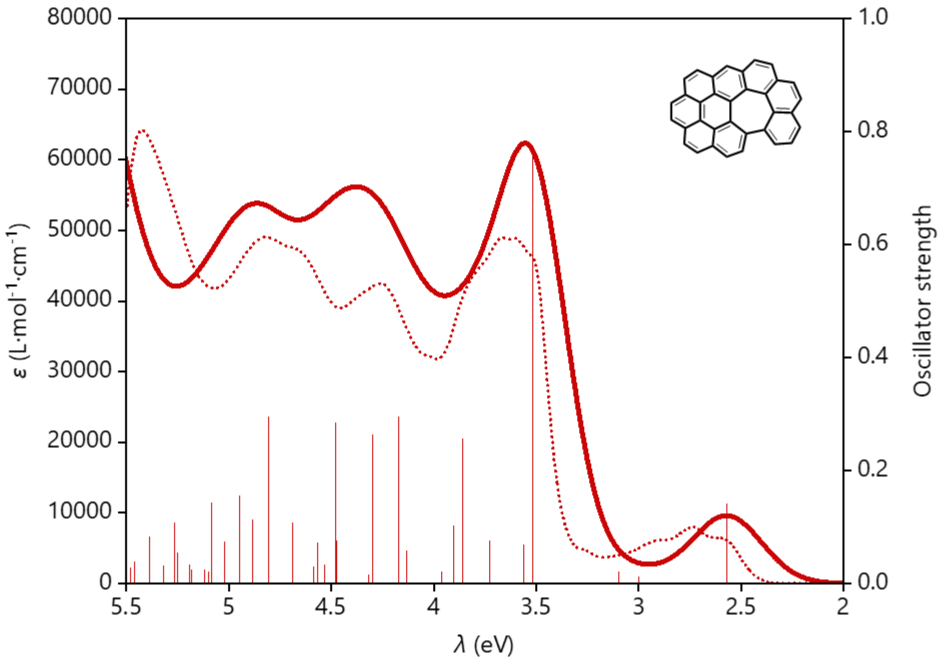

Supplementary Figure 126. Calculated (solid) and experimental UV-vis absorption spectra (dotted, $c = 2 \times 10^{-5}$ mol·L^{-1}, in CH_2Cl_2) of **OO8H**. Calculated excitation energies and oscillator strengths f are displayed as stick spectrum.

Supplementary Table 33. Selected dominant excitations and occupied (occ) – unoccupied (unocc) MO pair contributions (greater than 10%) of **OO8H**.

Excitation	E (eV)	λ (nm)	f	occ no.	unocc no.	%
1	2.57	483	0.1419	110	111	95.2
2	2.99	414	0.0116	110	112	58.0
				110	113	25.2
				109	111	13.3
3	3.09	401	0.0210	110	112	36.7
				109	111	31.6
				110	113	27.9
4	3.51	353	0.7609	109	111	46.2
				110	113	36.1

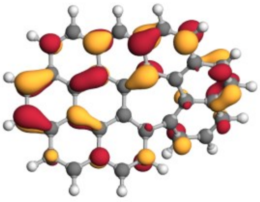

MO113
−1.40 eV

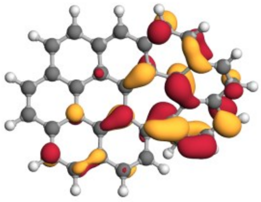

MO112
−1.53 eV

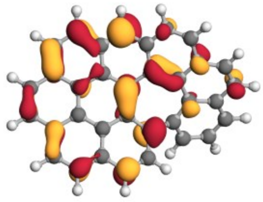

MO111 (LUMO)
−2.16 eV

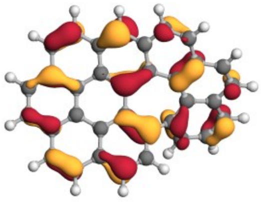

MO110 (HOMO)
−5.34 eV

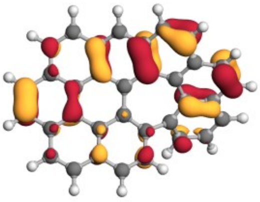

MO109
−6.16 eV

Supplementary Figure 127. Isosurfaces of MOs involved in the selected transitions of **OO8H**.

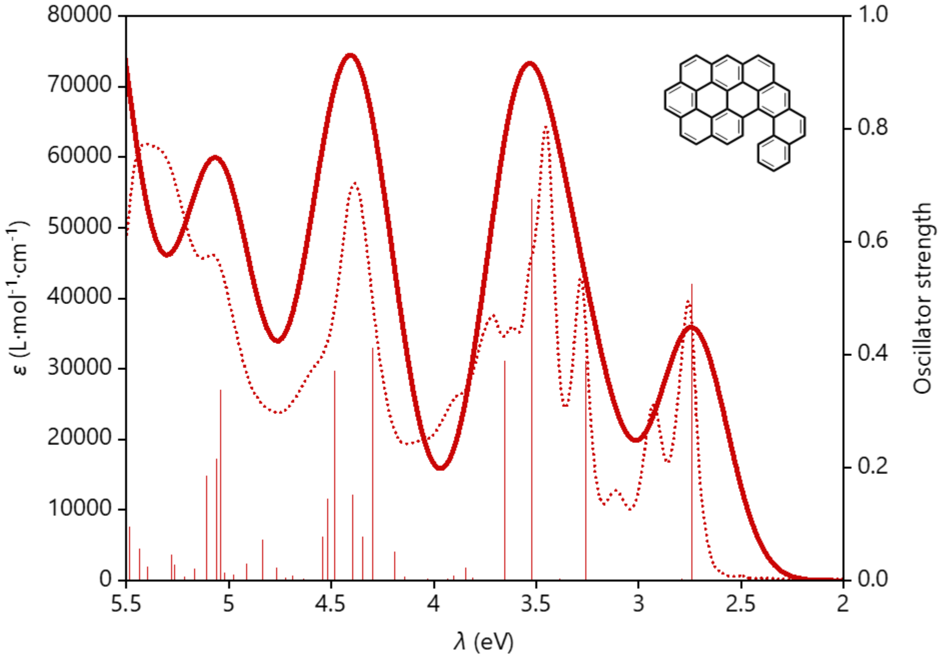

Supplementary Figure 128. Calculated (solid) and experimental UV-vis absorption spectra (dotted, $c = 2 \times 10^{-5}$ mol·L^{-1}, in CH_2Cl_2) of **OO9H**. Calculated excitation energies and oscillator strengths f are displayed as stick spectrum.

Supplementary Table 34. Selected dominant excitations and occupied (occ) – unoccupied (unocc) MO pair contributions (greater than 10%) of **OO9H**.

Excitation	E (eV)	λ (nm)	f	occ no.	unocc no.	%
1	2.73	453	0.5254	123	124	93.0
2	2.78	446	0.0030	122	124	49.5
				123	125	48.4
3	3.25	382	0.3886	123	125	45.8
				122	124	45.5
4	3.38	367	0.0036	121	124	56.4
				123	126	35.1
5	3.51	353	0.6757	122	125	76.7
6	3.65	340	0.3889	123	126	42.5
				121	124	30.1
				122	125	12.5

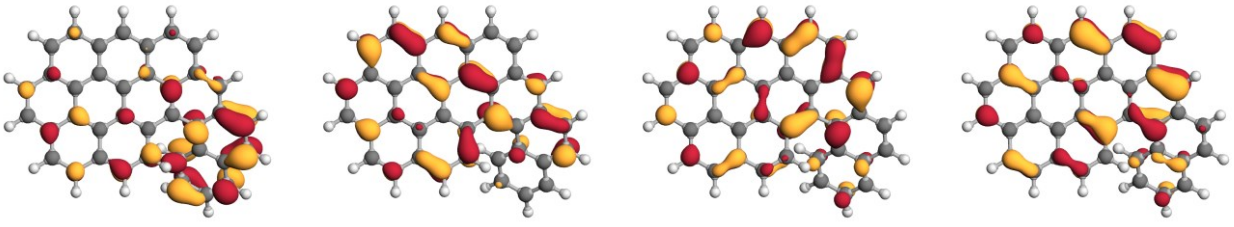

MO126 −1.24 eV

MO125 −1.82 eV

MO124 (LUMO) −2.24 eV

MO123 (HOMO) −5.49 eV

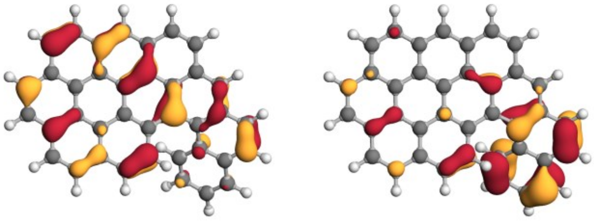

MO122 −5.90 eV

MO121 −6.42 eV

Supplementary Figure 129. Isosurfaces of MOs involved in the selected transitions of **OO9H**.

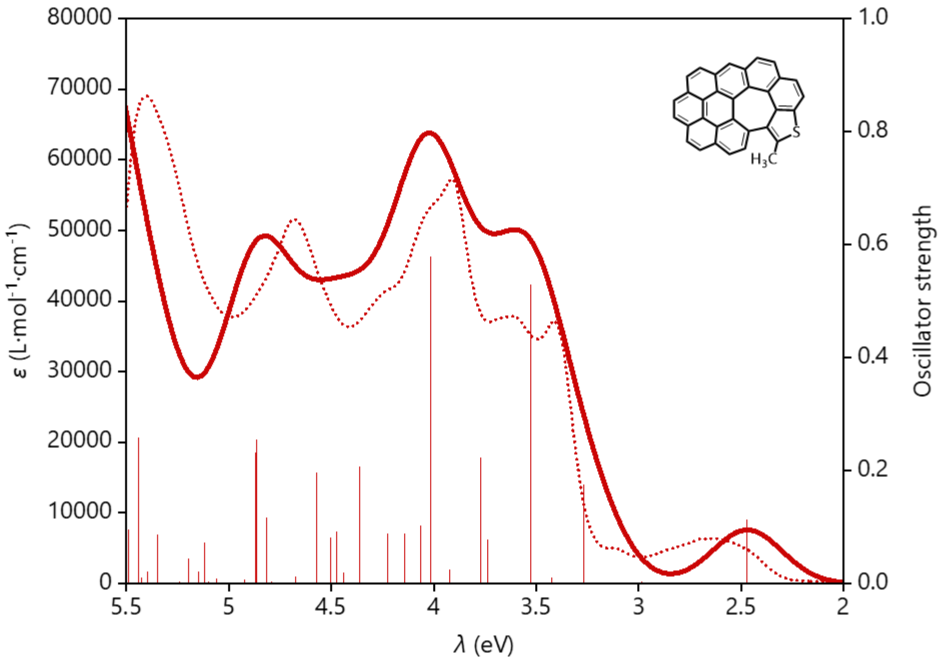

Supplementary Figure 130. Calculated (solid) and experimental UV-vis absorption spectra (dotted, $c = 2 \times 10^{-5}$ mol·L^{-1}, in CH_2Cl_2) of **OO8H$_{mt}$**. Calculated excitation energies and oscillator strengths f are displayed as stick spectrum.

Supplementary Table 35. Selected dominant excitations and occupied (occ) – unoccupied (unocc) MO pair contributions (greater than 10%) of **OO8H$_{mt}$**.

Excitation	E (eV)	λ (nm)	f	occ no.	unocc no.	%
1	2.47	503	0.1125	115	116	95.8
2	2.98	416	0.0035	114	116	46.2
				115	117	45.8
3	3.26	380	0.1750	114	116	43.9
				115	117	25.7
				113	116	22.0
4	3.42	363	0.0104	115	118	79.3
5	3.52	352	0.5292	113	116	35.6
				115	117	20.8
				115	119	20.4
				115	118	11.2

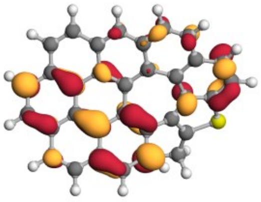

MO119
−0.81 eV

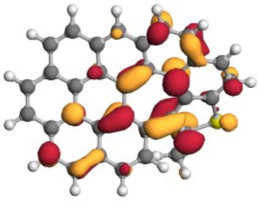

MO118
−1.14 eV

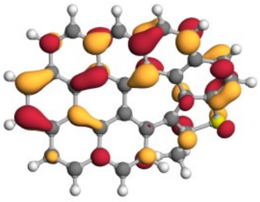

MO117
−1.44 eV

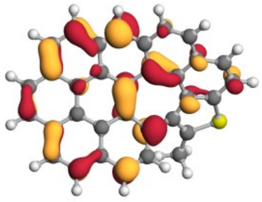

MO116 (LUMO)
−2.18 eV

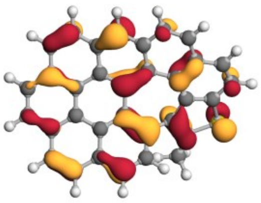

MO115 (HOMO)
−5.31 eV

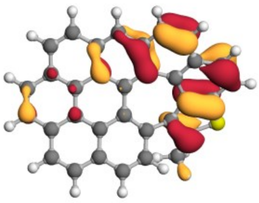

MO114
−6.03 eV

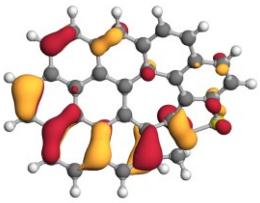

MO113
−6.35 eV

Supplementary Figure 131. Isosurfaces of MOs involved in the selected transitions of **OO8H$_{\mathbf{mt}}$**.

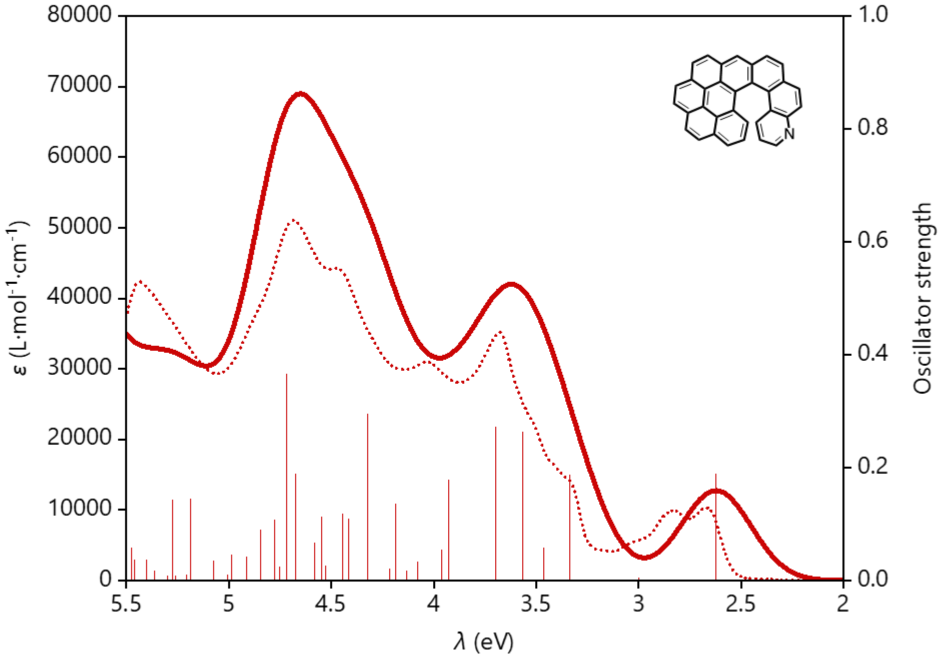

Supplementary Figure 132. Calculated (solid) and experimental UV-vis absorption spectra (dotted, c = 2 × 10^{-5} mol·L^{-1}, in CH_2Cl_2) of **O8H$_{\mathbf{a}}$**. Calculated excitation energies and oscillator strengths f are displayed as stick spectrum.

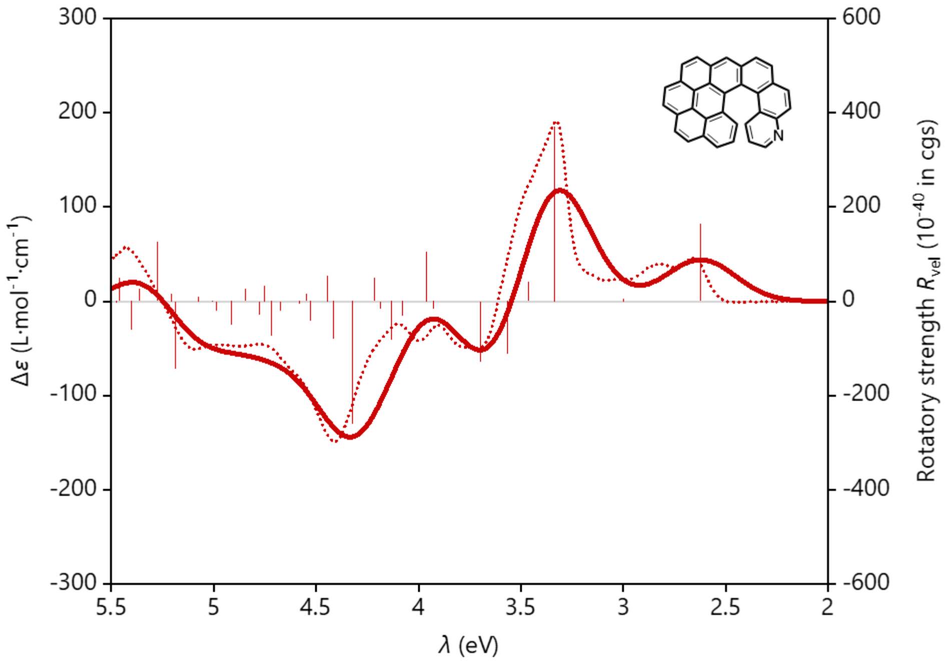

Supplementary Figure 133. Calculated (solid) and experimental ECD spectra (dotted, $c = 2 \times 10^{-5}$ mol·L^{-1} in CH_2Cl_2) of *P*-**O8H**$_\mathbf{a}$. Calculated excitation energies and rotatory strengths R_{vel} are displayed as stick spectrum.

Supplementary Table 36. Selected dominant excitations and occupied (occ) – unoccupied (unocc) MO pair contributions (greater than 10%) of *P*-**O8H**$_\mathbf{a}$.

Excitation	*E* (eV)	*λ* (nm)	*f*	R_{vel} (10^{-40} in cgs)	occ no.	unocc no.	%
1	2.62	474	0.1886	164.1	111	112	97.6
2	3.00	414	0.0051	4.1	111	113	54.4
					110	112	40.3
3	3.33	372	0.1871	370.3	111	113	38.6
					110	112	34.8
					111	114	22.0

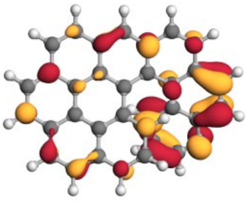

MO114

−1.37 eV

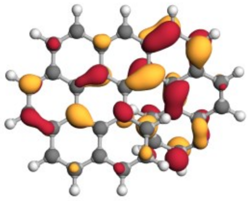

MO113

−1.61 eV

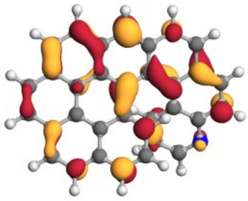

MO112 (LUMO)

−2.30 eV

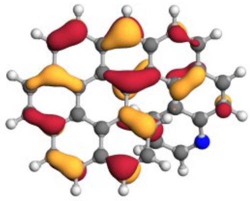

MO111 (HOMO)

−5.52 eV

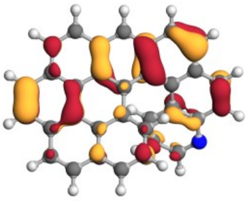

MO110

−6.27 eV

Supplementary Figure 134. Isosurfaces of MOs involved in the selected transitions of **O8H**$_\mathbf{a}$.

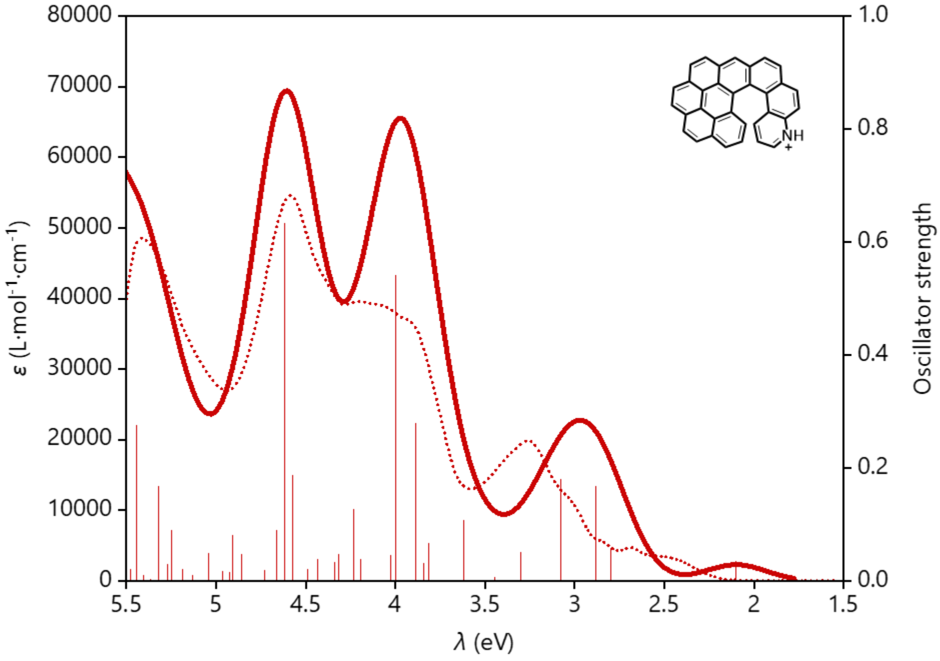

Supplementary Figure 135. Calculated (solid) and experimental UV-vis absorption spectra (dotted, c = 2×10^{-5} mol·L^{-1}, in CH_2Cl_2) of **O8H**$_\mathbf{a}$H$^+$. Calculated excitation energies and oscillator strengths f are displayed as stick spectrum.

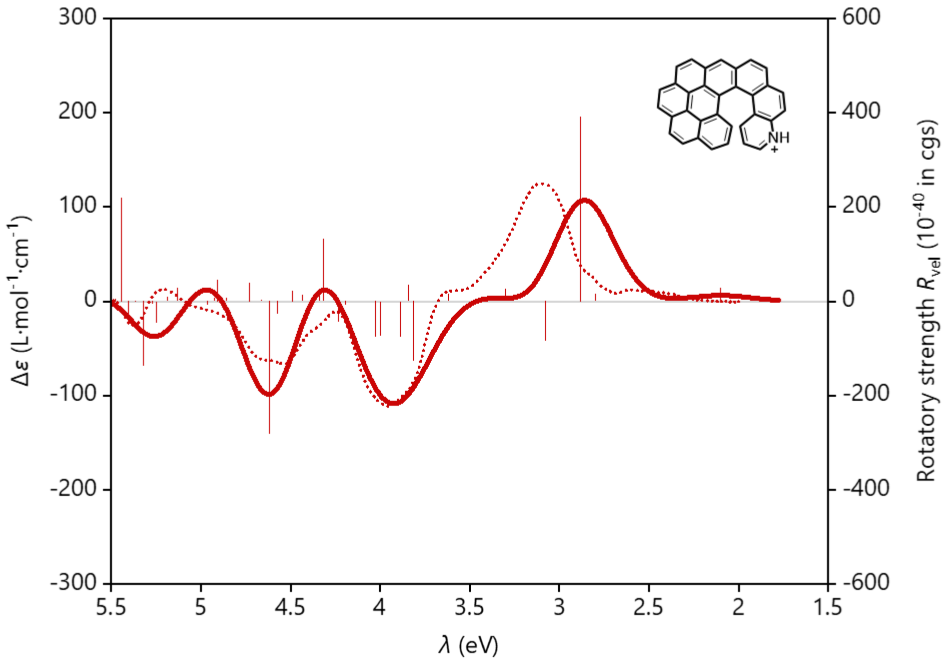

Supplementary Figure 136. Calculated (solid) and experimental ECD spectra (dotted, c = 2 × 10^{-5} mol·L^{-1}, in CH_2Cl_2) of *P*-**O8H**$_{\mathbf{a}}$$H^+$. Calculated excitation energies and rotatory strengths R_{vel} are displayed as stick spectrum.

Supplementary Table 37. Selected dominant excitations and occupied (occ) – unoccupied (unocc) MO pair contributions (greater than 10%) of *P*-**O8H**$_{\mathbf{a}}$$H^+$.

Excitation	*E* (eV)	*λ* (nm)	*f*	R_{vel} (10^{-40} in cgs)	occ no.	unocc no.	%
1	2.10	591	0.0342	29.0	111	112	98.4
2	2.79	444	0.0579	16.6	111	113	43.2
					110	112	30.9
					111	114	21.0
3	2.88	431	0.1673	391.4	111	113	49.1
					110	112	45.3

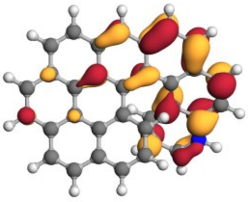

MO114
−2.15 eV

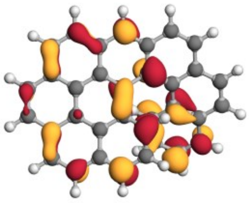

MO113
−2.41 eV

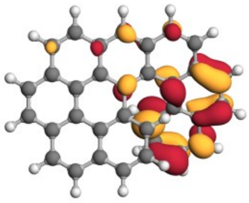

MO112 (LUMO)
−3.10 eV

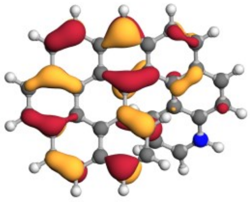

MO111 (HOMO)
−5.89 eV

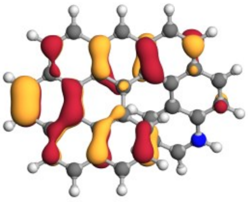

MO110
−6.68 eV

Supplementary Figure 137. Isosurfaces of MOs involved in the selected transitions of **O8H$_{\mathbf{a}}$**H$^+$.

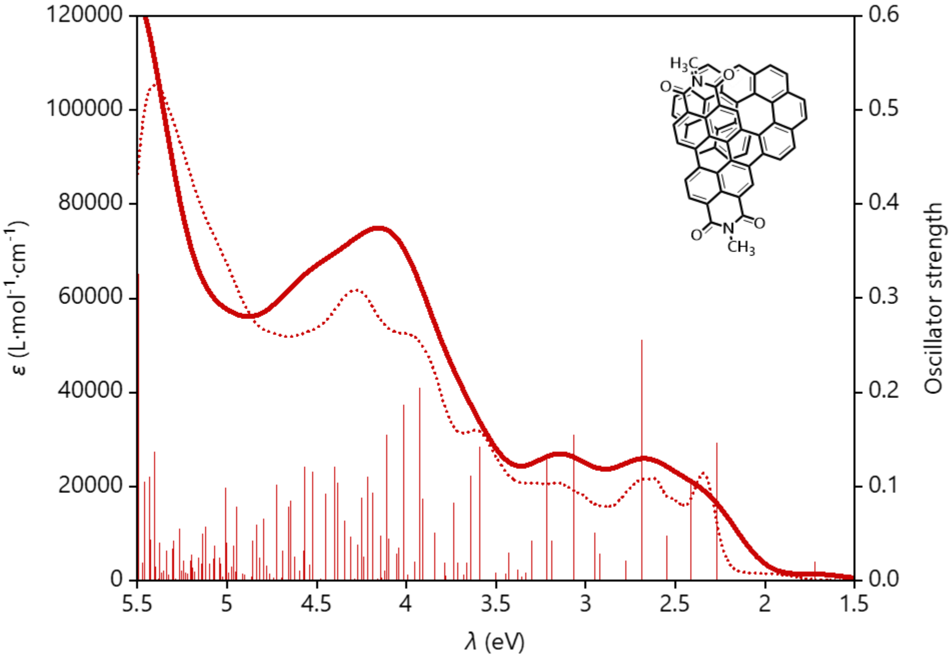

Supplementary Figure 138. Calculated (solid) UV-vis absorption spectra of **CO8H'$_{\mathbf{PDI\text{-}CH3}}$** (simplified model of **CO8H'$_{\mathbf{PDI}}$** using methyl groups to replace the long alkyl chains) and experimental UV-vis absorption spectra of **CO8H'$_{\mathbf{PDI}}$** (dotted, c = 2 × 10^{-5} mol·L^{-1}, in CH_2Cl_2). Calculated excitation energies and oscillator strengths f are displayed as stick spectrum.

Supplementary Table 38. Selected dominant excitations and occupied (occ) – unoccupied (unocc) MO pair contributions (greater than 10%) of **CO8H'$_{\mathbf{PDI\text{-}CH3}}$**.

Excitation	*E* (eV)	λ (nm)	*f*	occ no.	unocc no.	%
1	1.72	721	0.0196	217	218	99.1
2	2.26	548	0.1466	216	218	91.1
3	2.41	515	0.1017	215	218	89.7
4	2.54	488	0.0481	217	219	91.9
5	2.68	462	0.2555	214	218	86.8

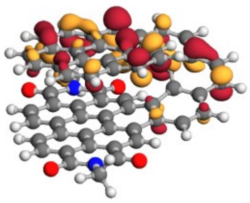

MO219
−2.30 eV

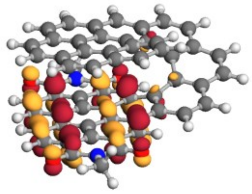

MO218 (LUMO)
−3.12 eV

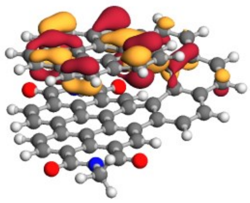

MO217 (HOMO)
−5.49 eV

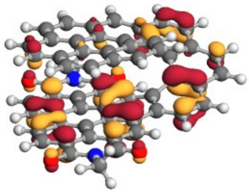

MO216
−5.96 eV

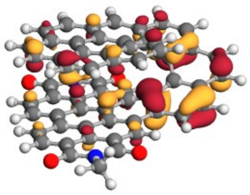

MO215
−6.19 eV

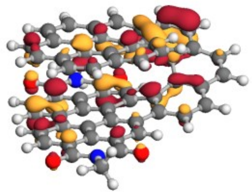

MO214
−6.38 eV

Supplementary Figure 139. Isosurfaces of MOs involved in the selected transitions of **CO8H'$_{\mathbf{PDI\text{-}CH3}}$**.

Supplementary References

1. Jakubec, M., et al. 2-Bromo[6]helicene as a key intermediate for [6]helicene functionalization. *J. Org. Chem.* **83**, 3607-3616 (2018).
2. Shen, C., et al. Helicene-derived aggregation-induced emission conjugates with highly tunable circularly polarized luminescence. *Mater. Chem. Front.* **4**, 837-844 (2020).
3. Rajasingh, P., Cohen, R., Shirman, E., Shimon, L. J. W. & Rybtchinski, B. Selective bromination of perylene diimides under mild conditions. *J. Org. Chem.* **72**, 5973-5979 (2007).
4. Nakai, Y., Mori, T. & Inoue, Y. Theoretical and experimental studies on circular dichroism of carbo[*n*]helicenes. *J. Phys. Chem. A* **116**, 7372-7385 (2012).
5. Frisch, M. J., et al. Gaussian 09 Rev. E.01. (Wallingford, CT, 2013).
6. Adamo, C. & Barone, V. Toward reliable density functional methods without adjustable parameters: The PBE0 model. *J. Chem. Phys.* **110**, 6158-6170 (1999).
7. Weigend, F. & Ahlrichs, R. Balanced basis sets of split valence, triple zeta valence and quadruple zeta valence quality for H to Rn: Design and assessment of accuracy. *Phys. Chem. Chem. Phys.* **7**, 3297-3305 (2005).
8. Marenich, A. V., Cramer, C. J. & Truhlar, D. G. Universal solvation model based on solute electron density and on a continuum model of the solvent defined by the bulk dielectric constant and atomic surface tensions. *J. Phys. Chem. B* **113**, 6378-6396 (2009).
9. Chen, Z., Wannere, C. S., Corminboeuf, C., Puchta, R. & Schleyer, P. v. R. Nucleus-independent chemical shifts (NICS) as an aromaticity criterion. *Chem. Rev.* **105**, 3842-3888 (2005).
10. Krygowski, T. M., Szatylowicz, H., Stasyuk, O. A., Dominikowska, J. & Palusiak, M. Aromaticity from the viewpoint of molecular geometry: application to planar systems. *Chem. Rev.* **114**, 6383-6422 (2014).
11. Martin, R. H., Jespers, J. & Defay, N. 1-hydroxymethyl [6] helicene: acid catalysed intramolecular rearrangement involving the helicene skeleton. *Tetrahedron Lett.* **16**, 1093-1096 (1975).
12. Herz, W. & Caple, G. Migratory aptitudes of unsaturated groups. *J. Org. Chem.* **29**, 1691-1699 (1964).
13. Zheng, J., Xu, X. & Truhlar, D. G. Minimally augmented Karlsruhe basis sets. *Theor. Chem. Acc.* **128**, 295-305 (2011).
