# Supplementary material for: Oxidative cyclo-rearrangement of helicenes into chiral nanographenes
Source: Nat Commun. 2021 May 13;12:2786. doi: 10.1038/s41467-021-22992-6 (PMC8119938; doi:10.1038/s41467-021-22992-6)
Supplement: Supplementary file 3 — Description of Additional Supplementary Files [file 41467_2021_22992_MOESM3_ESM.pdf]

Description of Additional Supplementary Files

File Name: Supplementary Data 1
Description: Cartesian coordinates of the optimized geometries
