# Supplementary material for: Oxidative cyclo-rearrangement of helicenes into chiral nanographenes
Source: Nat Commun. 2021 May 13;12:2786. doi: 10.1038/s41467-021-22992-6 (PMC8119938; doi:10.1038/s41467-021-22992-6)
Supplement: Supplementary file 4 — Supplementary Data 1 [file 41467_2021_22992_MOESM4_ESM.zip › NCOMMS-20-49707A_Supplementary_Data_1.pdf]

Cartesian coordinates of the optimized geometries

CF_3SO_3H

C	−0.998032	0.003262	−0.003433	F	−1.520253	−0.941620	0.751668	S	0.852733	−0.135501	0.069995
F	−1.406938	−0.139560	−1.246342	O	1.232431	0.076251	1.448728	O	1.247619	1.131192	−0.818057
F	−1.358812	1.189859	0.451816	O	1.224119	−1.303710	−0.686323	H	1.285136	1.940466	−0.268373

$CF_3SO_3^-$

C	−0.941245	0.000025	−0.000149	F	−1.426521	−0.199446	1.227680	S	0.913326	0.000060	0.000016
F	−1.427593	−0.963330	−0.786310	O	1.231828	1.109742	0.911674	O	1.232795	0.234872	−1.416664
F	−1.427969	1.162503	−0.441163	O	1.232003	−1.344445	0.504838				

CF_3SO_3H

ma-SVP was used as the basis set.

C	1.009656	0.005370	−0.003501	F	1.526649	−0.995359	0.679935	O	−1.240677	0.000253	1.452966
F	1.413369	−0.046774	−1.255047	S	−0.856634	−0.136696	0.062574	O	−1.259759	1.170373	−0.762687
F	1.371428	1.153974	0.537934	O	−1.228919	−1.271950	−0.747985	H	−1.319960	1.958946	−0.183935

$CF_3SO_3^-$

ma-SVP was used as the basis set.

C	−0.955609	0.000301	−0.000168	F	−1.434012	−0.792918	−0.959340	O	1.241201	−1.416504	0.251885
F	−1.433025	−0.434148	1.166558	S	0.913730	−0.000098	−0.000006	O	1.243280	0.489552	−1.352290
F	−1.433047	1.227900	−0.207278	O	1.242360	0.925984	1.100612				

DDQ

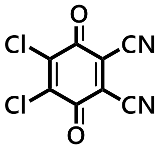

C	−1.149825	−0.677164	0.000000	C	0.117045	−1.458471	0.000000	C	2.596996	−1.449229	0.000000
C	−1.149825	0.677164	0.000000	O	0.135632	−2.663619	0.000000	N	3.567985	−2.079705	0.000000
C	0.117045	1.458471	0.000000	O	0.135632	2.663619	0.000001	C	2.596996	1.449229	−0.000001
C	1.397112	0.677752	0.000000	Cl	−2.578171	−1.600379	0.000000	N	3.567985	2.079705	−0.000001
C	1.397112	−0.677752	0.000000	Cl	−2.578171	1.600379	0.000000				

$DDQH^{\cdot}$

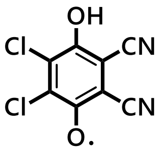

C	1.109440	0.639762	0.000000	C	−0.112373	1.373164	−0.000001	C	−2.553865	1.443325	0.000001
C	1.109515	−0.736293	0.000000	O	−0.151761	2.684181	−0.000001	N	−3.534591	2.059355	0.000000
C	−0.142173	−1.505909	0.000000	O	−0.157016	−2.734076	0.000001	C	−2.596863	−1.437607	0.000001
C	−1.372491	−0.715064	0.000000	Cl	2.567795	1.552163	0.000001	N	−3.593593	−2.030041	−0.000001
C	−1.349119	0.677521	0.000000	Cl	2.553977	−1.629167	−0.000001	H	0.744961	3.069630	0.000001

DDQH$_2$

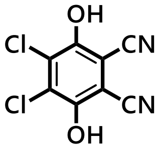

C	−1.075584	−0.696744	−0.000003	O	0.180380	−2.752488	−0.000020	N	3.559484	2.023220	−0.000004
C	−1.075620	0.696685	0.000002	Cl	−2.549848	−1.583509	−0.000003	H	−0.714327	−3.131814	0.000078
C	0.130279	1.424841	−0.000002	Cl	−2.549893	1.583448	0.000009	O	0.180223	2.752472	−0.000002
C	1.334602	0.707634	−0.000005	C	2.561267	−1.434322	0.000007	H	−0.714526	3.131707	−0.000003
C	1.334635	−0.707576	0.000000	N	3.559515	−2.023161	0.000021				
C	0.130343	−1.424846	−0.000005	C	2.561183	1.434471	−0.000010				

DDQ

ma-SVP was used as the basis set.

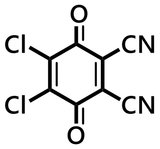

C	0.000000	0.677111	−1.151357	C	0.000000	1.456691	0.117569	C	0.000000	1.448009	2.600176
C	0.000000	−0.677111	−1.151357	O	0.000000	2.662069	0.135947	N	0.000000	2.073770	3.573883
C	0.000000	−1.456691	0.117569	O	0.000000	−2.662069	0.135947	C	0.000000	−1.448009	2.600176
C	0.000000	−0.677402	1.399041	Cl	0.000000	1.598630	−2.582196	N	0.000000	−2.073770	3.573883
C	0.000000	0.677402	1.399041	Cl	0.000000	−1.598630	−2.582196				

DDQH$^{\cdot}$

ma-SVP was used as the basis set.

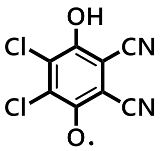

C	1.110195	0.640984	0.000000	C	−0.113112	1.371609	−0.000001	C	−2.555757	1.439185	0.000000
C	1.111528	−0.735491	0.000000	O	−0.159802	2.682640	0.000000	N	−3.537761	2.052660	0.000000
C	−0.140496	−1.504418	−0.000002	O	−0.154425	−2.733312	0.000001	C	−2.598054	−1.438652	0.000001
C	−1.372757	−0.716439	−0.000001	Cl	2.568539	1.553372	0.000001	N	−3.595793	−2.028852	0.000000
C	−1.349689	0.674800	−0.000001	Cl	2.559133	−1.626260	0.000000	H	0.727125	3.088351	0.000001

DDQH_2

ma-SVP was used as the basis set.

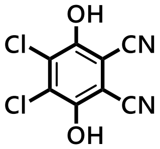

C	1.076471	0.697196	0.000000	O	−0.185518	2.752112	0.000000	N	−3.559011	−2.022571	−0.000012
C	1.076467	−0.697200	0.000000	Cl	2.552188	1.582183	0.000001	H	0.699614	3.151588	0.000046
C	−0.129397	−1.423402	−0.000003	Cl	2.552179	−1.582195	0.000000	O	−0.185538	−2.752107	−0.000005
C	−1.334152	−0.706946	−0.000003	C	−2.561155	1.433423	0.000007	H	0.699591	−3.151590	−0.000007
C	−1.334147	0.706958	0.000002	N	−3.559017	2.022567	0.000009				
C	−0.129388	1.423406	0.000003	C	−2.561165	−1.433402	−0.000005				

[5]helicene **5H**

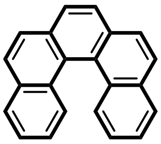

C	−2.953272	−0.307097	−0.179956	C	1.367958	2.024312	0.322886	H	−3.966708	−3.420069	0.828782
C	−1.576667	−0.363067	0.194908	C	0.721492	0.789193	0.051609	H	−4.828556	−1.377126	−0.298017
C	−1.145565	−1.508195	0.911590	C	2.753262	2.050231	0.678769	H	−4.554767	0.934673	−0.977667
C	−1.986126	−2.578571	1.140566	C	3.503840	0.914139	0.677191	H	−3.195035	3.010910	−0.957003
C	−3.311275	−2.561266	0.662916	C	2.953272	−0.307097	0.179956	H	−1.181825	4.186024	−0.390071
C	−3.786096	−1.433984	0.027587	C	1.576667	−0.363067	−0.194908	H	1.181825	4.186024	0.390071
C	−3.503840	0.914139	−0.677191	C	3.786096	−1.433984	−0.027587	H	3.195035	3.010910	0.957003
C	−2.753262	2.050231	−0.678769	C	3.311275	−2.561266	−0.662916	H	4.554767	0.934673	0.977667
C	−1.367958	2.024312	−0.322886	C	1.986126	−2.578571	−1.140566	H	4.828556	−1.377126	0.298017
C	−0.721492	0.789193	−0.051609	C	1.145565	−1.508195	−0.911590	H	3.966708	−3.420069	−0.828782
C	−0.651315	3.248315	−0.204922	H	−0.132516	−1.538678	1.312112	H	1.620248	−3.439796	−1.705510
C	0.651315	3.248315	0.204922	H	−1.620248	−3.439796	1.705510	H	0.132516	−1.538678	−1.312112

[6]helicene **6H**

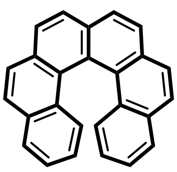

C	−1.289324	0.785335	−0.037588	C	−2.297479	2.912287	−0.765671	C	0.000000	1.445998	0.000000
C	−1.552461	−0.533097	0.514999	C	−2.792939	−1.177698	0.226948	C	0.000000	2.871202	0.000000
C	−1.166407	3.583865	−0.400051	C	−3.804442	−0.475587	−0.499221	C	2.395467	1.507096	0.554409
C	−3.631568	0.836602	−0.819515	C	1.289324	0.785335	0.037588	C	2.297479	2.912287	0.765671
C	−3.039065	−2.482031	0.719832	C	1.552461	−0.533097	−0.514999	C	2.792939	−1.177698	−0.226948
C	−0.943869	−2.447525	1.902885	C	1.166407	3.583865	0.400051	C	3.804442	−0.475588	0.499221
C	−0.667676	−1.190363	1.404544	C	3.631568	0.836601	0.819515	C	2.125142	−3.118582	−1.532811
C	−2.395466	1.507096	−0.554409	C	0.667676	−1.190363	−1.404544	C	−2.125143	−3.118582	1.532811

C	3.039064	−2.482031	−0.719832	H	0.243501	−0.685892	1.724756	H	3.170114	3.447353	1.149361
C	0.943869	−2.447525	−1.902885	H	−3.170113	3.447354	−1.149361	H	4.741375	−0.990385	0.728199
H	−1.121683	4.675342	−0.443481	H	−4.741375	−0.990384	−0.728200	H	2.329508	−4.122443	−1.913939
H	−4.436944	1.407567	−1.289475	H	1.121683	4.675341	0.443481	H	−2.329509	−4.122442	1.913939
H	−3.985837	−2.965439	0.462889	H	4.436944	1.407566	1.289474	H	3.985837	−2.965439	−0.462889
H	−0.240474	−2.919513	2.593644	H	−0.243501	−0.685892	−1.724756	H	0.240474	−2.919513	−2.593643

[7]helicene **7H**

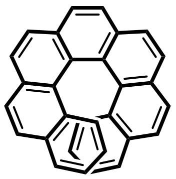

C	0.687162	−1.103227	1.065665	C	−2.865083	2.779036	0.542567	H	3.955823	−2.011970	1.634082
C	0.667349	0.026503	1.920089	C	−2.858364	−1.388720	−0.228272	H	2.812525	−3.723137	0.336821
C	0.724804	−3.660961	−0.128725	C	−4.083455	−0.663113	−0.161280	H	1.743104	1.311330	3.254182
C	3.040071	−1.418372	1.704333	C	0.687236	1.103187	−1.065670	H	3.904498	0.074502	2.986181
C	1.884212	−1.878446	1.027900	C	0.667352	−0.026524	−1.920118	H	−1.683537	4.528282	0.911596
C	1.884179	−3.146636	0.368838	C	0.725038	3.660901	0.128756	H	−5.020309	1.204432	0.317752
C	1.796058	0.436815	2.600671	C	3.040168	1.418192	−1.704325	H	−3.819570	−3.255417	−0.781877
C	3.007657	−0.268023	2.463679	C	−0.469476	1.547909	−0.309930	H	−3.819365	3.255643	0.781880
C	−1.625554	0.725488	−0.025066	C	−0.480685	2.891467	0.139620	H	−5.020385	−1.204125	−0.317768
C	−1.625601	−0.725395	0.025057	C	1.884336	1.878328	−1.027889	H	−0.264360	−0.571533	−2.066353
C	−1.696344	3.483295	0.590985	C	1.884382	3.146509	−0.368808	H	0.696284	4.670829	0.546770
C	−4.083413	0.663361	0.161264	C	1.796038	−0.436897	−2.600703	H	3.955958	2.011730	−1.634061
C	−2.865258	−2.778868	−0.542565	C	3.007682	0.267859	−2.463693	H	2.812765	3.722949	−0.336779
C	−0.480870	−2.891450	−0.139605	C	−1.696565	−3.483203	−0.590970	H	1.743028	−1.311395	−3.254232
C	−0.469577	−1.547887	0.309930	H	−0.264329	0.571577	2.066307	H	3.904502	−0.074715	−2.986198
C	−2.858276	1.388890	0.228263	H	0.695987	−4.670892	−0.546726	H	−1.683824	−4.528195	−0.911570

[8]helicene **8H**

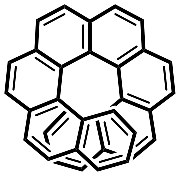

C	−2.786703	−1.096522	2.444970	C	−0.726911	−0.004159	1.663082	C	1.947624	−0.000015	−0.000002
C	−0.681576	−2.249485	2.621859	C	1.982648	2.389063	0.547605	C	3.367769	−0.000029	−0.000002
C	−0.025153	−1.161645	2.083750	C	3.395883	2.395084	0.353072	C	1.982603	−2.389096	−0.547602
C	−2.082207	−2.235347	2.772225	C	−0.769755	2.389073	1.128744	C	3.395838	−2.395142	−0.353072
C	1.273580	1.156762	0.556507	C	−0.075588	3.612909	0.905194	C	−0.769802	−2.389057	−1.128733
C	−0.070455	1.157326	1.089592	C	1.273558	−1.156781	−0.556508	C	−0.075657	−3.612905	−0.905178
C	4.076452	1.223401	0.188343	C	−0.070477	−1.157323	−1.089590	C	−2.841468	−1.269814	−1.738401
C	1.277582	3.614384	0.720953	C	4.076429	−1.223471	−0.188347	C	−2.841441	1.269868	1.738415
C	−2.170294	2.411147	1.419107	C	1.277514	−3.614404	−0.720942	C	−0.726913	0.004171	−1.663087
C	−2.128543	0.045366	1.925999	C	−2.170342	−2.411106	−1.419091	C	−2.786687	1.096570	−2.444970

C	−0.681537	2.249487	−2.621884	H	5.168511	1.207985	0.141321	H	3.924282	−3.351292	−0.393056
C	−0.025133	1.161637	−2.083771	H	1.836374	4.551955	0.659266	H	−0.636502	−4.549830	−0.955122
C	−2.128547	−0.045327	−1.925995	H	−2.692774	3.370031	1.366651	H	−3.918456	−1.285843	−1.925406
C	−2.082169	2.235378	−2.772240	H	3.924345	3.351224	0.393058	H	−3.918428	1.285916	1.925423
H	−3.866799	−1.045345	2.608475	H	−0.636415	4.549845	0.955143	H	−3.866786	1.045415	−2.608466
H	−0.106275	−3.123287	2.938240	H	5.168489	−1.208075	−0.141325	H	−0.106219	3.123274	−2.938279
H	1.061176	−1.186337	2.007941	H	1.836288	−4.551986	−0.659251	H	1.061197	1.186307	−2.007971
H	−2.599967	−3.107701	3.179206	H	−2.692840	−3.369979	−1.366630	H	−2.599913	3.107740	−3.179223

[9]helicene **9H**

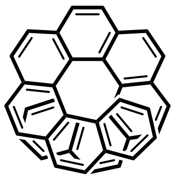

C	0.026461	−0.601535	−1.491338	C	−2.368192	2.931650	1.558367	H	−4.111266	−2.704793	−1.375327
C	−1.425885	−0.559303	−1.498538	C	1.146941	2.949183	−0.821896	H	−4.084492	1.589399	−1.733561
C	2.747243	−0.710212	−2.251482	C	0.582922	4.176141	−0.356148	H	−1.638464	1.582987	−1.775481
C	−0.115707	−3.044097	−1.796216	C	−0.026463	−0.601540	1.491336	H	2.488319	−2.832997	−2.384626
C	−3.572689	−1.754408	−1.427690	C	1.425883	−0.559310	1.498532	H	−2.033206	−3.956558	−1.534412
C	−3.547065	0.644079	−1.623531	C	−2.747245	−0.710212	2.251481	H	−5.354302	−0.552242	−1.452479
C	−2.166822	0.641268	−1.633566	C	0.115701	−3.044102	1.796218	H	−3.905145	1.710669	2.419207
C	0.649343	−1.835390	−1.800404	C	2.166822	0.641261	1.633550	H	−1.075116	5.113896	0.627344
C	2.032351	−1.869150	−2.144035	C	−0.872908	0.550401	1.279553	H	2.884120	3.877887	−1.741759
C	−2.157775	−1.783290	−1.474261	C	−2.180526	0.528054	1.831982	H	−2.884111	3.877889	1.741771
C	−1.459244	−3.026852	−1.572680	C	−0.649347	−1.835394	1.800403	H	1.075129	5.113895	−0.627327
C	−4.261676	−0.561194	−1.482026	C	−2.032355	−1.869151	2.144034	H	−3.776944	−0.716608	2.618231
C	−0.492717	1.726669	0.529083	C	2.157771	−1.783299	1.474260	H	−0.410183	−3.987807	1.963966
C	0.492722	1.726670	−0.529076	C	1.459238	−3.026859	1.572683	H	1.638464	1.582981	1.775458
C	−2.916415	1.744055	1.954118	C	4.261674	−0.561205	1.482018	H	−2.488325	−2.832997	2.384625
C	−0.582911	4.176141	0.356162	C	2.916419	1.744054	−1.954112	H	2.033199	−3.956567	1.534418
C	2.368200	2.931649	−1.558358	C	3.572685	−1.754419	1.427689	H	5.354300	−0.552255	1.452470
C	2.180527	0.528054	−1.831980	C	3.547065	0.644070	1.623515	H	3.905149	1.710667	−2.419202
C	0.872909	0.550403	−1.279551	H	3.776942	−0.716610	−2.618232	H	4.111262	−2.704805	1.375330
C	−1.146933	2.949183	0.821906	H	0.410176	−3.987803	−1.963962	H	4.084492	1.589389	1.733538

[5]phenacene

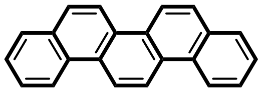

C	3.581501	0.778903	−0.000017	C	2.834827	1.995839	−0.000127	C	−0.684279	−1.666672	−0.000421
C	2.883137	−0.462272	−0.000025	C	1.472003	1.991263	−0.000165	C	0.684279	−1.666672	−0.000407
C	3.650211	−1.652659	0.000174	C	0.722749	0.770073	−0.000154	C	−1.472003	1.991263	0.000010
C	5.031191	−1.616546	0.000325	C	1.431256	−0.458110	−0.000187	C	−2.834827	1.995839	0.000077
C	5.714622	−0.385316	0.000285	C	−0.722749	0.770073	−0.000119	C	−3.581501	0.778903	0.000067
C	4.996458	0.792125	0.000126	C	−1.431256	−0.458110	−0.000190	C	−2.883137	−0.462272	−0.000041

C	−4.996458	0.792125	0.000201	H	6.807462	−0.365594	0.000400	H	−0.951853	2.948728	0.000069
C	−5.714622	−0.385316	0.000247	H	5.511078	1.756997	0.000120	H	−3.380503	2.943274	0.000167
C	−5.031191	−1.616546	0.000179	H	3.380503	2.943274	−0.000160	H	−5.511078	1.756997	0.000276
C	−3.650211	−1.652659	0.000040	H	0.951853	2.948728	−0.000236	H	−6.807462	−0.365594	0.000353
H	3.154781	−2.624375	0.000258	H	−1.201461	−2.626431	−0.000645	H	−5.595980	−2.552373	0.000244
H	5.595980	−2.552373	0.000489	H	1.201461	−2.626431	−0.000623	H	−3.154781	−2.624375	0.000025

[6]phenacene

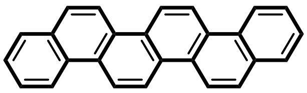

C	4.615226	0.897416	0.000039	C	−0.500100	1.796795	0.000125	H	7.902579	−0.056273	0.000399
C	3.991037	−0.383317	−0.000070	C	−1.864893	1.715104	0.000108	H	6.484322	1.986780	0.000271
C	4.826694	−1.526740	0.000037	C	−2.542302	0.464014	0.000024	H	4.287276	3.045991	0.000043
C	6.202878	−1.409549	0.000202	C	−1.764545	−0.719298	0.000015	H	1.859829	2.907000	−0.000046
C	6.812787	−0.139990	0.000270	C	−3.991035	0.383317	0.000016	H	0.044914	−2.786782	−0.000934
C	6.027171	0.993378	0.000196	C	−4.615230	−0.897413	0.000203	H	2.437476	−2.642988	−0.000913
C	3.798480	2.068092	0.000011	C	−3.798490	−2.068092	0.000396	H	−0.044907	2.786771	0.000291
C	2.437956	1.982890	−0.000034	C	−2.437966	−1.982896	0.000282	H	−2.437473	2.642981	0.000244
C	1.764543	0.719288	−0.000105	C	−4.826684	1.526745	−0.000201	H	−4.287290	−3.045989	0.000649
C	2.542303	−0.464021	−0.000232	C	−6.202869	1.409562	−0.000205	H	−1.859844	−2.907009	0.000503
C	0.320485	0.633328	−0.000060	C	−6.812785	0.140006	0.000013	H	−4.388617	2.525574	−0.000407
C	−0.320486	−0.633340	−0.000183	C	−6.027176	−0.993366	0.000210	H	−6.821745	2.310573	−0.000387
C	0.500103	−1.796805	−0.000559	H	4.388634	−2.525571	0.000033	H	−7.902578	0.056295	0.000012
C	1.864896	−1.715112	−0.000570	H	6.821759	−2.310557	0.000293	H	−6.484333	−1.986766	0.000366

[7]phenacene

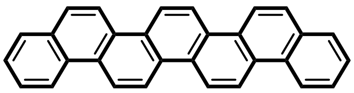

C	5.739506	0.738659	0.000822	C	−1.436985	0.726763	−0.001017	H	8.965471	−0.405780	0.002072
C	5.041408	−0.503008	0.000161	C	−0.724090	−0.498993	−0.001079	H	7.669233	1.716680	0.001996
C	5.808558	−1.693488	0.000280	C	−2.882963	0.728772	−0.000574	H	5.538668	2.902928	0.001504
C	7.189391	−1.657066	0.000954	C	−3.590197	−0.498585	−0.000289	H	3.108053	2.907222	0.000593
C	7.872634	−0.425557	0.001545	C	−2.841033	−1.707333	−0.000551	H	0.960980	−2.670607	−0.001989
C	7.154583	0.751836	0.001495	C	−1.473270	−1.708758	−0.000902	H	3.358119	−2.667161	−0.001563
C	4.993038	1.955482	0.000902	C	−3.629921	1.950531	−0.000313	H	1.193795	2.899161	−0.001599
C	3.629920	1.950531	0.000331	C	−4.993038	1.955482	0.000193	H	−1.193794	2.899161	−0.001675
C	2.882963	0.728772	−0.000433	C	−5.739506	0.738659	0.000535	H	−3.358119	−2.667161	−0.000439
C	3.590197	−0.498585	−0.000517	C	−5.041407	−0.503008	0.000326	H	−0.960980	−2.670607	−0.000961
C	1.436985	0.726763	−0.000965	C	−7.154583	0.751836	0.001141	H	−3.108054	2.907222	−0.000468
C	0.724090	−0.498993	−0.001186	C	−7.872634	−0.425557	0.001550	H	−5.538669	2.902928	0.000382
C	1.473270	−1.708758	−0.001481	C	−7.189391	−1.657066	0.001392	H	−7.669234	1.716680	0.001292
C	2.841033	−1.707333	−0.001228	C	−5.808558	−1.693488	0.000796	H	−8.965471	−0.405780	0.002017
C	0.683027	1.936653	−0.001293	H	5.313040	−2.665166	−0.000115	H	−7.754452	−2.592722	0.001752
C	−0.683027	1.936653	−0.001356	H	7.754452	−2.592722	0.001035	H	−5.313040	−2.665166	0.000736

[8]phenacene

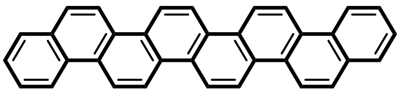

C	−4.686824	0.455809	−0.000887	C	3.939117	0.747214	−0.000342	H	−4.527360	2.631299	−0.003675
C	−3.939117	−0.747215	−0.000357	C	4.686824	−0.455809	−0.000870	H	−2.131259	2.714539	−0.005041
C	−4.644479	−1.993420	0.001912	C	3.978489	−1.689233	−0.002776	H	0.209954	−2.778990	−0.002898
C	−6.006665	−2.043971	0.003356	C	2.611692	−1.736429	−0.003539	H	−2.179283	−2.859569	−0.001948
C	−6.793471	−0.852869	0.002728	C	4.644478	1.993419	0.001976	H	−0.209954	2.778989	−0.003124
C	−6.137314	0.411623	0.000683	C	6.006664	2.043971	0.003454	H	2.179283	2.859568	−0.002157
C	−3.978489	1.689233	−0.002846	C	6.793471	0.852870	0.002777	H	4.527360	−2.631299	−0.003554
C	−2.611691	1.736428	−0.003632	C	6.137314	−0.411623	0.000669	H	2.131259	−2.714539	−0.004895
C	−1.822453	0.552288	−0.002854	C	8.207344	0.913272	0.004356	H	4.090315	2.931930	0.002924
C	−2.493889	−0.697185	−0.001789	C	8.964315	−0.239513	0.003902	H	6.520268	3.009078	0.005340
C	−0.376009	0.600057	−0.003049	C	8.322706	−1.493202	0.001877	H	8.689475	1.894755	0.005978
C	0.376009	−0.600057	−0.003008	C	6.943898	−1.575731	0.000301	H	10.055865	−0.183184	0.005139
C	−0.334636	−1.834905	−0.002808	C	−8.207344	−0.913271	0.004303	H	8.918588	−2.409557	0.001553
C	−1.700368	−1.880895	−0.002116	C	−8.964315	0.239514	0.003924	H	6.481113	−2.563347	−0.001202
C	0.334636	1.834904	−0.002940	C	−8.322705	1.493203	0.001994	H	−8.689476	−1.894754	0.005867
C	1.700368	1.880894	−0.002232	C	−6.943898	1.575732	0.000414	H	−10.055865	0.183185	0.005155
C	2.493889	0.697184	−0.001805	H	−4.090317	−2.931930	0.002821	H	−8.918587	2.409559	0.001754
C	1.822453	−0.552289	−0.002814	H	−6.520269	−3.009078	0.005183	H	−6.481112	2.563348	−0.000993

[9]phenacene

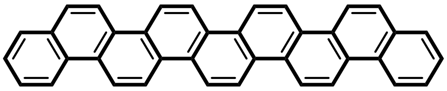

C	−3.594212	0.702659	−0.056919	C	5.039462	0.704632	−0.011514	C	−10.026476	−0.447762	0.153587
C	−2.881911	−0.523536	−0.079378	C	5.746757	−0.522509	0.001348	C	−9.308092	0.729293	0.136656
C	−3.631182	−1.733294	−0.068728	C	4.998440	−1.731520	−0.032764	H	−3.119026	−2.695069	−0.087274
C	−4.998416	−1.731505	−0.032118	C	3.631205	−1.733315	−0.069372	H	−5.515738	−2.691182	−0.027055
C	−5.746748	−0.522494	0.001630	C	5.784738	1.926646	0.027311	H	−3.352888	2.874681	−0.074578
C	−5.039464	0.704656	−0.011367	C	7.147090	1.932096	0.073492	H	−0.962601	2.874284	−0.109463
C	−2.841183	1.912612	−0.076781	C	7.893856	0.715525	0.086717	H	1.196011	−2.694510	−0.142218
C	−1.474954	1.912492	−0.098796	C	7.197051	−0.526397	0.052398	H	−1.196041	−2.694494	−0.142166
C	−0.723196	0.702068	−0.105454	C	9.308062	0.729314	0.137015	H	0.962633	2.874275	−0.110565
C	−1.434707	−0.523029	−0.105774	C	10.026462	−0.447733	0.153871	H	3.352970	2.874635	−0.075696
C	0.723214	0.702062	−0.105651	C	9.344424	−1.679515	0.122136	H	5.515774	−2.691192	−0.028000
C	1.434712	−0.523043	−0.105993	C	7.964480	−1.716570	0.072626	H	3.119069	−2.695095	−0.088195
C	0.683509	−1.732853	−0.126968	C	−7.197050	−0.526399	0.052449	H	5.261537	2.882661	0.024712
C	−0.683522	−1.732847	−0.126903	C	−7.893881	0.715516	0.086494	H	7.691731	2.879638	0.103439
C	1.474981	1.912486	−0.099450	C	−7.147134	1.932094	0.073048	H	9.821633	1.694390	0.162973
C	2.841208	1.912593	−0.077476	C	−5.784775	1.926662	0.027014	H	11.118593	−0.427505	0.192739
C	3.594217	0.702631	−0.057294	C	−7.964468	−1.716580	0.072688	H	9.909709	−2.614921	0.136876
C	2.881919	−0.523563	−0.079773	C	−9.344417	−1.679537	0.122037	H	7.469986	−2.688510	0.050885

H	−7.691797	2.879636	0.102618
H	−5.261632	2.882707	0.024080
H	−7.469965	−2.688518	0.051051
H	−9.909691	−2.614950	0.136783
H	−11.118611	−0.427547	0.192355
H	−9.821676	1.694367	0.162431

Benzo[*ghi*]perylene BP

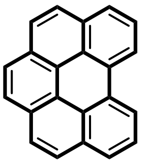

C	−0.733398	−1.588605	0.000015
C	0.733398	−1.588605	−0.000016
C	−3.565473	−1.537579	0.000024
C	−0.712888	0.895202	−0.000012
C	2.856518	−0.322913	0.000011
C	2.882294	−2.743273	−0.000076
C	1.485459	−2.768508	−0.000076
C	−1.485459	−2.768508	0.000073
C	−2.882294	−2.743273	0.000074
C	−1.429252	−0.343634	−0.000007
C	−2.847739	2.112679	−0.000051
C	−3.537830	0.937921	−0.000042
C	−2.856518	−0.322913	−0.000011
C	1.429252	−0.343634	0.000007
C	1.416710	2.126143	0.000029
C	0.712888	0.895202	0.000012
C	−1.416710	2.126143	−0.000028
C	−0.687400	3.340959	−0.000015
C	0.687400	3.340959	0.000016
C	2.847739	2.112679	0.000053
C	3.537830	0.937921	0.000043
C	3.565473	−1.537579	−0.000024
H	−4.658514	−1.516191	0.000019
H	3.435704	−3.685678	−0.000120
H	0.984955	−3.737612	−0.000134
H	−0.984955	−3.737612	0.000131
H	−3.435704	−3.685678	0.000118
H	−3.378189	3.068675	−0.000072
H	−4.631166	0.935819	−0.000054
H	−1.239489	4.284635	−0.000028
H	1.239489	4.284635	0.000028
H	3.378189	3.068675	0.000074
H	4.631166	0.935819	0.000056
H	4.658514	−1.516191	−0.000018

O7H

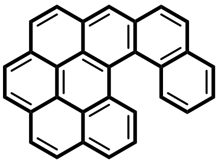

C	4.363481	1.654299	0.593426
C	4.743631	0.331567	0.646977
C	3.811562	−0.701614	0.391995
C	2.460535	−0.360025	0.125497
C	2.061121	1.005114	0.095225
C	3.028251	2.020998	0.301931
C	0.685302	1.347660	−0.121639
C	0.328215	2.732336	−0.207867
C	1.342798	3.736103	−0.057499
C	2.633077	3.396341	0.207715
C	4.202128	−2.078362	0.345421
C	3.316233	−3.044329	−0.028031
C	1.958396	−2.721168	−0.357932
C	1.513268	−1.372832	−0.213156
C	−0.308100	0.337116	−0.224630
C	−1.682669	0.725724	−0.148347
C	−2.006340	2.091607	−0.424732
C	−0.998362	3.064270	−0.464864
C	1.069978	−3.689293	−0.855646
C	−0.208875	−3.319582	−1.248326
C	−0.658040	−2.011365	−1.066605
C	0.153872	−1.032060	−0.477582
C	−2.784309	−0.139527	0.267986
C	−4.126744	0.271250	0.023332
C	−4.386519	1.579284	−0.506869
C	−3.374087	2.471672	−0.651790
C	−2.593153	−1.317673	1.028496
C	−3.656889	−2.107752	1.420525
C	−4.972929	−1.754386	1.071309
C	−5.199192	−0.571473	0.396566
H	5.097341	2.445307	0.769761
H	5.781595	0.064446	0.862863
H	1.047463	4.785379	−0.139778
H	3.394651	4.167964	0.348488
H	5.238890	−2.334663	0.579419
H	3.632452	−4.088251	−0.103518
H	−1.270689	4.107573	−0.646232
H	1.412585	−4.721337	−0.968114
H	−0.877941	−4.056668	−1.699392
H	−1.667203	−1.753088	−1.386994
H	−5.421860	1.867524	−0.707566
H	−3.575428	3.503490	−0.952077
H	−1.585356	−1.603424	1.329591
H	−3.470404	−3.007025	2.013157
H	−5.810734	−2.391922	1.365031
H	−6.219198	−0.248705	0.170031

O8H

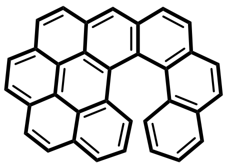

C	−2.409079	0.792920	−0.031778	C	−1.109112	1.420701	−0.217554	H	−5.077180	−2.831934	1.115541
C	−2.615982	−0.471739	0.648475	C	−1.087036	2.852593	−0.310566	H	−1.057792	−2.759286	2.667979
C	−2.293311	3.561991	−0.614790	C	1.335009	1.460482	−0.029969	H	−0.664848	−0.598651	1.591527
C	−4.832086	0.858172	−0.485655	C	3.671840	2.914913	0.693114	H	−4.374848	3.419638	−1.083623
C	−4.093923	−2.360570	1.198812	C	2.509037	3.585186	0.470998	H	−5.956712	−0.921919	−0.097144
C	−1.846069	−2.311354	2.057649	C	1.305993	2.885607	0.120999	H	0.099046	4.647763	−0.159179
C	−1.622122	−1.097149	1.441970	C	1.527044	−1.323201	−0.583455	H	−1.375076	−3.130222	−2.441283
C	−3.558674	1.504243	−0.449368	C	3.884572	−1.314151	0.153488	H	−1.619343	−0.777350	−1.784705
C	−3.465856	2.893401	−0.780656	C	2.662118	−0.628299	−0.066877	H	4.578144	3.452857	0.983812
C	−3.897638	−1.098406	0.586297	C	3.742211	1.490431	0.541050	H	2.463339	4.672675	0.572526
C	−4.986465	−0.421297	−0.040928	C	4.943146	0.780316	0.777992	H	5.826145	1.337353	1.102703
C	0.148298	0.740584	−0.325300	C	5.011481	−0.583494	0.598279	H	5.949026	−1.117058	0.776118
C	0.295966	−0.637835	−0.799796	C	0.583261	−3.350153	−1.572030	H	0.682277	−4.405303	−1.840195
C	0.113050	3.554810	−0.130473	C	−3.082806	−2.969852	1.910025	H	−3.247695	−3.940896	2.383919
C	2.584405	0.776758	0.141408	C	3.953114	−2.717029	−0.123003	H	4.891768	−3.242424	0.072495
C	1.654397	−2.700370	−0.934701	C	2.886783	−3.378057	−0.655838	H	2.958370	−4.441773	−0.898442
C	−0.564844	−2.641674	−1.894452	H	−2.238098	4.645600	−0.747529				
C	−0.705514	−1.306217	−1.515435	H	−5.683787	1.417369	−0.882123				

O9H

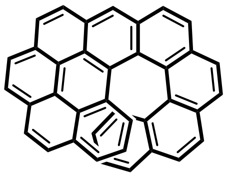

C	−0.833220	−3.171058	2.055024	C	−4.884625	−0.104291	−0.424917	C	1.433357	−1.051863	−0.999025
C	0.322890	−1.153158	2.672220	C	0.166435	1.035219	−0.505568	C	3.803304	−1.221782	−0.327318
C	−0.578208	−0.399003	1.947182	C	0.234250	−0.292045	−1.118026	C	2.612979	−0.460651	−0.454652
C	0.221734	−2.557361	2.696714	C	0.239050	3.798256	0.062119	C	3.808805	1.539524	0.317575
C	−2.358252	1.102008	−0.059586	C	2.609741	0.921307	−0.117366	C	4.979048	0.755495	0.452287
C	−2.570265	−0.249321	0.402199	C	1.483132	−2.395594	−1.476067	C	4.975334	−0.588225	0.148442
C	−2.198108	3.924630	−0.256293	C	−0.743780	−2.133733	−2.383482	C	0.369248	−2.928223	−2.147197
C	−4.764597	1.251673	−0.551832	C	−0.809522	−0.833816	−1.879601	C	−2.959526	−3.040995	0.804412
C	−3.956473	−2.287307	0.263313	C	−1.054262	1.729863	−0.233695	C	3.793194	−2.597670	−0.723954
C	−1.790314	−2.413815	1.337247	C	−0.994177	3.157201	−0.130077	C	2.683820	−3.156055	−1.285353
C	−1.622645	−1.003545	1.205377	C	1.389028	1.671760	−0.176566	H	−0.963932	−4.255232	2.112511
C	−3.502792	1.882386	−0.368375	C	3.806127	2.942607	0.617025	H	1.115775	−0.652240	3.233453
C	−3.390027	3.306357	−0.473516	C	2.665115	3.677571	0.520785	H	−0.493417	0.686617	1.968249
C	−3.800484	−0.877024	0.078657	C	1.419665	3.067697	0.150746	H	0.953222	−3.151313	3.250459

H	−2.124727	5.014870	−0.228063
H	−5.622368	1.867832	−0.833908
H	−4.893406	−2.749169	−0.059638
H	−4.297260	3.885531	−0.664904
H	−5.829481	−0.607447	−0.646573
H	0.261897	4.885190	0.178699
H	−1.584791	−2.526372	−2.960332
H	−1.694430	−0.232818	−2.086507
H	4.744560	3.410249	0.926951
H	2.670031	4.747220	0.746419
H	5.895682	1.237419	0.802962
H	5.889324	−1.179235	0.251978
H	0.407370	−3.958200	−2.511445
H	−3.066598	−4.124417	0.904081
H	4.707448	−3.183749	−0.597364
H	2.695231	−4.196831	−1.620188

OO7H

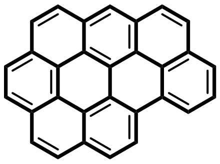

C	−4.533899	1.361646	0.000097
C	−4.799162	0.012803	0.000089
C	−3.748280	−0.940796	0.000040
C	−2.403584	−0.483128	0.000020
C	−2.131156	0.914586	0.000031
C	−3.200857	1.845722	0.000060
C	−0.779553	1.373882	0.000000
C	−0.510400	2.776295	−0.000026
C	−1.611805	3.690476	−0.000007
C	−2.901712	3.243677	0.000039
C	−4.001068	−2.344484	0.000001
C	−2.972106	−3.245628	−0.000059
C	−1.613361	−2.811967	−0.000071
C	−1.327068	−1.420737	−0.000020
C	0.292208	0.445312	−0.000009
C	1.628290	0.912672	−0.000036
C	1.887804	2.320291	−0.000072
C	0.820058	3.218144	−0.000065
C	−0.532472	−3.723861	−0.000142
C	0.769888	−3.282557	−0.000122
C	1.089726	−1.901246	−0.000027
C	0.026237	−0.966675	−0.000011
C	2.712458	−0.017313	−0.000009
C	4.054954	0.471666	−0.000003
C	4.283630	1.891364	−0.000069
C	3.251284	2.774081	−0.000104
C	2.464623	−1.422342	0.000041
C	3.568801	−2.292010	0.000162
C	4.872934	−1.807330	0.000183
C	5.119993	−0.438150	0.000085
H	−5.354411	2.084288	0.000132
H	−5.831948	−0.345935	0.000118
H	−1.396751	4.762366	−0.000038
H	−3.733060	3.953899	0.000054
H	−5.038725	−2.688843	0.000013
H	−3.175792	−4.319808	−0.000101
H	1.025130	4.292304	−0.000091
H	−0.745903	−4.796019	−0.000226
H	1.569547	−4.024343	−0.000198
H	5.317703	2.246357	−0.000088
H	3.438454	3.851213	−0.000151
H	3.413967	−3.371221	0.000250
H	5.708216	−2.512148	0.000278
H	6.145149	−0.058461	0.000081

OO8H

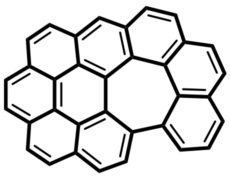

C	−2.440798	0.735696	−0.361463
C	−2.880393	−0.534990	0.188122
C	−2.034113	3.498715	−0.930439
C	−4.845269	1.204365	−0.823943
C	−4.705545	−1.848018	1.233187
C	−2.455242	−2.636682	1.347791
C	−2.007134	−1.602051	0.528990
C	−3.471067	1.584634	−0.854521
C	−3.217780	2.931047	−1.250460
C	−4.274962	−0.757580	0.441657
C	−5.247335	0.108168	−0.130472
C	0.166547	0.581542	0.019445
C	0.351044	−0.866920	−0.151395
C	0.148442	3.417018	0.058928
C	2.658420	0.743993	0.312832
C	1.868962	−2.739157	−0.804701
C	−0.487461	−3.115251	−0.603184
C	−0.694147	−1.808755	−0.110464
C	−1.079384	1.281828	−0.229931
C	−0.985189	2.715315	−0.358395
C	1.351646	1.341373	0.277306
C	3.661681	2.892389	0.998189
C	2.457936	3.502392	0.860847
C	1.304090	2.762911	0.436029
C	1.669891	−1.391495	−0.377906
C	4.129813	−1.157691	−0.211218
C	2.825133	−0.602374	−0.100504
C	3.804401	1.508263	0.659584
C	5.084956	0.912643	0.607083
C	5.247086	−0.380060	0.166769

C	−3.795252	−2.741748	1.746476	H	−1.746552	−3.411686	1.648004	H	5.953134	1.512140	0.893516
C	4.285211	−2.492622	−0.693879	H	−4.028729	3.511844	−1.696231	H	6.244451	−0.822668	0.100577
C	3.192795	−3.246359	−0.995488	H	−6.307083	−0.122973	0.004207	H	−4.112551	−3.559762	2.397633
C	0.753235	−3.566463	−0.993356	H	0.126112	4.509739	0.042259	H	5.294729	−2.896723	−0.805408
H	−1.848214	4.562445	−1.097894	H	−1.345603	−3.787745	−0.660269	H	3.303107	−4.272277	−1.356414
H	−5.575257	1.880807	−1.275391	H	4.547979	3.452989	1.306500	H	0.885016	−4.579923	−1.380187
H	−5.775419	−1.955303	1.430291	H	2.343567	4.574647	1.039705				

OO9H

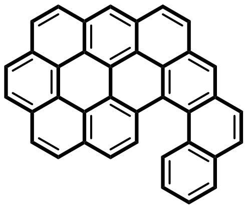

C	2.362017	3.429377	0.349537	C	−0.269281	−2.930173	−1.100525	H	2.782416	4.429727	0.485531
C	3.214552	2.313827	0.419829	C	−1.116055	−1.859011	−0.957176	H	−1.836989	5.169269	−0.369448
C	2.671037	1.013643	0.208460	C	−0.666515	−0.605063	−0.459133	H	0.582758	5.434714	0.064399
C	1.277638	0.854234	−0.006550	C	−3.711669	−0.743414	0.363366	H	−3.776255	3.707577	−0.780520
C	0.432674	1.985502	−0.047012	C	−5.121279	−0.832237	0.161664	H	−0.652685	−3.880301	−1.481582
C	0.998682	3.293180	0.097482	C	−5.827908	0.274202	−0.411643	H	−2.161178	−1.973244	−1.242648
C	−0.977429	1.820992	−0.212176	C	−5.185842	1.449331	−0.643498	H	−6.904325	0.180831	−0.578357
C	−1.797512	2.991365	−0.341488	C	−3.104104	−1.744349	1.160913	H	−5.737464	2.331906	−0.978326
C	−1.190831	4.294336	−0.259670	C	−3.818907	−2.829321	1.629324	H	−2.052594	−1.648710	1.431947
C	0.137549	4.440360	−0.025406	C	−5.185877	−2.967926	1.324407	H	−3.318010	−3.577698	2.248731
C	3.534343	−0.121098	0.185755	C	−5.825663	−1.972215	0.614771	H	−5.743145	−3.838419	1.679783
C	3.003232	−1.406115	−0.122287	C	4.922711	0.035866	0.426549	H	−6.900090	−2.032381	0.420080
C	1.608395	−1.559632	−0.382092	C	5.760779	−1.109244	0.381693	H	6.829007	−0.985969	0.579446
C	0.727689	−0.441230	−0.267670	C	5.251216	−2.349567	0.080899	H	5.911194	−3.220218	0.037890
C	−1.560003	0.523651	−0.245732	C	3.868821	−2.529372	−0.192965	H	5.026454	3.438389	0.846804
C	−2.990287	0.419264	−0.137709	C	4.616743	2.440244	0.670554	H	6.504776	1.457497	0.876248
C	−3.767377	1.570079	−0.461626	C	5.434341	1.344566	0.684060	H	3.998020	−4.657793	−0.606762
C	−3.154674	2.832349	−0.572643	C	3.325823	−3.797200	−0.552433	H	1.592506	−4.911158	−1.137221
C	1.104233	−2.828324	−0.766869	C	1.994671	−3.938212	−0.842296				

OO8H$_{mt}$

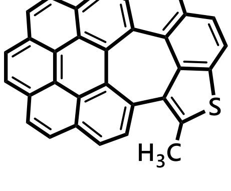

C	−4.626899	1.807073	−0.658967	C	−2.885185	3.435581	−1.079597	C	−0.562128	−2.919180	−0.825422
C	−5.088040	0.640095	−0.104146	C	−3.238913	2.092778	−0.748567	C	−0.691580	−1.631843	−0.261869
C	−2.017537	−1.340849	0.289474	C	−2.238864	1.131296	−0.396675	C	0.416167	−0.753583	−0.234962
C	−2.748240	−0.138960	0.008477	C	1.817059	−2.716328	−0.912113	C	1.701043	−1.371234	−0.442757
C	−1.627007	3.862444	−0.838905	C	0.654393	−3.447910	−1.185855	C	1.585652	1.367024	0.251255

C	0.343093	0.712615	−0.041564
C	−0.831433	1.539852	−0.275325
C	−0.621290	2.962297	−0.358951
C	1.660763	2.786678	0.426937
C	0.573864	3.550864	0.054824
C	2.838108	0.663663	0.319658
C	5.331080	−0.668681	0.241614
C	5.260453	0.624733	0.700882
C	4.032778	1.323993	0.716060
C	2.860062	3.422462	0.890734
C	4.001070	2.711817	1.064273
C	2.907825	−0.684947	−0.111600
C	3.101849	−3.323205	−1.072522
C	4.167656	−1.341673	−0.192362
C	4.237925	−2.671116	−0.706508
C	−2.781530	−2.304101	0.917274
S	−4.449035	−1.827512	1.027835
C	−4.125526	−0.313412	0.252683
C	−2.392543	−3.629174	1.483205
H	−5.330416	2.584840	−0.965890
H	−6.153715	0.461494	0.054707
H	−1.347313	4.909430	−0.978864
H	−3.663305	4.118222	−1.429475
H	0.726902	−4.448178	−1.619605
H	−1.466754	−3.511183	−0.969166
H	0.649333	4.641210	0.069146
H	6.289373	−1.192983	0.200255
H	6.163240	1.146570	1.029001
H	2.829675	4.499148	1.076093
H	4.921390	3.190914	1.407992
H	3.142038	−4.343041	−1.463899
H	5.216317	−3.150756	−0.792829
H	−1.302180	−3.703187	1.591316
H	−2.843178	−3.767240	2.479195
H	−2.731585	−4.469465	0.854577

O8H$_a$

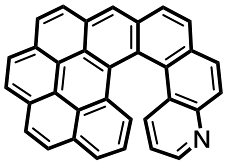

C	−2.407969	0.778436	−0.036158
C	−2.602087	−0.485325	0.643961
C	−2.313551	3.543391	−0.624640
C	−4.838073	0.827579	−0.448899
N	−4.111875	−2.333210	1.166382
C	−1.878416	−2.328873	2.033598
C	−1.614810	−1.115854	1.437664
C	−3.567262	1.481296	−0.438252
C	−3.485193	2.869284	−0.776306
C	−3.877901	−1.122313	0.606291
C	−4.983202	−0.450324	0.002644
C	0.146439	0.736304	−0.331397
C	0.300062	−0.644102	−0.798605
C	0.096344	3.552071	−0.150829
C	2.580496	0.785785	0.139859
C	1.666998	−2.701834	−0.916884
C	−0.548498	−2.656586	−1.886738
C	−0.695713	−1.319333	−1.516056
C	−1.113110	1.411783	−0.228210
C	−1.100659	2.842943	−0.325208
C	1.328792	1.463415	−0.038172
C	3.657257	2.932121	0.679490
C	2.492208	3.596310	0.451075
C	1.292382	2.889415	0.104032
C	1.533093	−1.323392	−0.573810
C	3.888437	−1.299829	0.169089
C	2.663901	−0.620368	−0.058705
C	3.734207	1.506814	0.537177
C	4.937406	0.803187	0.781658
C	5.011374	−0.561618	0.611448
C	−3.141174	−2.919884	1.831380
C	3.963236	−2.704295	−0.098123
C	2.901216	−3.372994	−0.629959
C	0.600686	−3.359070	−1.554801
H	−2.265334	4.626615	−0.763030
H	−5.698975	1.382494	−0.831359
H	−1.129840	−2.824608	2.655192
H	−0.652328	−0.630416	1.599832
H	−4.399339	3.390410	−1.072178
H	−5.944130	−0.968668	−0.021332
H	0.077183	4.644761	−0.184576
H	−1.353834	−3.150893	−2.435595
H	−1.609693	−0.794949	−1.794069
H	4.560726	3.475794	0.968363
H	2.441983	4.684190	0.545692
H	5.817519	1.365770	1.104574
H	5.950606	−1.090251	0.794994
H	−3.358770	−3.904786	2.262709
H	4.903369	−3.224760	0.103308
H	2.977931	−4.437813	−0.865987
H	0.704546	−4.415264	−1.816901

O8H$_a$H$^+$

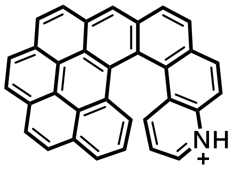

C	−2.376981	0.818352	−0.052547
C	−2.580662	−0.446433	0.618429
C	−2.265521	3.580179	−0.611175
C	−4.808768	0.889143	−0.445957
C	−1.842434	−2.320763	1.997150
C	−1.599886	−1.096038	1.399649
C	−3.535872	1.534251	−0.434405
C	−3.446829	2.922450	−0.762703
C	−3.866740	−1.047645	0.576290

C	−4.976505	−0.393093	−0.007306
C	0.173129	0.747282	−0.344241
C	0.312858	−0.633474	−0.814339
C	0.145371	3.565325	−0.144447
C	2.602449	0.771649	0.140805
C	1.658176	−2.705295	−0.924268
C	−0.549450	−2.634243	−1.912644
C	−0.685154	−1.295676	−1.541449
C	−1.078197	1.435926	−0.241884
C	−1.057411	2.867869	−0.322699
C	1.359098	1.462501	−0.040576
C	3.696766	2.905868	0.690527
C	2.539513	3.583065	0.460894
C	1.334289	2.888724	0.108352
C	1.536806	−1.326105	−0.580591
C	3.887391	−1.327154	0.174328
C	2.671441	−0.634760	−0.059003
C	3.760680	1.480292	0.545161
C	4.955502	0.764369	0.794142
C	5.015792	−0.600948	0.622558
C	−3.089641	−2.911979	1.837042
C	3.949189	−2.732135	−0.093414
C	2.883516	−3.389947	−0.631275
C	0.589217	−3.349481	−1.571300
H	−2.207131	4.663707	−0.741882
H	−5.666969	1.451251	−0.822538
H	−1.078944	−2.820671	2.593310
H	−0.636233	−0.613272	1.560763
H	−4.356769	3.453924	−1.051096
H	−5.947528	−0.891213	−0.037633
H	0.136711	4.658045	−0.172426
H	−1.351768	−3.118188	−2.474996
H	−1.587961	−0.758550	−1.833033
H	4.604582	3.439876	0.983381
H	2.499786	4.670855	0.558705
H	5.839845	1.317284	1.121697
H	5.948633	−1.139388	0.809488
H	−3.359167	−3.880974	2.260189
H	4.883150	−3.261858	0.111866
H	2.951083	−4.454907	−0.868942
H	0.684934	−4.405518	−1.836879
N	−4.036949	−2.264763	1.165612
H	−4.964399	−2.689516	1.106109

5H* (racemization transition state)

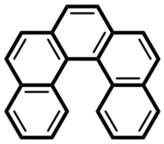

C	−2.909604	−0.214211	0.474562
C	−1.662339	−0.405977	−0.211602
C	−1.506026	−1.690592	−0.781035
C	−2.347447	−2.754495	−0.502124
C	−3.438722	−2.590337	0.361406
C	−3.731668	−1.316846	0.803163
C	−3.414304	1.102130	0.697489
C	−2.725269	2.161831	0.208827
C	−1.381350	2.011893	−0.264125
C	−0.735533	0.736714	−0.345176
C	−0.679901	3.220742	−0.497576
C	0.679895	3.220743	−0.497573
C	1.381345	2.011896	−0.264120
C	0.735531	0.736715	−0.345174
C	2.725263	2.161836	0.208836
C	3.414300	1.102135	0.697493
C	2.909605	−0.214207	0.474561
C	1.662340	−0.405974	−0.211604
C	3.731672	−1.316841	0.803159
C	3.438728	−2.590332	0.361401
C	2.347453	−2.754491	−0.502128
C	1.506030	−1.690590	−0.781037
H	−0.767274	−1.843198	−1.552008
H	−2.166523	−3.717156	−0.987322
H	−4.086226	−3.433652	0.614094
H	−4.643383	−1.123926	1.375419
H	−4.387614	1.224668	1.179482
H	−3.134407	3.173704	0.266136
H	−1.244874	4.154320	−0.558677
H	1.244866	4.154323	−0.558670
H	3.134397	3.173709	0.266151
H	4.387610	1.224674	1.179488
H	4.643386	−1.123919	1.375415
H	4.086235	−3.433646	0.614087
H	2.166531	−3.717152	−0.987327
H	0.767278	−1.843200	−1.552011

6H* (racemization transition state)

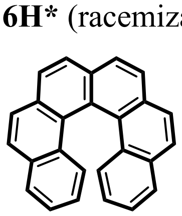

C	1.312367	0.774344	−0.206209
C	1.724091	−0.627320	−0.183004
C	1.198099	3.544601	−0.759001
C	3.384687	1.213922	1.088338
C	3.045685	−2.432041	0.844361
C	1.814709	−2.862989	−1.170785
C	1.400234	−1.543818	−1.204445
C	2.334370	1.665765	0.223037
C	2.308382	3.033080	−0.161609
C	2.703880	−1.062113	0.764924
C	3.475153	−0.087956	1.471310
C	−1.312974	0.773520	−0.206053

C	−1.723645	−0.628412	−0.182884	C	−2.580611	−3.335928	−0.093921	H	4.218117	−0.418419	2.201716
C	−1.200632	3.543797	−0.758881	C	2.583141	−3.334227	−0.093613	H	−1.154904	4.593910	−1.057941
C	−3.385708	1.211540	1.088325	C	−3.044030	−2.434201	0.844069	H	−4.078368	1.961844	1.479292
C	−1.398866	−1.544524	−1.204348	C	−1.812331	−2.864012	−1.170907	H	−0.886018	−1.171833	−2.085334
C	−0.000518	1.361957	−0.517275	H	1.151657	4.594675	−1.058082	H	−3.181301	3.656931	0.052827
C	−0.001007	2.775467	−0.766659	H	4.076745	1.964759	1.479349	H	−4.218211	−0.421479	2.201400
C	−2.335620	1.664204	0.223170	H	3.749289	−2.748457	1.619501	H	−2.873936	−4.387631	−0.042157
C	−2.310558	3.031539	−0.161448	H	1.554895	−3.532188	−1.994894	H	2.877270	−4.385697	−0.041691
C	−2.703258	−1.064005	0.764840	H	0.887229	−1.171762	−2.085612	H	−3.747523	−2.751256	1.619049
C	−3.475342	−0.090442	1.471158	H	3.178703	3.659057	0.052669	H	−1.551838	−3.532950	−1.995016

O7H* (racemization transition state)

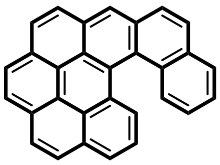

C	−4.435023	−1.538018	0.301393	C	1.852857	−2.174173	−0.119586	H	−1.269703	−4.669807	−0.852547
C	−4.722369	−0.255387	0.703332	C	0.843163	−3.089276	−0.413986	H	−3.632485	−3.987346	−0.492462
C	−3.731842	0.754442	0.654473	C	−0.987331	3.832689	−0.248444	H	−5.014904	2.348508	1.395282
C	−2.428690	0.422757	0.200758	C	0.126557	3.509161	−0.996750	H	−3.349383	4.137618	0.985778
C	−2.109894	−0.914746	−0.155985	C	0.506724	2.172268	−1.163034	H	1.060704	−4.159490	−0.362371
C	−3.132449	−1.898860	−0.121074	C	−0.164890	1.107824	−0.555184	H	−1.275552	4.873441	−0.079425
C	−0.748609	−1.272347	−0.448093	C	2.906940	0.028229	−0.133134	H	0.720690	4.291848	−1.475172
C	−0.469110	−2.667699	−0.571191	C	3.905857	−0.480145	0.762791	H	1.306897	1.968900	−1.855799
C	−1.532710	−3.624735	−0.669131	C	3.914028	−1.868608	1.121135	H	4.719405	−2.244775	1.757229
C	−2.822976	−3.253329	−0.462132	C	3.008318	−2.699684	0.558181	H	3.060852	−3.780771	0.709864
C	−4.025267	2.109841	0.996705	C	3.285367	1.201897	−0.820326	H	2.770429	1.478344	−1.728419
C	−3.107428	3.092483	0.774669	C	4.395015	1.960083	−0.472837	H	4.629293	2.856985	−1.051978
C	−1.829586	2.798262	0.199431	C	5.222436	1.561047	0.581226	H	6.076754	2.170606	0.886087
C	−1.449765	1.437394	−0.005946	C	4.996599	0.323126	1.155008	H	5.700793	−0.085836	1.884926
C	0.305331	−0.297109	−0.505733	H	−5.209141	−2.309740	0.322820				
C	1.667536	−0.759948	−0.327920	H	−5.727376	0.006874	1.044502				

O8H* (racemization transition state)

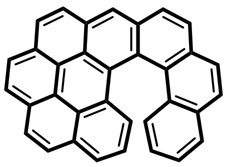

C	−2.255972	1.039753	−0.046960	C	−4.274600	−1.901237	1.244691	C	−2.782974	3.426588	0.336055
C	−2.913910	−0.263057	0.004580	C	−3.686970	−2.335926	−1.041097	C	−3.702757	−0.609641	1.148669
C	−1.776653	3.801103	−0.490443	C	−3.037751	−1.117738	−1.112666	C	−4.078394	0.409254	2.074206
C	−3.835532	1.707787	1.742725	C	−2.958235	2.047996	0.665208	C	0.250967	0.618782	−0.685140

C	0.361522	−0.837413	−0.843050	C	2.521250	−0.831449	0.351333	H	−4.672509	0.145058	2.952825
C	0.517823	3.403270	−1.021687	C	3.838454	1.231126	0.352851	H	0.602201	4.477667	−1.204962
C	2.629207	0.554664	0.058350	C	4.877735	0.530410	1.013510	H	−0.903251	−3.489936	−2.591068
C	1.432261	−2.966875	−0.170449	C	4.749397	−0.801182	1.340368	H	−0.965313	−1.021454	−2.509406
C	−0.294758	−2.959791	−1.854311	C	0.527280	−3.666101	−0.992836	H	4.936436	3.105354	0.168876
C	−0.366918	−1.563344	−1.783473	C	−4.243975	−2.771207	0.174270	H	3.030831	4.352450	−0.810512
C	−0.954030	1.428621	−0.628682	C	3.479222	−2.939968	1.154065	H	5.796953	1.069541	1.258369
C	−0.751479	2.849804	−0.801624	C	2.454583	−3.635294	0.579719	H	5.566477	−1.328138	1.840384
C	1.502925	1.268754	−0.479847	H	−1.619819	4.848670	−0.758986	H	0.549138	−4.759034	−1.007149
C	3.979279	2.609921	−0.014706	H	−4.259625	2.526984	2.329756	H	−4.717214	−3.753913	0.245569
C	2.935327	3.293648	−0.555700	H	−4.814415	−2.163550	2.158954	H	4.273813	−3.464115	1.691930
C	1.655490	2.666996	−0.728283	H	−3.779254	−2.951905	−1.939198	H	2.422140	−4.726579	0.641659
C	1.402907	−1.543591	−0.167337	H	−2.691921	−0.758108	−2.076827				
C	3.584614	−1.522819	0.983973	H	−3.466680	4.159945	0.772085				

OO9H* (racemization transition state)

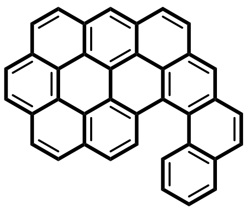

C	2.440079	3.398287	−0.047687	C	−0.147215	−2.954255	−1.299723	H	2.879044	4.398649	−0.000076
C	3.244384	2.283586	0.230927	C	−0.964592	−1.848435	−1.354241	H	−1.715947	5.181137	−0.782697
C	2.671001	0.983153	0.134162	C	−0.637817	−0.604851	−0.760739	H	0.752088	5.407742	−0.623791
C	1.297854	0.833752	−0.189547	C	−3.839699	−0.731898	0.007533	H	−3.643565	3.842761	−0.160236
C	0.484132	1.966560	−0.397700	C	−4.861659	−0.627358	1.011144	H	−0.470762	−3.889729	−1.763718
C	1.087040	3.266979	−0.355354	C	−5.329481	0.657465	1.434480	H	−1.853044	−1.915052	−1.960347
C	−0.933906	1.812722	−0.547847	C	−4.836258	1.766131	0.836770	H	−6.147958	0.711420	2.156871
C	−1.708148	3.015772	−0.558628	C	−3.843441	−1.960771	−0.690845	H	−5.252869	2.754465	1.046181
C	−1.074078	4.302536	−0.677503	C	−4.569619	−3.068105	−0.279115	H	−3.356367	−2.033717	−1.651324
C	0.270695	4.427348	−0.583193	C	−5.377084	−3.003737	0.861806	H	−4.525462	−3.983382	−0.874969
C	3.488899	−0.164780	0.340657	C	−5.550388	−1.773225	1.464932	H	−5.922745	−3.882331	1.214695
C	2.945544	−1.459856	0.116328	C	4.849629	−0.015253	0.711869	H	−6.277110	−1.648550	2.272562
C	1.589785	−1.602111	−0.309361	C	5.638593	−1.177496	0.904500	H	6.681966	−1.060547	1.209507
C	0.730197	−0.462625	−0.389402	C	5.115607	−2.431549	0.690590	H	5.740919	−3.318321	0.824954
C	−1.567880	0.521966	−0.583410	C	3.772413	−2.604996	0.267275	H	5.049691	3.408643	0.684690
C	−2.991088	0.453295	−0.261652	C	4.620929	2.406463	0.600955	H	6.435513	1.410495	1.137683
C	−3.642730	1.697175	0.039531	C	5.386491	1.302311	0.849057	H	3.880478	−4.767612	0.076952
C	−3.057584	2.927968	−0.282853	C	3.239294	−3.890700	−0.046677	H	1.579494	−5.005723	−0.813553
C	1.120385	−2.886469	−0.687909	C	1.967489	−4.023391	−0.531361				

OO8H* (racemization transition state)

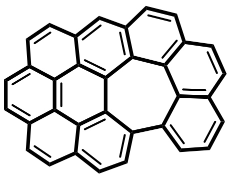

C	−2.371196	0.882281	0.108432	C	−0.739352	−1.793458	−0.668026	H	−1.886939	4.675340	−0.886571
C	−2.861129	−0.489221	0.142756	C	−1.039541	1.339254	−0.310804	H	−5.125890	2.431966	1.521818
C	−2.029456	3.645923	−0.550536	C	−0.929555	2.739586	−0.638378	H	−5.838575	−2.064964	1.004549
C	−4.516172	1.597958	1.165366	C	1.425500	1.313746	−0.259460	H	−2.625693	−3.580937	−1.297062
C	−4.854480	−1.949442	0.544403	C	3.840198	2.884374	−0.079668	H	−3.958187	3.958489	0.310879
C	−3.016928	−2.738045	−0.739970	C	2.700748	3.451102	−0.544423	H	−5.855733	0.064495	1.820680
C	−2.213807	−1.619148	−0.477118	C	1.475919	2.705071	−0.595201	H	0.315491	4.411731	−1.164787
C	−3.312563	1.893279	0.468949	C	1.611279	−1.477723	−0.049531	H	−1.053942	−3.742524	−1.581850
C	−3.148995	3.257440	0.093172	C	3.977722	−1.281962	0.693680	H	4.764302	3.460276	0.015132
C	−4.174028	−0.722802	0.695571	C	2.747381	−0.683689	0.296562	H	2.670138	4.503247	−0.838665
C	−4.915731	0.315438	1.323649	C	3.850913	1.498691	0.275172	H	5.916958	1.519627	0.906953
C	0.192057	0.577276	−0.271898	C	5.035477	0.892834	0.748160	H	6.020271	−0.931158	1.311811
C	0.306147	−0.896154	−0.289989	C	5.093600	−0.462029	0.971140	H	−4.849734	−3.844102	−0.478906
C	0.298207	3.354139	−0.891347	C	−4.312422	−2.919996	−0.253640	H	5.036391	−3.150044	1.056250
C	2.674588	0.716483	0.114869	C	4.097777	−2.702818	0.719318	H	3.180533	−4.554318	0.162012
C	1.856137	−2.874795	−0.189582	C	3.081440	−3.468551	0.240123	H	1.088466	−4.679966	−1.102080
C	−0.344411	−3.089462	−1.087429	C	0.881702	−3.641775	−0.831014				

OO8H$_{mt}$* (racemization transition state)

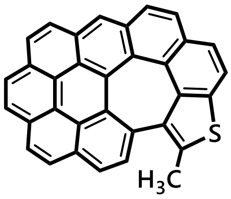

C	4.265001	2.212373	1.042302	C	0.696606	1.611449	−0.180928	C	4.077379	−0.068206	0.506717
C	4.849769	0.980460	1.022271	C	0.427972	3.003996	−0.439508	C	3.392167	−3.599687	−0.966240
C	2.211729	−1.253640	−0.351792	C	−1.940950	2.648010	−0.642845	H	4.826059	3.091281	1.367949
C	2.713088	0.021308	0.147793	C	−0.841185	3.462489	−0.786177	H	5.884504	0.820822	1.332961
C	1.388154	4.042290	−0.230090	C	−2.938028	0.538788	0.014363	H	1.096604	5.068424	−0.464303
C	2.570101	3.760437	0.352107	C	−5.252540	−0.878028	0.802657	H	3.280152	4.547268	0.617112
C	2.945387	2.400414	0.558404	C	−5.347377	0.451811	0.473345	H	−0.676492	−4.717991	−0.670850
C	2.086999	1.311709	0.187310	C	−4.212273	1.172074	0.043992	H	1.355461	−3.548071	−1.200447
C	−1.682187	−2.939244	0.038290	C	−3.248036	3.231241	−0.740836	H	−0.977501	4.518401	−1.031402
C	−0.603411	−3.641813	−0.495880	C	−4.347779	2.531659	−0.376102	H	−6.135194	−1.438286	1.121742
C	0.552165	−2.966611	−0.779018	C	−2.852257	−0.845591	0.289939	H	−6.308410	0.970296	0.523123
C	0.777281	−1.605167	−0.473948	C	−2.847361	−3.645076	0.467406	H	−3.317320	4.274242	−1.059696
C	−0.367765	−0.795067	−0.174464	C	−4.025338	−1.562299	0.667334	H	−5.342769	2.983409	−0.392313
C	−1.607095	−1.512934	0.065186	C	−3.976513	−2.978867	0.820901	H	−2.805327	−4.737220	0.485699
C	−1.743336	1.272268	−0.292737	C	3.247319	−2.188510	−0.468679	H	−4.873729	−3.509349	1.149481
C	−0.426292	0.692403	−0.209170	S	4.749218	−1.598401	0.135473	H	2.883553	−4.331889	−0.319292

H	3.017873	−3.724978	−1.993479	H	4.456688	−3.878583	−0.985269

8H$^{\cdot+}$

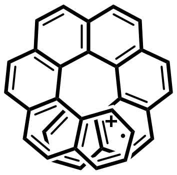

C	2.753350	1.279064	2.234902	C	−1.042728	3.565212	−0.921859	H	−1.123850	1.346946	2.047883
C	0.663402	2.452019	2.466765	C	2.362969	2.106355	−1.389083	H	2.608974	3.340718	2.829966
C	−0.033519	1.325828	2.054614	C	−1.939297	0.096327	−0.041368	H	−5.194947	−0.967696	0.108707
C	2.066920	2.443023	2.523777	C	−3.366459	0.170623	−0.054085	H	−2.009993	−4.424846	0.929316
C	−1.324170	−1.055507	0.604070	C	−1.843873	2.412332	−0.765066	H	2.562606	−3.267266	1.550115
C	−0.035884	−1.044777	1.198906	C	−3.260595	2.517815	−0.611016	H	−4.051860	−3.132970	0.517461
C	−4.105274	−1.006239	0.182323	C	0.954278	2.189381	−1.165645	H	0.457213	−4.464141	1.319508
C	−1.422509	−3.503961	0.943914	C	0.327613	3.455584	−1.013443	H	−5.102558	1.453281	−0.329554
C	2.039555	−2.308149	1.546472	C	2.955324	0.907712	−1.658445	H	−1.522507	4.546603	−0.915102
C	2.057804	0.109505	1.848109	C	2.737197	−1.136074	1.723689	H	2.952332	3.024987	−1.336073
C	0.645459	0.153847	1.668815	C	0.748127	−0.206986	−1.642201	H	−3.725116	3.498576	−0.737071
C	−2.076230	−2.269971	0.644954	C	2.758433	−1.477948	−2.267661	H	0.949012	4.353955	−1.049322
C	−3.467684	−2.213679	0.428822	C	0.580509	−2.496531	−2.461114	H	4.036831	0.838018	−1.799385
C	0.641212	−2.300438	1.325834	C	2.164100	−0.265796	−1.839580	H	3.822512	−1.157034	1.851412
C	−0.080374	−3.522894	1.184584	C	1.985673	−2.581086	−2.557805	H	3.844346	−1.512023	−2.388319
C	−1.204041	1.134757	−0.681525	C	−0.021142	−1.338634	−2.018641	H	−0.037588	−3.349540	−2.751542
C	0.166241	1.013881	−1.143243	H	3.841169	1.234696	2.330858	H	2.454807	−3.508013	−2.896706
C	−4.011427	1.408988	−0.357300	H	0.110482	3.348370	2.757543	H	−1.109639	−1.284115	−1.988509

8HH^{+}

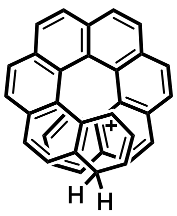

C	−2.747664	−1.312178	2.256295	C	3.244902	2.616865	0.363803	C	−0.567986	−2.464513	−1.090268
C	−0.573557	−2.306026	2.550520	C	−0.921821	2.331614	1.092831	C	0.212323	−3.641605	−0.896839
C	0.029541	−1.164133	2.064811	C	−0.304646	3.599856	0.892490	C	−2.730012	−1.442536	−1.625063
C	−1.977922	−2.400615	2.609499	C	1.386955	−1.085908	−0.518424	C	−2.935612	1.060015	1.589602
C	1.210007	1.238408	0.562501	C	0.046914	−1.189849	−1.031095	C	−0.710150	−0.074224	−1.556663
C	−0.138803	1.150665	1.075703	C	4.176685	−0.946383	−0.177995	C	−0.777422	2.212794	−2.530397
C	4.006108	1.495723	0.201935	C	1.561937	−3.537643	−0.720167	C	−2.110134	−0.193984	−1.773620
C	1.048200	3.691172	0.725427	C	−1.960830	−2.553129	−1.352125	C	−2.140209	2.185295	−2.588522
C	−2.332281	2.252279	1.323325	C	1.970404	0.124743	0.023441	C	−0.082467	1.090068	−2.032548
C	−2.147728	−0.115410	1.796101	C	3.384522	0.225245	0.015690	H	−3.835847	−1.344958	2.357991
C	−0.734288	−0.058069	1.614461	C	2.178980	−2.265283	−0.533365	H	0.045384	−3.137879	2.895604
C	1.834128	2.515925	0.551931	C	3.588934	−2.167021	−0.347013	H	1.117508	−1.104419	2.053990

H	−2.451231	−3.316433	2.972311	H	2.189965	−4.430782	−0.672980	H	−2.705661	3.056915	−2.929985
H	5.096348	1.556620	0.156453	H	−2.425640	−3.542341	−1.328626	H	1.008288	1.119805	−2.056710
H	1.544813	4.663489	0.676970	H	4.189231	−3.078958	−0.393273	C	−2.888946	0.990677	−2.202148
H	−2.917037	3.174324	1.266459	H	−0.279005	−4.615507	−0.954083	H	−3.546046	1.287446	−1.351656
H	3.705938	3.606889	0.405658	H	−3.804436	−1.538212	−1.795868	H	−3.629309	0.734793	−2.983532
H	−0.925388	4.498308	0.938483	H	−4.018381	0.997652	1.727162				
H	5.264515	−0.851283	−0.132601	H	−0.212386	3.087533	−2.856435				

C8H$^{\cdot+}$

“opt=loose” was applied in the calculation.

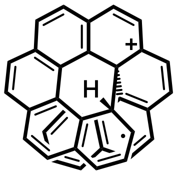

C	−0.570642	−2.923019	2.655477	C	3.801670	−0.926914	−0.847533	H	1.547484	0.228650	1.872449
C	1.525155	−1.736004	2.675122	C	0.875786	−2.969133	−2.009416	H	1.245631	−3.751054	3.445041
C	0.948647	−0.665070	2.031330	C	1.072113	1.501952	−0.058706	H	1.678546	4.757848	0.813865
C	0.771826	−2.895979	2.956574	C	1.800216	2.638477	0.321712	H	−2.986283	3.765908	0.389857
C	−0.428261	1.456781	0.075620	C	3.169944	0.308222	−0.493743	H	−4.329313	−0.662503	1.278123
C	−1.074515	0.387445	0.941550	C	3.885700	1.421851	0.016690	H	−0.779101	4.896643	0.615922
C	1.096103	3.863383	0.578127	C	1.665779	−1.897665	−1.511204	H	−4.245188	1.612466	0.491807
C	−2.459116	2.809012	0.402233	C	3.074087	−2.001894	−1.284530	H	3.775440	3.462975	0.673352
C	−3.240182	−0.707041	1.354178	C	−0.464365	−2.795207	−2.253442	H	4.884815	−1.009211	−0.726453
C	−1.202406	−1.821035	2.026889	C	−2.612029	−1.819152	1.837575	H	1.349991	−3.937795	−2.185621
C	−0.418018	−0.683483	1.628863	C	−0.295306	−0.440266	−1.641809	H	4.973130	1.360463	0.105195
C	−1.060963	2.777553	0.313131	C	−2.450655	−1.286730	−2.397378	H	3.570505	−2.951457	−1.499876
C	−0.269458	3.940217	0.476011	C	−2.241286	1.084016	−1.942202	H	−1.077789	−3.630015	−2.601280
C	−2.479506	0.416387	0.923191	C	−1.074137	−1.516302	−2.098409	H	−3.183943	−2.701332	2.135844
C	−3.152026	1.616345	0.510117	C	−3.004338	−0.012448	−2.331278	H	−3.063938	−2.130522	−2.724133
C	1.763288	0.403793	−0.615628	C	−0.851352	0.934955	−1.535965	H	−2.634147	2.087716	−2.100336
C	1.036644	−0.659390	−1.249384	H	−1.182635	−3.789353	2.920327	H	−4.039929	0.139892	−2.644261
C	3.221109	2.576053	0.356568	H	2.573157	−1.679669	2.979426	H	−0.233459	1.652071	−2.101258

C8HH^{+}

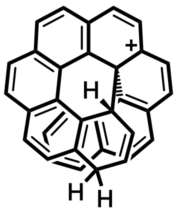

C	−0.585054	−2.951643	2.592123	C	−1.079746	0.393204	0.948396	C	−0.426947	−0.694501	1.605406
C	1.515325	−1.770066	2.630926	C	1.082497	3.879622	0.507222	C	−1.065954	2.792615	0.286561
C	0.942005	−0.688180	2.003731	C	−2.460490	2.839469	0.455077	C	−0.285771	3.960434	0.376291
C	0.759412	−2.933641	2.887753	C	−3.252668	−0.678041	1.408387	C	−2.486222	0.443651	0.979882
C	−0.431160	1.459956	0.063787	C	−1.215972	−1.834888	1.992696	C	−3.155166	1.670794	0.667360

C	1.780309	0.411750	−0.586547
C	1.068416	−0.665675	−1.211096
C	3.216281	2.609672	0.384284
C	3.840182	−0.897393	−0.771901
C	0.945040	−2.963714	−1.966420
C	1.075612	1.508617	−0.049197
C	1.794358	2.657694	0.321386
C	3.189528	0.331199	−0.439918
C	3.890756	1.456581	0.069237
C	1.719093	−1.893400	−1.459544
C	3.127287	−1.979447	−1.213893
C	−0.398683	−2.788034	−2.222244
C	−2.629155	−1.815110	1.834566
C	−0.274328	−0.463366	−1.593318
C	−2.195610	1.091317	−1.975607
C	−1.027011	−1.528749	−2.061314
C	−2.935542	0.055193	−2.402809
C	−0.805682	0.940876	−1.489314
H	−1.198355	−3.820387	2.845063
H	2.563009	−1.721638	2.937016
H	1.542559	0.206527	1.862072
H	1.232409	−3.800381	3.356000
H	1.657718	4.785684	0.718986
H	−2.973314	3.803533	0.454816
H	−4.342591	−0.611111	1.371375
H	−0.792168	4.923773	0.466551
H	−4.245681	1.692042	0.746333
H	3.757338	3.507405	0.693077
H	4.921955	−0.967468	−0.633878
H	1.423387	−3.928979	−2.151266
H	4.977338	1.402684	0.171808
H	3.635967	−2.925488	−1.417685
H	−0.995101	−3.627388	−2.590576
H	−3.204198	−2.698627	2.122100
H	−2.585354	2.104242	−2.088554
H	−3.926966	0.252838	−2.823172
H	−0.167829	1.612243	−2.090228
C	−2.481698	−1.361469	−2.391440
H	−3.090928	−1.935809	−1.665658
H	−2.708650	−1.825664	−3.367229

8H-IM1

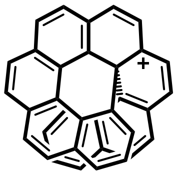

C	1.395333	1.890009	−3.231151
C	2.251868	−0.100943	−2.138347
C	1.116247	−0.201674	−1.356600
C	2.384342	0.941840	−3.078730
C	−1.499886	−0.867063	−0.461913
C	−1.274840	0.395202	−1.092220
C	−1.983457	−3.498661	0.381194
C	−3.903174	−0.408303	−0.457135
C	−2.134416	2.424540	−2.148689
C	0.179007	1.754806	−2.525780
C	0.021651	0.666014	−1.623695
C	−2.831575	−1.312838	−0.225218
C	−3.042499	−2.641108	0.235209
C	−2.360984	1.240740	−1.380862
C	−3.670976	0.835972	−0.988286
C	0.980861	−1.103285	−0.071124
C	1.258585	0.003730	1.002454
C	0.453276	−3.935239	0.210437
C	3.395313	−1.721154	0.199933
C	2.940116	1.693321	1.628003
C	−0.411550	−1.685425	−0.113632
C	−0.647676	−3.037178	0.191231
C	2.065743	−2.147301	0.014442
C	1.754333	−3.511733	0.018849
C	2.600750	0.434949	1.056766
C	3.646946	−0.436331	0.619441
C	1.969191	2.494834	2.157673
C	−0.913984	2.657895	−2.716929
C	0.292511	0.702911	1.780397
C	−0.318295	2.750557	3.018980
C	−1.855687	0.894343	2.934475
C	−0.964229	0.161554	2.187402
C	0.644260	2.002519	2.299065
C	−1.554774	2.221234	3.311934
H	1.521058	2.724409	−3.925446
H	3.076834	−0.803481	−2.033799
H	3.296397	0.997196	−3.677821
H	−2.142949	−4.546258	0.646834
H	−4.921300	−0.728905	−0.223430
H	−2.970739	3.104954	−2.327084
H	−4.064304	−2.980500	0.420977
H	−4.506019	1.515683	−1.177109
H	0.252427	−4.999666	0.362711
H	4.208122	−2.443612	0.096275
H	3.985887	2.009213	1.609011
H	2.570799	−4.237330	0.007041
H	4.679024	−0.100585	0.752945
H	2.208179	3.487790	2.545188
H	−0.759645	3.529080	−3.358759
H	−0.042829	3.747879	3.371198
H	−2.798826	0.439162	3.245797
H	−1.200801	−0.874960	1.965294
H	−2.285318	2.807554	3.874747

8H-TS1

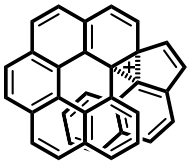

C	−1.019001	−4.044781	−0.163102
C	−1.909193	−2.173591	−1.416164
C	−0.871250	−1.350194	−0.996862
C	−1.981112	−3.513623	−0.999326
C	1.645300	−0.016665	−0.845769
C	1.457636	−1.252332	−0.156858
C	2.070052	2.351589	−2.310395
C	4.012549	−0.056420	−0.220290
C	2.366620	−3.145391	1.102357
C	0.117940	−3.281211	0.176147
C	0.214615	−1.941509	−0.290414
C	2.941547	0.558270	−0.925854
C	3.128279	1.741915	−1.687627
C	2.544674	−1.859274	0.506073
C	3.813447	−1.211004	0.491140
C	−0.820354	0.136993	−1.190622
C	−1.530416	0.841101	0.250118
C	−0.365393	2.572522	−2.650666
C	−3.302985	0.457061	−1.670122
C	−3.175544	0.254032	1.961629
C	0.541327	0.650233	−1.427338
C	0.752240	1.839498	−2.160669
C	−1.912330	0.960834	−1.704503
C	−1.648172	2.177087	−2.388957
C	−2.813506	0.386910	0.609429
C	−3.765842	0.132992	−0.458767
C	−2.291214	0.618206	2.948320
C	1.202523	−3.836553	0.925983
C	−0.678204	1.436562	1.247686
C	−0.232248	1.721514	3.670096
C	1.249349	2.698768	2.059849
C	0.479701	2.221066	1.019954
C	−1.063445	1.241083	2.624079
C	0.915020	2.427301	3.401299
H	−1.096393	−5.074156	0.196063
H	−2.688918	−1.793594	−2.071443
H	−2.817236	−4.130324	−1.337113
H	2.214410	3.267603	−2.887406
H	5.001292	0.407024	−0.260070
H	3.200083	−3.589774	1.651994
H	4.132537	2.166462	−1.761341
H	4.644585	−1.678459	1.025356
H	−0.181167	3.486343	−3.221482
H	−3.853020	0.351932	−2.608470
H	−4.165296	−0.137523	2.206090
H	−2.499308	2.763693	−2.738530
H	−4.757529	−0.274837	−0.258602
H	−2.550068	0.485315	4.001680
H	1.090919	−4.846868	1.328037
H	−0.542484	1.528736	4.700415
H	2.128024	3.306680	1.830159
H	0.758865	2.506107	0.012447
H	1.545060	2.797698	4.213087

8H-IM2

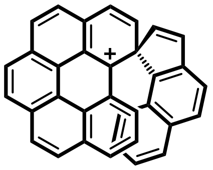

C	−1.083138	−3.802305	0.443018
C	0.485211	−2.064559	1.021670
C	−0.452519	−1.055141	0.695609
C	0.173943	−3.410310	0.896141
C	−2.429336	0.876317	0.034605
C	−2.731225	−0.500030	−0.089138
C	−1.859408	3.630215	0.241241
C	−4.698591	1.406521	−0.736560
C	−4.300455	−2.304142	−0.664901
C	−2.056328	−2.846248	0.111458
C	−1.745798	−1.463527	0.239312
C	−3.419365	1.837609	−0.293524
C	−3.106494	3.211514	−0.176023
C	−4.020258	−0.912880	−0.541967
C	−4.990603	0.070616	−0.858439
C	−0.147773	0.336997	0.806518
C	2.433040	0.255460	0.545466
C	0.436813	3.161774	0.976459
C	1.531646	0.420100	2.731152
C	4.610226	−0.712045	1.069780
C	−1.132783	1.292471	0.480733
C	−0.856632	2.702248	0.570103
C	1.212039	0.797194	1.282830
C	1.406684	2.283851	1.300427
C	3.357898	−0.200705	1.483081
C	2.779498	−0.086785	2.814704
C	4.895641	−0.754965	−0.274950
C	−3.352413	−3.236174	−0.349785
C	2.706413	0.253458	−0.840807
C	4.271788	−0.333534	−2.637597
C	2.136175	0.679212	−3.161837
C	1.807696	0.739213	−1.826364
C	3.971296	−0.286087	−1.251995
C	3.375498	0.135280	−3.572199
H	−1.326225	−4.863476	0.342681
H	1.475578	−1.792927	1.375979
H	0.921953	−4.162394	1.154227
H	−1.634103	4.696040	0.317267
H	−5.448326	2.162388	−0.982667
H	−5.288934	−2.610936	−1.014736
H	−3.873619	3.948360	−0.428538
H	−5.975767	−0.253036	−1.202371
H	0.623048	4.237377	1.011232
H	0.838204	0.639147	3.543222
H	5.329570	−1.071403	1.809249
H	2.398729	2.622142	1.608393
H	3.282770	−0.387369	3.735377
H	5.852905	−1.154704	−0.620204
H	−3.570391	−4.303022	−0.443673
H	5.233309	−0.749222	−2.951172
H	1.436097	1.056531	−3.911540
H	0.853609	1.170035	−1.515815
H	3.621289	0.092787	−4.636231

8H-TS2

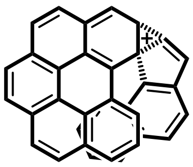

C	0.632407	−3.510717	−1.389174	C	−1.326386	2.630347	−1.662869	H	−1.385896	−3.364739	−2.122517
C	−0.687349	−1.486416	−1.337839	C	−4.521269	0.727251	−1.410089	H	2.461100	4.538245	0.939106
C	0.352594	−0.775425	−0.725744	C	1.393940	1.348990	0.057543	H	5.890249	1.259776	1.138383
C	−0.550092	−2.838869	−1.655430	C	1.336049	2.770764	0.344795	H	5.016413	−3.297466	0.021371
C	2.642609	0.678693	0.202997	C	−1.016350	1.286865	−0.228962	H	4.612492	3.333694	1.189029
C	2.734202	−0.722561	−0.049750	C	−1.073089	2.737546	−0.087847	H	6.043200	−1.176628	0.714268
C	2.532517	3.466376	0.741578	C	−3.180780	1.158822	−1.278176	H	0.084139	4.504687	0.494999
C	5.010772	0.702928	0.806321	C	−2.454510	2.060908	−2.152339	H	−0.568886	3.116396	−2.280359
C	4.060583	−2.793210	−0.140423	C	−5.044132	−0.129590	−0.470819	H	−5.123772	1.075485	−2.252078
C	1.729904	−2.824090	−0.840688	C	2.982945	−3.479353	−0.613628	H	−1.988622	3.137083	0.369100
C	1.599113	−1.439504	−0.525499	C	−2.902787	−0.158574	0.768595	H	−2.755721	2.230517	−3.190211
C	3.795540	1.394812	0.627938	C	−4.806097	−1.461116	1.605778	H	−6.076414	−0.478130	−0.557128
C	3.707980	2.804993	0.878557	C	−2.697559	−1.470914	2.799043	H	3.058360	−4.545631	−0.841386
C	3.971971	−1.392837	0.135340	C	−2.138321	−0.621697	1.873017	H	−5.844329	−1.786796	1.500647
C	5.094299	−0.653157	0.571382	C	−4.267034	−0.582296	0.631346	H	−2.102073	−1.821737	3.645474
C	0.229357	0.619509	−0.322933	C	−4.040733	−1.895764	2.664190	H	−1.099605	−0.300077	1.978283
C	−2.383159	0.693075	−0.235702	H	0.734929	−4.572378	−1.627859	H	−4.469746	−2.572169	3.407672
C	0.138505	3.438282	0.263207	H	−1.619674	−0.984550	−1.589074				

8H-TS2'

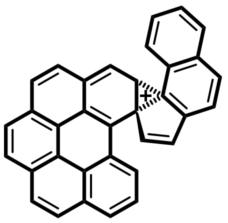

C	−2.542718	3.594760	0.909375	C	−0.272184	0.436819	−0.868638	C	2.669076	−2.386286	2.108458
C	−0.482036	2.800670	−0.073144	C	2.626750	0.172968	−0.688475	C	2.271833	−1.723975	0.963456
C	−1.018240	1.517159	−0.250964	C	1.237994	−1.791126	−1.876573	C	4.079225	−0.122166	1.270699
C	−1.241915	3.827661	0.489217	C	1.397606	1.924677	−2.191659	C	3.776462	−1.943799	2.856475
C	−2.110143	−1.128723	−0.363215	C	4.417697	1.778043	−0.207264	H	−3.136345	4.400637	1.348484
C	−2.882626	−0.056125	0.168718	C	−0.799523	−0.880727	−0.869355	H	0.551860	3.003165	−0.351111
C	−0.632410	−3.303373	−1.439223	C	−0.045367	−1.991300	−1.418725	H	−0.798230	4.818851	0.608126
C	−3.958726	−2.658911	0.091243	C	0.952849	0.660552	−1.556567	H	−0.042084	−4.123883	−1.853090
C	−4.953345	0.776254	1.203750	C	1.801577	−0.465307	−1.938703	H	−4.371809	−3.669869	0.055777
C	−3.106248	2.310504	0.803977	C	3.296081	1.387035	−0.961370	H	−5.962404	0.571078	1.569799
C	−2.335770	1.257493	0.230134	C	2.676722	2.253519	−1.952797	H	−2.320493	−4.515742	−0.966050
C	−2.656467	−2.439194	−0.403160	C	4.805443	1.030730	0.878461	H	−5.712921	−1.806654	0.988206
C	−1.880850	−3.515465	−0.950847	C	−4.430277	2.032856	1.273748	H	1.808963	−2.627822	−2.285494
C	−4.191801	−0.305344	0.660801	C	2.954249	−0.576245	0.498117	H	0.723162	2.435422	−2.884590
C	−4.705063	−1.620712	0.608286	C	4.467124	−0.830388	2.436446	H	4.938700	2.700645	−0.472154

H	2.442824	−0.313444	−2.816906	H	−5.011914	2.854409	1.699414	H	1.398284	−2.091438	0.436795
H	3.198363	3.103091	−2.395274	H	5.333445	−0.467579	2.995710	H	4.081009	−2.479100	3.758695
H	5.662408	1.337605	1.483400	H	2.107320	−3.264577	2.436166				

8H-IM3

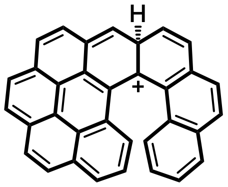

C	0.474602	−3.358562	−1.571407	C	−2.179685	3.571968	−0.729280	H	−1.482581	−3.088082	−2.424752
C	−0.758562	−1.280693	−1.505203	C	−4.733894	0.900349	−0.642207	H	2.631637	4.622890	0.569345
C	0.266797	−0.645611	−0.799671	C	1.380699	1.433011	−0.013640	H	5.878778	1.181980	1.030571
C	−0.655718	−2.621299	−1.884619	C	1.407918	2.866528	0.167634	H	4.800890	−3.362064	0.029784
C	2.602082	0.716301	0.128771	C	−1.055052	1.410566	−0.116746	H	4.721603	3.326287	0.911748
C	2.634043	−0.694696	−0.085325	C	−1.042561	2.894634	−0.031483	H	5.913248	−1.273095	0.713306
C	2.646193	3.535802	0.464660	C	−3.459040	1.515414	−0.551361	H	0.248640	4.666897	0.011600
C	4.972606	0.654276	0.723786	C	−3.294685	2.880624	−1.011808	H	−2.080273	4.639254	−0.940269
C	3.872515	−2.816489	−0.156280	C	−4.948499	−0.348522	−0.122116	H	−5.543453	1.443733	−1.135045
C	1.569579	−2.739211	−0.942529	C	2.784509	−3.450068	−0.678071	H	−1.307013	3.081774	1.056655
C	1.479394	−1.360635	−0.590017	C	−2.627552	−0.433604	0.699062	H	−4.135135	3.363355	−1.516197
C	3.793264	1.398604	0.505655	C	−4.180966	−2.268943	1.224460	H	−5.928060	−0.824605	−0.214481
C	3.786462	2.825235	0.650783	C	−1.951580	−2.277479	2.138738	H	2.825415	−4.514603	−0.922513
C	3.837509	−1.413249	0.118608	C	−1.668979	−1.078901	1.518455	H	−5.174089	−2.712810	1.116882
C	4.990058	−0.711759	0.546943	C	−3.922486	−1.027257	0.591796	H	−1.193374	−2.749620	2.768350
C	0.163368	0.734314	−0.311566	C	−3.209792	−2.893255	1.974798	H	−0.695550	−0.615128	1.680113
C	−2.374054	0.810991	0.015342	H	0.543060	−4.415666	−1.840355	H	−3.417845	−3.848968	2.461947
C	0.243888	3.573026	0.009553	H	−1.660183	−0.729991	−1.770987				

8H-IM3'

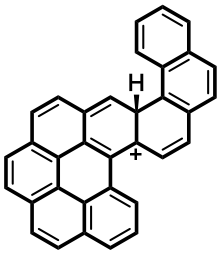

C	−3.948958	−3.215418	−0.854180	C	−4.250156	−1.887490	−0.500633	C	0.857664	−2.245898	0.942929
C	−1.585089	−2.738990	−0.687225	C	−3.185236	−0.981401	−0.221898	C	4.586456	−2.291018	0.870151
C	−1.833305	−1.436969	−0.232939	C	−2.727883	2.718904	0.299954	C	−1.074213	0.887347	0.214937
C	−2.630507	−3.618038	−0.984109	C	−1.666695	3.671167	0.446887	C	−0.036035	1.871661	0.406066
C	−2.425559	1.335410	0.161493	C	−4.830793	0.839227	−0.001370	C	0.530429	−0.929599	0.507022
C	−3.484534	0.393808	0.001683	C	−5.097815	2.219336	0.161986	C	1.632327	0.065724	0.631472
C	−0.372285	3.266865	0.494643	C	−0.769916	−0.506856	0.148760	C	3.262723	−1.769785	0.818159
C	−4.077276	3.133478	0.300564	C	3.027863	−0.455816	0.376867	C	2.154457	−2.610062	1.171138
C	−5.882827	−0.107034	−0.203447	C	1.275123	1.471690	0.501399	C	5.643889	−1.533061	0.457113

C	−5.601500	−1.417080	−0.452172
C	4.104354	0.301231	−0.183753
C	6.519074	0.474451	−0.661752
C	5.013704	2.177971	−1.459215
C	3.940430	1.518699	−0.903484
C	5.431154	−0.247230	−0.113076
C	6.321937	1.670951	−1.313118
H	−4.767158	−3.908063	−1.066718
H	−0.562464	−3.077153	−0.850246
H	−2.397023	−4.628604	−1.327240
H	0.441165	3.985451	0.617834
H	−4.300944	4.196772	0.414672
H	−6.916032	0.248077	−0.181915
H	−1.925189	4.729421	0.530697
H	−6.137890	2.555578	0.161747
H	2.047630	2.225384	0.654556
H	0.059076	−2.959981	1.141177
H	4.735357	−3.304095	1.251184
H	1.686026	0.147123	1.765506
H	2.370464	−3.603614	1.572164
H	6.665103	−1.917739	0.515932
H	−6.406095	−2.132903	−0.638266
H	7.521303	0.046278	−0.577558
H	4.846842	3.099055	−2.022856
H	2.944390	1.927648	−1.067845
H	7.169441	2.214324	−1.737936

O8H'

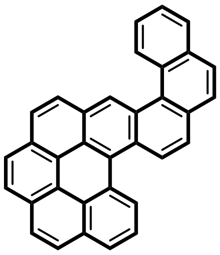

C	3.993762	3.196555	−0.431366
C	5.051002	2.318915	−0.340325
C	4.831788	0.935838	−0.141212
C	3.498988	0.452839	−0.054317
C	2.407614	1.358853	−0.154338
C	2.657314	2.742272	−0.337101
C	1.061987	0.865562	−0.111960
C	−0.018166	1.796919	−0.234402
C	0.269090	3.194915	−0.386445
C	1.550436	3.648494	−0.438419
C	3.246091	−0.930572	0.193385
C	1.906318	−1.421605	0.281452
C	0.793471	−0.520842	−0.023341
C	5.916253	0.014477	0.004927
C	5.684140	−1.300171	0.278455
C	4.350318	−1.808243	0.410727
C	4.110181	−3.142587	0.782770
C	2.811549	−3.581255	0.987080
C	1.727816	−2.732965	0.749802
C	−1.637010	−0.035245	−0.178713
C	−1.330628	1.335421	−0.196217
C	−0.548408	−0.969612	−0.255974
C	−0.841872	−2.289117	−0.726469
C	−2.116499	−2.665352	−1.029481
C	−3.229188	−1.815221	−0.752214
C	−3.008811	−0.523625	−0.226924
C	−4.552882	−2.268068	−1.037370
C	−5.634389	−1.481059	−0.776771
C	−5.472038	−0.229984	−0.110135
C	−4.159505	0.237340	0.219761
C	−6.607977	0.519354	0.281703
C	−6.480235	1.666104	1.035228
C	−5.199911	2.076872	1.453329
C	−4.075093	1.379982	1.059235
H	4.175639	4.264350	−0.580024
H	6.078929	2.684646	−0.409993
H	−0.571879	3.888688	−0.468127
H	1.759742	4.713955	−0.565612
H	6.938316	0.392800	−0.082149
H	6.517351	−1.993887	0.419426
H	4.958713	−3.811520	0.948726
H	2.626067	−4.597731	1.343180
H	0.725798	−3.101750	0.965843
H	−2.128710	2.072138	−0.279443
H	−0.023003	−2.972733	−0.946768
H	−2.306830	−3.642460	−1.480993
H	−4.676652	−3.256063	−1.488770
H	−6.644580	−1.815272	−1.027805
H	−7.596817	0.151676	−0.006399
H	−7.365558	2.231090	1.337806
H	−5.092697	2.943845	2.110351
H	−3.106860	1.697086	1.444603

8HH$^+$

ma-SVP was used as the basis set.

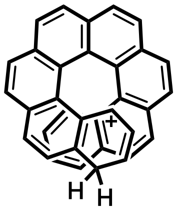

C	2.736583	−1.341981	−2.257586
C	0.554829	−2.321148	−2.550444
C	−0.040158	−1.173207	−2.068096

C	1.959138	−2.426407	−2.607931
C	−1.202872	1.238255	−0.568933
C	0.144984	1.141883	−1.082770
C	−3.997966	1.507334	−0.210799
C	−1.029170	3.689235	−0.742790
C	2.344899	2.228857	−1.335075
C	2.144807	−0.139546	−1.800594
C	0.731960	−0.072039	−1.619078
C	−1.821274	2.518371	−0.564379
C	−3.232216	2.625565	−0.377299
C	0.934747	2.318089	−1.104872
C	0.323805	3.590568	−0.909200
C	−1.387586	−1.079150	0.523779
C	−0.048514	−1.182237	1.038535
C	−4.176656	−0.933896	0.180820
C	−1.566424	−3.529057	0.739384
C	1.959144	−2.543315	1.366373
C	−1.967471	0.130065	−0.024772
C	−3.381087	0.235018	−0.018264
C	−2.181831	−2.256644	0.544992
C	−3.591891	−2.155716	0.356999
C	0.565826	−2.456557	1.104259
C	−0.216318	−3.633912	0.916583
C	2.727394	−1.430640	1.634778
C	2.940582	1.031432	−1.597563
C	0.706922	−0.063935	1.560700
C	0.770405	2.224557	2.530757
C	2.106759	−0.181295	1.777874
C	2.133665	2.200176	2.586701
C	0.076955	1.098811	2.037582
H	3.824549	−1.383483	−2.359390
H	−0.070021	−3.149368	−2.893668
H	−1.127767	−1.106016	−2.058237
H	2.426219	−3.346809	−2.967588
H	−5.088100	1.573278	−0.166610
H	−1.520367	4.664647	−0.699084
H	2.936348	3.146942	−1.281218
H	−3.690399	3.616797	−0.424328
H	0.948305	4.486295	−0.958754
H	−5.264388	−0.836969	0.134223
H	−2.195220	−4.422076	0.697537
H	2.425076	−3.532154	1.348197
H	−4.195370	−3.065436	0.408007
H	0.272865	−4.608659	0.978841
H	3.801991	−1.525425	1.805862
H	4.023178	0.961994	−1.733800
H	0.204340	3.098803	2.856504
H	2.697981	3.074259	2.924004
H	−1.013912	1.125575	2.065275
C	2.884373	1.005229	2.204010
H	3.543637	1.299147	1.354154
H	3.622993	0.752085	2.987996

8H$H^{+}\cdot CF_3SO_3^{-}$

ma-SVP was used as the basis set.

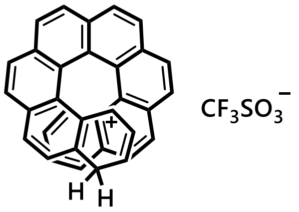

C	−3.187665	−2.278175	2.493033
C	−3.310523	0.079920	2.973187
C	−3.117197	0.375282	1.638978
C	−3.304939	−1.258443	3.414938
C	−2.225016	0.897426	−1.247498
C	−2.674382	−0.374444	−0.727767
C	−1.985340	3.537973	−2.236088
C	−2.943050	0.165467	−3.486836
C	−3.102035	−2.758749	−1.185490
C	−3.032505	−1.998021	1.113682
C	−2.910656	−0.645913	0.677806
C	−2.522855	1.201868	−2.603649
C	−2.391457	2.541821	−3.077051
C	−2.938951	−1.415176	−1.651405
C	−3.049560	−1.122281	−3.041725
C	−0.637400	1.568815	0.655540
C	−0.090112	0.270822	0.951938
C	−1.047012	4.262257	−0.060533
C	0.133597	2.307334	2.878292
C	0.593209	−1.368611	2.637941
C	−1.455108	1.875452	−0.500314
C	−1.496197	3.225391	−0.932671
C	−0.358207	2.614656	1.575009
C	−0.584839	3.969120	1.190785
C	0.275667	−0.028941	2.285672
C	0.349144	1.012926	3.255623
C	0.636343	−2.379872	1.705880
C	−3.072189	−3.050736	0.145665
C	0.188896	−0.739704	−0.044905
C	0.652521	−1.352664	−2.408872
C	0.378563	−0.410191	−1.398232
C	0.486999	−2.070918	0.345338
C	0.786117	−2.669170	−2.075729
C	0.715413	−3.108729	−0.684625
H	−0.081736	−3.880198	−0.603661
H	1.631139	−3.685270	−0.450783
H	−3.242967	−3.322905	2.811399
H	−3.470721	0.891065	3.687764
H	−3.147034	1.415891	1.316857
H	−3.431429	−1.486364	4.476466
H	−1.973349	4.580614	−2.563855
H	−3.129900	0.414758	−4.534618
H	−3.232933	−3.550560	−1.928103
H	−2.669403	2.759440	−4.111599
H	−3.281597	−1.936657	−3.732931
H	−1.129610	5.298831	−0.397659
H	0.292356	3.128724	3.581566
H	0.812459	−1.585592	3.686838
H	−0.332073	4.764298	1.896472
H	0.645627	0.764025	4.277259
H	0.870400	−3.403565	2.005755
H	−3.152634	−4.083897	0.494254
H	0.767643	−1.012259	−3.438967
H	0.354270	0.642123	−1.685041
H	0.970855	−3.426012	−2.843066
C	3.882225	0.808106	0.231824
F	3.680954	0.545671	1.522464

F	2.748849	1.297376	−0.271346	S	4.416910	−0.731330	−0.686389	O	4.447622	−0.282189	−2.091706
F	4.812324	1.758802	0.144708	O	5.731642	−1.031568	−0.089710	O	3.349726	−1.699400	−0.364958

7H$^{\cdot+}$

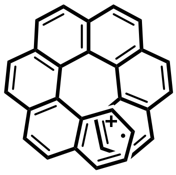

C	0.673209	−1.036134	1.115037	C	−2.863992	2.807221	0.485963	H	3.983310	−1.848689	1.573686
C	0.636107	0.081462	1.971349	C	−2.853296	−1.403068	−0.222584	H	2.841685	−3.587883	0.310708
C	0.732614	−3.606588	−0.072080	C	−4.056315	−0.675938	−0.156383	H	1.718170	1.396128	3.277414
C	3.052503	−1.284834	1.674785	C	0.673260	1.036108	−1.115041	H	3.903188	0.229785	2.943849
C	1.890347	−1.772736	1.034386	C	0.636112	−0.081470	−1.971375	H	−1.668160	4.562685	0.770078
C	1.899365	−3.038149	0.378378	C	0.732774	3.606544	0.072106	H	−5.001164	1.206770	0.296928
C	1.778976	0.526208	2.619135	C	3.052570	1.284709	−1.674774	H	−3.816376	−3.287174	−0.722940
C	3.003618	−0.137854	2.444786	C	−0.488908	1.522616	−0.384039	H	−3.816232	3.287329	0.722940
C	−1.617507	0.728749	−0.037863	C	−0.488516	2.889036	0.044648	H	−5.001216	−1.206560	−0.296941
C	−1.617540	−0.728686	0.037853	C	1.890431	1.772654	−1.034375	H	−0.307231	−0.598325	−2.148488
C	−1.696026	3.508872	0.483867	C	1.899502	3.038059	−0.378352	H	0.733262	4.619348	0.482069
C	−4.056286	0.676107	0.156368	C	1.778964	−0.526258	−2.619162	H	3.983402	1.848519	−1.573661
C	−2.864115	−2.807107	−0.485963	C	3.003637	0.137744	−2.444793	H	2.841846	3.587751	−0.310675
C	−0.488644	−2.889025	−0.044635	C	−1.696180	−3.508811	−0.483855	H	1.718121	−1.396162	−3.277458
C	−0.488978	−1.522601	0.384038	H	−0.307214	0.598365	2.148445	H	3.903193	−0.229930	−2.943856
C	−2.853234	1.403183	0.222575	H	0.733060	−4.619395	−0.482034	H	−1.668361	−4.562627	−0.770057

7HH^{+}

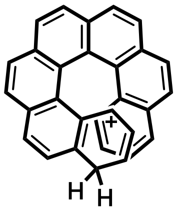

C	0.243112	1.576556	0.314730	C	3.957955	1.222245	−0.109921	C	−0.582258	−0.059804	−1.878697
C	−0.839489	0.979166	1.075575	C	3.255786	−2.374511	0.530422	C	−1.576128	−2.080628	−1.009334
C	2.447309	3.136715	−0.530140	C	−0.164003	−3.730830	0.018579	C	−2.887227	−0.450879	−2.405431
C	−1.233749	3.500402	−0.141383	C	1.517004	0.933745	0.053628	C	−2.860912	−1.694090	−1.636434
C	−3.212524	0.978284	1.722357	C	2.641132	1.764370	−0.195856	H	3.323465	3.745806	−0.766570
C	−1.733743	−0.706034	2.598716	C	3.051332	−0.988962	0.256019	H	−1.345802	4.501456	−0.565894
C	−0.669758	−0.146148	1.919371	C	4.155803	−0.090877	0.209066	H	−4.197142	1.449947	1.660672
C	0.065372	2.903602	−0.152465	C	0.925145	−2.821705	0.088987	H	−1.563605	−1.568059	3.248588
C	1.188182	3.658637	−0.603300	C	2.209741	−3.251365	0.534767	H	0.325455	−0.563675	2.065698
C	−2.130115	1.586991	1.040985	C	−1.404277	−3.356576	−0.451327	H	1.026988	4.686474	−0.937886
C	−2.307100	2.838907	0.375619	C	−3.027266	−0.162446	2.474611	H	−3.304926	3.284476	0.351254
C	1.739259	−0.498852	0.012842	C	−0.488228	−1.168097	−1.017093	H	4.806224	1.896177	−0.254813
C	0.726342	−1.472509	−0.294340	C	−1.766971	0.316077	−2.546262	H	4.267386	−2.710589	0.771473

H	−0.002657	−4.754369	0.367528
H	5.161935	−0.485383	0.370229
H	2.348158	−4.297149	0.818712
H	−2.234043	−4.066830	−0.457646
H	−3.867814	−0.619218	3.002948
H	−1.767197	1.212697	−3.168212
H	0.320242	0.520748	−2.077956
H	−3.828636	−0.162826	−2.881708
H	−3.283248	−2.518189	−2.243541
H	−3.623155	−1.599792	−0.828634

C7H$^{\cdot+}$

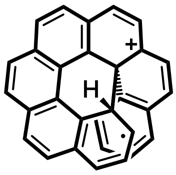

C	−0.003575	−1.186263	1.007691
C	0.625971	0.078503	1.469541
C	−1.323003	−3.572342	0.317395
C	2.000984	−2.433584	1.613683
C	0.660655	−2.418976	1.126753
C	−0.022343	−3.613437	0.754502
C	1.966499	−0.049510	2.022450
C	2.628399	−1.268547	2.048616
C	−1.058298	1.302474	0.071839
C	−1.857863	0.138444	0.084922
C	0.416929	3.646480	0.681273
C	−3.089758	2.630537	−0.174228
C	−3.959232	−1.007786	−0.423060
C	−1.986232	−2.328249	0.135988
C	−1.275980	−1.136564	0.405516
C	−1.689729	2.555796	0.061396
C	−0.923591	3.723174	0.393775
C	−3.234618	0.214336	−0.237860
C	−3.832079	1.492260	−0.380839
C	1.131948	0.472679	−0.996788
C	0.475835	−0.267722	−1.980244
C	2.549728	2.429831	0.492787
C	3.267355	−0.332503	−1.873318
C	0.439682	1.187451	0.142343
C	1.140244	2.451240	0.467770
C	2.548503	0.475223	−0.972430
C	3.230038	1.383511	−0.089133
C	1.203542	−1.030915	−2.893824
C	2.598187	−1.088084	−2.825321
C	−3.348437	−2.225333	−0.292608
H	−0.020573	0.597032	2.197195
H	−1.859339	−4.496116	0.086559
H	2.527525	−3.389695	1.670884
H	0.501175	−4.567863	0.850327
H	2.402710	0.807396	2.535467
H	3.632973	−1.325940	2.474018
H	0.969863	4.543803	0.969837
H	−3.564205	3.614516	−0.199227
H	−5.014469	−0.948265	−0.701623
H	−1.440922	4.684349	0.450177
H	−4.896629	1.557945	−0.618709
H	−0.611322	−0.246284	−2.055560
H	3.091167	3.267985	0.938850
H	4.359423	−0.335269	−1.825876
H	4.321372	1.340541	−0.038993
H	0.670622	−1.586262	−3.669526
H	3.158930	−1.705509	−3.530916
H	−3.906854	−3.145117	−0.484407

C7HH^{+}

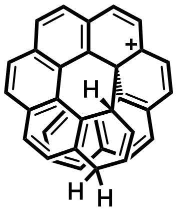

C	−0.514234	1.124828	−0.208169
C	−1.171316	0.435561	0.988698
C	0.704211	3.724415	−0.538243
C	−2.694557	2.310374	−0.530933
C	−3.263730	−0.340090	1.996492
C	−1.159127	−1.015108	2.930613
C	−0.474956	−0.277452	1.959495
C	−1.281767	2.355821	−0.571606
C	−0.626901	3.561129	−0.863966
C	−2.587728	0.436643	1.037582
C	−3.326070	1.329116	0.187032
C	1.852721	0.243620	−0.075727
C	1.363489	−1.076662	−0.365148
C	2.910783	2.828434	0.153401
C	4.026820	−0.725140	0.505898
C	1.606531	−3.485970	−0.251518
C	0.977589	1.345717	−0.107478
C	1.526752	2.642022	−0.117786
C	3.216978	0.430064	0.278016
C	3.721512	1.750351	0.404937
C	2.165727	−2.200420	−0.068141
C	3.509009	−1.985299	0.381343
C	0.316061	−3.617673	−0.717634
C	−2.552812	−1.074043	2.935728
C	0.088490	−1.225374	−0.955353
C	−1.942889	−0.225622	−2.028838
C	−0.457622	−2.493072	−1.093989
C	−2.486179	−1.446710	−2.126152
C	−0.605497	0.027188	−1.433000
C	−1.846132	−2.693286	−1.626632
H	1.159974	4.714884	−0.628561
H	−3.266088	3.116642	−0.996716
H	−4.356830	−0.338386	1.999834

H	−0.585829	−1.545845	3.694678
H	0.613465	−0.256379	1.991734
H	−1.216145	4.413101	−1.209951
H	−4.418458	1.301436	0.230523
H	3.312247	3.844547	0.157516
H	5.067593	−0.581297	0.806065
H	2.205420	−4.369300	−0.015377
H	4.773660	1.893166	0.662846
H	4.130734	−2.858200	0.597975
H	−0.120995	−4.613748	−0.830909
H	−3.078723	−1.669719	3.685079
H	−2.454335	0.619534	−2.492201
H	−3.445674	−1.557894	−2.641585
H	0.022499	0.500086	−2.210239
H	−1.835998	−3.445665	−2.435597
H	−2.477436	−3.147147	−0.838661

7H-IM1

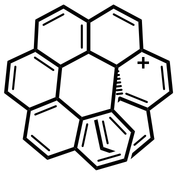

C	0.620771	−1.319842	−0.717790
C	−0.599931	−0.694180	−1.097481
C	3.015562	−2.616580	0.067332
C	−0.097243	−3.368759	−1.845230
C	0.852711	−2.682646	−1.054555
C	2.051363	−3.316568	−0.601156
C	−1.471564	−1.373242	−1.927729
C	−1.213485	−2.707580	−2.306779
C	0.343063	1.471606	−0.313595
C	1.549741	0.838542	0.025479
C	−1.878939	2.678243	−1.594886
C	1.506256	3.630859	−0.271863
C	3.859210	0.951004	0.826725
C	2.865002	−1.215120	0.296502
C	1.661709	−0.585861	−0.069419
C	0.354268	2.852332	−0.584073
C	−0.749525	3.413111	−1.276465
C	2.685051	1.621494	0.384656
C	2.620146	3.035481	0.259494
C	−1.418576	0.149000	0.995012
C	−0.591266	0.005725	2.101182
C	−3.372784	0.864602	−0.975312
C	−3.205641	−0.953524	2.248133
C	−0.928627	0.665017	−0.394440
C	−2.064414	1.406829	−1.049436
C	−2.759436	−0.303946	1.084085
C	−3.693797	0.000072	0.034770
C	−1.061383	−0.613297	3.266564
C	−2.357432	−1.118240	3.336346
C	3.935402	−0.417982	0.804165
H	3.937781	−3.107081	0.388655
H	0.082424	−4.414933	−2.105176
H	2.191574	−4.377490	−0.823462
H	−2.383813	−0.903452	−2.291966
H	−1.923556	−3.216321	−2.963030
H	−2.703161	3.149861	−2.134247
H	1.480833	4.705758	−0.465389
H	4.712490	1.547676	1.158061
H	−0.695539	4.465090	−1.572133
H	3.492904	3.631211	0.536997
H	0.428482	0.387437	2.093078
H	−4.147106	1.260082	−1.637403
H	−4.237552	−1.311835	2.287133
H	−4.718706	−0.368445	0.131538
H	−0.395639	−0.688805	4.129866
H	−2.710379	−1.617205	4.241420
H	4.851681	−0.920843	1.124637

7H-TS1

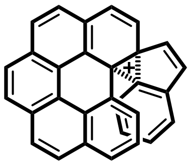

C	1.003582	−1.405439	−0.426672
C	−0.273767	−1.132780	−0.994683
C	3.540177	−1.990495	0.692542
C	0.720377	−3.775108	−0.953684
C	1.482647	−2.744390	−0.367074
C	2.747875	−3.007557	0.246162
C	−0.940374	−2.162304	−1.651953
C	−0.445974	−3.474950	−1.630187
C	0.181932	1.316283	−0.720907
C	1.482157	1.010352	−0.251311
C	−2.469578	2.025729	−1.441888
C	0.789401	3.692842	−0.774081
C	3.672411	1.745183	0.568606
C	3.137671	−0.629496	0.529102
C	1.862401	−0.344599	−0.007297
C	−0.169839	2.661806	−0.969573
C	−1.498258	2.981575	−1.374809
C	2.404735	2.060547	0.008562
C	2.033390	3.400558	−0.282163
C	−1.755737	0.268738	0.685777
C	−1.119833	0.911798	1.750152
C	−3.260722	−0.334057	−1.346778
C	−3.179001	−0.929809	2.236544
C	−0.820576	0.245222	−0.821501
C	−2.173587	0.658232	−1.163713
C	−2.836720	−0.616016	0.921629
C	−3.610624	−1.007851	−0.244782
C	−1.510558	0.613042	3.055845
C	−2.512342	−0.323975	3.301516
C	4.018428	0.446150	0.835176

H	4.515122	−2.198350	1.140368
H	1.089215	−4.802799	−0.904681
H	3.079934	−4.045851	0.325219
H	−1.860804	−1.967294	−2.196723
H	−0.999157	−4.260993	−2.149176
H	−3.495394	2.280526	−1.712401
H	0.501061	4.724251	−0.988662
H	4.368475	2.560165	0.780957
H	−1.737905	4.023118	−1.603511
H	2.756705	4.198290	−0.096476
H	−0.295728	1.605639	1.591420
H	−3.716964	−0.446521	−2.333389
H	−4.005649	−1.621329	2.416679
H	−4.400982	−1.758351	−0.197054
H	−1.001353	1.109030	3.885188
H	−2.792173	−0.568582	4.328857
H	4.997438	0.213662	1.262042

7H-IM2

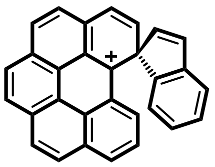

C	−1.371785	1.424154	−0.139385
C	−0.017797	1.012205	−0.351834
C	−4.048080	2.271813	0.276867
C	−0.693622	3.761336	−0.312409
C	−1.705710	2.807605	−0.122295
C	−3.064169	3.200983	0.089702
C	0.959469	2.020256	−0.536160
C	0.626166	3.366270	−0.516080
C	−0.717533	−1.332619	−0.173090
C	−2.070903	−0.912814	0.042704
C	1.913607	−2.333774	−0.587565
C	−1.467752	−3.667710	0.025052
C	−4.434929	−1.436537	0.448779
C	−3.744206	0.880035	0.265908
C	−2.395049	0.463858	0.055703
C	−0.429884	−2.743503	−0.179257
C	0.908876	−3.207893	−0.387171
C	−3.097911	−1.870724	0.242016
C	−2.765853	−3.244747	0.229478
C	2.788562	−0.306031	0.329299
C	2.810532	−0.295273	1.716267
C	2.299121	−0.457836	−1.984725
C	5.021528	0.644628	0.208873
C	0.304164	−0.381711	−0.372950
C	1.722147	−0.846723	−0.620448
C	3.882558	0.153676	−0.432640
C	3.543217	0.043869	−1.846388
C	3.949489	0.211303	2.350099
C	5.040820	0.671251	1.604299
C	−4.749386	−0.100247	0.459565
H	−5.083357	2.581164	0.438363
H	−0.954851	4.822748	−0.296795
H	−3.298827	4.268348	0.098937
H	1.999294	1.749650	−0.695234
H	1.406474	4.116196	−0.659762
H	2.939246	−2.676970	−0.742754
H	−1.231182	−4.733737	0.019305
H	−5.212023	−2.190040	0.598816
H	1.096971	−4.283640	−0.374933
H	−3.561225	−3.978535	0.385070
H	1.960419	−0.660807	2.297624
H	1.762567	−0.668064	−2.910481
H	5.876056	1.003334	−0.370113
H	4.198113	0.345872	−2.665723
H	3.987010	0.248864	3.441675
H	5.920876	1.058925	2.123759
H	−5.779678	0.226101	0.618969

7H-TS2

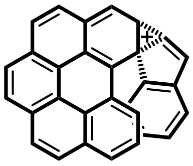

C	1.366124	1.470441	0.117710
C	0.011129	1.036618	0.226227
C	4.047658	2.369908	−0.092753
C	0.657363	3.779153	0.489850
C	1.684610	2.856514	0.226690
C	3.047466	3.275874	0.092098
C	−0.963994	1.987076	0.557581
C	−0.643638	3.340275	0.679669
C	0.777749	−1.319964	−0.024627
C	2.129341	−0.870703	−0.082189
C	−1.881080	−2.302782	−0.028730
C	1.630245	−3.667215	−0.155409
C	4.518331	−1.339306	−0.291551
C	3.764395	0.969124	−0.147560
C	2.418649	0.525658	−0.052698
C	0.523624	−2.749666	−0.071534
C	−0.766398	−3.218199	−0.061185
C	3.192617	−1.807739	−0.194887
C	2.905958	−3.214105	−0.217671
C	−2.847651	−0.110996	−0.439390
C	−3.086089	0.595931	−1.610383
C	−2.308676	−1.846589	1.443959
C	−5.103969	0.340537	0.351538
C	−0.287949	−0.377761	0.042521
C	−1.608603	−0.873133	−0.117767
C	−3.860815	−0.270009	0.528792
C	−3.402623	−1.071864	1.648104
C	−4.339994	1.192709	−1.785532
C	−5.335899	1.072613	−0.813557
C	4.795348	0.011485	−0.273193
H	5.086681	2.696138	−0.183895
H	0.903948	4.840740	0.572205
H	3.268411	4.344439	0.155069

H	−1.990982	1.679679	0.742284
H	−1.431436	4.053923	0.931500
H	−2.782726	−2.653142	−0.547921
H	1.408384	−4.736412	−0.177684
H	5.328560	−2.066587	−0.383834
H	−0.964469	−4.291490	−0.111812
H	3.743119	−3.912645	−0.290711
H	−2.305369	0.705996	−2.366037
H	−1.715718	−2.290471	2.245747
H	−5.878189	0.243077	1.116457
H	−3.860179	−1.005447	2.639404
H	−4.534788	1.771832	−2.691474
H	−6.303275	1.556983	−0.966048
H	5.828653	0.360082	−0.347982

7H-TS2'

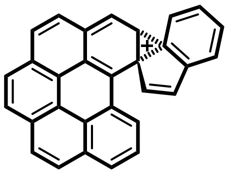

C	−1.767876	1.313143	−0.056092
C	−0.374716	1.339153	−0.369039
C	−4.535021	1.300591	0.568506
C	−1.780646	3.752009	0.094109
C	−2.475583	2.532343	0.159811
C	−3.876079	2.487739	0.457439
C	0.281391	2.579325	−0.367964
C	−0.415345	3.767691	−0.147966
C	−0.370214	−1.143484	−0.431736
C	−1.755138	−1.153555	−0.080844
C	2.346448	−1.237836	−1.237837
C	−0.422401	−3.640873	−0.405737
C	−3.817321	−2.369612	0.405370
C	−3.842887	0.061144	0.397224
C	−2.457719	0.074987	0.076965
C	0.306280	−2.417014	−0.607967
C	1.627510	−2.450646	−0.991913
C	−2.443684	−2.383782	0.089164
C	−1.736852	−3.622325	−0.071961
C	2.983339	−0.470411	0.077811
C	2.932072	−1.110836	1.324956
C	2.378371	1.123005	−1.842966
C	4.785962	0.956274	0.855928
C	0.305803	0.084427	−0.637076
C	1.642853	0.032230	−1.148759
C	3.869553	0.616278	−0.140310
C	3.601198	1.379232	−1.351074
C	3.816361	−0.716680	2.320876
C	4.758146	0.291272	2.079860
C	−4.498724	−1.179517	0.551954
H	−5.601982	1.273963	0.803479
H	−2.326361	4.684204	0.260971
H	−4.404264	3.433355	0.603675
H	1.358668	2.626279	−0.516202
H	0.127946	4.715367	−0.159064
H	3.172219	−1.300792	−1.955745
H	0.112949	−4.583957	−0.535341
H	−4.338628	−3.321766	0.530159
H	2.128526	−3.407521	−1.154276
H	−2.286174	−4.555396	0.076126
H	2.246656	−1.942972	1.496163
H	1.939437	1.569725	−2.739030
H	5.484267	1.778521	0.682682
H	4.305134	2.100274	−1.768763
H	3.791221	−1.223082	3.288348
H	5.463785	0.572231	2.865272
H	−5.563884	−1.184066	0.797146

7H-IM3

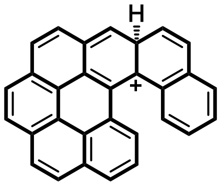

C	−1.474848	1.404391	−0.207407
C	−0.120980	1.040338	−0.455557
C	−4.157785	2.161424	0.304409
C	−0.972002	3.711715	−0.828645
C	−1.890960	2.760666	−0.351207
C	−3.246003	3.111562	−0.045968
C	0.724616	1.999634	−1.020655
C	0.304120	3.319221	−1.201565
C	−0.720764	−1.347846	−0.100333
C	−2.080587	−0.971309	0.080082
C	1.979819	−2.180613	−0.136418
C	−1.455375	−3.735714	−0.078965
C	−4.411037	−1.569465	0.514686
C	−3.790937	0.779305	0.347802
C	−2.445329	0.409067	0.107543
C	−0.407308	−2.755482	−0.164620
C	0.901867	−3.136281	−0.325775
C	−3.082735	−1.967955	0.251098
C	−2.738392	−3.355088	0.145173
C	2.759898	0.114150	0.342580
C	2.606191	1.264267	1.141694
C	3.317027	−2.473944	−0.740152
C	5.154035	0.571839	0.237050
C	0.301759	−0.341961	−0.191081
C	1.635826	−0.741898	−0.020752
C	4.068964	−0.266798	−0.065241
C	4.282034	−1.541622	−0.725452
C	3.695807	2.054651	1.466404
C	4.971310	1.724754	0.986871
C	−4.750992	−0.236184	0.574202
H	−5.194059	2.433128	0.519983
H	−1.290829	4.751147	−0.940456
H	−3.538703	4.161931	−0.121839

H	1.733326	1.724982	−1.327692	H	1.162208	−4.187126	−0.481489	H	5.269093	−1.755835	−1.142751
H	0.995746	4.044977	−1.635652	H	−3.532815	−4.097853	0.249578	H	3.557730	2.934802	2.098464
H	2.154278	−2.457702	0.952855	H	1.617715	1.526290	1.521404	H	5.826812	2.361640	1.225004
H	−1.182249	−4.789987	−0.161519	H	3.483138	−3.482395	−1.125649	H	−5.785940	0.055912	0.770830
H	−5.171552	−2.339501	0.663442	H	6.152647	0.286645	−0.103322				

7H-IM3'

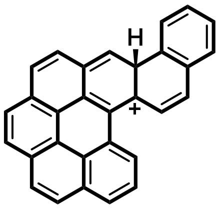

C	−2.344638	1.152264	0.037411	C	1.763612	−1.935506	0.037840	H	−5.392977	2.756464	−0.052071
C	−0.949334	1.424253	0.164366	C	−2.373446	−2.610912	0.017712	H	0.466488	2.937184	0.788392
C	−5.124261	0.641759	−0.206256	C	−1.452542	−3.702757	0.144168	H	−1.167438	4.756537	0.905997
C	−2.844747	3.522207	0.366200	C	3.763587	−0.331730	−0.045009	H	2.331805	−0.833150	−1.426459
C	−3.292796	2.215392	0.099906	C	4.674559	−1.303773	0.370685	H	0.594147	−4.306135	0.274224
C	−4.684850	1.923989	−0.065188	C	1.937237	1.771196	−0.727154	H	−4.124223	−3.873704	−0.088223
C	−0.569929	2.720916	0.533449	C	5.592753	1.279473	−0.174118	H	2.439715	−2.787507	0.127798
C	−1.505155	3.756395	0.624382	C	0.003480	0.326923	−0.026693	H	−1.852095	−4.718637	0.188445
C	−0.489896	−1.014179	0.029031	C	1.373998	0.528498	−0.302858	H	4.348143	−2.321356	0.590611
C	−1.889730	−1.272650	0.000451	C	4.225763	0.975986	−0.320758	H	1.276339	2.576865	−1.043933
C	2.309783	−0.631171	−0.309384	C	3.286071	1.972701	−0.754769	H	5.936181	2.292177	−0.400595
C	−0.115801	−3.479699	0.193580	C	6.020591	−0.981268	0.531927	H	3.677349	2.935848	−1.093235
C	−3.761032	−2.843384	−0.091360	C	6.484522	0.309101	0.254253	H	6.715884	−1.747112	0.884243
C	−4.203629	−0.451366	−0.174969	C	−4.647697	−1.794621	−0.198641	H	7.543268	0.549341	0.375531
C	−2.813992	−0.188539	−0.073940	H	−6.189978	0.424548	−0.312538	H	−5.720021	−1.990424	−0.281234
C	0.409239	−2.143973	0.090724	H	−3.572889	4.335924	0.413132				

O7H'

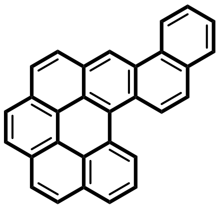

C	−3.711409	2.885225	0.200925	C	−1.381065	3.694528	−0.094955	C	−0.630973	−2.724240	−0.583976
C	−4.616979	1.855297	0.319389	C	−2.362777	−1.127249	−0.032265	C	2.323603	0.537380	−0.072859
C	−4.194481	0.506287	0.258064	C	−0.973260	−1.417963	−0.201575	C	1.815036	1.829150	−0.245375
C	−2.812486	0.221653	0.101097	C	0.005648	−0.336103	−0.061380	C	1.410147	−0.550596	0.115459
C	−1.874201	1.285909	−0.000014	C	−5.126313	−0.577644	0.311942	C	1.952450	−1.797797	0.582074
C	−2.329910	2.627907	0.032769	C	−4.710221	−1.864830	0.146734	C	3.291661	−2.002769	0.709038
C	−0.472823	0.998193	−0.097697	C	−3.328083	−2.177856	−0.066966	C	4.239246	−0.991452	0.348514
C	0.447991	2.089701	−0.187209	C	−2.911644	−3.490824	−0.349880	C	3.762227	0.290705	−0.036697
C	−0.050541	3.434934	−0.210090	C	−1.582927	−3.744703	−0.648588	C	5.628973	−1.238882	0.402124

C	6.536911	−0.251806	0.070828
C	6.071609	1.015086	−0.322261
C	4.714065	1.279648	−0.370079
H	−4.051122	3.923821	0.235663
H	−5.681932	2.068205	0.445296
H	0.670990	4.251275	−0.300768
H	−1.749769	4.723683	−0.090394
H	−6.185481	−0.349343	0.458086
H	−5.431003	−2.686904	0.153267
H	−3.654166	−4.292692	−0.376609
H	−1.269091	−4.749483	−0.942541
H	0.394387	−2.956333	−0.868343
H	2.491255	2.677198	−0.361657
H	1.270970	−2.582618	0.906851
H	3.664571	−2.951401	1.104472
H	5.973386	−2.230285	0.709458
H	7.610332	−0.453917	0.110377
H	6.784069	1.797628	−0.595516
H	4.385777	2.269832	−0.690190

O7H$^{\cdot+}$

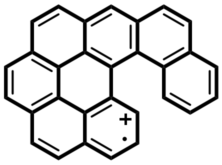

C	4.380913	1.641316	0.605525
C	4.756440	0.325175	0.633605
C	3.814645	−0.711142	0.363912
C	2.457285	−0.363956	0.116523
C	2.060855	0.994842	0.114845
C	3.033634	2.014764	0.326013
C	0.695637	1.338430	−0.092203
C	0.340466	2.716886	−0.193893
C	1.339251	3.714932	−0.041926
C	2.642416	3.373132	0.239879
C	4.200258	−2.073485	0.294037
C	3.295040	−3.041780	−0.071057
C	1.943050	−2.711464	−0.371496
C	1.508629	−1.363866	−0.224550
C	−0.300964	0.329100	−0.213190
C	−1.690590	0.727336	−0.150034
C	−2.006559	2.080633	−0.472949
C	−0.989407	3.047723	−0.491082
C	1.035542	−3.681800	−0.845176
C	−0.255622	−3.317229	−1.213446
C	−0.693650	−2.011057	−1.035015
C	0.145063	−1.020012	−0.465745
C	−2.771869	−0.121727	0.285491
C	−4.120775	0.289792	0.028829
C	−4.380767	1.580638	−0.526024
C	−3.365248	2.469783	−0.703057
C	−2.574665	−1.301127	1.054429
C	−3.637863	−2.087428	1.440633
C	−4.954260	−1.735700	1.078881
C	−5.188626	−0.558660	0.398510
H	5.113204	2.431265	0.788036
H	5.793574	0.049283	0.839008
H	1.051875	4.764296	−0.141549
H	3.396857	4.150057	0.384079
H	5.238983	−2.339370	0.504255
H	3.608220	−4.085553	−0.153593
H	−1.252806	4.092551	−0.676831
H	1.374923	−4.714397	−0.960072
H	−0.930825	−4.056439	−1.649206
H	−1.697207	−1.741048	−1.362778
H	−5.416262	1.864989	−0.728589
H	−3.562319	3.495932	−1.022335
H	−1.569755	−1.573851	1.376383
H	−3.458148	−2.980968	2.043000
H	−5.790284	−2.374947	1.373132
H	−6.209594	−0.246099	0.165451

O7H-TS$^{\cdot+}$

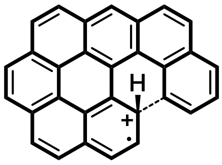

C	4.551702	1.295280	0.228487
C	4.781901	−0.063875	0.263437
C	3.713745	−0.989718	0.157485
C	2.391863	−0.496336	0.017204
C	2.154431	0.901527	0.000923
C	3.239803	1.810527	0.097840
C	0.819046	1.388641	−0.065573
C	0.567408	2.799610	−0.069464
C	1.690399	3.692982	−0.013726
C	2.966542	3.218760	0.073401
C	3.918634	−2.398839	0.170453
C	2.871808	−3.280350	0.021834
C	1.546931	−2.815091	−0.161078
C	1.304972	−1.405550	−0.148571
C	−0.257748	0.482087	−0.118238
C	−1.592598	0.969017	0.000312
C	−1.843457	2.361094	−0.124721
C	−0.747929	3.252682	−0.157192
C	0.452324	−3.705614	−0.371558
C	−0.801414	−3.231833	−0.641159
C	−1.091161	−1.822306	−0.641658
C	−0.003008	−0.912093	−0.314741
C	−2.674202	0.072787	0.241331
C	−3.994299	0.518054	0.049820
C	−4.231217	1.911162	−0.188254
C	−3.200512	2.806831	−0.213984
C	−2.400922	−1.311805	0.644157
C	−3.516352	−2.201613	0.706156
C	−4.801009	−1.769205	0.390860
C	−5.055709	−0.430650	0.104738

H	5.387204	1.995398	0.306598
H	5.800830	−0.445748	0.366118
H	1.499349	4.768810	−0.032880
H	3.809507	3.911952	0.130589
H	4.936884	−2.777261	0.292856
H	3.055180	−4.357350	0.028825
H	−0.941995	4.326511	−0.226823
H	0.649988	−4.780345	−0.380102
H	−1.607150	−3.924425	−0.895816
H	−1.740832	−1.497323	−1.463109
H	−5.262723	2.248744	−0.316073
H	−3.393927	3.875703	−0.333268
H	−1.686513	−1.431486	1.468590
H	−3.353905	−3.224642	1.053356
H	−5.633042	−2.475815	0.442273
H	−6.078685	−0.089707	−0.072264

CO7H$^{\cdot+}$

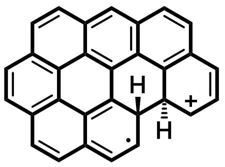

C	4.579508	1.315960	0.092288
C	4.820124	−0.035427	0.098556
C	3.749816	−0.974129	0.053817
C	2.413677	−0.492614	0.005309
C	2.165267	0.902651	0.013203
C	3.252205	1.822690	0.048656
C	0.829406	1.376488	−0.006434
C	0.578899	2.780473	−0.025190
C	1.683969	3.680437	−0.002580
C	2.974120	3.218089	0.039815
C	3.968224	−2.369197	0.051417
C	2.910427	−3.262597	0.002643
C	1.582985	−2.809769	−0.061105
C	1.325003	−1.407066	−0.070952
C	−0.253236	0.455225	−0.031607
C	−1.607546	0.949682	−0.016417
C	−1.838166	2.364668	−0.080133
C	−0.748774	3.239126	−0.083827
C	0.470659	−3.731731	−0.086165
C	−0.800753	−3.302369	−0.197228
C	−1.150074	−1.862856	−0.371893
C	−0.004774	−0.922949	−0.121763
C	−2.682879	0.066791	0.101144
C	−4.012728	0.564770	0.055950
C	−4.232073	1.976046	−0.054882
C	−3.183806	2.846317	−0.104596
C	−2.417938	−1.392849	0.366071
C	−3.618487	−2.258797	0.189921
C	−4.876801	−1.737553	0.103485
C	−5.091790	−0.339993	0.078712
H	5.410747	2.024631	0.122546
H	5.845285	−0.412600	0.135251
H	1.482192	4.754346	−0.019321
H	3.811510	3.919740	0.062814
H	4.994987	−2.741901	0.093292
H	3.102220	−4.338071	0.013615
H	−0.935246	4.316084	−0.119687
H	0.689608	−4.800216	−0.017785
H	−1.609393	−4.034643	−0.241395
H	−1.371401	−1.753732	−1.461677
H	−5.261472	2.340767	−0.087695
H	−3.353286	3.924137	−0.163210
H	−2.175700	−1.458581	1.456195
H	−3.488039	−3.341737	0.225735
H	−5.738231	−2.407839	0.051630
H	−6.110551	0.052498	0.032407

O8H$^{\cdot+}$

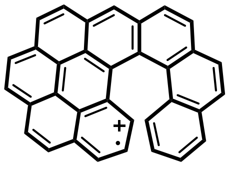

C	−2.394167	0.802792	−0.000450
C	−2.585343	−0.470665	0.678723
C	−2.295670	3.565550	−0.637502
C	−4.814325	0.841223	−0.482799
C	−4.040714	−2.382790	1.185638
C	−1.817760	−2.289274	2.106502
C	−1.601950	−1.065991	1.500747
C	−3.558355	1.512462	−0.424657
C	−3.475779	2.891777	−0.766462
C	−3.851488	−1.117628	0.583260
C	−4.941397	−0.448500	−0.055103
C	0.147189	0.735257	−0.324157
C	0.284989	−0.617146	−0.808325
C	0.109118	3.545328	−0.153899
C	2.574398	0.767679	0.149898
C	1.630713	−2.681564	−0.954230
C	−0.612266	−2.617966	−1.889042
C	−0.738290	−1.284142	−1.519232
C	−1.121267	1.425909	−0.217672
C	−1.094139	2.852644	−0.351088
C	1.336294	1.450279	−0.011715
C	3.667606	2.888145	0.712339
C	2.493656	3.567322	0.476557
C	1.307219	2.870437	0.127652
C	1.516085	−1.306832	−0.604242
C	3.873023	−1.319568	0.126752
C	2.648963	−0.627470	−0.081437
C	3.738484	1.481719	0.555671
C	4.947616	0.761984	0.783001
C	5.009976	−0.591857	0.585241
C	0.541906	−3.327604	−1.575195
C	−3.033686	−2.972165	1.922752
C	3.937235	−2.707496	−0.163230

C	2.853529	−3.364919	−0.692115
H	−2.241562	4.646960	−0.782939
H	−5.674172	1.384308	−0.882164
H	−5.012099	−2.872542	1.078583
H	−1.039064	−2.721122	2.739877
H	−0.660774	−0.546500	1.682369
H	−4.389541	3.412306	−1.062900
H	−5.901281	−0.966228	−0.129166
H	0.101448	4.638521	−0.175850
H	−1.425854	−3.106258	−2.429228
H	−1.639507	−0.740220	−1.801007
H	4.568492	3.431816	1.007052
H	2.457116	4.655846	0.563159
H	5.830067	1.314961	1.113790
H	5.943407	−1.133763	0.755770
H	0.633487	−4.383400	−1.842655
H	−3.191207	−3.946621	2.391450
H	4.873362	−3.241407	0.016716
H	2.919266	−4.428236	−0.935962

O8H-TS$^{\cdot+}$

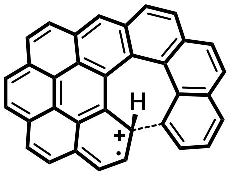

C	−2.436030	0.882851	−0.035344
C	−2.764318	−0.428616	0.442923
C	−2.140864	3.670321	−0.485522
C	−4.882187	1.225847	−0.356342
C	−4.389063	−2.235839	0.824604
C	−2.095818	−2.649504	1.364958
C	−1.725031	−1.356391	0.866402
C	−3.546705	1.727695	−0.343650
C	−3.372762	3.114941	−0.603664
C	−4.098756	−0.910806	0.417935
C	−5.157472	−0.060825	−0.012350
C	0.189404	0.703246	−0.203261
C	0.326162	−0.698617	−0.512306
C	0.229179	3.527403	−0.097957
C	2.670073	0.756131	0.125491
C	1.681291	−2.769800	−0.667472
C	−0.668107	−2.892057	−1.212623
C	−0.836719	−1.505435	−0.837187
C	−1.078154	1.426666	−0.159897
C	−0.992287	2.856035	−0.247798
C	1.410284	1.424151	0.013252
C	3.806489	2.906825	0.547236
C	2.628910	3.565784	0.376905
C	1.417473	2.849474	0.114005
C	1.576077	−1.365441	−0.409437
C	3.996403	−1.316598	0.077231
C	2.751045	−0.645419	−0.056102
C	3.858299	1.485927	0.420008
C	5.085145	0.792768	0.571324
C	5.154611	−0.568890	0.409131
C	0.531635	−3.508180	−1.095941
C	−3.397538	−3.099063	1.294766
C	4.052875	−2.713867	−0.148141
C	2.927031	−3.415130	−0.519061
H	−1.995481	4.748398	−0.587004
H	−5.682808	1.910296	−0.646640
H	−5.428523	−2.571234	0.780077
H	−1.308941	−3.284719	1.778434
H	−0.885902	−0.912323	1.407221
H	−4.250456	3.723024	−0.832309
H	−6.177409	−0.451123	−0.039270
H	0.232631	4.619960	−0.128068
H	−1.549255	−3.434192	−1.562692
H	−1.605031	−0.995947	−1.422168
H	4.729418	3.449251	0.765327
H	2.578948	4.655215	0.444738
H	5.982725	1.367431	0.812367
H	6.106922	−1.093296	0.519697
H	0.652244	−4.564539	−1.347918
H	−3.659689	−4.102887	1.635663
H	5.011221	−3.227758	−0.039165
H	2.985933	−4.489069	−0.710977

CO8H$^{\cdot+}$

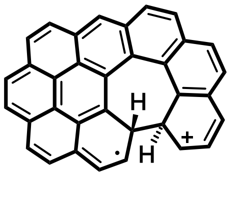

C	−2.450149	0.864514	−0.106743
C	−2.818947	−0.431020	0.363546
C	−2.097407	3.650776	−0.585540
C	−4.882338	1.276177	−0.461260
C	−4.514464	−2.144829	0.893695
C	−2.212563	−2.692764	1.301698
C	−1.751103	−1.405027	0.730016
C	−3.538070	1.738687	−0.458646
C	−3.332178	3.112072	−0.746956
C	−4.165884	−0.853012	0.408542
C	−5.196057	0.019376	−0.040239
C	0.195593	0.665833	−0.134908
C	0.345371	−0.733692	−0.399195
C	0.229587	3.492158	−0.050433
C	2.684912	0.752565	0.167517
C	1.738585	−2.767739	−0.682364
C	−0.642608	−2.983144	−0.993059
C	−0.856564	−1.572783	−0.605652
C	−1.080220	1.392936	−0.166034
C	−0.980726	2.821608	−0.270508
C	1.416904	1.400204	0.083986

C	3.798035	2.905832	0.644034
C	2.608675	3.550295	0.491683
C	1.413991	2.821444	0.200319
C	1.613436	−1.370922	−0.387051
C	4.052182	−1.283792	−0.006504
C	2.788221	−0.637626	−0.067348
C	3.868929	1.494638	0.463590
C	5.114397	0.821921	0.554483
C	5.206618	−0.526775	0.324035
C	0.577793	−3.542821	−1.032809
C	−3.535015	−3.038672	1.355784
C	4.130432	−2.664343	−0.290758
C	3.001115	−3.382515	−0.629787
H	−1.927152	4.723269	−0.702774
H	−5.663330	1.970307	−0.780234
H	−5.570655	−2.422661	0.916161
H	−1.442289	−3.375937	1.668017
H	−1.071108	−0.946762	1.469178
H	−4.190739	3.728106	−1.022515
H	−6.231414	−0.329840	−0.028330
H	0.229971	4.585178	−0.071168
H	−1.535910	−3.564529	−1.234476
H	−1.472820	−1.122365	−1.403007
H	4.713438	3.455285	0.875020
H	2.540353	4.636779	0.585072
H	6.005446	1.404884	0.798753
H	6.171191	−1.036853	0.382709
H	0.715616	−4.589586	−1.313449
H	−3.833812	−4.006160	1.766976
H	5.104183	−3.158837	−0.248596
H	3.076125	−4.447631	−0.860146

O8H-TS'$^{\cdot+}$

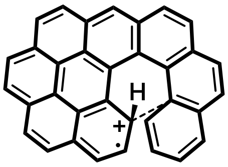

C	−2.207239	1.042117	−0.012506
C	−2.327224	−0.432994	0.280287
C	−1.963608	3.846667	−0.216977
C	−4.579768	1.178237	−0.603453
C	−3.952659	−2.294509	0.511371
C	−2.100678	−2.107413	2.076190
C	−1.759988	−0.890152	1.578247
C	−3.325514	1.825957	−0.316747
C	−3.179658	3.242862	−0.406651
C	−3.680581	−0.993694	0.044063
C	−4.709931	−0.182542	−0.502137
C	0.245701	0.825452	−0.167880
C	0.125561	−0.546089	−0.505260
C	0.501773	3.615382	0.068354
C	2.673319	0.577977	0.020925
C	1.114096	−2.793109	−0.621477
C	−1.260737	−2.531529	−1.095845
C	−1.196427	−1.089642	−0.883804
C	−0.926237	1.637453	−0.064301
C	−0.789429	3.054467	−0.065614
C	1.519024	1.404237	0.006015
C	4.061726	2.570074	0.395360
C	2.957585	3.372521	0.347580
C	1.645911	2.822257	0.158170
C	1.252238	−1.380900	−0.429753
C	3.675425	−1.659769	−0.080433
C	2.534524	−0.821086	−0.152664
C	3.956321	1.149610	0.239486
C	5.081458	0.292812	0.301975
C	4.944579	−1.071161	0.152033
C	−0.170779	−3.333845	−0.941799
C	−3.131581	−2.880846	1.457566
C	3.502094	−3.059396	−0.256208
C	2.262906	−3.608218	−0.513056
H	−1.872912	4.935299	−0.224990
H	−5.426794	1.793835	−0.915313
H	−4.875730	−2.789829	0.197144
H	−1.605385	−2.490554	2.971764
H	−0.972880	−0.291211	2.041544
H	−4.069618	3.843701	−0.610584
H	−5.670529	−0.655923	−0.724228
H	0.600887	4.703572	0.110399
H	−2.203379	−2.955479	−1.444650
H	−1.594151	−0.562465	−1.768033
H	5.052637	3.003333	0.551591
H	3.056159	4.455392	0.458262
H	6.067956	0.729863	0.474679
H	5.822871	−1.719764	0.203869
H	−0.248472	−4.409172	−1.120100
H	−3.353273	−3.883356	1.831114
H	4.380400	−3.707220	−0.190232
H	2.158988	−4.687144	−0.648579

CO8H'$^{\cdot+}$

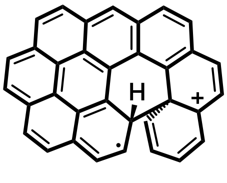

C	−2.186183	1.036186	−0.079972
C	−2.298719	−0.458785	0.193274
C	−1.928089	3.846367	−0.271930
C	−4.538679	1.201461	−0.722212
C	−4.037013	−2.251997	0.500375
C	−2.285464	−1.979203	2.171689
C	−1.844422	−0.836777	1.597409
C	−3.300243	1.834232	−0.405229
C	−3.144019	3.252576	−0.480555

C	−3.696501	−0.988608	−0.045527
C	−4.691770	−0.169119	−0.599440
C	0.257995	0.795069	−0.101431
C	0.131277	−0.569711	−0.405875
C	0.516982	3.592823	0.086213
C	2.691477	0.557677	0.062096
C	1.143346	−2.801641	−0.629147
C	−1.265986	−2.584689	−0.955113
C	−1.221906	−1.111532	−0.772546
C	−0.915047	1.622257	−0.072103
C	−0.764042	3.044440	−0.085867
C	1.537280	1.378337	0.065549
C	4.079416	2.545102	0.437569
C	2.967432	3.346000	0.394109
C	1.665023	2.792714	0.202753
C	1.273637	−1.398465	−0.404669
C	3.706737	−1.669146	−0.123702
C	2.556735	−0.837237	−0.146148
C	3.977235	1.133506	0.267151
C	5.112267	0.279005	0.290243
C	4.980710	−1.076315	0.106062
C	−0.161740	−3.357390	−0.900299
C	−3.319014	−2.769182	1.556135
C	3.541842	−3.055113	−0.341908
C	2.293676	−3.606416	−0.585987
H	−1.826434	4.934051	−0.284222
H	−5.376778	1.819939	−1.051687
H	−4.959610	−2.737750	0.168730
H	−1.883524	−2.292876	3.138855
H	−1.080619	−0.220734	2.077336
H	−4.026473	3.859664	−0.697817
H	−5.665025	−0.620033	−0.815120
H	0.623523	4.680490	0.127896
H	−2.227580	−3.028116	−1.216883
H	−1.450457	−0.692448	−1.780140
H	5.068656	2.982789	0.591214
H	3.063742	4.429232	0.503402
H	6.098247	0.719639	0.456286
H	5.862130	−1.722474	0.126146
H	−0.229921	−4.431942	−1.087115
H	−3.615436	−3.717149	2.011285
H	4.424860	−3.699624	−0.319942
H	2.195319	−4.681213	−0.754023

O9H$^{\cdot+}$

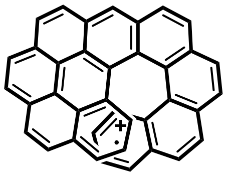

C	−0.731422	−3.135950	2.103801
C	0.380431	−1.081499	2.693793
C	−0.543870	−0.355208	1.970672
C	0.311358	−2.487946	2.731918
C	−2.362012	1.079136	−0.035035
C	−2.535690	−0.284749	0.425184
C	−2.252198	3.903725	−0.299257
C	−4.766433	1.174687	−0.536215
C	−3.868727	−2.351768	0.282385
C	−1.710061	−2.411245	1.382461
C	−1.577717	−0.996395	1.242544
C	−3.529554	1.843951	−0.344505
C	−3.443293	3.262026	−0.471816
C	−3.743528	−0.941573	0.088777
C	−4.841838	−0.187496	−0.415956
C	0.145360	1.020453	−0.506004
C	0.215798	−0.275534	−1.130532
C	0.185207	3.785991	0.023774
C	2.580806	0.928326	−0.115154
C	1.472543	−2.365476	−1.506417
C	−0.786703	−2.126295	−2.372920
C	−0.850701	−0.828443	−1.876886
C	−1.094515	1.718389	−0.241718
C	−1.045563	3.147318	−0.186630
C	1.363738	1.662994	−0.161278
C	3.759583	2.941236	0.634387
C	2.600389	3.675446	0.525189
C	1.378796	3.056418	0.150125
C	1.423325	−1.025539	−1.029936
C	3.794045	−1.194160	−0.368856
C	2.594702	−0.439756	−0.480205
C	3.779206	1.557429	0.326721
C	4.965399	0.776904	0.445893
C	4.969709	−0.554162	0.120682
C	0.343273	−2.907183	−2.155093
C	−2.856832	−3.071937	0.844355
C	3.793067	−2.552632	−0.781056
C	2.671026	−3.117357	−1.336855
H	−0.833629	−4.222201	2.173623
H	1.162436	−0.558251	3.249599
H	−0.493619	0.733466	1.992802
H	1.058649	−3.057756	3.289616
H	−2.190580	4.994320	−0.290312
H	−5.642881	1.761663	−0.820776
H	−4.790229	−2.840356	−0.044147
H	−4.362298	3.822794	−0.658700
H	−5.773685	−0.712742	−0.641044
H	0.205505	4.872454	0.144755
H	−1.630373	−2.528812	−2.937154
H	−1.729513	−0.221179	−2.091633
H	4.687184	3.422941	0.953283
H	2.602594	4.746842	0.739360
H	5.877017	1.263002	0.801667
H	5.885757	−1.142773	0.212668
H	0.384124	−3.936456	−2.520242
H	−2.936072	−4.156571	0.953397
H	4.710292	−3.136636	−0.674026
H	2.686702	−4.156506	−1.674750

O9H-TS$^{\cdot+}$

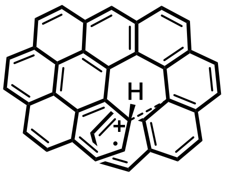

C	−1.442361	−2.978459	2.247269
C	−0.318320	−1.015386	3.066363
C	−0.889955	−0.250140	2.051214
C	−0.569844	−2.388400	3.149084
C	−2.065851	1.356866	−0.206775
C	−2.260237	−0.132271	−0.119371
C	−1.688060	4.151941	−0.037037
C	−4.443260	1.689842	−0.692546
C	−3.916870	−1.962635	−0.320704
C	−2.058213	−2.211989	1.238704
C	−1.738962	−0.835516	1.107828
C	−3.150542	2.228485	−0.356977
C	−2.937865	3.635894	−0.260530
C	−3.623304	−0.589159	−0.457082
C	−4.630305	0.339726	−0.826029
C	0.374707	1.052338	−0.431629
C	0.188959	−0.265863	−0.922604
C	0.767235	3.771495	0.167205
C	2.770730	0.636174	−0.181555
C	1.048545	−2.544759	−1.246439
C	−1.272887	−2.067303	−1.812303
C	−1.144037	−0.675705	−1.400036
C	−0.756165	1.899829	−0.225245
C	−0.551495	3.294147	−0.031523
C	1.668185	1.531587	−0.153562
C	4.253172	2.490663	0.450479
C	3.201232	3.360466	0.453454
C	1.865813	2.914245	0.169351
C	1.257356	−1.173167	−0.888839
C	3.639212	−1.642420	−0.462272
C	2.555952	−0.728039	−0.499072
C	4.072937	1.099953	0.147360
C	5.138365	0.170427	0.173295
C	4.926613	−1.162693	−0.116777
C	−0.237323	−2.953267	−1.704930
C	−3.079707	−2.781049	0.401620
C	3.391921	−3.006448	−0.790499
C	2.141388	−3.441379	−1.168745
H	−1.682897	−4.042278	2.321020
H	0.329641	−0.531239	3.801127
H	−0.677210	0.817944	2.013205
H	−0.102003	−2.987302	3.934060
H	−1.543273	5.226147	0.100983
H	−5.267847	2.384078	−0.871455
H	−4.840468	−2.354988	−0.754687
H	−3.802157	4.298841	−0.349271
H	−5.609334	−0.052691	−1.113494
H	0.919702	4.839026	0.348519
H	−2.199747	−2.378161	−2.294457
H	−1.518937	0.000021	−2.183508
H	5.260536	2.843950	0.684468
H	3.358454	4.417167	0.683888
H	6.140096	0.522399	0.431940
H	5.761083	−1.868254	−0.090558
H	−0.365826	−3.987998	−2.032355
H	−3.285931	−3.851347	0.484702
H	4.225199	−3.712876	−0.747763
H	1.980368	−4.490620	−1.427242

CO9H$^{\cdot+}$

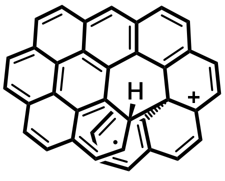

C	−1.997939	−2.745312	2.471133
C	−0.799982	−0.810776	3.247834
C	−1.151430	−0.124444	2.081773
C	−1.200019	−2.133400	3.431478
C	−1.953321	1.385962	−0.367082
C	−2.189678	−0.120362	−0.300431
C	−1.486991	4.176212	−0.172289
C	−4.287546	1.833350	−0.950850
C	−4.014653	−1.844066	−0.497487
C	−2.383682	−2.057197	1.308065
C	−1.907268	−0.739937	1.085484
C	−3.005704	2.308653	−0.560559
C	−2.743417	3.708372	−0.443747
C	−3.602060	−0.496255	−0.703418
C	−4.533740	0.472427	−1.078907
C	0.470409	0.971022	−0.367881
C	0.259171	−0.338230	−0.821901
C	0.919179	3.695050	0.179457
C	2.861470	0.517406	−0.086582
C	1.116268	−2.604681	−1.251167
C	−1.233846	−2.142207	−1.720910
C	−1.109494	−0.721143	−1.302007
C	−0.643566	1.873418	−0.281820
C	−0.389352	3.270330	−0.097470
C	1.776093	1.426522	−0.064318
C	4.368505	2.332886	0.583808
C	3.325639	3.223211	0.564185
C	2.000487	2.801188	0.242909
C	1.333403	−1.253313	−0.849445
C	3.712020	−1.756251	−0.447534
C	2.634103	−0.832344	−0.451668
C	4.170262	0.959333	0.258723
C	5.230963	0.013506	0.262844

C	5.007900	−1.300009	−0.073304
C	−0.196023	−3.004158	−1.700160
C	−3.368473	−2.631591	0.414518
C	3.457797	−3.092185	−0.829872
C	2.194434	−3.504440	−1.223626
H	−2.361406	−3.765306	2.623013
H	−0.206541	−0.302067	4.011509
H	−0.825959	0.909513	1.968091
H	−0.911616	−2.678187	4.333857
H	−1.303037	5.244753	−0.037095
H	−5.078488	2.557067	−1.159750
H	−4.917515	−2.202014	−1.001102
H	−3.578272	4.402721	−0.566262
H	−5.529233	0.135567	−1.382348
H	1.099647	4.758101	0.363549
H	−2.188138	−2.459325	−2.140310
H	−1.270665	−0.142108	−2.239629
H	5.375654	2.669275	0.841246
H	3.496225	4.277370	0.796915
H	6.233777	0.349182	0.537675
H	5.832803	−2.017191	−0.065707
H	−0.323376	−4.028339	−2.059064
H	−3.695414	−3.659045	0.593760
H	4.284131	−3.808137	−0.820342
H	2.027906	−4.541112	−1.524663

O9H-TS'$^{\cdot+}$

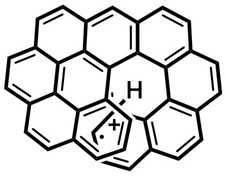

C	0.262886	−2.873192	−2.079480
C	−0.866610	−0.750870	−1.985864
C	0.289747	−0.107687	−1.374710
C	−0.875647	−2.096425	−2.299203
C	2.551001	0.881710	0.035286
C	2.598035	−0.454515	−0.493909
C	2.543086	3.599873	0.656124
C	4.965390	0.751347	0.407185
C	3.775703	−2.566791	−0.861705
C	1.455327	−2.335130	−1.534876
C	1.464720	−0.982404	−1.140235
C	3.760356	1.523890	0.393340
C	3.723160	2.902881	0.728960
C	3.790754	−1.212564	−0.422978
C	4.975118	−0.573672	0.061509
C	0.031996	0.758875	0.047806
C	−0.211212	−0.330853	1.082699
C	0.112578	3.678414	0.153179
C	−2.438500	0.989968	−0.107213
C	−1.672327	−2.225292	1.663976
C	0.510145	−2.001633	2.687505
C	0.739296	−0.812811	1.968740
C	1.313338	1.543843	0.184454
C	1.310164	2.941517	0.365907
C	−1.179198	1.618412	−0.074210
C	−3.480302	3.122688	−0.687768
C	−2.289036	3.763450	−0.470017
C	−1.101944	3.030433	−0.152512
C	−1.466089	−0.988456	0.998272
C	−3.804132	−1.033880	0.204583
C	−2.554722	−0.379093	0.294286
C	−3.597326	1.716531	−0.491165
C	−4.822893	1.014197	−0.637952
C	−4.916242	−0.323402	−0.329619
C	−0.652029	−2.725193	2.503009
C	2.631449	−3.125294	−1.368248
C	−3.932809	−2.348449	0.750973
C	−2.915294	−2.909294	1.472278
H	0.249770	−3.932593	−2.352034
H	−1.704555	−0.122013	−2.289368
H	0.575368	0.724986	−2.046662
H	−1.758466	−2.545474	−2.757796
H	2.526916	4.678935	0.828814
H	5.890216	1.242561	0.721294
H	4.691717	−3.156624	−0.779175
H	4.652200	3.409497	1.002097
H	5.900386	−1.152458	0.112154
H	0.142583	4.769539	0.202761
H	1.278563	−2.364071	3.374461
H	1.686470	−0.288768	2.101011
H	−4.369606	3.690046	−0.974399
H	−2.221604	4.851047	−0.552341
H	−5.704542	1.561728	−0.981295
H	−5.872104	−0.844107	−0.428841
H	−0.806422	−3.674826	3.021846
H	2.608139	−4.173898	−1.673831
H	−4.885308	−2.872584	0.638830
H	−3.047334	−3.887705	1.942037

CO9H'$^{\cdot+}$

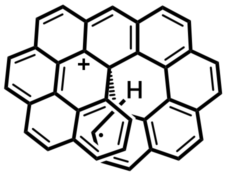

C	0.393400	−2.848990	−2.205476
C	−0.828949	−0.787880	−1.997900
C	0.303444	−0.130705	−1.335182
C	−0.772205	−2.095259	−2.414350
C	2.540106	0.889583	0.068722
C	2.614197	−0.449351	−0.455063
C	2.494592	3.607583	0.684498
C	4.948027	0.789292	0.480871
C	3.850848	−2.526451	−0.870733

C	1.545559	−2.316506	−1.596315
C	1.504114	−0.984257	−1.122323
C	3.740020	1.546067	0.446148
C	3.680998	2.924955	0.779456
C	3.825967	−1.185675	−0.387054
C	4.986506	−0.536736	0.122316
C	0.025360	0.727094	0.009349
C	−0.230786	−0.330372	1.087445
C	0.078538	3.662037	0.121920
C	−2.457849	0.955672	−0.151264
C	−1.697168	−2.205735	1.719220
C	0.476391	−1.948888	2.753442
C	0.709249	−0.781830	1.997939
C	1.297252	1.538128	0.178638
C	1.274236	2.938375	0.362762
C	−1.199944	1.589205	−0.128977
C	−3.507233	3.074263	−0.754630
C	−2.317268	3.721048	−0.532958
C	−1.130974	2.996754	−0.203089
C	−1.486087	−0.992043	1.012369
C	−3.824671	−1.056112	0.224117
C	−2.576444	−0.401798	0.292430
C	−3.620130	1.673475	−0.541535
C	−4.848505	0.965404	−0.672109
C	−4.940641	−0.361357	−0.332649
C	−0.682251	−2.678514	2.582524
C	2.738628	−3.086353	−1.437426
C	−3.957684	−2.350532	0.813464
C	−2.941094	−2.891587	1.551984
H	0.420532	−3.889358	−2.543467
H	−1.697466	−0.177858	−2.249283
H	0.597494	0.658721	−2.065808
H	−1.622713	−2.546515	−2.928029
H	2.462551	4.685538	0.862112
H	5.861365	1.289727	0.814027
H	4.778414	−3.097475	−0.786562
H	4.598531	3.442000	1.071400
H	5.921905	−1.097996	0.179687
H	0.094689	4.753326	0.170147
H	1.240652	−2.288443	3.456569
H	1.655179	−0.253500	2.122247
H	−4.396222	3.637249	−1.050925
H	−2.255151	4.808854	−0.618686
H	−5.730069	1.505912	−1.026906
H	−5.894974	−0.887297	−0.418254
H	−0.841698	−3.610638	3.130838
H	2.750626	−4.117270	−1.798334
H	−4.912550	−2.874265	0.720450
H	−3.076377	−3.852739	2.055277

CO8H'$_{PDI-CH3}$

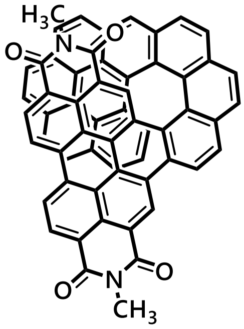

C	−2.612402	2.970229	0.058865
C	−1.389980	3.226327	−0.661285
C	−5.271339	2.296584	0.718480
C	−3.354879	5.235563	−0.530062
C	0.265176	4.895920	−1.351216
C	−3.646919	3.930922	−0.038787
C	−4.985098	3.567144	0.324927
C	−1.072252	4.570169	−0.982268
C	−2.081561	5.571142	−0.902077
C	−1.849475	0.970532	1.473495
C	−0.528853	1.466870	1.859045
C	−4.513801	0.041227	1.368137
C	−1.102951	−1.319608	2.046854
C	1.841164	0.993575	2.381320
C	1.037143	3.275781	2.332996
C	−0.252458	2.828807	2.044801
C	−2.858481	1.774249	0.853744
C	−4.220219	1.357225	0.985754
C	−2.148381	−0.389803	1.731920
C	−2.747468	−3.149722	2.050605
C	−3.757829	−2.270847	1.806453
C	−3.494732	−0.872194	1.625162
C	0.515573	0.533434	2.122605
C	1.283146	−1.793516	2.423822
C	0.238720	−0.865908	2.176472
C	−1.391323	−2.701125	2.175449
C	−0.330007	−3.608703	2.404607
C	0.970086	−3.169544	2.521707
C	2.086555	2.375018	2.456281
C	1.243827	3.941894	−1.358032
C	2.618994	−1.307269	2.591693
C	2.884307	0.029764	2.579482
C	−1.592024	−1.888497	−1.402079
C	−0.220659	−1.556707	−1.257355
C	0.160471	−0.179864	−1.225622
C	−0.821155	0.831557	−1.255585
C	−2.165574	0.462548	−1.548056
C	−2.545810	−0.851986	−1.603689
C	−2.006435	−3.228234	−1.361567
C	−1.060808	−4.236314	−1.198114
C	0.288624	−3.922537	−1.074441
C	0.738049	−2.594479	−1.097520
C	2.146643	−2.236644	−0.944133
C	2.517868	−0.861602	−0.991307
C	1.541129	0.169904	−1.126655
C	3.885966	−0.503772	−0.877859
C	4.258981	0.865997	−0.944711
C	3.308665	1.843215	−1.109714
C	1.926842	1.528488	−1.182525
C	3.150732	−3.193066	−0.747606
C	4.488288	−2.829088	−0.616098
C	4.866442	−1.492282	−0.687142
C	0.916410	2.562241	−1.198806
C	−0.439078	2.203003	−1.028607
O	−3.834338	−4.724010	−1.364852
C	6.291469	−1.127934	−0.544188
C	5.679112	1.255558	−0.809549

N	6.600593	0.231884	−0.612051
O	6.053857	2.412378	−0.858469
O	7.159527	−1.962562	−0.371118
C	7.987476	0.640519	−0.471867
N	−4.326930	−2.524699	−1.691591
C	−3.439171	−3.577611	−1.465873
C	−3.968169	−1.185580	−1.824987
C	−5.743012	−2.819896	−1.815118
O	−4.814710	−0.350338	−2.080607
H	−6.303997	1.977609	0.881049
H	−4.159685	5.974803	−0.558589
H	0.507770	5.938706	−1.571198
H	−5.777748	4.309776	0.202747
H	−1.830005	6.596418	−1.185053
H	−5.557397	−0.281278	1.411079
H	1.218125	4.346944	2.450903
H	−1.057295	3.558630	1.958390
H	−2.954029	−4.218543	2.150012
H	−4.789841	−2.620068	1.717191
H	−0.560516	−4.674392	2.484331
H	1.779694	−3.882477	2.699193
H	3.104047	2.726553	2.646467
H	2.275495	4.237182	−1.550022
H	3.421552	−2.032041	2.751877
H	3.903362	0.394578	2.733884
H	−2.920301	1.226451	−1.731083
H	−1.396525	−5.274352	−1.162536
H	0.999743	−4.738723	−0.946601
H	3.651778	2.877709	−1.138716
H	2.895140	−4.251366	−0.691505
H	5.257914	−3.587513	−0.460161
H	8.093141	1.333915	0.374215
H	8.593755	−0.254014	−0.300956
H	8.326202	1.154386	−1.382697
H	−5.889046	−3.887983	−1.628343
H	−6.313664	−2.227093	−1.086434
H	−6.101299	−2.564289	−2.822624
